# Targeting polycomb repressor complex 2‐mediated bivalent promoter epigenetic silencing of secreted frizzled‐related protein 1 inhibits cholangiocarcinoma progression

**DOI:** 10.1002/ctm2.1502

**Published:** 2023-12-04

**Authors:** Guanhua Wu, Qi Wang, Da Wang, Fei Xiong, Wenzheng Liu, Junsheng Chen, Bing Wang, Wenhua Huang, Xin Wang, Yongjun Chen

**Affiliations:** ^1^ Department of Biliary‐Pancreatic Surgery Tongji Hospital, Tongji Medical College, Huazhong University of Science and Technology Wuhan P. R. China; ^2^ Department of Emergency Tongji Hospital, Tongji Medical College, Huazhong University of Science and Technology Wuhan P. R. China; ^3^ Departement of Pediatric Surgery Wuhan Children's Hospital, Tongji Medical College, Huazhong University of Science and Technology Wuhan P. R. China

**Keywords:** bivalent promoter, DNMT3a/b, Histone modification, PRC2, SFRP1

## Abstract

**Background:**

Cholangiocarcinoma (CCA) refers to a collection of malignancies that are associated with a dismal prognosis. Currently, surgical resection is the only way to cure patients with CCA. Available systemic therapy is limited to gemcitabine plus cisplatin; however, this treatment is palliative in nature. Therefore, there is still a need to explore new effective therapeutic targets to intervene against CCA.

**Methods:**

We analyzed the expression of EZH2 and the prognosis of patients in CCA. The proliferation, migration and invasion of CCA cells after gene knockdown and overexpression were examined and validated by a xenograft model and a primary CCA mouse model with corresponding gene intervention. Targeting DNA methylation, and RNA‐sequencing‐based transcriptomic analysis in EZH2 and SUZ12 knockout CCA cells was performed. Bisulfite sequencing polymerase chain reaction (PCR), chromatin immunoprecipitation‐quantitative PCR (ChIP‐qPCR) and reverse‐ChIP assays were performed for research purposes.

**Results:**

Increased expression of EZH2 in CCA exhibited a significantly poorer prognosis. DNA hypomethylation of the promoter and increased mRNA levels of secreted frizzled‐related protein 1 (SFRP1) were observed in CCA cells following the inhibition of polycomb repressor complex 2 (PRC2), which was achieved through a knockout of EZH2, SUZ12 and EED, respectively, or treatment with GSK126 and GSK343. Targeting the SFRP1 promoter DNA hypermethylation with dCas9‐DNMT3a decreased the mRNA level of SFRP1. The expression of SFRP1 is regulated by both H3K27me3 and DNA methylation and H3K27me3 plays a crucial role in promoting SFRP1 promotor DNA methylation. GSK343 is a small molecule inhibitor that targets the catalytic activity of EZH2. It effectively inhibits the progression and development of subcutaneous xenografts and primary CCA mouse models.

**Conclusion:**

Overall, our data strongly suggested that targeting PRC2 promotes the expression of SFRP1, thereby inhibiting the progression of CCA.

**Key Points/Headlights:**

- Cholangiocarcinoma (CCA) exhibits elevated expression of EZH2, SUZ12 and EED, resulting in increased levels of H3K27me3.
- Targeting polycomb repressor complex 2 (PRC2) leads to the removal of H3K27me3 from the secreted frizzled‐related protein 1 (SFRP1) promoter and DNA hypomethylation, thereby activating the transcription of SFRP1.
- Inhibiting PRC2, including the use of EZH2 inhibitors, holds promise as a potential strategy for developing anti‐cancer drugs for CCA.

## Graphical abstract

Schematic diagrams illustrating the potential molecular mechanisms of polycomb repressor complex 2‐mediated bivalent promoter epigenetic silencing of secreted frizzled‐related protein 1, which promotes cholangiocarcinoma progression (top), along with the hypothesis of pharmacological intervention (bottom).

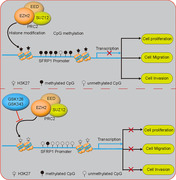

## 1 INTRODUCTION

Cholangiocarcinoma (CCA) refers to a collection of malignancies that are associated with a dismal prognosis.^1^ The incidence of CCA has been increasing in many countries globally.^2^, ^3^, ^4^ Currently, surgical resection is the only effective curative option. However, it is unfortunate that at the time of diagnosis, almost two‐thirds of CCA patients already have advanced disease. Additionally, even after resection, a significant percentage of patients (between 68% and 86%) experience tumour recurrence, either locally or distantly.^5^ Currently, the available systemic therapy for CCA patients is limited to gemcitabine + cisplatin, which remains the standard chemotherapy option. However, this treatment is palliative and offers little benefit.^6^ As a result, there is still a need for the exploration of novel drug targets for CCA therapeutic interventions.

The polycomb repressor complex 2 (PRC2) consists of three core factors: EZH2, SUZ12 and EED, which functions as a chromatin‐associated methyltransferase that catalyzes the mono‐, di‐ and trimethylation of lysine 27 on histone H3 (H3K27).^7^ PRC2 is the only identified methyltransferase with activity on H3K27, and EZH2 acts as the catalytic subunit of PRC2 but requires the physical presence of both EED and SUZ12 to generate catalytic activity.^8^ PRC2 is known to have oncogenic properties in both hematologic and solid tumours, as evidenced by multiple studies.^9^, ^10^, ^11^ Furthermore, its subunits have been found to be significantly dysregulated in various cancers such as prostate, breast, lung, bladder and pancreas.^12^, ^13^, ^14^ The role of PRC2 includes promoting cancer progression through multiple mechanisms. First, PRC2 catalyzes the methylation of histone H3 at lysine 27, known as H3K27me3, which has been linked to cancer metastasis and growth.^15^ Additionally, EZH2 has non‐histone methylase catalytic activity, which also contributes to cancer progression.^14^ There is currently insufficient evidence to determine whether PRC2 contributes to the progression of and plays a role in CCA.

Secreted frizzled‐related protein 1 (SFRP1) has been identified as a tumour suppressor gene that is commonly downregulated in human cancers.^16^, ^17^, ^18^ Over‐expression of SFRP1 can significantly inhibit the proliferation of tumour cells by negatively regulating β‐Catenin.^16^, ^19^ However, the expression of SFRP1 is highly regulated by DNA methylation.^18^, ^20^, ^21^ Generally, DNMTs regulate DNA methylation, with three common isoforms identified in humans: DNMT1, DNMT3a and DNMT3b. DNMT1 is primarily responsible for maintaining methylation, while DNMT3a and DNMT3b are involved in de novo methylation.^22^ DNMT3a/DNMT3b can actively introduce methylation into DNA to regulate gene expression, which plays a pivotal role in carcinogenesis.^23^

Clinical data and gain‐ and loss‐of‐function studies have yielded evidence suggesting that EZH2 plays an oncogenic role in CCA. Through transcriptome sequencing of CCA models with knockout of EZH2 and SUZ12, respectively, we identified SFRP1, a gene that plays a tumour suppressor role in CCA. The expression of SFRP1 is regulated by both H3K27me3 and DNA methylation and H3K27me3 plays a crucial role in promoting SFRP1 promotor DNA methylation. Additionally, we utilized GSK343, a small molecule inhibitor that targets the catalytic activity of EZH2, to impede the progression of CCA in subcutaneous xenograft and primary CCA models.

## 2 RESULTS

### 2.1 Increased EZH2 expression is associated with poor prognosis in CCA

**FIGURE 1:**
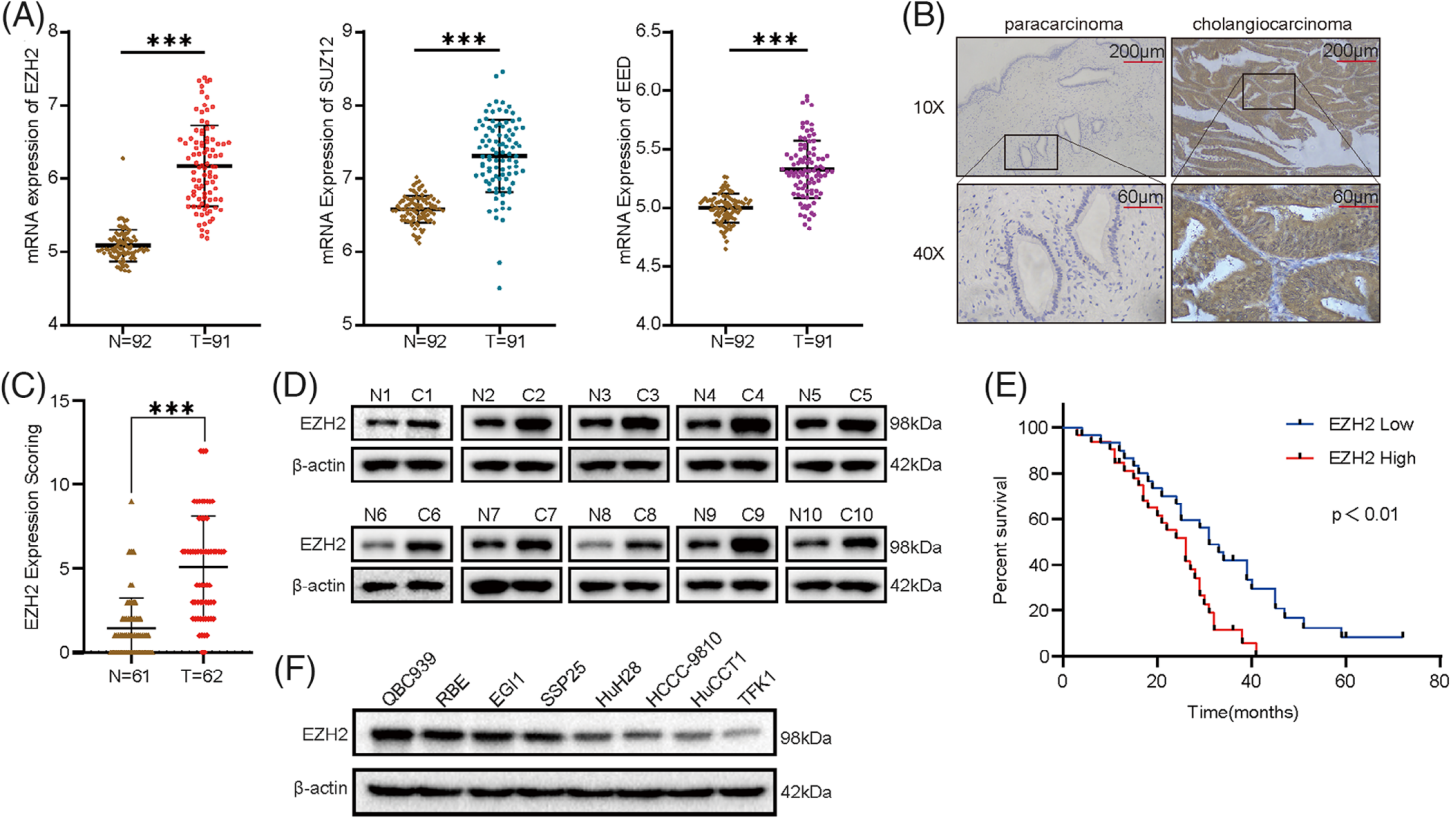
Increased EZH2 expression is associated with poor prognosis in cholangiocarcinoma (CCA). (A) The expression level of EZH2, SUZ12 and EED in CCA and adjacent tissue from GSE76297(‘T’ stands for tumour tissue (CCA) and ‘N’ stands for normal tissue adjacent to tumour tissue). (B, C) Immunohistochemical staining assay detected EZH2 in human CCA and adjacent tissue. The data are presented as a representative image (B) and summary graphs (C) (normal group, *n* = 61 and cancer group, *n* = 62). (D) Western blot analysis of EZH2 expression in 10 pairs of CCA tissue(C) and corresponding adjacent normal bile duct tissue(N). (E) The Kaplan‐Meier analysis was used to estimate the overall survival rate of CCA patients, which were stratified based on the median EZH2 expression (high and low expressing tumours; *n* = 62). (F) Western blot of EZH2 expression in CCA cell lines.

From the Gene Expression Omnibus (GEO) database, EZH2, SUZ12 and EED, the three core subunits of PRC2, were significantly upregulated in CCA tissues compared to adjacent tissues (Figure 1A). In human CCA tissue specimens, the protein expression level of EZH2 was significantly upregulated compared to the adjacent tissues (Figure 1B–D). Kaplan‐Meier survival analysis showed that higher expression levels of EZH2 in CCA patients had decreased overall survival (OS) and a poorer prognosis (Figure 1E). Based on the findings, it is highly probable that PRC2 functions as an oncoprotein complex in cases of CCA. To conduct the follow‐up study, we analysed the protein expression levels of EZH2 in the CCA cell lines (Figure 1F).

### 2.2 Inhibition of EZH2 impairs CCA progression in vitro and in vivo

**FIGURE 2:**
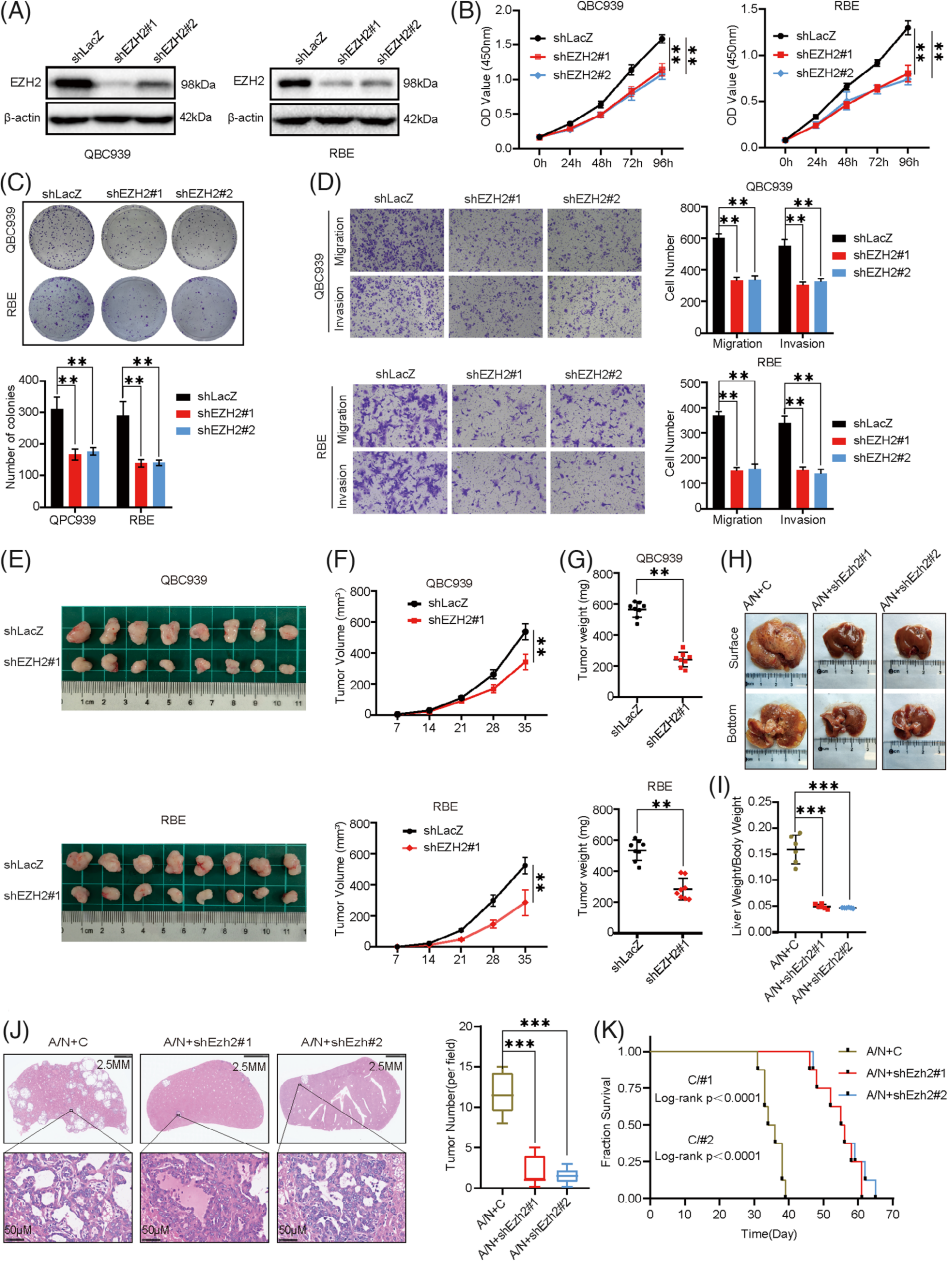
Inhibition of EZH2 impairs cholangiocarcinoma (CCA) progression in vitro and in vivo. (A) Knockdown of EZH2 in QBC939 and RBE cells was confirmed by western blot. (B) Proliferative ability of EZH2‐knockdown cells and control cells examined by Cell Counting Kit 8 (CCK‐8) assay. (C) Colony‐formation assay of EZH2‐knockdown and control cells (Top: representative images. Bottom: quantification data). (D) Cell migration assay Matrigel invasion assay of EZH2‐knockdown and control cells (Left panels: representative images. Right panels: quantification data). (E) Overview of tumours in transplanted xenografts with EZH2‐knockdown and control cells (*n* = 8). shEZH2#1 was used in this experiment. (F, G) Tumor volume (F) and weight (G) were measured. (H) Representative images of tumour burden in A/N+C, A/N+shEzh2#1 and A/N+shEzh2#2 mouse livers. (I) Ratios of liver weight to body weight in A/N+C, A/N+shEzh2#1 and A/N+shEzh2#2 mice. (J) H&E‐staining images of livers in A/N+C, A/N+shEzh2#1 and A/N+shEzh2#2 mice (Left: Representative H&E‐staining images. Right: Quantitative analysis of tumour number). (K) Survival curve of A/N+C, A/N+shEzh2#1 and A/N+shEzh2#2.

To investigate the role of EZH2 in CCA, we utilized two independent short hairpin RNA (shRNA) viruses to perform a knockdown of EZH2 in the QBC939 and RBE cell lines (Figure 2A). Loss of EZH2 inhibits cell growth and colony‐forming abilities of CCA cells (Figure 2B,C). In addition, the loss of EZH2 inhibits the migration and invasive abilities of CCA cells (Figure 2D). We then further verified the in vivo oncogenic function of EZH2 in CCA using a xenograft mouse model. Compared to controls, mice injected with EZH2‐ knockdown CCA cells (shEZH2#1) exhibited significantly slower tumour progression (Figure 2E). This was evident from the reduced tumour size and weight observed (Figure 2F,G). Aiming to further investigate the role of EZH2 in regulating CCA progression in vivo, we established a CCA primary tumour mouse model by hydrodynamic transfection of activated forms of Akt and NICD1 plasmids into C57BL/6 mice. The Ezh2 knockdown plasmid was co‐injected with the Akt/NICD1 plasmid to observe whether the knockdown of Ezh2 had an inhibitory effect on the progression of CCA in vivo. Notably, Akt/NICD1/Control (A/N+C) mice showed a large tumour load in the liver, while Akt/NICD1/ Ezh2 knockdown (A/N+shEzh2#1, A/N+shEzh2#2) mice showed only a few small lesions in the liver after 5 weeks of plasmid injection (Figure 2H). The ratio of liver weight to body weight was significantly lower in A/N+shEzh2#1, A/N+shEzh2#2 mice compared to A/N+C mice for tumour load (Figure 2I). Hematoxylin and eosin (H&E) staining showed that the number of tumour lesions was significantly decreased in A/N+shEzh2#1 and A/N+shEzh2#2 mice compared to A/N+C mice (Figure 2J). It was consistently observed that the survival outcomes of mice with Ezh2 knockdown were superior to those of the control group (Figure 2K). Histochemical analysis revealed positive staining for both A/N+shEzh2#1, A/N+shEzh2#2 and A/N+C transfection‐induced tumour cytokeratin 19, a classical biomarker for CCA and the knockdown effects of Ezh2 were also demonstrated by histochemical staining (Figure S1A). Overall, our findings illustrate the significant impact of Ezh2 knockdown in suppressing both the tumourigenesis and progression of CCA in vivo.

### 2.3 Overexpression of EZH2 promotes CCA progression in vitro and in vivo

**FIGURE 3:**
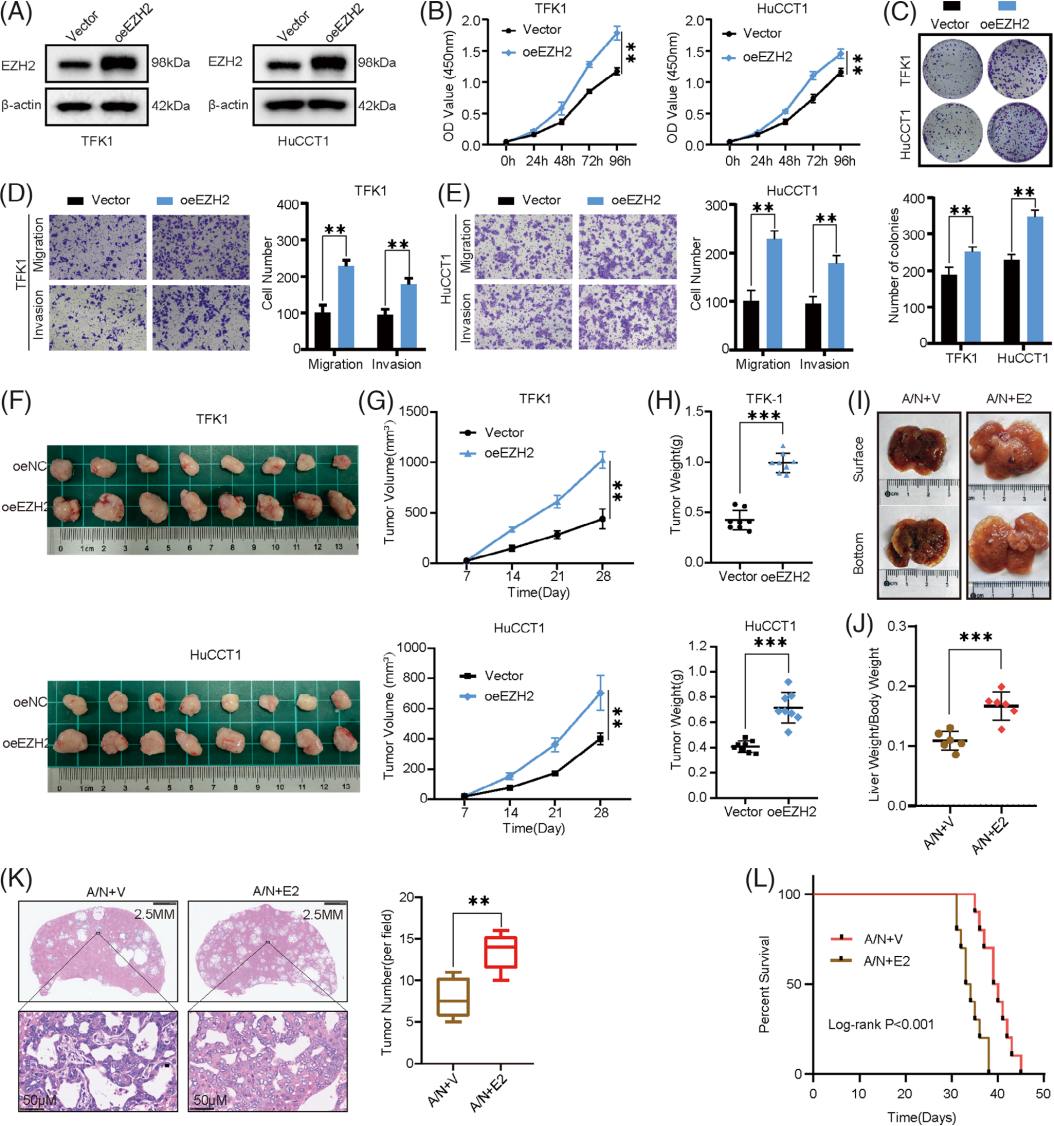
Overexpression of EZH2 promotes cholangiocarcinoma (CCA) progression in vitro and in vivo. (A) Overexpression of EZH2 in HuCCT1 and TFK1 cells was confirmed by western blot. (B) Proliferative ability of EZH2‐overexpression cells and control cells examined by Cell Counting Kit 8 (CCK‐8) assay. (C) Colony‐formation assay of EZH2‐ overexpression and control cells (Top: representative images. Bottom: quantification data). (D, E) Cell migration assay and Matrigel invasion assay of EZH2‐overexpression and control cells (Left: representative images. Right: quantification data). (F) Overview of tumours in transplanted xenografts with EZH2‐overexpression and control cells (*n* = 8). G, H. Tumor volume (G) and weight (H) were measured. (I) Representative overview of tumour burden in A/N+V and A/N+E2 mouse livers. J. Ratios of liver weight to body weight in A/N+C and A/N+E2 mice. (K) H&E‐staining images of livers in A/N+V and A/N+E2 mice (Left: Representative H&E‐staining images. Right: Quantitative analysis of tumour number). (L) Survival curve of A/N+C and A/N+E2 mice.

To further explore the role of EZH2 in CCA, we used lentivirus to overexpress EZH2 in HuCCT1 and TFK1 cell lines (Figure 3A). Overexpression of EZH2 significantly promoted cell growth and colony‐forming viability in two CCA cell lines (Figure 3B,C). Furthermore, overexpression of EZH2 promoted the migration and invasive ability of CCA cells (Figure 3D,E). Next, we utilized a nude mouse xenograft model to investigate the role of EZH2 in CCA. Cell lines with stably overexpressed EZH2 were established and subcutaneously injected into nude mice in two independent experimental groups with their own controls. Consistent with previous in vitro findings, EZH2 promoted xenograft tumour growth (Figure 3F), reflected by larger tumour mass and heavier tumour weight (Figure 3G,H). We established a CCA primary mouse model by hydrodynamic transfection of activated forms of Akt and NICD1 plasmids into C57BL/6 mice. The Ezh2 overexpression plasmid was co‐injected with the Akt/NICD1 plasmid to observe whether Ezh2 had a role in promoting CCA progression in vivo. Notably, Akt/NICD1+Ezh2 (A/N+E2) mice developed a large tumour load 4 weeks after plasmid injection, while Akt/NICD1/Vector (A/N+V) mice developed only a small number of small lesions in the liver (Figure 3I). The ratio of mouse liver weight to body weight showed a significantly increased tumour load in A/N+E2 compared to A/N+V mice (Figure 3J). H&E staining showed a significant increase in the number of tumour lesions in A/N+E2 mice compared to A/N+V mice (Figure 3K). The survival results were consistently poorer in Ezh2 overexpressing mice compared to controls (Figure 3L). Histological analysis showed positive staining for cytokeratin 19 in both A/N+E2 and A/N+V transfected induced tumours, and histochemical staining further demonstrated the overexpression effects of Ezh2 (Figure S2A). Overall, our data demonstrate the potential function of Ezh2 in promoting CCA tumourigenesis and progression in vivo.

### 2.4 PRC2 promotes transcriptional silencing of SFRP1

**FIGURE 4:**
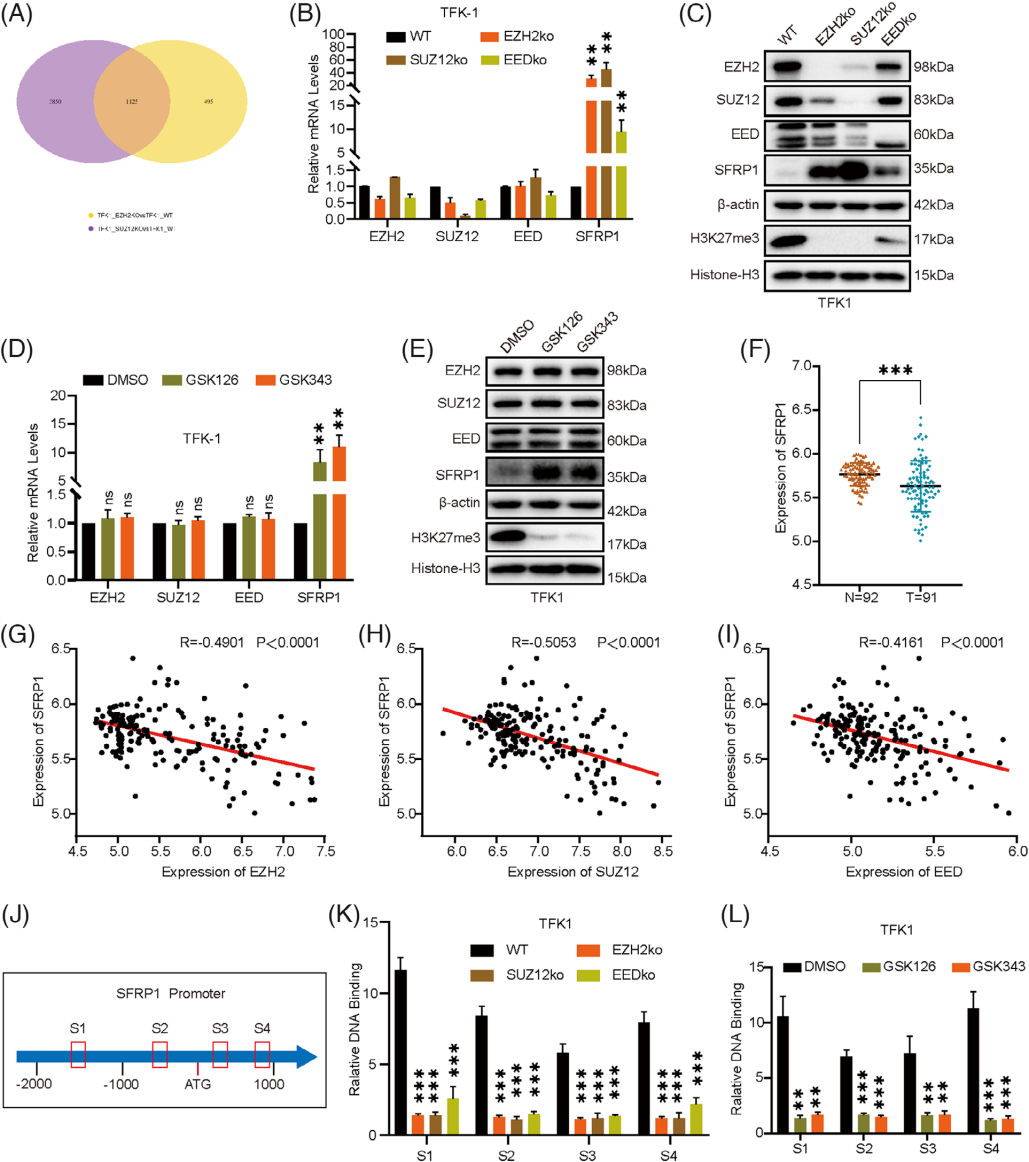
Polycomb repressor complex 2 (PRC2) promotes transcriptional silencing of secreted frizzled‐related protein 1 (SFRP1). (A) Venn diagram of RNA‐seq in EZH2 and SUZ12 knockout compared to control TFK1 cells. (B) The mRNA levels of SFRP1 in EZH2, SUZ12 and EED knockout TFK1 cells were detected by qRT‐PCR. (C) Protein levels of the indicated targets were examined by Western blot in EZH2, SUZ12, EED Knockout and control TFK1 cells. (D) The mRNA levels of SFRP1 in GSK126, GSK343 treated and control TFK1 cells were examined by qRT‐PCR. (E) Protein levels of the indicated targets were detected by Western blot in GSK126, GSK343 treated and control TFK1 cells. (F) The expression level of SFRP1 in cholangiocarcinoma (CCA) and adjacent tissue from GSE76297. (G–I) The correlation between the expression of EZH2, SUZ12 and EED with the expression of SFRP1 in CCA tumour tissue and non‐tumour tissue was determined using the Pearson correlation coefficient. This analysis was performed using data from the GSE76297 dataset. (J) A schematic diagram of the SFRP1 promoter region with potential H3K27me3 sites was shown. The sequences were indicated. S1, S2, S3 and S4 indicate four sites (Region 1, Region 2, Region 3 and Region 4) in the SFRP1 promoter. (K) A chromatin immunoprecipitation‐quantitative polymerase chain reaction (ChIP‐qPCR) assay was used to examine the binding of H3K27me3 at the SFRP1 promoter in EZH2, SUZ12 and EED Knockout and control TFK1 cells. (L) A ChIP‐qPCR assay was used to examine the binding of H3K27me3 at the SFRP1 promoter in GSK126, GSK343 treated and control TFK1 cells. The data represent three independent experimental results.

To investigate the mechanism of PRC2 in CCA, we knockout EZH2, SUZ12 and EED, the three core subunits of PRC2, respectively, using CRISPR/Cas9 in the TFK1 cell line. Knockout of EZH2, SUZ12 and EED in the TFK1 cell line resulted in suppressed cell growth and colony‐forming abilities, as well as reduced migration and invasive capabilities (Figure S3A–E). The analysis of TFK1‐WT, TFK1‐EZH2KO and TFK1‐SUZ12KO through transcriptome sequencing revealed a common alteration in 1125 genes following EZH2 knockout and SUZ12 knockout (Figure 4A and Tables S1 and S2). These genes are hypothesized to be primarily regulated by PRC2 in CCA, as depicted in the associated volcano plot (Figure S3F–I). Further analysis showed a significant increase in the expression of SFRP1 in both knockout groups, a gene that has been shown in our previous studies to play a tumour suppressor role in CCA.^21^ We then tested SFRP1 expression at the transcriptional and protein levels in TFK1 cells knockout by EZH2, SUZ12 and EED, and found that the knockout of each of the three subunits of PRC2 impaired the catalytic function of PRC2 and caused upregulation of SFRP1 expression (Figure 4B,C). To provide further support for this finding, we evaluated the transcript and protein levels of SFRP1 in TFK1 cells following treatment with GSK126 and GSK343. These inhibitors target the catalytic activity of EZH2 and confirm that when EZH2 function is impaired, it leads to the activation of SFRP1 transcription (Figure 4D,E). Thus, we analyzed the expression of SFRP1 in the GEO database and found that the expression of SFRP1 was significantly lower in CCA tissues than in adjacent tissues (Figure 4F). Further analysis of the GEO database uncovered that the expression of EZH2, SUZ12 and EED in CCA and adjacent tissues were negatively correlated with the expression of SFRP1, respectively (Figure 4G–I). SFRP1 is a known upstream inhibitor of the Wnt/β‐catenin pathway. In order to investigate this further, we conducted Western Blot assays to measure β‐catenin protein expression and real‐time quantitative polymerase chain reaction (RT‐qPCR) to measure mRNA expression after knockout of EZH2, SUZ12 and EED. Our findings revealed that while the mRNA expression of β‐catenin remained unchanged, there was a significant decrease in the protein level of β‐catenin following the knockout of EZH2, SUZ12 and EED (Figure S3J,K). These results provide additional evidence supporting the role of PRC2 in regulating the Wnt/β‐catenin pathway through the regulation of SFRP1.

To examine how PRC2 controls the expression of SFRP1, we conducted chromatin immunoprecipitation‐qPCR (ChIP‐qPCR) experiments on the SFRP1 promoter. Specifically, we utilized an antibody targeting the PRC2 catalytic product H3K27me3, and our results showed that cells with EZH2, SUZ12 and EED knockout, as well as those treated with GSK126 and GSK343, exhibited decreased levels of H3K27me3 in the SFRP1 promoter (Figure 4J–L). Reverse‐ChIP experiments were conducted using biotin‐labeled probes to capture the SFRP1 promoter. Subsequent analysis involved qPCR experiments and Western blot assays. The results showed the presence of H3K27me3 on the SFRP1 promoter (Figure S3L,M). These findings indicate that the presence of H3K27me3 negatively impacts the transcriptional activity of the SFRP1 promoter.

### 2.5 PRC2 causes SFRP1 promoter DNA hypermethylation in CCA

**FIGURE 5:**
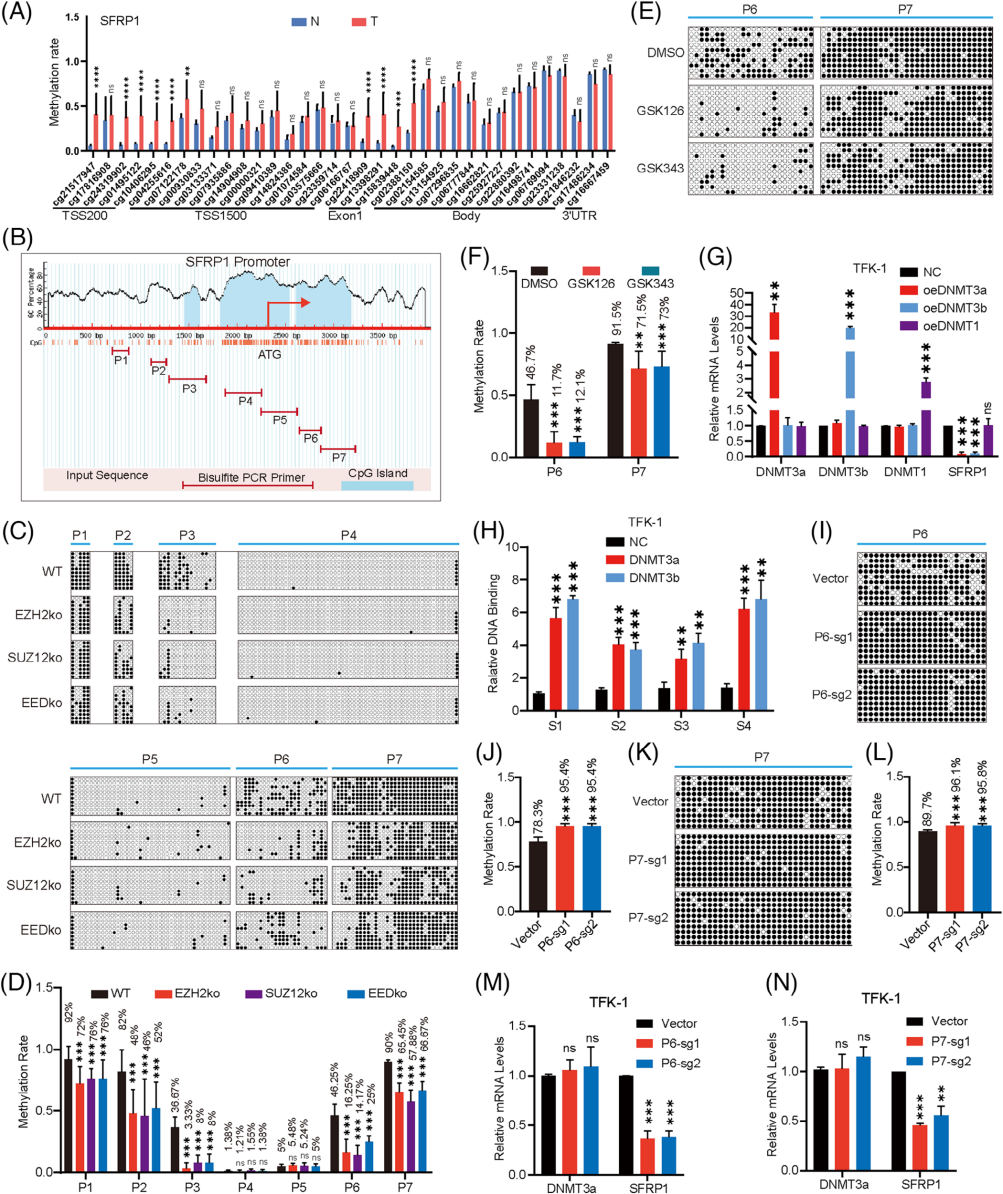
Polycomb repressor complex 2 (PRC2) causes secreted frizzled‐related protein 1 (SFRP1) promoter DNA hypermethylation in cholangiocarcinoma (CCA). (A) Differences in DNA methylation of SFRP1 in CCA and adjacent tissue from the GSE201241 dataset. (B) Distribution of SFRP1 promoter CpG island and location of bisulphite sequencing PCR (BSP) fragment. Images were generated by MethPrimer (http://www.urogene.org/methprimer) and followed up with labelled BSP fragment positions for SFRP1. (C, D) EZH2, SUZ12 and EED knockout and control TFK1 cells were examined in the BSP assay (Each row represents a sequence, and the dots represent CpG sites. The white and black dots represent unmethylated and methylated CpG, respectively.) (C). Quantitative analysis of DNA methylation rate in BSP assay (D). (E, F) DMSO, GSK126 and GSK343 treated TFK1 cells for 48 h were examined in the BSP assay (E). Quantitative analysis of DNA methylation rate in BSP assay (F). (G) The levels of DNMT3a, DNMT3b, DNMT1 and SFRP1 mRNA in DNMT3a, DNMT3b and DNMT1 transfected TFK1 cells were examined by qRT‐PCR. (H) A chromatin immunoprecipitation‐quantitative polymerase chain reaction (ChIP‐qPCR) assay was used to examine the binding of DNMT3a/ DNMT3b at the SFRP1 promoter in DNMT3a‐Flag and DNMT3b‐Flag overexpressing TFK1 cells. (I–L) Vector and dCas9‐DNMT3a‐gRNA1, dCas9‐DNMT3a‐gRNA2 Transfected TFK1 cells for 48 h were examined in the BSP assay (I, K). Quantitative analysis of DNA methylation rate in BSP assay (J, L). (M, N) The levels of SFRP1 mRNA in TFK1 cells in Vector and dCas9‐DNMT3a‐gRNA1, dCas9‐DNMT3a‐gRNA2 groups were examined by qRT‐PCR. The data represent three independent experimental results.

SFRP1 expression levels were highly regulated by DNA methylation in our previous study. GEO database analysis discovered that the DNA methylation of the SFRP1 promoter was significantly higher in CCA tissues than in adjacent tissues (Figure 5A). Then we designed seven pairs of bisulphite sequencing PCR (BSP) primers (named P1–P7, respectively) at the SFRP1 promoter, to determine whether the function of PRC2 affects DNA methylation at the SFRP1 promoter (Figure 5B). The results showed that DNA methylation of the SFRP1 promoter was significantly reduced in five fragments(P1, P2, P3, P6 and P7) in TFK1 cells with knockout of EZH2, SUZ12 and EED, respectively, and did not differ in the two middle fragments(P4 and P5)(Figure 5C,D). Furthermore, we examined the DNA methylation rates of P6 and P7 in TFK1 cells treated with PRC2 inhibitors GSK126 and GSK343. The results were consistent with the above findings, showing that impaired PRC2 function led to a reduction in DNA methylation rates. (Figure 5E,F).

DNA methyltransferases, including DNMT3a, DNMT3b and DNMT1, are enzymes responsible for catalyzing DNA methylation. Our analysis of the GEO database revealed that DNMT3a, DNMT3b and DNMT1 were generally expressed at higher levels in CCA compared to adjacent tissues. (Figure S4A–L). To determine the DNA methyltransferases responsible for SFRP1 DNA hypermethylation, we overexpressed DNMT3a, DNMT3b and DNMT1 in TFK1 cells. Interestingly, the overexpression of DNMT3a and DNMT3b resulted in decreased transcript levels of SFPR1, while overexpression of DNMT1 did not affect the transcript levels of SFRP1 (Figure 5G). We verified that DNMT3a and DNMT3b were anchored to the SFRP1 promoter using ChIP‐qPCR assay (Figure 5H). Aiming to confirm whether the transcriptional level of SFRP1 is affected by promoter DNA methylation, we developed two sgRNAs targeting P1, P2, P3, P6 and P7. The transcriptional level of SFRP1 was reduced by dCas9‐DNMT3a by targeting the DNA methylation of promoter P1, P2 and P3 (Figure S4M–R) or P6 and P7 (Figure 5I–N) using a sequence‐specific sgRNA. To dismiss the impact of off‐target effects, we employed a rigorous approach. First, we identified the top four off‐target genes predicted by each sgRNA for qPCR validation. Remarkably, no significant alterations were observed in the expression levels of these genes, indicating that the possibility of off‐target effects was effectively ruled out (Figure S5A–K). In order to further investigate the relationship between DNA methylation and PRC2‐mediated regulation of SFRP1 expression by H3K27me3, we treated TFK1 cells with Decitabine, a cytosine analogue that acts as a broad‐spectrum DNA methyltransferase inhibitor. After the treatment, we analyzed the DNA methylation status of the SFRP1 gene promoter and evaluated the expression of SFRP1. The results showed a reduction in SFRP1 promoter DNA methylation in wild‐type, EZH2ko, SUZ12ko and EEDko cells following Decitabine treatment, with the most significant reduction observed in wild‐type cells. Furthermore, SFRP1 expression increased in all four cell types after treatment, with the highest increase observed in wild‐type cells (Figure S6A–E). This intriguing result suggests that the expression of SFRP1 is influenced by both H3K27me3 and DNA methylation. Furthermore, the presence of PRC2‐mediated H3K27me3 affects DNA methylation at the SFRP1 promoter.

### 2.6 SFRP1 is required for the progression of CCA in vitro and in vivo

**FIGURE 6:**
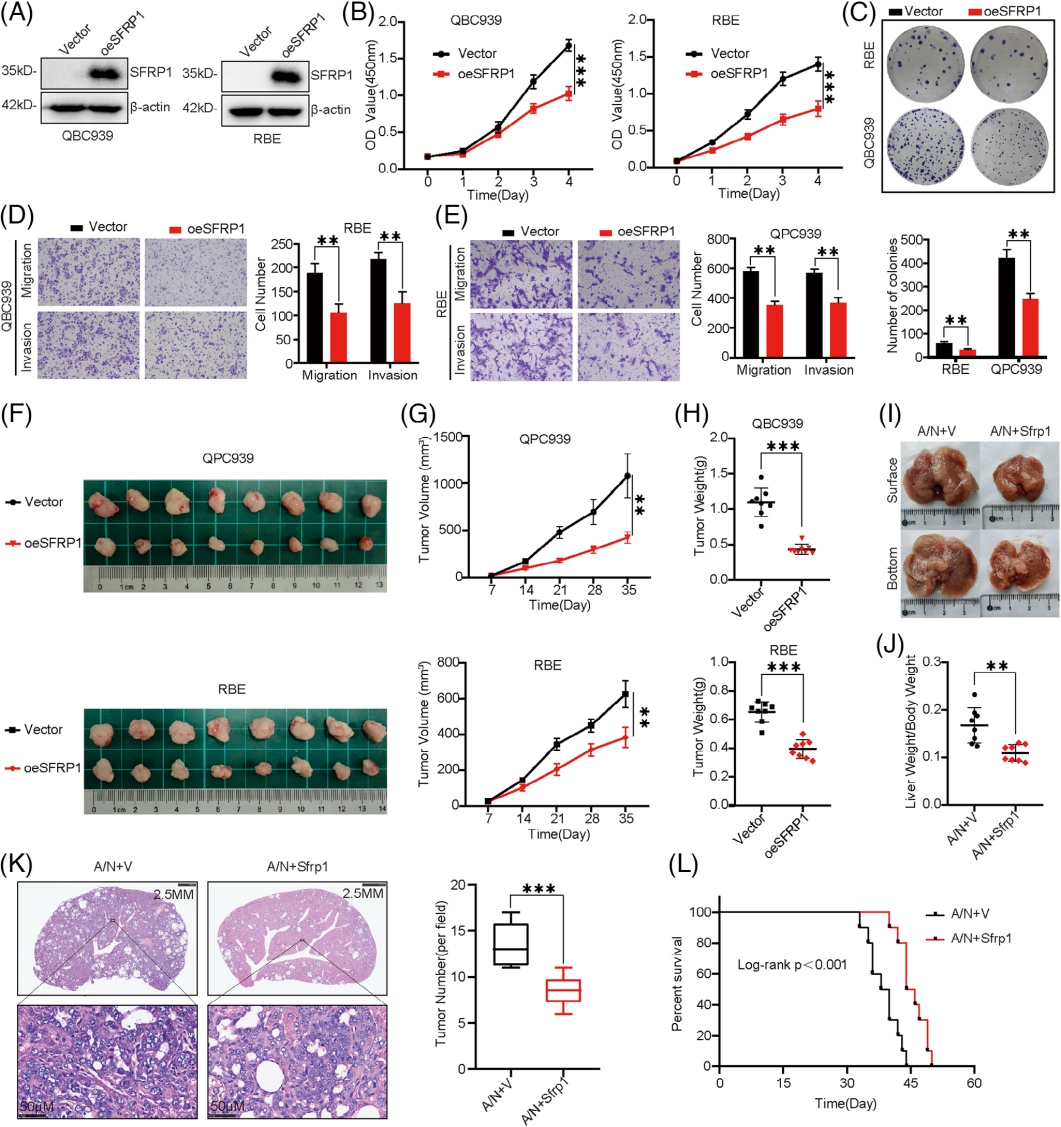
Secreted frizzled‐related protein 1 (SFRP1) is required for the tumourigenesis of cholangiocarcinoma (CCA) in vitro and in vivo. (A) Overexpression of SFRP1 in QBC939 and RBE cells was confirmed by western blot. (B) Proliferative ability of SFRP1‐overexpression cells and control cells examined by Cell Counting Kit 8 (CCK‐8) assay. (C) Colony‐formation assay of SFRP1 overexpression and control cells (Top: representative images. Bottom: quantification data). (D, E) Cell migration assay and Matrigel invasion assay of SFRP1‐overexpression and control cells (Left: representative images. Right: quantification data). (F) Overview of tumours in transplanted xenografts with SFRP1‐overexpression and control cells (*n* = 8) (G, H) Tumor volume (G) and weight (H) were measured. (I) Representative overview of tumour burden in A/N+V and A/N+Sfrp1 mouse livers. (J) Ratios of liver weight to body weight in A/N+V and A/N+Sfrp1 mice. (K) H&E‐staining images of livers in A/N+V and A/N+Sfrp1 mice (Left: Representative H&E‐staining images. Right: Quantitative analysis of tumour number). (L) Survival curve of A/N+C and A/N+Sfrp1 mice.

To validate the role of SFPR1 in CCA, we performed overexpression of SFRP1 in QBC939 and RBE cells (Figure 6A). Overexpression of SFPR1 inhibits cell growth and colony‐forming abilities in two CCA cell lines (Figure 6B,C). In addition, overexpression of SFPR1 inhibited the migration and invasive abilities in CCA cells (Figure 6D,E). We then further validated the role of SFPR1 in CCA using a xenograft tumour model in vivo. Cell lines with stably overexpressed SFRP1 were established and subcutaneously injected into nude mice in two independent experimental groups with their own controls. The study's findings reveal that SFRP1 Inhibited xenograft tumour growth in two CCA cell lines (Figure 6F), reflected by smaller tumour mass and lower tumour weight (Figure 6G,H). Consistent with the above, we established a CCA primary tumour mouse model by hydrodynamic transfection of activated forms of Akt and NICD plasmids into C57BL/6 mice to further investigate the role of Sfrp1 in regulating CCA progression in vivo. The Sfrp1 overexpression plasmid was co‐injected with the Akt/NICD plasmid to observe whether Sfrp1 had a role in inhibiting CCA progression in vivo. Notably, Akt/NICD1+ Sfrp1 (A/N+Sfrp1) mice developed a smaller tumour load 5 weeks after plasmid injection compared to Akt/NICD1/ Vector (A/N+V) mice (Figure 6I). The ratio of mouse liver weight to body weight showed a significantly decreased tumour load in A/N+ Sfrp1 compared to A/N+V mice (Figure 6J). H&E staining showed a significant increase in the number of tumour lesions in A/N+Sfrp1 mice compared to A/N+V mice (Figure 6K). The survival results were consistently better in Sfrp1 overexpressing mice compared to controls (Figure 6L). Histological analysis showed positive staining for cytokeratin 19 in both A/N+Sfrp1 and A/N+V transfected induced tumours, and histochemical staining further demonstrated the overexpression effects of Sfrp1 (Figure S7A). Overall, our data demonstrate the potential function of Sfrp1 in inhibiting CCA progression in vivo.

### 2.7 Therapeutic effects of EZH2 inhibitor in CCA

**FIGURE 7:**
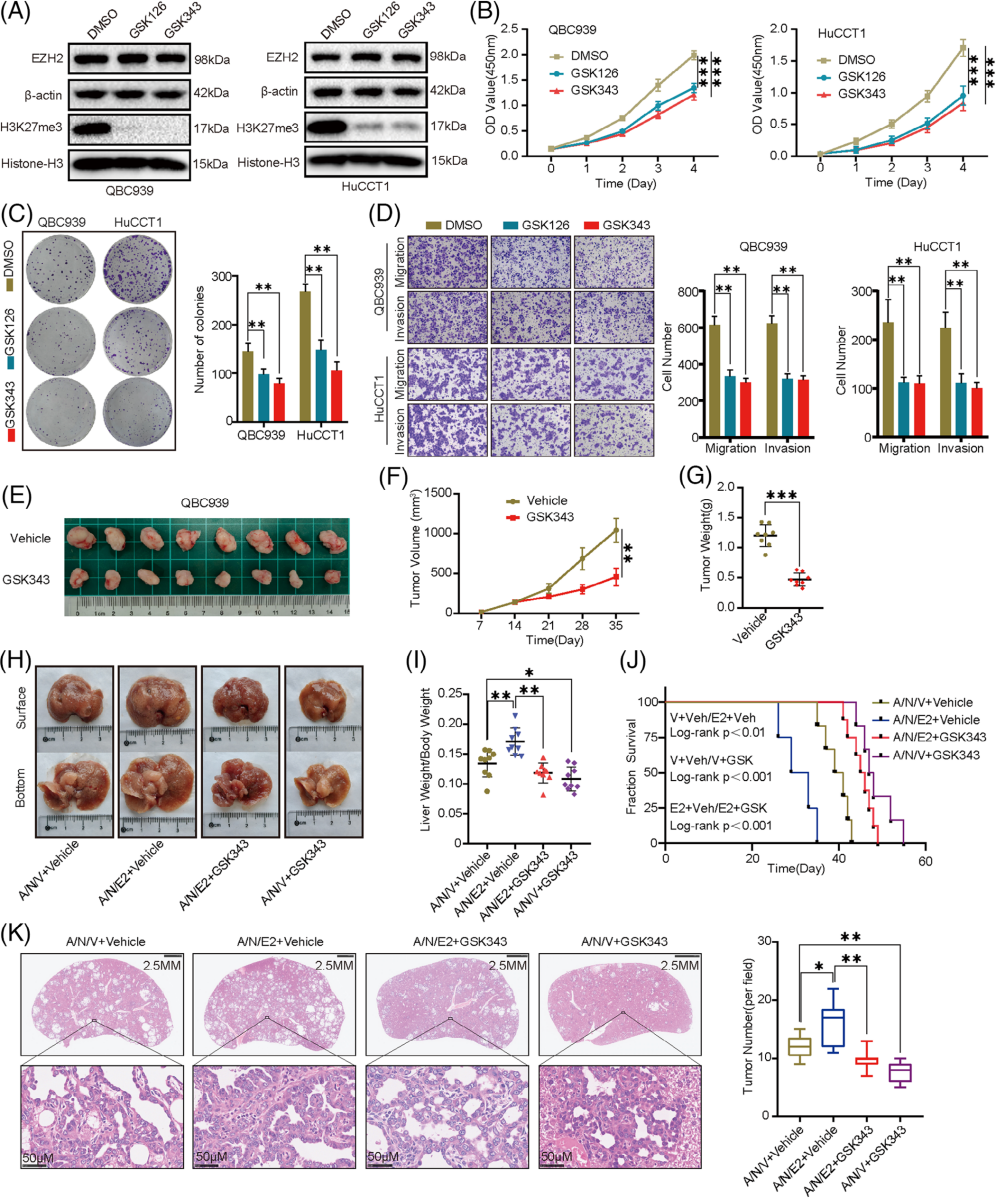
Therapeutic effects of EZH2 inhibitor in cholangiocarcinoma (CCA). (A) Treatment of GSK126 and GSK343 for 48 h in QBC939 and HuCCT1 cells examined by western blot. (B) Proliferative ability of GSK126 and GSK343 treated cells and control cells examined by Cell Counting Kit 8 (CCK‐8) assay. (C) Colony‐formation assay of GSK126 and GSK343 treated cells and control cells (Left: representative images. Right: quantification data). (D) Cell migration assay and Matrigel invasion assay of GSK126 and GSK343 treated cells and control cells (Left: representative images. Middle and right panels: quantification data). (E) A representative overview of tumours in transplanted xenografts with the vehicle and GSK343 treated (*n* = 8). (F, G) Tumor volume (F) and weight (G) were measured. (H) Overview of tumour burden in A/N/C+Vehicle, A/N/E2+Vehicle, A/N/C+GSK343 and A/N/E2+GSK343 mouse livers. (I) Ratios of liver weight to body weight in A/N/C+Vehicle, A/N/E2+Vehicle, A/N/C+GSK343 and A/N/E2+GSK343 mice. (J) Survival curve of A/N/C+Vehicle, A/N/E2+Vehicle, A/N/C+GSK343 and A/N/E2+GSK343 mice. (K) H&E‐staining images of livers in A/N/C+Vehicle, A/N/E2+Vehicle, A/N/C+GSK343 and A/N/E2+GSK343 mice (Left: Representative H&E‐staining images. Right: Quantitative analysis of tumour number).

After elucidating the role of EZH2‐catalyzed production of H3K27me3 in CCA progression, we next evaluated the potential therapeutic effects of EZH2‐specific inhibitors. We first tested the IC50 of GSK126 and GSK343 in two CCA cell lines, QBC939 and HuCCT1 (Figure S8A,B). When CCA cells QBC939 and HuCCT1 were treated with GSK126 and GSK343, it was observed that the function of EZH2 was significantly impaired and the levels of H3K27me3 were significantly decreased (Figure 7A). GSK126 and GSK343 inhibit cell growth and colony‐forming abilities of two CCA cell lines (Figure 7B,C). In addition, GSK126 and GSK343 inhibited the migration and invasive abilities in CCA cells (Figure 7D). Next, our objective was to explore the effects of GSK343 treatment on both subcutaneous xenografts and primary CCA models in vivo. Notably, GSK343 administration inhibited tumour growth in the subcutaneous xenografts model (Figure 7E), reflected by smaller tumour mass and lighter tumour weight (Figure 7F,G). Akt/NICD1/Vector+GSK343 (A/N/V+GSK343) and Akt/NICD1/Ezh2+GSK343(A/N/E2+GSK343) mice developed a smaller tumour load 5 weeks after plasmid injection, while Akt/NICD1/Vector+Vehicle (A/N/V+Vehicle) and Akt/NICD1/Ezh2+Vehicle (A/N/E2+Vehicle) mice developed a greater number of large lesions in the liver (Figure 7H). The ratio of mouse liver weight to body weight showed a significantly decreased tumour load in A/N/V+GSK343 and A/N/E2+GSK343 and compared to A/N/V+Vehicle and A/N/E2+Vehicle mice (Figure 7I). The survival results were consistently better in GSK343 treatment mice compared to controls (Figure 7J). Notably, GSK343 administration significantly reduced progression and improved OS in both wild‐type and Ezh2 overexpressing primary CCA mouse models (Figure 7J). H&E staining showed a significant decrease in the number of tumour lesions in A/N/V+GSK343 and A/N/E2+GSK343 mice compared to A/N/V+Vehicle and A/N/E2+Vehicle mice (Figure 7K). Histological analysis further demonstrated that the therapeutic effect of GSK343 was reflected in H3K27me3 staining intensity. The A/N/E2+Vehicle group had a higher positive rate than the A/N/V+Vehicle group, while the GSK343‐treated groups A/N/V+GSK343 and A/N/E2+GSK343 had negative staining of H3K27me3 (Figure S8C). Further, we observed the H&E staining of the kidney from each group of mice and determined the safety of GSK343 treatment (Figure S8D).

This study provides further insights into the potential mechanism by which PRC2 promotes CCA progression. It is observed that targeting PRC2 leads to the decrease of H3K27me3 and DNA hypomethylation, resulting in the activation of the SFRP1 promoter and subsequent inhibition of CCA proliferation.

## 3 DISCUSSION

CCA is a highly aggressive malignant tumour, with a survival rate of less than 10% after 5 years.^24^ Individuals with advanced CCA typically have a median survival of less than 2 years, whereas surgical resection, which can potentially cure the disease, is only feasible for those diagnosed at an early stage.^25^, ^26^ The carcinogenesis of CCA is associated with genomic and epigenetic aberrations as well as environmental influences.^27^ Although several potential targets for therapy have been identified, such as those mentioned in references,^28^, ^29^, ^30^ there is still a need for the identification of additional therapeutic targets.

Considerable studies have revealed an important role for PRC2 in cancer progression and suggest that it may be a useful therapeutic target.^31^, ^32^, ^33^ From the molecular function of PRC2, it establishes a repressive state of some genes during the origin of tumour cells and tumour development.^34^ EZH2 is an enzyme subunit of the PRC2 complex that plays an essential role in tumour development through its catalytic and non‐catalytic activities.^35^ Targeting EZH2 for cancer therapy is a current research topic involving tumour immune, tumour metabolism and tumour drug resistance.^36^ It specifically catalyzes the trimethylation of histone H3 lysine 27 (H3K27me3) to promote transcriptional silencing.^37^ Small molecule compound blocking methyltransferase EZH2 activity is in clinical development for the treatment of non‐Hodgkin's lymphoma with acquired mutations in EZH2 function.^38^ In addition to the classical PRC2‐mediated and H3k27me3‐dependent functions of EZH2 in malignant tumours, a part of the study found that EZH2 also has non‐classical functions in promoting tumour progression.^39^, ^40^ In this study, we confirmed the oncogenic role of EZH2, the core catalytic subunit of PRC2, from clinical data, CCA cells, xenograft mice and primary CCA models. We showed that knockout of the three core subunits of PRC2 (EZH2, SUZ12 and EED), respectively, and EZH2 inhibitor GSK126, GSK343, impaired PRC2‐specific H3K27me3 catalytic function and further led to DNA hypomethylation of SFRP1 promoter and transcriptional activation of SFRP1.

We previously confirmed that SFRP1 as a highly regulated gene for transcription by DNA methylation in CCA.^21^ Numerous ChIP‐Seqs illustrated that detectable PRC2 binding was observed in a relatively small fraction of the genome, overlapping mainly with H3K27me3‐positive CpG islands.^41^ It suggests that PRC2 and DNA methylation together protect tumour cells in cancer.^42^ Indeed, EZH2 is required for DNA methylation in the promoters of certain genes as a platform to recruit DNA methyltransferases.^43^ In some tumours, EZH2 can recruit DNA methyltransferases to the promoter regions of related genes, promoting DNA methylation.^44^, ^45^ It has also been confirmed that the deposition of histone modifications is not sufficient to repress target gene transcription.^46^ The reason is that the expression of target genes is regulated by the bivalent epigenetic regulation of H3K27me3 and DNA methylation.^47^, ^48^

From our data, we observed that targeting PRC2 leads to the loss of SFRP1 promoter H3K27me3 and DNA hypomethylation, resulting in transcriptional activation of SFRP1. In our experiments with wild‐type, EZH2ko, SUZ12ko and EEDko CCA cells, treatment with the broad‐spectrum DNA methyltransferase inhibitor Decitabine reduced DNA methylation and activated SFRP1 transcription. However, the increase in SFRP1 expression and the decrease in SFRP1 DNA methylation were more pronounced in the wild‐type group compared to the EZH2ko, SUZ12ko and EEDko groups. The knockout groups already exhibited decreased DNA methylation of the SFRP1 promoter, suggesting that PRC2 suppresses SFRP1 in CCA through a mechanism other than H3K27me3‐mediated repression. It is possible that PRC2 affects the enzymatic activity of DNA methyltransferase to promote promoter DNA methylation, and this mechanism may involve various subunits of PRC2, such as EZH2, SUZ12 and EED, or H3K27me3. Published studies have demonstrated that EZH2 can influence DNA methyltransferases, thereby modifying DNA methylation in specific regions.^44^, ^49^ However, our findings indicate that the knockdown of all three subunits of PRC2, namely EZH2, SUZ12 and EED, leads to a reduction in DNA methylation at the SFRP1 promoter. Therefore, the observed effect cannot be solely attributed to EZH2, SUZ12 and EED, suggesting a potential involvement of H3K27me3. Likewise, the presence of the histone marker H4K20me3 affects the enzymatic activity of DNMT1 at the LINE‐1 gene, leading to alterations in the DNA methylation status.^50^ In our preliminary experiment, we observed that DNMT3a and DNMT3b can bind H3K27me3 through immunoprecipitation and immunofluorescence techniques. However, it is important to note that H3K27me3 is solely a histone mark, and this binding does not prove a direct interaction between H3K27me3 and DNMT3a or DNMT3b. In future studies, we plan to perform more advanced experimental methods to investigate whether DNMT3a and DNMT3b can directly read and bind to H3K27me3. We also aim to examine the enzymatic activity of DNMT3a and DNMT3b when they read and bind to H3K27me3, as well as its impact on local DNA methylation status.

From our results, the knockout of one of the three subunits of PRC2 in CCA resulted in reduced protein levels of the other two subunits, both of which also impaired the H3K27me3 catalytic function of PRC2. In contrast, post‐transcriptional modification of SUZ12 promotes catalysis and facilitates suppression of target genes by enhancing PRC2 function.^51^ Targeting EZH2, a variety of inhibitors have now emerged, including those targeting EZH2‐specific H3K27me3 catalytic activity^52^, ^53^ and those that decrease the protein levels of EZH2 or EED.^33^, ^54^ DNA methyltransferase inhibitors including decitabine have significant inhibitory effects on DNMT3a, DNMT3b and DNMT1 and are approved for the treatment of hematologic tumours and expanding into solid tumours.^55^ However, it non‐specifically reduces genome‐wide methylation and causes genomic instability, which inversely increases the risk of tumour proliferation and invasion.^56^ As a result, 40% of DAC‐related clinical trials registered on Clinical trials were stopped. Thus, a novel anti‐cancer idea is to target tumour progression caused by PRC2‐mediated DNA hypermethylation through EZH2 inhibitors.

This study provides insights into the role and potential mechanisms of PRC2 in CCA. It focuses on targeting PRC2‐mediated H3K27me3 and DNA hypermethylation, which leads to the bivalent promoter epigenetic silencing of SFPR1 and inhibits the progression of CCA. The findings suggest that targeting PRC2, such as using an EZH2 inhibitor, could be a promising approach for developing anti‐cancer drugs for CCA. However, further research is needed to explore the characteristics and biological safety of different EZH2 inhibitors for effective clinical treatment against CCA.

## 4 MATERIALS AND METHODS

### 4.1 Patient samples

Human CCA tissue samples were collected from the Department of Biliary and Pancreatic Surgery, Tongji Hospital, Huazhong University of Science and Technology (Wuhan, China) and were surgically resected and diagnosed as CCA by pathological examination. Patients were followed up after surgery, and the date of death or the last follow‐up was recorded. The Ethics Committee of Tongji Hospital of Huazhong University of Science and Technology approved the study. The patients were informed and signed informed consent forms.

### 4.2 Cell culture

Human CCA cells (TFK1, QBC939, EGI1, HuCCT1, HuH28, RBE, SSP25 and HCCC‐9810) and human embryonic kidney (HEK293T) cells were cultured in RPMI 1640 or DMEM medium (HyClone) containing 10% fetal bovine serum (NEWZERUM), 100 units/mL penicillin and 100 μg/mL streptomycin in an incubator at 37°C with 5% CO_2_. Some cells were treated with 5 μM GSK343 (#S7164; Selleck Chemicals), 20 μM GSK126 (#S7061; Selleck Chemicals), or 10 μM decitabine (#S1200; Selleck Chemicals) for 72 h. The treatment was performed by changing the complete medium daily.

### 4.3 Western blot

Western blot analysis Cell samples were lysed using RIPA buffer containing proteinase inhibitor cocktail and PhosSTOP phosphatase inhibitor (Roche). Total protein was extracted, mixed with 5× loading buffer and denatured at 95°C for 15 min. Proteins in each lysate sample were separated by sodium dodecyl sulphate‐polyacrylamide gel electrophoresis and transferred onto nitrocellulose membranes (Millipore, USA). The membranes were then blocked with 5% BSA at room temperature and incubated overnight at 4°C with primary antibodies. Next, the membranes were incubated with secondary antibodies, and the signals were visualized using enhanced chemiluminescence (Thermo Fisher Scientific). The results were analyzed using Image Lab software (Bio‐Rad). The primary antibodies used in this study are listed in Table S3.

### 4.4 Plasmid construction

The full‐length sequences of EZH2, DNMT3a, DNMT3b and DNMT1 were obtained by PCR amplification from human cell cDNA and cloned into pHAGE‐Flag vector, respectively. Gene‐specific sequence shRNA primers were provided by Sangon Biotech and inserted into the Plko.1 vector. The primer sequence information is shown in Table S4.

### 4.5 Viral packaging and viral infection

Transient transfections were performed using linear polyethene terephthalate MW40000 (40816ES02; Yeasen), according to the manufacturer's protocol. The target gene plasmid was co‐transfected with pMD2G and psPAX packaging plasmids in HEK293T cells. After 72 h, the supernatant was collected with a 0.45 μm filter (BS‐PES‐45; Biosharp). QBC939, RBE, HuCCT1 and TFK1 cells were transfected with 1 mL of viral supernatant and 1 mL of RPMI 1640 complete medium, polybrene 2 μL (40804ES76; Yeasen) for 24 h and screened with puromycin hydrochloride (CL13900; Selleck) (1 μg/ml) for 10 days. Western blots were then performed to examine the efficiency of target gene knockdown or expression.

### 4.6 Cell proliferation assay

Cell viability was measured by a Cell Counting Kit 8 (CCK‐8) (40203ES92; Yeasen). 1000 stably transduced cells were plated into 96‐well plates, wells were spiked with CCK‐8 reagent according to the manufacturer's protocol, cells were incubated at 37°C, 5% CO_2_ for 90 min, and absorbance was measured at 450 nm. For colony formation experiments, 1000 cells were planted in 6‐well plates, added to 2 mL of complete medium and grown for 2 weeks. Visible colonies were stained with 1% crystal violet and the number of colonies was counted.

### 4.7 Transwell assay

For migration assay, 200 μL of serum‐free medium containing 3 × 10^4^ cells were added to the transwell (polycarbonate membrane pore diameter 8 μm; Corning Incorporated), and the transwell was placed in a Receiving well containing 800 μL of complete medium. For the invasion assay, transwells were pre‐wrapped with 100 mL Matrigel at 37°C for 1 h. Cells were then added as described in the migration assay. 48 or 72 h later, these chambers were collected and stained with 1% crystal violet.

### 4.8 Construction of EZH2, SUZ12 and EED respective knockout cell lines

EZH2, SUZ12 and EED respective knockout cell lines were constructed using CRISPR/Cas9 system technology. Briefly, the EZH2, SUZ12, EED targeting sequences EZH2‐sgRNA (5′‐AGAAGGGACCAGTTTGTTGG‐3′), SUZ12‐sgRNA (5′‐GCTTCGGGCGGCAAATCCGG‐3′) and EED‐sgRNA (5′‐GAGGGAAGTGTCGACTGCGC‐3′), and inserted into PX459 vector (48139; Addgene). After 24 h of TFK1 cell transfection with the plasmid, the cell was treated with 1 μg/mL puromycin dihydrochloride for 3 days. Monoclonal cell lines were isolated by restriction dilution using 96‐well plates. The correct clone was finally confirmed by western blots and specific antibody analysis.

### 4.9 Total RNA isolation and RT‐qPCR

TRIzol (R711; Vazyme) was used to extract total RNA and cDNA was obtained using HiScript III RT SuperMix for qPCR (R222; Vazyme) according to the manufacturer's protocol. RT‐qPCR was performed using ChamQ universal SYBR qPCR master mix (Q711; Vazyme) with β‐actin as the internal reference. The results were analyzed using Bio‐Rad CFX Manager 2.1. The primers are listed in Tables S5 and S6.

### 4.10 Bisulphite sequencing PCR

The BSP primers were designed online by MethPrimer (plus CpG Island Prediction) (http://www.urogene.org/methprimer).^57^ The primers are listed in Table S7. DNA extraction using a DNA extraction kit (D3396020000J12T006; Omega), BSP transformation reaction using a kit (EM101‐02; Vazyme). PCR amplification was then performed using Taqase (C601‐02; Vazyme). the PCR products were purified and inserted into the vector (EM101‐02; Vazyme), and finally, 10 bacterial clones were randomly selected for sequence measurement.

### 4.11 ChIP‐quantitative PCR

After CCA cells were prepared, the cells were added with 1% formaldehyde for protein cross‐linking with DNA, and then neutralized with 10% glycine. Cells were then collected for ultrasonication and examined, and DNA‐protein complexes were precipitated with A/G magnetic beads and first‐antibody overnight at 4°C. Sample washing and uncross‐linking. DNA was purified, concentration was measured and qPCR was performed. The primer sequence information is listed in Table S8.

### 4.12 Reverse‐ChIP

First, probes were designed to complement the SFRP1 promoter positive or antisense strand sequences. The probes were synthesized by Sangon Biotech and labelled with biotin at the 3′ ends. A total of six primers were divided into odd and even groups. The sequences of the probes can be found in Table S9. Note that, 1×10^9^ TFK1 cells were cross‐linked with 3% formaldehyde for 10 min at room temperature and then neutralized with 1.375 M Glycine for 5 min at room temperature. Nuclei were prepared using Sucrose Buffer, RNA was removed using RNase Buffer, and chromatin was fragmented by sonication. The samples were categorized as Reverse‐ChIP NC group, Reverse‐ChIP odd group, Reverse‐ChIP even group, Input Protein group and Input DNA group, with a ratio of 20:20:20:1:1. Agarose beads were pre‐washed for the Reverse‐ChIP NC group, Reverse‐ChIP odd group and Reverse‐ChIP even group. After denaturation at 85°C for 3 min, the probes were quickly transferred to an ice bath for pre‐denaturation. The probes were added at a ratio of 1 pmol probe per strip per 1×10^9^ cells and divided into three groups: NC group, odd group and even group. Hybridization was performed by warming the probes at 85°C for 10 min followed by hybridization at 37°C for 30 min; warming at 70°C for 5 min followed by hybridization at 37°C for 30 min; and warming at 55°C for 2.5 min followed by hybridization at 37°C for 60 min. Streptavidin beads were added to each tube of the samples separately and thoroughly mixed, then incubated for 90 min at room temperature on a vertical mixer to facilitate binding. The samples from the Reverse‐ChIP NC group, Reverse‐ChIP odd group and Reverse‐ChIP even group were washed 4 times. Subsequently, the samples were divided into two groups, the Protein group and DNA group, at a ratio of 19:1 for each group. The Protein group was eluted with Protein Elution Buffer and then added to the loading buffer for western blotting. The DNA group underwent qPCR after DNA extraction.

### 4.13 Experiments with animals

BALB/c (nu/nu) male nude mice and male C57BL/6J mice were purchased from Gempharmatech Co., Ltd, and housed in a specific pathogen‐free facility at the Animal Center of Tongji Hospital, Tongji Medical College, Huazhong University of Science and Technology.

For xenografts, 5‐week‐old BALB/c (nu/nu) nude male mice were randomly grouped (*n* = 8 per group). 3×10^6^ stably transfected cells were resuspended in 100 μL PBS and injected subcutaneously into the right axillae of the mice. The tumour was measured once a week and the tumour volume was calculated by the formula: Total tumour volume (mm^3^) = 0.52 × length × width^2^. After 4 or 5 weeks, the mice were euthanized and died and the tumour was resected for follow‐up experiments.

The establishment of the primary CCA mouse model. 20 μg of pT3EF1aH‐myr‐Akt (179909; Addgene) and 20 μg of pT3EF1aH‐NICD1 (86500; Addgene) together with 6 μg of the transposase plasmid pCMV (CAT)T7‐SB100 (34879; Addgene) were diluted in 2.0 mL saline. 2 mL of plasmid mixture was injected into the lateral tail vein of 5‐week‐old C57BL/6J mice within 7s. To knock down Ezh2, the plasmid was converted to pT3‐U6 based on pT3‐EF1aH (180149; Addgene) plasmid. Design and synthesis of an Ezh2 gene‐specific sequence of shRNA primers for insert into the pT3‐U6 vector. Add 20 μg of pT3‐U6‐Ezh2#1, pT3‐U6‐Ezh2#2 plasmid or the corresponding control plasmid pT3‐U6 to the above plasmid mixture. For the overexpression of Ezh2 and Sfrp1, the pT3EF1aH‐Ezh2/pT3EF1aH‐Sfrp1 plasmid was constructed using pT3‐EF1aH, and 20 μg of pT3EF1aH‐Ezh2/pT3EF1aH‐Sfrp1 plasmid or the corresponding control plasmid pT3‐EF1aH was added to the above plasmid mixture. Livers are collected at 4 or 5 weeks after hydrodynamic transfection or for analysis of CCA tumourigenesis and progression.

Tumor treatment. When the xenograft tumour volume reached 110–160 mm^3^ or when the primary CCA mice were transfected for 2 weeks, the mice were treated for 2 weeks as follows: The control group was treated with 100 μL vehicle (5% DMSO+40% PEG+5% Tween80+50% water) intraperitoneally daily, and treatment group was treated with 20 mg/kg GSK343 (dissolved in 5% DMSO+ 40%PEG+5%Tween80+50% water) intraperitoneally daily. The mice were treated for 14−21 days, then the mice were euthanized and died and the tumour was resected for follow‐up experiments.

### 4.14 Statistical analysis

All experiments were performed independently at least three times and data were presented as the mean ± S.D. unless indicated otherwise. GraphPad Prism software 8.0 (GraphPad Prism Software Inc.) was used for statistical analysis. Data analysis was performed with a two‐sample t‐test for two independent samples, while analysis of variance was used to compare between groups. the Pearson coefficient for the linear correlation between two different parameters. Survival curves were analyzed using the Kaplan–Meier method and the log‐rank test. *p*‐Values < .05 were considered statistically significant, the p values are represented as follows: * *p* < .05;** *p* < .01;*** *p* < .001, and not statistically significant when *p* > .05.

## AUTHOR CONTRIBUTIONS

Guanhua Wu participated in the research design and drafted the paper; Guanhua Wu, Qi Wang and Da Wang conducted experiments; Fei Xiong, Wenzheng Liu, Junsheng Chen, Bing Wang, Wenhua Huang and Xin Wang analysed the data; Yongjun Chen supervised the study; all authors approved the final manuscript.

## FUNDING INFORMATION

This work was supported by grants from the National Natural Science Foundation of China (#81974438 and #82173069).

## CONFLICT OF INTEREST STATEMENT

The authors declare no conflict of interest.

## ETHICS STATEMENT

This study was approved by the animal ethics committee of Tongji Hospital, Tongji Medical College, Huazhong University of Science and Technology. Animal care and experiments were conducted in compliance with the Institutional Animal Care and Use Committee and NIH guidelines.

## Supporting information

Supporting Information

FIGURE S1 (A) Immunohistochemical staining assay detected cytokeratin 19 and Ezh2 in the tumours.

FIGURE S2 (A) Immunohistochemical staining assay detected cytokeratin 19 and Ezh2 in the tumours.

FIGURE S3 (A) The proliferative ability of EZH2KO, SUZ12KO and EEDKO cell lines and WT cells was examined using the CCK‐8 assay. (B, C) The colony‐formation assay was performed on EZH2KO, SUZ12KO, EEDKO cell lines and WT cells (B: representative images. C: quantification data). (D, E) Cell migration assay and Matrigel invasion assay were conducted on EZH2KO, SUZ12KO and EEDKO cell lines and WT cells (D: representative images. E: quantification data). (F, G) Volcano plot showing the transcriptome analysis performed on TFK1 cells. F for EZH2KO and WT, G for SUZ12KO and WT. (H, I) Volcano plot showing the 1125 genes following EZH2KO and SUZ12KO. H for EZH2KO and WT, I for SUZ12KO and WT. (J, K) The relative mRNA expression (J) and protein level (K) of β‐catenin were measured using RT‐qPCR and Western blot assays after the knockout of EZH2, SUZ12 and EED in TFK1 cells. L‐M. To detect the distribution of H3K27me3 on the SFRP1 promoter, a Reverse‐ChIP assay was performed in TFK1 cells. The success of the Reverse‐ChIP assay in pulling down the SFRP1 promoter was verified through qPCR experiments (L), while the detection of proteins binding to the SFRP1 promoter was done using Western blot (M).

FIGURE S4 (A–D) The expression level of DNMT1 in CCA and adjacent tissue from GSE32879, GSE76297, GSE107943 and GSE119337. (E–H) The expression level of DNMT3a in CCA and adjacent tissue from GSE32879, GSE76297, GSE107943 and GSE119337. (I–L) The expression level of DNMT3b in CCA and adjacent tissue from GSE32879, GSE76297, GSE107943 and GSE119337. (M–O) The BSP assay was performed on TFK1 cells transfected with vector and dCas9‐DNMT3a‐gRNA1(sg1), dCas9‐DNMT3a‐gRNA2(sg2) for 48 h. M represents P1‐sg1 and P1‐sg2, N represents P2‐sg1 and P2‐sg2 and O represents P3‐sg1 and P3‐sg2. (P–R) The expression levels of SFRP1 mRNA in TFK1 cells were examined using RT‐qPCR in the Vector and dCas9‐DNMT3a‐gRNA1, dCas9‐DNMT3a‐gRNA2 groups. P represents P1‐sg1 and P1‐sg2, Q represents P2‐sg1 and P2‐sg2 and R represents P3‐sg1 and P3‐sg2.

FIGURE S5 (A) P1, P2, P3, P6 and P7 targeted methylated gRNA1 (sg1) and gRNA2 (sg2), selecting the four highest scoring off‐target gene sequences for each gRNA (individual off‐target sequences are less than four). (B–K) RT‐qPCR examination of the expression of off‐target genes targeted by methylated gRNA. B represents P1‐sg1, C represents P1‐sg2, D represents P2‐sg1, E represents P2‐sg2, F represents P3‐sg1, G represents P3‐sg2, H represents P6‐sg1, I represents P6‐sg2 and J represents P7‐sg1 and K represents P7‐sg2.

FIGURE S6 (A–D) The SFRP1 promoter P6 and P7 BSP assay was performed on wild‐type, EZH2ko, SUZ12ko and EEDko TFK1 cells treated with 10 μM Decitabine for 72 h. (E) The expression of SFRP1 was tested by RT‐qPCR in wild‐type, EZH2ko, SUZ12ko and EEDko TFK1 cells treated with 10 μM Decitabine for 72 h.

FIGURE S7 Immunohistochemical staining assay detected cytokeratin 19 and Sfrp1 in the tumours.

FIGURE S8 (A, B) The IC50 values of GSK126 and GSK343 in QBC939/HuCCT1 cells. (C) Immunohistochemical staining assay detected H3K27me3 in the tumours. (D) The H&E‐staining of mouse kidney.

## Data Availability

The data that support the findings of this study are available from the corresponding author upon reasonable request.
