# Supplementary material for: Targeting polycomb repressor complex 2‐mediated bivalent promoter epigenetic silencing of secreted frizzled‐related protein 1 inhibits cholangiocarcinoma progression
Source: Clin Transl Med. 2023 Dec 4;13(12):e1502. doi: 10.1002/ctm2.1502 (PMC10696163; doi:10.1002/ctm2.1502)
Supplement: Supplementary file 1 — Supporting Information [file CTM2-13-e1502-s009.docx]

Table S1. Gene expression log2FoldChange for EZH2ko vs TFK1-WT and SUZ12ko vs TFK1-WT respectively.

| gene_name | EZH2ko vs TFK1-WT log2FoldChange | gene_name | SUZ12ko vs TFK1-WT log2FoldChange |
| --- | --- | --- | --- |
| 1-Mar | 1.98967077 | 1-Mar | 3.057820347 |
| A1CF | 3.317001137 | 4-Mar | -3.939680507 |
| A2M-AS1 | 1.077503505 | 4-Sep | -1.54126319 |
| A4GALT | 1.728532599 | 5-Sep | 2.523617906 |
| AARD | 4.976965206 | 9-Sep | -1.279389802 |
| ABCA1 | 1.688454787 | 11-Sep | 1.387032961 |
| ABCA12 | -1.283464233 | A2M | 2.286437832 |
| ABCA5 | 1.029574076 | A2M-AS1 | 2.019399609 |
| ABCA8 | 3.774690164 | A4GALT | 3.303421073 |
| ABCA9 | 2.068967249 | AACSP1 | -4.968481957 |
| ABCC6 | 1.102710054 | AADAC | -1.632778491 |
| ABCC9 | 2.455063199 | AADACL2-AS1 | -2.387490672 |
| ABCD1 | 1.477762899 | AADACP1 | -1.82285977 |
| ABCG1 | -1.272092539 | AANAT | -2.235500078 |
| ABI3BP | 4.779732062 | AARD | 4.662494296 |
| ABLIM2 | -1.152467444 | AASS | 2.705278127 |
| ABLIM3 | 2.074239511 | AATBC | -2.827337893 |
| AC002310.6 | -1.024251678 | AATF | -1.074899934 |
| AC002401.4 | 1.001507639 | AATK | -1.686880661 |
| AC002524.1 | 1.254298494 | ABALON | -2.87790075 |
| AC004585.1 | -1.105715929 | ABCA1 | 1.893772714 |
| AC004656.1 | 1.295794023 | ABCA12 | -3.171435523 |
| AC004704.1 | 7.963497379 | ABCA4 | 1.885136816 |
| AC004706.3 | -1.742920313 | ABCA5 | 1.299115127 |
| AC004816.1 | -1.021161286 | ABCA8 | 5.793922781 |
| AC005326.1 | 1.630648483 | ABCA9 | 2.559296151 |
| AC005336.1 | 1.634784135 | ABCB1 | 1.065128535 |
| AC006262.1 | -2.670889254 | ABCB6 | 1.077248559 |
| AC006262.2 | -1.581813491 | ABCC12 | -3.999252221 |
| AC007066.2 | 1.08972097 | ABCC2 | -3.181674157 |
| AC007405.3 | 1.568192533 | ABCC4 | -1.459242278 |
| AC007686.3 | 1.217665338 | ABCC9 | 2.77339334 |
| AC007743.1 | -1.808405103 | ABCG1 | 1.02457581 |
| AC008440.3 | -1.150588237 | ABCG2 | -2.51046441 |
| AC008443.4 | -1.799748333 | ABCG8 | -5.236213575 |
| AC008537.3 | 1.173125408 | ABHD1 | 1.605256591 |
| AC008667.1 | 4.024118103 | ABHD11 | -1.083319206 |
| AC009041.2 | -2.174978352 | ABHD14A | 1.719958114 |
| AC010327.3 | 3.701044239 | ABHD14B | 1.060883356 |
| AC010343.3 | 1.632502223 | ABI3BP | 6.086042905 |
| AC010754.1 | 3.299936898 | ABLIM1 | -1.522356078 |
| AC010890.1 | 1.871833468 | ABLIM3 | 2.435093012 |
| AC011379.2 | 1.419973591 | ABR | -2.05889961 |
| AC012313.8 | -1.031441229 | AC002066.1 | -4.275515657 |
| AC017048.3 | 3.370008255 | AC002350.1 | -2.184546005 |
| AC018629.1 | 1.336402753 | AC002401.4 | -2.7298995 |
| AC018638.6 | 2.159181521 | AC002429.2 | 4.926824828 |
| AC019069.1 | -1.11711231 | AC003005.2 | -4.400123508 |
| AC022239.1 | 3.82360154 | AC003070.2 | -4.420796826 |
| AC022613.1 | -1.083731041 | AC004233.3 | -2.421673707 |
| AC022916.1 | -2.017112888 | AC004264.2 | -2.26588193 |
| AC023043.3 | 3.234367212 | AC004477.1 | -1.294923208 |
| AC023906.5 | 1.771351654 | AC004540.2 | 4.574453224 |
| AC025884.2 | -1.289951569 | AC004585.1 | -1.211332666 |
| AC026368.1 | 1.570346501 | AC004656.1 | 1.830124487 |
| AC027559.1 | 1.205904945 | AC004704.1 | 7.990559025 |
| AC034213.1 | -1.253939297 | AC004706.3 | -1.956365747 |
| AC034223.1 | 3.107331712 | AC004801.2 | -1.453240718 |
| AC040970.1 | -1.015062529 | AC004816.1 | -1.173206952 |
| AC060780.3 | -1.348233264 | AC004816.2 | 2.857390834 |
| AC061975.1 | 4.673121965 | AC004947.1 | 5.016461627 |
| AC061975.5 | 3.356811603 | AC004987.2 | 1.971543096 |
| AC062029.1 | 1.237536194 | AC005034.4 | 3.230363858 |
| AC068580.4 | 1.874039793 | AC005077.4 | -2.740970385 |
| AC078850.1 | 1.543292554 | AC005330.1 | -1.29336914 |
| AC079949.1 | -1.287094504 | AC005332.1 | 2.534552101 |
| AC080038.1 | 1.754567765 | AC005332.4 | 1.751553756 |
| AC087482.1 | -1.362992515 | AC005336.1 | 1.562238294 |
| AC087623.1 | -1.141845927 | AC005393.1 | -1.506738224 |
| AC090001.1 | -2.443137441 | AC005540.1 | -4.271254463 |
| AC090197.1 | 1.438459703 | AC005622.1 | 2.125069105 |
| AC090409.1 | -2.895238189 | AC005674.2 | -1.255674367 |
| AC090502.1 | -1.58727643 | AC005722.3 | -4.281008671 |
| AC091133.2 | -1.130748673 | AC005730.2 | -2.655589302 |
| AC091212.1 | -1.291557501 | AC005736.1 | 3.117593606 |
| AC091948.1 | 8.000708119 | AC005865.1 | -3.497005094 |
| AC092327.2 | 3.853781959 | AC005865.2 | -2.175453558 |
| AC096537.1 | -2.483639385 | AC005972.3 | -2.353987335 |
| AC097478.1 | 2.226704683 | AC005993.1 | 5.541912257 |
| AC098617.1 | -1.94642614 | AC006262.1 | -1.736315834 |
| AC099066.2 | 1.674686682 | AC006449.5 | -1.520439262 |
| AC099560.2 | 1.006359897 | AC006504.1 | -2.062484247 |
| AC099850.3 | 1.012022608 | AC006504.8 | 1.081931532 |
| AC103702.2 | -1.230576971 | AC007066.2 | 1.303863235 |
| AC103925.1 | 5.391313885 | AC007240.2 | -1.381232762 |
| AC104072.1 | 5.606197256 | AC007249.1 | -2.92039051 |
| AC104137.1 | 1.887900928 | AC007249.2 | -1.661246902 |
| AC106869.1 | -2.082084698 | AC007285.1 | -2.049478846 |
| AC107021.2 | 1.08366535 | AC007325.2 | -2.598156079 |
| AC108751.5 | 3.481293644 | AC007405.3 | 1.849362454 |
| AC110285.6 | -2.75423072 | AC007495.1 | 3.425772523 |
| AC110285.7 | -1.760703401 | AC007622.1 | -4.876463121 |
| AC112236.2 | -1.995598806 | AC007686.1 | -4.403737575 |
| AC122710.2 | -1.15598969 | AC007686.3 | 1.780918899 |
| AC125232.2 | -1.574233726 | AC007731.3 | 5.910267083 |
| AC127070.1 | -1.896463516 | AC007743.1 | -1.314957491 |
| AC127502.2 | 1.108566081 | AC008013.1 | -2.560766482 |
| AC129492.1 | -1.224271615 | AC008014.1 | -1.229053806 |
| AC137936.2 | 1.002637942 | AC008124.1 | 1.187041169 |
| AC139718.1 | 5.963691533 | AC008147.2 | 2.297354043 |
| AC145207.8 | -1.044427731 | AC008267.3 | -1.412048128 |
| AC211433.1 | 2.756337495 | AC008429.1 | -1.010091418 |
| AC239859.6 | 1.948433632 | AC008440.3 | -4.678608271 |
| AC241952.1 | 1.131832044 | AC008676.3 | -4.624983702 |
| AC243562.2 | 1.83564315 | AC008687.6 | -5.762804582 |
| AC244197.2 | -2.612753711 | AC008691.1 | -2.482987129 |
| AC245100.6 | 1.860158768 | AC008703.1 | -4.505150123 |
| ACAP1 | -1.310466429 | AC008735.2 | 1.250585367 |
| ACBD4 | -1.06083481 | AC008740.1 | -4.512587151 |
| ACHE | -2.22263214 | AC008750.5 | 2.454057939 |
| ACKR3 | 4.796999012 | AC008957.3 | 5.328319198 |
| ACOX2 | 1.504238992 | AC009005.1 | -2.211022297 |
| ACSL4 | 1.927912736 | AC009041.4 | 2.262759927 |
| ACSS3 | -3.561752973 | AC009113.1 | 1.340540512 |
| ACVR1C | 2.954742215 | AC009163.6 | 1.345987912 |
| ADAM11 | -1.241646655 | AC009237.14 | 2.083808361 |
| ADAM12 | 2.053318237 | AC009237.3 | 1.924465132 |
| ADAM23 | 1.626064219 | AC009404.1 | -1.03771041 |
| ADAMTS1 | 2.733149102 | AC009435.1 | 5.075184084 |
| ADAMTS12 | 2.499423875 | AC010186.2 | -1.485941523 |
| ADAMTS16 | -1.384376873 | AC010343.3 | -4.744676533 |
| ADAMTS17 | -2.149661263 | AC010478.1 | 3.837749233 |
| ADAMTS2 | -2.480136123 | AC010536.1 | -1.34675476 |
| ADAMTS6 | 4.999069755 | AC010547.2 | 5.490921485 |
| ADAMTS8 | -2.243790377 | AC010616.1 | -1.553631158 |
| ADAMTSL1 | 4.441161482 | AC010731.2 | 1.697921148 |
| ADAMTSL4-AS1 | 1.058632333 | AC010735.1 | -3.837242611 |
| ADAP1 | -1.170628216 | AC010754.1 | 4.003081504 |
| ADARB1 | 2.216932174 | AC010890.1 | 1.519081426 |
| ADD3 | 2.044525169 | AC010980.1 | 3.086200634 |
| ADGRB1 | -1.177570922 | AC010980.2 | 4.074290559 |
| ADGRE2 | -2.204046781 | AC011450.1 | 1.104809322 |
| ADGRG1 | 1.861333343 | AC011451.3 | -4.506343141 |
| ADGRL2 | 1.202538775 | AC011503.2 | -4.037716409 |
| ADGRL3 | 1.401693878 | AC011504.1 | 5.852693798 |
| ADORA2B | -1.042989212 | AC011604.2 | -3.393088756 |
| ADRA1B | -1.509993999 | AC012313.4 | -1.044962897 |
| ADRA1D | -1.653273531 | AC012313.5 | -1.630341983 |
| ADRA2A | 5.033384859 | AC012313.8 | -1.403574636 |
| ADRB2 | 1.543187423 | AC012358.2 | 1.250856558 |
| AF121898.1 | 4.219623134 | AC012360.3 | 1.135646499 |
| AFAP1L2 | -1.443048394 | AC012414.5 | 2.738921664 |
| AFF3 | 4.020658445 | AC012501.2 | 4.705132363 |
| AFP | 2.690519004 | AC012615.1 | 1.489053157 |
| AGAP1 | 1.410195554 | AC013244.1 | 4.479246415 |
| AGPAT4 | 1.612144786 | AC013268.4 | -4.92012404 |
| AGR2 | 1.266935572 | AC013451.2 | -2.698243285 |
| AGTR1 | 3.051633854 | AC013565.1 | 5.522347832 |
| AHRR | 1.208734505 | AC015849.3 | -1.448865628 |
| AIF1L | -1.438117932 | AC015849.4 | -3.23741893 |
| AK5 | 2.193327129 | AC015849.5 | 1.335253349 |
| AKAP12 | -1.027469559 | AC015912.3 | -1.732996491 |
| AKNAD1 | -2.451313724 | AC016168.1 | 4.570819791 |
| AKR1C1 | 2.27562055 | AC016168.2 | 2.984170745 |
| AL021392.1 | -1.988861596 | AC016168.3 | 3.36376315 |
| AL023284.4 | 1.838322129 | AC016205.1 | 1.189438862 |
| AL035446.1 | 3.043950162 | AC016355.1 | 1.366018423 |
| AL121832.2 | -1.206964036 | AC016717.2 | 3.824432663 |
| AL133325.3 | -1.118737944 | AC016745.2 | -3.03471932 |
| AL133330.1 | 1.502837177 | AC016831.6 | -2.764667417 |
| AL137003.2 | 1.227227933 | AC016876.3 | -1.754775004 |
| AL139393.2 | 2.19224048 | AC016999.1 | -5.092015328 |
| AL353147.1 | 6.453323492 | AC017048.3 | 3.718808855 |
| AL354751.1 | 2.444460683 | AC018413.1 | -1.575744726 |
| AL354822.1 | -2.100658999 | AC018553.1 | 1.811368085 |
| AL355032.1 | -1.214409245 | AC018635.2 | -4.528691841 |
| AL356274.2 | 3.295295214 | AC018638.5 | 1.07313765 |
| AL356414.1 | 3.956481564 | AC018665.1 | -1.120559181 |
| AL358334.2 | 1.324485515 | AC018688.1 | -4.435058017 |
| AL358473.1 | 3.61956365 | AC018797.2 | 1.469621797 |
| AL390726.6 | -2.971939268 | AC019117.1 | -2.711751666 |
| AL391056.1 | 2.213097237 | AC019155.1 | 5.884419182 |
| AL391427.1 | 1.714881016 | AC020571.1 | -2.845842819 |
| AL445309.1 | 1.599005797 | AC020917.4 | 1.115998062 |
| AL513314.2 | 1.167635291 | AC021028.1 | -1.7246785 |
| AL513318.2 | 1.113409174 | AC021205.3 | 2.204901362 |
| AL627230.4 | -4.550583849 | AC021491.2 | -1.544762357 |
| AL645608.7 | 1.07845367 | AC021594.2 | 4.163059967 |
| AL845552.2 | 3.380182941 | AC021683.1 | 5.08859828 |
| ALG1L | -1.448646168 | AC021683.2 | 5.388847067 |
| ALG1L7P | -1.439339953 | AC022007.1 | 2.02860454 |
| ALOXE3 | -1.279118991 | AC022079.1 | -4.885880018 |
| ALPK2 | 1.000955431 | AC022154.1 | 1.724516482 |
| ALX4 | 1.199605769 | AC022239.1 | 1.665032603 |
| AMOT | 2.554156501 | AC022306.1 | 3.542244679 |
| AMPH | 2.0430607 | AC022497.1 | 1.415715791 |
| ANGPT1 | 2.76472747 | AC022613.1 | -1.327641909 |
| ANK1 | -2.654192564 | AC022905.1 | 4.359109234 |
| ANKFN1 | -2.00832847 | AC023043.3 | 3.370764856 |
| ANKRD1 | -1.200406921 | AC023137.1 | 6.178430808 |
| ANKRD18A | -1.262811469 | AC023449.2 | -4.70692382 |
| ANKRD2 | -1.103951105 | AC023632.6 | -1.974779483 |
| ANKRD20A5P | -1.082747405 | AC024361.3 | -2.380992407 |
| ANKRD44 | 1.20403045 | AC024884.2 | -4.404716275 |
| ANKS1B | 2.263940739 | AC025031.4 | -2.010439355 |
| ANKS4B | 2.239503043 | AC025048.2 | -1.420222402 |
| ANPEP | 3.710381348 | AC025419.1 | -4.800966348 |
| ANTXR1 | 1.547327024 | AC025580.2 | 2.162613528 |
| ANXA10 | 2.814210057 | AC025627.1 | -2.432264481 |
| ANXA13 | 1.692139437 | AC025884.2 | -1.212568417 |
| ANXA6 | 1.104031618 | AC025917.1 | 2.350232386 |
| AP000662.1 | -1.673963762 | AC026250.1 | -1.858191615 |
| AP001542.3 | 2.961209729 | AC026368.1 | 2.163616887 |
| AP001574.1 | 3.41984632 | AC026462.3 | -4.870724685 |
| AP001972.5 | 1.306972579 | AC026523.1 | 2.692873197 |
| AP001992.1 | 1.286994756 | AC026774.1 | 5.630153093 |
| AP002852.1 | 2.724184394 | AC026785.3 | 2.36327173 |
| AP003119.3 | 1.826414767 | AC026954.1 | -4.452392735 |
| AP003419.2 | 2.430992929 | AC026979.4 | 1.81860183 |
| AP005233.2 | -1.418673857 | AC026992.2 | 1.146300543 |
| APBB1IP | 1.773759202 | AC027117.1 | -1.258032574 |
| APOE | 1.273016783 | AC027117.2 | -1.934892201 |
| AREG | -1.57312837 | AC027290.2 | -1.006446206 |
| ARFGEF3 | -1.046040224 | AC027612.1 | -4.408729411 |
| ARFRP1 | -1.12555948 | AC027627.1 | 3.703670137 |
| ARHGAP21 | 1.031303244 | AC027644.1 | -1.535379851 |
| ARHGAP28 | 1.13042985 | AC027702.1 | -1.709507337 |
| ARHGAP30 | -1.041346873 | AC027763.2 | -2.463738118 |
| ARHGDIG | -2.64715708 | AC027796.4 | -1.758943145 |
| ARHGEF37 | -1.034273101 | AC034213.1 | -4.506694244 |
| ARHGEF6 | 4.118265585 | AC034223.2 | -5.036026235 |
| ARL2BP | -1.313642964 | AC036214.1 | -2.185315997 |
| ARL4C | -1.505769349 | AC037441.1 | -5.841608569 |
| ARLNC1 | 2.687635718 | AC040174.1 | 2.220929157 |
| ARMC12 | -1.460412299 | AC040970.1 | -1.24151068 |
| ARMC3 | -2.234019436 | AC061961.1 | 5.467971777 |
| ARMC4 | 3.169473626 | AC061992.1 | 2.775582851 |
| ARMCX4 | 2.293785584 | AC062017.1 | -1.673461375 |
| ARRDC4 | -1.396417284 | AC062029.1 | 1.692836565 |
| ARSE | -1.16895602 | AC063944.1 | 3.200607241 |
| ARSI | 2.317832257 | AC063952.1 | 2.471821413 |
| ARSJ | -1.027182822 | AC064805.1 | 3.483760981 |
| ARVCF | -2.363614618 | AC064875.1 | 4.263309059 |
| ARX | -1.286748753 | AC066613.1 | 1.007214779 |
| ASB4 | -1.771367425 | AC067930.4 | -2.59680629 |
| ASS1 | 1.769981526 | AC068587.4 | 2.334337138 |
| ATF3 | -1.117171733 | AC068594.1 | -4.508522358 |
| ATL1 | 1.138782169 | AC068620.2 | 3.162383607 |
| ATP6V0D2 | 3.490362046 | AC068647.2 | -1.781447595 |
| ATP6V0E1 | 1.212588826 | AC068700.1 | 5.195265837 |
| ATP6V1FNB | 1.058976274 | AC068831.7 | 1.5825293 |
| ATP8B1 | -1.222462232 | AC068896.1 | -3.92761032 |
| AXIN2 | -1.322055505 | AC069113.2 | 4.018181941 |
| AZU1 | -1.576731711 | AC069277.1 | 5.790007607 |
| B3GALT4 | -1.67275129 | AC072022.2 | 2.355215094 |
| B3GNT3 | 1.098962373 | AC073073.2 | 1.211494869 |
| B4GALNT2 | -5.025253582 | AC073107.1 | -1.220653214 |
| B4GALNT4 | -1.046359772 | AC073111.5 | 1.075940676 |
| BACE1 | 1.287451562 | AC073254.1 | 1.782503398 |
| BAHCC1 | -1.634661145 | AC073316.3 | 4.092238448 |
| BAIAP3 | -1.695061026 | AC073343.2 | -3.054723925 |
| BARX1 | -3.719230329 | AC073365.1 | 2.456363531 |
| BCHE | 1.887424239 | AC074194.2 | 2.046136823 |
| BCL2L10 | 1.438443045 | AC078905.1 | 5.087347642 |
| BCL2L11 | 1.490882508 | AC078925.4 | -1.885837499 |
| BCO1 | -1.410504042 | AC079054.1 | 5.511157146 |
| BDH1 | -1.073833338 | AC079465.1 | 4.568112857 |
| BDKRB1 | 2.174760529 | AC079684.1 | -1.227522085 |
| BDKRB2 | 1.646883784 | AC079921.2 | -1.897351636 |
| BEGAIN | 1.452553597 | AC079949.1 | -2.720539573 |
| BIK | -3.106384165 | AC079949.2 | -1.940903587 |
| BIRC7 | -1.075673831 | AC080013.4 | 1.540228839 |
| BLK | 3.539403319 | AC080023.1 | -1.856005479 |
| BLOC1S1-RDH5 | 1.287504774 | AC080038.1 | 2.11864944 |
| BMP6 | -1.396221592 | AC082651.1 | 5.179928206 |
| BMP7 | -3.583924966 | AC083843.2 | 1.131462092 |
| BMPER | -1.606026235 | AC083843.3 | 1.715073158 |
| BNC1 | -3.062996083 | AC084880.1 | 2.697562371 |
| BNC2 | 1.038076513 | AC087071.2 | -2.898613921 |
| BOLA2B | 3.685891621 | AC087239.1 | -4.61925369 |
| BREA2 | -1.187655421 | AC087277.2 | 5.388757052 |
| BTNL9 | -1.5307262 | AC087385.1 | -1.853523796 |
| BVES | 1.664125893 | AC087482.1 | -2.251055504 |
| BX005266.2 | -1.325146575 | AC087564.1 | 2.373537443 |
| BX088651.4 | -1.378377448 | AC087623.1 | -2.103107298 |
| BX322234.2 | -3.117852056 | AC087623.3 | -4.441369744 |
| C10orf90 | 3.065496291 | AC089983.1 | -5.027389818 |
| C11orf45 | -1.090195038 | AC090001.1 | -3.061614882 |
| C13orf46 | 1.600695529 | AC090061.1 | -2.411355628 |
| C15orf48 | 1.653382497 | AC090152.1 | 3.48870022 |
| C17orf102 | -4.432716752 | AC090192.2 | -2.431016316 |
| C19orf33 | -1.466113921 | AC090204.1 | 1.094146455 |
| C19orf73 | -1.148536024 | AC090246.1 | -2.298183774 |
| C19orf81 | -1.435370265 | AC090409.1 | -2.210142717 |
| C1GALT1C1 | 1.46154871 | AC090502.1 | -1.760667789 |
| C1orf116 | 1.26258562 | AC090673.1 | -4.643016038 |
| C1orf21 | 3.556140197 | AC090809.1 | 5.966689148 |
| C1orf226 | 1.057315338 | AC090833.1 | -5.405918568 |
| C1QL3 | 1.523345497 | AC091057.1 | 1.120780191 |
| C1QL4 | -2.036937555 | AC091133.2 | -1.390838514 |
| C1R | 1.504231963 | AC091133.4 | -1.875486469 |
| C1S | 1.594492834 | AC091212.1 | -4.52891707 |
| C2CD4C | -2.018343636 | AC091563.1 | 1.79957605 |
| C2orf81 | -1.133852447 | AC091804.1 | -5.228024414 |
| C3orf52 | -1.076832316 | AC091931.1 | 4.124201977 |
| C5AR1 | 1.087962066 | AC091948.1 | 9.358698181 |
| C6orf141 | -1.286122555 | AC091982.3 | -1.111073797 |
| C6orf223 | -2.71607216 | AC092422.1 | 6.37547032 |
| CA2 | 1.25976581 | AC092506.1 | 3.272272802 |
| CA8 | -3.341935688 | AC092611.2 | 3.242451108 |
| CACNA1B | -4.442209814 | AC092611.3 | 3.827111194 |
| CACNA1G | -1.40274848 | AC092691.1 | 2.460374731 |
| CACNA1H | -1.290680845 | AC092718.3 | -1.682816996 |
| CACNG4 | -1.252318784 | AC092720.2 | 2.161090408 |
| CACNG6 | -2.523720669 | AC092757.2 | -4.346878424 |
| CACNG7 | -2.74835743 | AC092757.3 | -4.493705972 |
| CACNG8 | -1.809983968 | AC092807.3 | -2.479449407 |
| CADM2 | -4.604720081 | AC092834.1 | 5.197242683 |
| CALCB | 1.614737852 | AC092868.1 | -5.213699459 |
| CALHM5 | 1.569619277 | AC092969.1 | 2.595203243 |
| CALU | 1.06843734 | AC093227.1 | -1.508427243 |
| CALY | -2.634508468 | AC093607.1 | 6.570943991 |
| CAMK2A | 4.662711694 | AC093673.1 | -1.382619714 |
| CAMK4 | 5.139973325 | AC093787.1 | 4.707032733 |
| CAMKV | -2.211993728 | AC093797.1 | 6.257782337 |
| CARMN | 7.087222093 | AC093838.1 | 4.896954294 |
| CASC19 | 1.482253375 | AC093908.1 | 6.964239596 |
| CASKIN1 | -1.243868922 | AC096537.1 | -3.652718473 |
| CASP1 | 3.425428232 | AC096541.1 | 3.964766546 |
| CASP4 | 1.154554955 | AC096677.1 | -2.189544726 |
| CAT | 1.095573223 | AC097534.1 | 3.197285256 |
| CATSPERE | 1.341332673 | AC097534.2 | 3.339848893 |
| CATSPERG | -1.292590801 | AC097717.1 | 2.300296741 |
| CBFA2T3 | -2.161032423 | AC098614.1 | -1.343269964 |
| CBLB | 1.381592778 | AC098617.1 | -2.541158932 |
| CCDC102B | 1.990316905 | AC098851.1 | -2.271023835 |
| CCDC114 | -1.986118829 | AC099489.1 | -1.032626304 |
| CCDC184 | -1.044740582 | AC099494.2 | 3.403691016 |
| CCDC189 | -1.720685345 | AC099521.1 | -3.321942139 |
| CCDC85A | -1.938097971 | AC099786.1 | -2.472575846 |
| CCDC88C | -1.161032328 | AC099786.2 | -2.62232547 |
| CCND2 | -3.897550862 | AC099850.3 | 1.532887316 |
| CCNJL | -1.754063949 | AC100803.2 | 3.898433755 |
| CCR1 | 5.645289 | AC100810.3 | -1.154155633 |
| CD1D | 1.349937545 | AC100821.1 | -1.632642791 |
| CD302 | 1.384718852 | AC100861.1 | -1.23092581 |
| CD36 | 2.247787653 | AC103591.4 | 3.114595512 |
| CD38 | -2.376854216 | AC103702.2 | -1.229691734 |
| CD40 | 1.08531492 | AC103739.2 | 2.966873604 |
| CD55 | 1.421129226 | AC103746.1 | 3.526599511 |
| CD59 | 1.148331688 | AC104051.2 | 10.85706072 |
| CD70 | -2.298948012 | AC104072.1 | 4.716554721 |
| CD74 | 1.629087955 | AC104083.1 | 7.562022302 |
| CDC42BPG | -1.381910631 | AC104109.2 | -1.128213267 |
| CDCP1 | -1.684605429 | AC104137.1 | 2.971760179 |
| CDH10 | -4.916946043 | AC104393.1 | 4.796003962 |
| CDH17 | 1.841004363 | AC104407.1 | 5.08859828 |
| CDH19 | -3.218210795 | AC104461.1 | -5.294212913 |
| CDH22 | -2.940918347 | AC104794.2 | -2.38441308 |
| CDHR1 | -4.548742128 | AC104809.2 | 4.620617178 |
| CDK14 | 2.254347349 | AC104958.2 | 2.692576668 |
| CDYL2 | 3.188670645 | AC105339.5 | 2.291902633 |
| CEACAM6 | 1.535019614 | AC105383.1 | 2.567804635 |
| CELF5 | -1.176650779 | AC106038.1 | 2.449392287 |
| CELSR1 | -1.293735427 | AC106782.1 | -3.482860125 |
| CELSR2 | -1.306366627 | AC106786.1 | 2.832378174 |
| CENPVL3 | -2.866841731 | AC106869.1 | -1.9494954 |
| CERKL | 2.968990528 | AC106881.1 | 4.748005256 |
| CERS1 | -1.956580674 | AC106886.2 | -5.156554647 |
| CFAP46 | -1.640206357 | AC106886.5 | -2.278501883 |
| CFAP57 | -2.911736181 | AC106897.1 | 3.666622131 |
| CFAP58 | 1.713345884 | AC107057.1 | 6.24464219 |
| CFAP58-DT | 3.374796561 | AC107918.4 | 7.447546051 |
| CFAP65 | -1.4114124 | AC107952.2 | 5.268597741 |
| CFI | 3.549198226 | AC107959.3 | -2.635945981 |
| CGAS | -7.942084388 | AC107959.4 | -1.563170019 |
| CGB8 | -1.347299687 | AC108693.1 | -4.317138518 |
| CHD5 | -1.855823711 | AC109466.1 | 3.451235671 |
| CHDH | -1.01078561 | AC110285.2 | 1.273699279 |
| CHGA | -1.851951514 | AC110597.1 | 4.20321517 |
| CHL1 | 7.90454287 | AC112184.1 | 2.738540095 |
| CHMP1B | 1.13709058 | AC112198.2 | 4.313976221 |
| CHMP1B2P | 1.200989014 | AC112236.1 | -4.796829758 |
| CHRDL1 | 5.406778796 | AC112236.2 | -2.105755713 |
| CHRDL2 | 2.856636209 | AC112484.1 | -1.092682292 |
| CHRM3 | -4.480269797 | AC113383.1 | 2.558064192 |
| CHRNA9 | 2.129990681 | AC114550.1 | -5.287615232 |
| CHRNB2 | -1.385081274 | AC114947.1 | -4.526325209 |
| CHST4 | 2.726423855 | AC115618.3 | 1.068249734 |
| CHSY3 | 1.57046724 | AC116021.1 | -2.046713104 |
| CIDEC | 2.801260425 | AC116351.1 | 2.207058936 |
| CKB | -1.801685014 | AC116614.1 | 5.369970005 |
| CKMT1B | -2.40149686 | AC116667.1 | 1.946559301 |
| CLDN1 | -1.221419393 | AC120024.1 | -4.212339619 |
| CLDN2 | 1.50338448 | AC120049.1 | 2.317601516 |
| CLDN3 | -2.450166951 | AC123768.5 | 2.344712315 |
| CLDND1 | 1.019468547 | AC124067.4 | -3.427727104 |
| CLEC2B | 1.984448599 | AC124276.1 | -3.165463396 |
| CLIC2 | 2.439255416 | AC124312.6 | -2.946257394 |
| CLMP | 3.629899354 | AC124944.2 | 1.461409117 |
| CLVS2 | 1.963429041 | AC125232.2 | -1.826569453 |
| CMAHP | 1.657761508 | AC125807.2 | -1.601778098 |
| CNIH3 | -1.282114289 | AC126696.1 | -4.419522323 |
| CNKSR2 | -2.241762148 | AC127024.4 | -5.296624067 |
| CNR1 | 2.733276846 | AC127024.5 | -2.713701515 |
| CNRIP1 | 1.8096461 | AC127024.8 | -5.285739939 |
| CNTNAP2 | 4.665000097 | AC127070.1 | -3.25809131 |
| CNTNAP3 | -1.232756656 | AC127496.2 | -4.690569911 |
| CNTNAP3B | -1.786041089 | AC127496.5 | -4.796615654 |
| CNTNAP3C | -2.761298819 | AC127496.7 | -1.217492948 |
| CNTNAP3P2 | -1.665731085 | AC127502.2 | 1.489320605 |
| COL12A1 | 1.363027288 | AC129492.1 | -1.534392368 |
| COL13A1 | -2.632146881 | AC131009.3 | -1.630369879 |
| COL14A1 | 4.45621095 | AC131212.3 | -1.156587341 |
| COL15A1 | 3.735859378 | AC131235.4 | 4.136895688 |
| COL21A1 | -2.002403587 | AC131532.1 | -3.833901896 |
| COL3A1 | 3.985769675 | AC132008.2 | 1.016491662 |
| COL4A3 | 1.047381481 | AC132938.3 | -1.086553374 |
| COL4A4 | 1.222333979 | AC134043.2 | 5.492061387 |
| COL4A6 | -1.552836951 | AC135050.3 | -2.418445624 |
| COL5A3 | 4.680835025 | AC135050.5 | 1.899090114 |
| COL6A1 | 2.217864548 | AC136475.1 | 3.05097296 |
| COL6A2 | 2.980590182 | AC136475.2 | 2.071117414 |
| COL8A1 | 8.774685249 | AC136475.5 | 3.094600936 |
| CPED1 | 5.497300334 | AC137630.3 | -2.290817785 |
| CPQ | 2.052794495 | AC137894.1 | -2.824828059 |
| CPS1 | -2.723114766 | AC137936.2 | -2.08024608 |
| CPVL | 2.405009358 | AC138028.2 | -1.659031799 |
| CREB3L1 | 2.89102919 | AC138207.2 | -2.270272663 |
| CREB3L2 | 1.331107062 | AC138393.3 | -1.520690343 |
| CREG1 | 1.276771947 | AC139149.1 | -4.523373882 |
| CRIP2 | -1.242243959 | AC139530.1 | 1.344157221 |
| CRISPLD1 | 3.792392079 | AC139718.1 | 5.647284137 |
| CRISPLD2 | 4.003282882 | AC139718.2 | 4.456547271 |
| CRTAC1 | -2.562135393 | AC139749.1 | -2.469270279 |
| CRYAB | 1.647875054 | AC142381.4 | 5.005353469 |
| CRYBG1 | 1.221421792 | AC144652.1 | -1.11744118 |
| CRYBG2 | 2.984328324 | AC144831.1 | 2.661016299 |
| CSF2RA | -2.303837819 | AC145676.1 | 3.69825509 |
| CSPG5 | -1.115805244 | AC156455.1 | -2.11250118 |
| CSRNP3 | 2.178557419 | AC207130.1 | -5.905793321 |
| CT62 | -2.449601954 | AC211433.1 | 4.289763301 |
| CTH | 1.758355623 | AC213203.2 | -2.280764456 |
| CTSA | 1.144791725 | AC233280.2 | 1.309652079 |
| CTSB | 1.1452772 | AC239859.6 | 1.824838201 |
| CTSD | 1.067747066 | AC242842.1 | 2.116790596 |
| CTSO | 1.222218797 | AC243562.1 | 1.373501212 |
| CTXN1 | -2.262052427 | AC243562.2 | 1.613262952 |
| CUL4B | 1.813008098 | AC243960.3 | 2.211199471 |
| CUX2 | -4.839460243 | AC243964.2 | 1.696841049 |
| CXCL5 | -1.030637481 | AC243964.3 | -1.08075273 |
| CXorf57 | 1.739939073 | AC244090.2 | 1.924029675 |
| CXXC5 | 2.426079372 | AC244197.2 | -2.909725567 |
| CYBRD1 | 1.445078253 | AC244230.2 | -2.002128544 |
| CYFIP2 | -1.03349696 | AC245041.1 | -2.918795511 |
| CYP1B1 | 1.632346746 | AC245041.2 | -3.313133543 |
| CYP2J2 | 1.71252519 | AC245060.6 | 1.384271804 |
| CYP2S1 | -1.156847533 | AC245297.3 | 1.15667732 |
| CYP2U1 | 1.024000922 | AC254633.1 | -4.776157993 |
| CYP2W1 | -1.017626677 | ACADL | 1.32486643 |
| CYP4F11 | 1.619155043 | ACAP1 | -1.34693972 |
| CYP7B1 | 1.592616093 | ACAP3 | 1.192934398 |
| DAB2 | 1.815791726 | ACBD4 | -1.301596199 |
| DACT2 | -2.925697503 | ACHE | -1.095198241 |
| DAW1 | 1.328607115 | ACKR1 | 4.794888708 |
| DDR2 | 5.620420301 | ACKR3 | 5.166765151 |
| DENND2A | -1.971687968 | ACOT11 | -1.172276871 |
| DGKI | 4.58856728 | ACOT12 | 3.537047851 |
| DHCR24 | 1.213617163 | ACOT9 | -1.479592771 |
| DHFRP1 | 1.047030568 | ACOX2 | 2.293773869 |
| DHRS13 | -1.336760572 | ACP5 | 1.496679513 |
| DHRS2 | -2.724701797 | ACPP | 4.658636901 |
| DIO2 | 1.068084893 | ACSL4 | 2.002559016 |
| DIO3OS | -1.696423552 | ACSS3 | 3.740413189 |
| DIRAS2 | 6.231050254 | ACTBP13 | -4.953409971 |
| DKK1 | -1.606848197 | ACTG2 | 5.540721029 |
| DKKL1 | -1.645397122 | ACTL6B | -3.4405828 |
| DLC1 | 1.915755719 | ACTN1 | -1.034056329 |
| DLG2 | 1.973114724 | ACTRT3 | 1.272660466 |
| DLX1 | 2.641049143 | ACVR1C | 2.678843776 |
| DLX2 | 4.19514362 | ACVRL1 | 2.751651714 |
| DMD | 3.523065457 | ADAM11 | -1.781365984 |
| DNAAF1 | -1.384670352 | ADAM12 | 1.839133831 |
| DNAAF3 | -1.480681114 | ADAM22 | 1.287527288 |
| DNAH11 | -1.728310011 | ADAM23 | 2.561601448 |
| DNAH17-AS1 | 1.984964753 | ADAM28 | 2.581087456 |
| DNAH5 | -1.299441424 | ADAM7 | 4.068113131 |
| DNAH7 | -1.514812151 | ADAMTS1 | 5.271247114 |
| DND1P1 | -2.093727805 | ADAMTS12 | 2.002934149 |
| DNER | 2.728882911 | ADAMTS14 | 1.724397962 |
| DNM1P51 | 1.430708623 | ADAMTS16 | -1.399178071 |
| DOC2B | -3.246880404 | ADAMTS18 | -4.610182921 |
| DOCK11 | 1.671072664 | ADAMTS19 | 5.253490128 |
| DOCK8 | 1.92459158 | ADAMTS2 | -3.814630621 |
| DPYSL5 | -1.598340146 | ADAMTS3 | 5.299423544 |
| DRC7 | -3.24135301 | ADAMTS5 | 5.009793354 |
| DRD1 | -2.638941908 | ADAMTS6 | 3.867089095 |
| DSE | 1.51018227 | ADAMTS8 | 1.282076545 |
| DSEL | 2.320449753 | ADAMTS9 | 4.11257755 |
| DUSP8 | -1.167556108 | ADAMTS9-AS1 | 2.64797231 |
| DUSP9 | -1.281717933 | ADAMTS9-AS2 | 5.524418372 |
| DUX4L9 | -2.396627862 | ADAMTSL1 | 8.870151953 |
| DYNC1I1 | 3.658023806 | ADAMTSL3 | 3.978394537 |
| DYSF | -1.758927732 | ADAMTSL4-AS1 | -2.395355359 |
| EBF1 | 2.115810132 | ADAP1 | -1.368703765 |
| ECM1 | 1.346328114 | ADARB1 | 2.794715946 |
| EDIL3 | 3.282103327 | ADARB2 | 7.025019895 |
| EDN1 | -1.413856905 | ADCY1 | -1.143168984 |
| EDNRA | 1.780390613 | ADCY10P1 | 1.047170246 |
| EDNRB | 5.055825338 | ADCY5 | -1.026455805 |
| EEF1A2 | -1.561582243 | ADCY9 | 1.441548792 |
| EFCAB12 | -1.53930159 | ADCYAP1 | 7.096672811 |
| EFEMP1 | 2.808373561 | ADCYAP1R1 | 2.369134156 |
| EFHD1 | -1.53276671 | ADD3 | 4.005382947 |
| EFNA1 | 1.467023363 | ADD3-AS1 | 2.956776686 |
| EFNB3 | -1.150897763 | ADGRA2 | 1.247943006 |
| EGF | 2.192022252 | ADGRA3 | 1.422556992 |
| EGFR-AS1 | -2.072573858 | ADGRB1 | 1.551094075 |
| ELF3 | -1.969927453 | ADGRB3 | 5.489542814 |
| ELL2 | 1.324687693 | ADGRD1 | 1.403796814 |
| EMC6 | -1.145996456 | ADGRD1-AS1 | -1.983097685 |
| EML5 | -1.185937236 | ADGRD2 | -3.145861327 |
| EMP1 | 2.004159574 | ADGRE2 | -1.936903727 |
| EN1 | -2.264417877 | ADGRF4 | -3.83811211 |
| ENDOG | -1.033898108 | ADGRL1 | 1.024269143 |
| ENKUR | -1.867931551 | ADGRL3 | 3.086922556 |
| ENOX1 | 1.641425797 | ADGRV1 | 5.271867283 |
| ENPP4 | 1.012815098 | ADH4 | 5.329192013 |
| EOMES | -1.369190114 | ADHFE1 | 4.467257637 |
| EPB41L4A | -1.064586255 | ADIRF-AS1 | -1.475372137 |
| EPHA10 | -1.326096476 | ADM | -1.032613052 |
| EPHA2 | -1.175047733 | ADM5 | 1.085656485 |
| EPHA5 | 2.098629776 | ADORA1 | 1.536384759 |
| EPOP | -1.257926108 | ADORA2A | -3.450919603 |
| EPS8 | 1.242170516 | ADORA2A-AS1 | -2.304039581 |
| EPS8L3 | 1.612582696 | ADORA2B | -2.169236907 |
| ERBB3 | 1.51543586 | ADPRM | -1.094097051 |
| ERCC6 | 1.078175418 | ADRA1A | 3.483463704 |
| EREG | -1.709424297 | ADRA1B | -1.517746477 |
| ERGIC1 | 1.467117082 | ADRA2A | 4.680726781 |
| ERICH5 | -1.015924256 | ADRB1 | -2.607081745 |
| ERRFI1 | -1.385277934 | ADRB2 | 2.975040456 |
| ERV3-1 | 1.069983401 | ADSSL1 | 2.076223875 |
| ERVMER34-1 | -1.77054889 | AEN | -1.286058065 |
| ETS1 | -1.221869418 | AF038458.3 | -3.92761032 |
| EVA1C | 1.093975456 | AF106564.1 | 5.172403837 |
| EVI2B | 2.947115777 | AF111167.2 | -1.356817573 |
| EXO5 | -1.844073904 | AF121898.1 | 7.389578384 |
| EXOC3L2 | -1.647622083 | AF131215.5 | 3.427669965 |
| EXOC3L4 | -1.084411782 | AFAP1L2 | -3.228338357 |
| EXPH5 | -1.918158069 | AFF2 | 6.400939553 |
| EYA2 | -1.995526198 | AFF3 | 3.894050961 |
| F2RL1 | -1.333232987 | AFP | 3.792906341 |
| F2RL2 | 1.427824323 | AGAP2-AS1 | -1.840542798 |
| F5 | 6.411322507 | AGBL1 | 4.84535532 |
| F8A2 | -1.1273232 | AGER | 1.001467769 |
| FAAHP1 | -1.696237088 | AGFG2 | -1.249243525 |
| FAM110C | -2.240464412 | AGPAT4 | 1.453303504 |
| FAM114A1 | 1.418753526 | AGPAT4-IT1 | 2.403955827 |
| FAM13C | 3.386888359 | AGTR1 | 3.07853137 |
| FAM155A | -2.669894557 | AGTR2 | 5.961097311 |
| FAM155B | -1.183398432 | AHCYL1 | 1.917381207 |
| FAM160A1 | 1.385277682 | AHNAK | -1.462421657 |
| FAM167A | 2.206275564 | AHNAK2 | -2.243052152 |
| FAM171A2 | -1.038247494 | AIFM3 | -5.098817667 |
| FAM171B | 1.130646061 | AJ239328.1 | 4.066573032 |
| FAM174B | -1.463554629 | AJUBA | -1.684421374 |
| FAM189A1 | -1.948999074 | AK3P5 | -2.205902823 |
| FAM189A2 | -1.670662427 | AK4 | 1.147431072 |
| FAM198B | 1.848357113 | AK5 | -1.061331362 |
| FAM19A5 | -1.516643684 | AKAP12 | -3.52495028 |
| FAM201A | -1.228115412 | AKNAD1 | -3.969370583 |
| FAM224B | -4.025131862 | AKR1B1 | -1.61289023 |
| FAM43A | 1.919992748 | AKR1B10 | -1.648033818 |
| FAM83A | 2.242546123 | AKR1C1 | 1.764237674 |
| FAM83A-AS1 | 3.167469822 | AKTIP | -1.076871298 |
| FAM84B | -1.016072541 | AL008707.1 | 6.052926033 |
| FAM95C | -1.54489129 | AL021154.1 | -2.131043674 |
| FAP | 6.208474175 | AL021392.1 | -1.841148722 |
| FBLN1 | 1.27139073 | AL021997.1 | 3.597588425 |
| FBLN2 | 3.65716569 | AL023284.4 | 1.831317968 |
| FBN1 | 1.737956346 | AL023803.2 | 4.810551829 |
| FBXL7 | 2.139842867 | AL024508.2 | 2.561430491 |
| FBXO32 | 1.784058915 | AL031009.1 | -1.432879747 |
| FDXR | -1.327472112 | AL031600.1 | 1.834228428 |
| FENDRR | 8.214521705 | AL031665.2 | 3.386731459 |
| FGA | -1.346239947 | AL031728.1 | -1.950907481 |
| FGF12 | 1.186549609 | AL031729.1 | 2.084051828 |
| FGF22 | 1.551884644 | AL031775.1 | 2.525119832 |
| FGF9 | 5.045603298 | AL031985.3 | -1.381524886 |
| FGFR2 | -1.425550589 | AL033397.1 | -1.471148532 |
| FGG | -2.587447314 | AL033397.2 | 1.884906726 |
| FHDC1 | 3.451675572 | AL033530.1 | 2.558431852 |
| FHOD3 | 2.033981469 | AL034374.1 | 5.380277715 |
| FIBCD1 | -1.018509482 | AL035563.1 | 1.136144865 |
| FIBIN | 2.188917151 | AL035665.1 | 3.525137871 |
| FILIP1 | 1.70436933 | AL049629.1 | -4.705979303 |
| FJX1 | -1.614980208 | AL049629.2 | -4.327732343 |
| FKBP7 | 1.394621308 | AL049795.1 | -2.606713361 |
| FLJ16779 | -2.807252221 | AL049796.1 | 1.728457739 |
| FLNC | 3.854300261 | AL049838.1 | 4.064082202 |
| FLRT2 | -3.699834809 | AL078590.3 | 3.658862715 |
| FLRT3 | -1.041379845 | AL096828.1 | -4.967138099 |
| FLT4 | -1.936351252 | AL109763.1 | 5.272126568 |
| FMN1 | -1.287106484 | AL109976.1 | 1.509822278 |
| FN1 | 3.035564657 | AL121753.2 | -1.240884683 |
| FN3K | -1.057131392 | AL121772.1 | -2.004053719 |
| FNDC1 | 1.659359769 | AL121772.3 | -1.594705372 |
| FNDC3A | 1.036093425 | AL121839.2 | 2.907746634 |
| FOXA2 | -1.788181607 | AL121949.2 | -1.826718186 |
| FOXA3 | -1.929318236 | AL132712.1 | -4.795712043 |
| FOXC2 | 1.094082348 | AL133215.2 | 1.41893647 |
| FOXD3-AS1 | -1.072496769 | AL133297.2 | -5.105076127 |
| FOXF1 | 9.12184453 | AL133367.1 | 1.407982766 |
| FOXL2 | -3.42875069 | AL133373.2 | 3.883621192 |
| FOXL2NB | -2.326609346 | AL133467.2 | 2.995654523 |
| FOXO1 | 1.253913639 | AL136018.1 | 3.69974215 |
| FOXP2 | 2.267529523 | AL136084.3 | 7.28685875 |
| FOXQ1 | -1.517341128 | AL136116.3 | 2.926719032 |
| FPR1 | 1.013243696 | AL136964.1 | 8.233646451 |
| FRAS1 | -1.42587382 | AL136988.2 | 2.657901041 |
| FRMD5 | -1.216433776 | AL137002.1 | 2.019595922 |
| FSIP1 | -1.065932137 | AL137003.2 | 1.004953675 |
| FSTL1 | 2.596321881 | AL137145.1 | -6.069439014 |
| FSTL4 | -1.334594751 | AL137145.2 | -5.610136741 |
| FYN | 3.090696962 | AL138828.1 | -1.761135024 |
| GAB1 | 1.174947611 | AL139132.1 | 4.576721002 |
| GAB3 | 2.738220107 | AL139220.2 | -1.330378031 |
| GABARAPL1 | 1.201588076 | AL139289.2 | -1.57532141 |
| GABRA2 | 6.896155429 | AL139384.1 | -1.585829343 |
| GABRA5 | 1.372009656 | AL139393.2 | 1.795676593 |
| GABRD | -4.084154122 | AL139424.2 | 2.217066501 |
| GABRE | 1.218135396 | AL157387.1 | -5.816238578 |
| GABRQ | 2.222325854 | AL157823.2 | -1.990055436 |
| GADD45A | -1.036093429 | AL158071.4 | 2.850249774 |
| GAL | -3.871854045 | AL158209.1 | -2.543531455 |
| GAL3ST1 | 1.036163393 | AL160254.1 | -3.170756715 |
| GALNT15 | 4.591436828 | AL160269.1 | -4.515876139 |
| GALNT16 | -1.600613009 | AL160286.1 | 3.828248842 |
| GALNT17 | 1.27834029 | AL161421.1 | 1.380307043 |
| GALNT18 | -1.100492201 | AL161431.1 | -2.929071475 |
| GALNT2 | 1.21146137 | AL161668.1 | 4.260151996 |
| GALNT5 | 1.5285289 | AL161908.1 | 3.800870763 |
| GAP43 | 5.101095873 | AL162411.1 | -1.62272651 |
| GAS1 | 8.282138139 | AL162582.1 | -4.00377657 |
| GATA2 | 1.314171411 | AL353147.1 | 5.713690876 |
| GATA3 | -1.132952918 | AL353622.1 | 1.16949438 |
| GATA6 | -1.22273761 | AL353807.3 | -1.789383411 |
| GATA6-AS1 | -1.895853773 | AL354718.1 | -2.610300557 |
| GBP1 | 2.596269361 | AL354718.3 | -4.336636068 |
| GBP3 | 1.054264577 | AL354740.1 | -2.426245367 |
| GBP5 | 2.199666799 | AL354892.2 | 1.304315303 |
| GCNT1 | 1.785244224 | AL354920.1 | 1.197187209 |
| GCOM1 | 1.019592638 | AL355032.1 | -1.443389679 |
| GDA | 2.313719618 | AL355388.2 | -1.5861804 |
| GDF15 | -1.352831142 | AL355472.1 | 1.618894329 |
| GDPD3 | 1.886012254 | AL355488.1 | 1.511336994 |
| GFRA1 | -2.482202016 | AL355864.2 | -2.30370708 |
| GGT4P | 1.001859282 | AL355994.2 | -4.276566012 |
| GGT5 | 2.466682324 | AL356274.2 | 3.292513426 |
| GHR | 2.945670285 | AL356356.1 | -3.758413502 |
| GIPC3 | 2.771519869 | AL356414.1 | 3.147743382 |
| GIPR | -2.01887877 | AL356417.2 | 6.520764511 |
| GJA3 | -4.144396026 | AL356488.3 | 1.594113828 |
| GJD3 | -1.977223239 | AL356585.2 | -7.662590647 |
| GLB1L2 | -1.474351184 | AL356740.1 | 5.470174721 |
| GLB1L3 | -3.258422719 | AL357060.1 | 2.412211114 |
| GLDN | 3.870912543 | AL357992.1 | -4.45472189 |
| GLI1 | -1.191907921 | AL358334.2 | 3.945944508 |
| GLI2 | -1.463378215 | AL358472.5 | -1.512214056 |
| GLI3 | 4.526108752 | AL359183.1 | -3.085537592 |
| GLIPR1 | 1.529142958 | AL359399.1 | 2.160640951 |
| GLIPR2 | 1.432054999 | AL359504.2 | 1.025714966 |
| GLIS1 | 6.387940307 | AL359636.1 | -2.057689086 |
| GLRB | -1.267732247 | AL359715.2 | 1.903903107 |
| GLUL | -4.254439049 | AL359851.1 | 4.11224226 |
| GM2A | 1.034285683 | AL359853.1 | 5.732825078 |
| GNAO1 | 2.487875753 | AL359915.2 | -1.436493649 |
| GOLT1A | -1.358944683 | AL365181.4 | -2.998659117 |
| GPAM | 2.268682589 | AL365436.2 | -4.528691841 |
| GPC2 | -1.490225601 | AL390066.1 | 2.075015428 |
| GPC3 | 7.853216353 | AL390719.1 | -1.014390138 |
| GPC4 | 2.459961055 | AL390726.6 | -4.825902397 |
| GPC5 | 9.123097486 | AL390783.1 | 4.686637484 |
| GPC6 | 1.365443898 | AL390879.1 | -1.093519142 |
| GPER1 | 2.335864903 | AL391001.1 | -1.49486556 |
| GPNMB | 3.973153544 | AL391056.1 | 4.005357571 |
| GPR1 | 1.811846243 | AL391069.2 | -3.005056588 |
| GPR137C | 2.130907608 | AL391427.1 | 1.812190216 |
| GPR162 | -4.311602648 | AL391684.1 | 1.461475718 |
| GPR176 | 1.473803522 | AL391730.1 | 5.100516747 |
| GPR37 | 1.476397243 | AL391807.1 | 4.538703383 |
| GPRC5B | -1.239115948 | AL441992.1 | 1.295325123 |
| GPS2 | -1.148772248 | AL445250.1 | 2.085977028 |
| GPX2 | 1.854549318 | AL445309.1 | 1.754585948 |
| GPX3 | 1.397716344 | AL445584.2 | -5.986838123 |
| GRAMD1B | 1.211831978 | AL445623.2 | 3.128081121 |
| GREB1L | 1.160164023 | AL445649.1 | 9.311347639 |
| GREM1 | -7.859398602 | AL450992.1 | -3.465395295 |
| GREM2 | 1.038700127 | AL450992.2 | 6.386220783 |
| GRHL3 | -1.870812862 | AL451069.3 | -1.033278099 |
| GRIK2 | 1.367340639 | AL512622.1 | 2.049255637 |
| GRIP2 | -1.442957867 | AL513318.2 | 2.381689606 |
| GSEC | 1.479680743 | AL513327.1 | -1.116462894 |
| HAGLR | 2.661577733 | AL513534.1 | 3.458565231 |
| HAS3 | -1.35569819 | AL589987.1 | 2.171928016 |
| HBQ1 | -1.045625706 | AL590004.3 | -2.655220078 |
| HCN2 | 1.031070528 | AL590560.1 | 1.706362706 |
| HDAC4 | 1.298782233 | AL590652.1 | 1.529192321 |
| HES2 | -1.615734027 | AL592148.2 | 1.427096153 |
| HES6 | -1.24751822 | AL596223.2 | -4.66231738 |
| HES7 | -2.068691284 | AL596244.1 | 1.071414146 |
| HEXIM2 | -1.096290788 | AL603756.1 | -1.600431106 |
| HEY2 | 1.262650942 | AL606534.4 | -1.270177047 |
| HGD | 2.184789798 | AL606534.5 | -5.036803003 |
| HHIP | -2.194897913 | AL606763.1 | -1.552195386 |
| HHIPL2 | 1.30469024 | AL606970.1 | -5.405126322 |
| HIC1 | -1.383420175 | AL627422.2 | -5.22778854 |
| HID1 | -1.471868406 | AL645608.1 | -1.557751506 |
| HIF1A-AS1 | 1.640796242 | AL645608.6 | 1.194977798 |
| HIST1H2BK | 1.003527933 | AL645940.1 | 1.938190848 |
| HIST1H4I | 1.216596348 | AL663070.1 | 4.455761268 |
| HKDC1 | -1.038361183 | AL683807.1 | -4.802664184 |
| HLA-DMA | 1.321934066 | AL683887.1 | 4.567895336 |
| HLA-DPA1 | 5.526130164 | AL691432.2 | 1.414379906 |
| HLA-DRA | 3.64231713 | AL691520.1 | 4.589318284 |
| HLA-F | 1.222424861 | AL731533.2 | -2.087608584 |
| HMGA2 | -1.476009222 | AL731577.2 | -1.412755371 |
| HMX1 | -5.73668357 | AL772337.3 | -3.607699713 |
| HNRNPA1P27 | 2.64920564 | AL845552.2 | 3.550248053 |
| HOXA1 | 1.197371382 | ALDH1A1 | -4.182529841 |
| HOXA5 | 1.273440074 | ALDH1A2 | 2.18106596 |
| HOXA-AS2 | 1.273802615 | ALDH1A3 | -1.31099306 |
| HOXB2 | -2.6224679 | ALDH1L1 | 3.263780333 |
| HOXB-AS1 | -2.85409412 | ALDH3A1 | -2.555895213 |
| HOXD8 | 1.307608773 | ALDH4A1 | -1.334412656 |
| HPCAL1 | 1.005125904 | ALG1L | -1.628253276 |
| HPD | 3.691039848 | ALG1L10P | -1.408725145 |
| HPGD | -1.303679717 | ALG1L13P | -1.6632927 |
| HRK | -1.157214777 | ALG1L2 | -1.563713148 |
| HS3ST6 | -3.025433483 | ALG1L7P | -2.786008729 |
| HSPA2 | 1.186274438 | ALG1L8P | -1.694331101 |
| HSPA8 | 1.126009499 | ALG1L9P | -1.967490638 |
| HSPB1P2 | -3.353029313 | ALKBH2 | -1.686270244 |
| HSPB8 | 1.142334508 | ALKBH3 | -1.086555773 |
| HTR6 | 1.584505694 | ALKBH8 | -1.694508475 |
| HTRA1 | 1.874596268 | ALOX12P2 | -1.393287376 |
| HUNK | -1.740209575 | ALOX15 | 2.4312218 |
| HYAL1 | 1.749694962 | ALOX5 | 1.71800475 |
| HYDIN | -1.550518768 | ALOXE3 | -5.89199666 |
| ICA1 | -1.052957201 | ALPI | 4.463419321 |
| ICAM5 | -1.273326455 | ALPK2 | -1.323567298 |
| ID4 | 1.616711977 | ALPK3 | -1.413254154 |
| IDI1 | 1.148947993 | ALPL | 1.481004333 |
| IER5 | -1.239772195 | ALS2CL | -3.634192444 |
| IFFO1 | 1.21468775 | ALX4 | 2.733353022 |
| IFFO2 | -1.020360539 | AMBP | 3.114153643 |
| IFI16 | 2.631175959 | AMHR2 | -4.136597482 |
| IFI44 | 2.093420968 | AMIGO2 | -2.470402115 |
| IFI44L | 2.779376581 | AMMECR1 | 1.351977367 |
| IFITM10 | 1.254774219 | AMN | -1.00770265 |
| IGDCC4 | -1.746112581 | AMN1 | -1.319065561 |
| IGF2BP3 | 1.193070474 | AMOT | 6.729921691 |
| IGFBP2 | -1.239102841 | AMOTL2 | -1.990486898 |
| IGFBP5 | 2.740991786 | AMPD3 | -2.579408797 |
| IGFBP7 | 3.44085716 | AMPH | 4.564278983 |
| IGFBPL1 | -3.135973743 | AMTN | 4.840054319 |
| IGFL2 | -1.114545619 | AMY2B | 1.0845511 |
| IGFL2-AS1 | -3.969677492 | ANGPT1 | 4.63821071 |
| IGFN1 | 3.34280123 | ANGPT4 | -1.438460414 |
| IGSF10 | 3.293021801 | ANGPTL1 | 3.412019266 |
| IGSF9B | -1.373949792 | ANGPTL4 | 1.121738535 |
| IL10RB | -1.708628226 | ANK1 | -2.998980823 |
| IL11 | 2.192292079 | ANK3 | 1.374488725 |
| IL13RA2 | 4.559370829 | ANKH | 1.127871208 |
| IL1B | 3.199933786 | ANKRD1 | -6.90153029 |
| IL1R1 | 1.167182966 | ANKRD13B | -1.139596665 |
| IL1RAP | 1.340427309 | ANKRD18A | -1.107401982 |
| IL1RN | 5.242501185 | ANKRD18B | -1.044211771 |
| IL31RA | 1.068347077 | ANKRD19P | 2.039472088 |
| IL33 | 6.653266229 | ANKRD2 | -2.445971567 |
| ILDR2 | -2.216313352 | ANKRD20A10P | 3.163434901 |
| IMPA2 | 1.914016843 | ANKRD20A5P | -3.99093546 |
| INA | -1.277837211 | ANKRD30A | -6.130836896 |
| INHBA | 6.808767951 | ANKRD34B | 2.47704723 |
| INHBA-AS1 | 3.208233309 | ANKRD36BP2 | 5.677212764 |
| INPP5J | -1.205259072 | ANKRD42 | -1.413329325 |
| INSIG1 | 1.655649925 | ANKRD44 | 2.040384947 |
| INSL4 | -2.351958071 | ANKRD55 | -1.290599859 |
| INSYN1 | 1.142118496 | ANKS1B | 2.399714564 |
| INSYN2 | 1.107120512 | ANKS3 | -1.279488393 |
| IQGAP2 | 1.481682935 | ANO3 | 7.196226565 |
| ISYNA1 | -1.844729755 | ANO4 | 2.612420369 |
| ITGA1 | 2.35304863 | ANO7L1 | -1.745794686 |
| ITGA11 | 2.81798549 | ANO9 | -2.018330426 |
| ITGA4 | 4.029776887 | ANOS1 | 7.063137681 |
| ITGB3 | 4.472223676 | ANPEP | -2.556466339 |
| ITGBL1 | -1.229813068 | ANTXR1 | 2.426158088 |
| ITIH2 | 1.220392575 | ANXA1 | -1.453956822 |
| ITPKA | -1.173315487 | ANXA10 | 1.042225586 |
| ITPKB | -2.603367464 | ANXA13 | -7.886564984 |
| ITPR1 | 2.506476551 | ANXA2 | -1.04874259 |
| ITPRIP | 1.823331062 | ANXA2P2 | -1.293089474 |
| JAG2 | -1.23662602 | ANXA3 | -3.727339932 |
| JAKMIP2 | 6.371256879 | ANXA4 | -1.304635648 |
| JAZF1 | 2.327026804 | ANXA6 | 1.532624006 |
| JCAD | 1.024141085 | ANXA8 | -4.636689672 |
| JPH1 | 3.03409496 | ANXA8L1 | -3.393790624 |
| KALRN | 2.675770986 | AOC2 | -2.47745369 |
| KANK1 | 1.074536181 | AOC3 | -2.293089974 |
| KANK3 | -1.664316621 | AOX1 | -4.746254776 |
| KANK4 | 4.855329951 | AP000346.2 | 2.412669463 |
| KAZN | 1.760719868 | AP000439.2 | -6.299320198 |
| KCNB1 | -1.052874513 | AP000547.3 | 6.791136704 |
| KCND2 | 4.830447711 | AP000577.1 | 2.071986905 |
| KCNE4 | 6.333348954 | AP000662.1 | -2.365469453 |
| KCNF1 | -1.259469692 | AP000695.2 | -3.527876226 |
| KCNH2 | -1.646341261 | AP000696.1 | -5.915519834 |
| KCNH3 | -2.41722517 | AP000759.1 | -1.591000723 |
| KCNH4 | -1.241673235 | AP000820.2 | 5.381604187 |
| KCNJ16 | 1.996911227 | AP001029.2 | -1.097104103 |
| KCNJ6 | 1.96377785 | AP001148.1 | -1.142172332 |
| KCNJ8 | 2.376304295 | AP001347.1 | -1.408043235 |
| KCNK5 | 1.266359901 | AP001453.2 | -1.737413242 |
| KCNK6 | -1.306180753 | AP001527.2 | -2.439771173 |
| KCNMA1 | 1.577524332 | AP001542.3 | 3.013101732 |
| KCNMA1-AS1 | 2.43030966 | AP001781.1 | 2.591554853 |
| KCNQ1 | -1.668797961 | AP001830.1 | -2.331687347 |
| KCNQ2 | -2.563570118 | AP001972.5 | 1.256040902 |
| KCNS3 | -1.661025211 | AP002495.1 | -1.243015947 |
| KDELC2 | 1.635771081 | AP002762.1 | -4.405913554 |
| KIF17 | -1.407575911 | AP002851.1 | -1.555345811 |
| KIF1A | -4.196933967 | AP002852.1 | 4.789757818 |
| KIF21B | 1.517223979 | AP002957.1 | -5.745311236 |
| KIRREL2 | -1.651789632 | AP003071.5 | -3.00863617 |
| KIT | 6.276042693 | AP003119.1 | 1.744440237 |
| KLC3 | -2.285742539 | AP003119.3 | 1.781828736 |
| KLF12 | 1.95917353 | AP003900.1 | -1.559103248 |
| KLF7 | 1.1034462 | AP005137.2 | 2.361528009 |
| KLF9 | 2.055646918 | AP005212.2 | -5.87869148 |
| KLHDC7A | -1.317421318 | AP005212.4 | -4.437698045 |
| KLHDC9 | -1.404710513 | AP005233.2 | -7.971360508 |
| KLHL13 | 4.014026293 | AP005264.5 | -2.849882994 |
| KLK2 | -2.748246161 | AP006545.1 | -1.687780248 |
| KLRA1P | 1.286663489 | AP006621.4 | -4.417099791 |
| KREMEN2 | -1.565702856 | AP1M2 | -4.336711499 |
| KRT16 | -4.358316743 | AP1S1 | -1.16506672 |
| KRT19 | -9.272515389 | AP5M1 | 1.168529843 |
| KRT6A | -1.234355361 | APBA1 | 3.305845101 |
| KRT84 | -6.741205612 | APBB1IP | -2.047185247 |
| KRT8P26 | 2.746078959 | APCDD1 | 8.317145706 |
| KRTAP5-1 | -2.701858125 | APOBEC3B | 1.436967093 |
| KRTAP5-AS1 | -2.724651617 | APOE | 3.514654553 |
| KSR2 | -1.449114701 | APOH | -3.226875209 |
| KYNU | 1.17936953 | APOLD1 | 1.555564437 |
| L1CAM | 1.29532717 | AQP1 | -1.309181542 |
| LAMA1 | 5.905721454 | AQP2 | 7.01163952 |
| LAMA4 | 6.179455355 | AQP6 | 2.347282655 |
| LAMA5-AS1 | -1.496858259 | AQP7 | -2.869178149 |
| LAMC3 | -1.546196232 | AQP9 | 3.561604508 |
| LAMP2 | 1.245449208 | AREG | -6.609051998 |
| LAMP3 | -2.43044623 | ARHGAP11A | 1.031046477 |
| LAMTOR5-AS1 | -1.252577377 | ARHGAP12 | -1.06091093 |
| LAPTM4A | 1.099533996 | ARHGAP18 | 1.308660112 |
| LAT2 | -1.246048654 | ARHGAP19 | 1.023644076 |
| LCP1 | 2.964440303 | ARHGAP20 | 2.831528762 |
| LDLR | 1.321328559 | ARHGAP25 | 1.34142222 |
| LENG9 | -1.277148153 | ARHGAP26 | -3.018510006 |
| LGALS3 | 1.008501935 | ARHGAP28 | 2.022769443 |
| LGALS9 | 1.782379912 | ARHGAP29 | -1.406772141 |
| LGI4 | -2.821995137 | ARHGAP30 | -2.038224837 |
| LGR6 | -3.686493552 | ARHGAP33 | 2.099091584 |
| LHFPL2 | 1.048271165 | ARHGAP36 | 7.601633588 |
| LHFPL6 | 1.616030323 | ARHGAP39 | -1.451004427 |
| LIFR | 1.841448574 | ARHGAP4 | -1.664945313 |
| LIMA1 | 2.856637057 | ARHGAP44 | -2.191615646 |
| LIMD2 | -1.391322181 | ARHGDIG | -1.362827237 |
| LINC00304 | -1.410214565 | ARHGEF18 | -1.045576954 |
| LINC00452 | 3.182794119 | ARHGEF19 | -1.201819035 |
| LINC00488 | 2.002310202 | ARHGEF28 | -1.703577712 |
| LINC00511 | -1.353844073 | ARHGEF40 | 1.663227747 |
| LINC00536 | 6.893783955 | ARHGEF5 | -3.786349739 |
| LINC00547 | 3.907875834 | ARHGEF6 | 4.2483574 |
| LINC00565 | 3.543264111 | ARID5A | -1.778883525 |
| LINC00601 | 3.226578493 | ARL14 | -4.210140758 |
| LINC00607 | 2.903706924 | ARL16 | -1.132307776 |
| LINC00632 | -1.822293518 | ARL4C | -1.609249564 |
| LINC00960 | -1.495472303 | ARL4D | -1.338999684 |
| LINC01085 | 2.331139122 | ARL6IP1 | 1.928576964 |
| LINC01116 | 2.080954142 | ARLNC1 | 2.739431519 |
| LINC01119 | 2.98102708 | ARMC12 | -1.728360491 |
| LINC01126 | -1.406997808 | ARMC3 | -1.332740005 |
| LINC01145 | 1.129660955 | ARMC4 | 3.214197459 |
| LINC01173 | 3.859249634 | ARMC9 | -1.181211621 |
| LINC01322 | 2.665472421 | ARMCX3 | 1.717483123 |
| LINC01433 | 1.284396683 | ARMCX4 | 3.119767579 |
| LINC01583 | -1.25833615 | ARMH4 | 5.122525413 |
| LINC01693 | 3.618826127 | ARNTL2 | -1.348921151 |
| LINC01807 | 6.321516108 | ARRDC4 | -2.469924693 |
| LINC01881 | 1.146542426 | ARSB | 1.669475079 |
| LINC01970 | -2.158976088 | ARSE | -2.132422063 |
| LINC02086 | -1.682071271 | ARSF | 4.99128805 |
| LINC02274 | 4.448333386 | ARSK | 1.185171429 |
| LINC02476 | 1.679138873 | ARVCF | -2.15968371 |
| LINC02532 | 1.572695742 | ARX | -1.114147482 |
| LINC02593 | 1.071088382 | ASAH1 | 1.148496024 |
| LINC-PINT | 1.104708652 | ASB1 | -1.436038469 |
| LITAF | 1.054632347 | ASB9 | -1.000586715 |
| LKAAEAR1 | -1.5044706 | ASIC2 | -1.183771848 |
| LMCD1 | -2.461704143 | ASIC5 | 2.811732071 |
| LMCD1-AS1 | -2.045353099 | ASIP | -3.057707149 |
| LMNTD1 | 4.812777457 | ASMTL-AS1 | -1.037500558 |
| LMTK3 | -1.044022553 | ASPA | 5.328160799 |
| LOXL1 | 2.251495273 | ASPDH | -2.423768065 |
| LOXL2 | 1.347495878 | ASPH | 1.120029967 |
| LOXL3 | 1.017835915 | ASPHD1 | -1.93950915 |
| LPAR6 | 1.180436182 | ASPM | 1.66685793 |
| LPCAT2 | 1.963957098 | ASS1 | 1.889179321 |
| LRCH2 | 5.556376434 | ASS1P12 | -2.563612349 |
| LRP1B | 5.27915514 | ASTN1 | 6.53622217 |
| LRRC10B | -4.794564534 | ASXL3 | 4.854104317 |
| LRRC4B | -1.086005953 | ATF7IP2 | 1.213400302 |
| LRRC75A | -1.333012406 | ATG101 | -1.6960181 |
| LSP1 | 2.526050385 | ATG9B | -4.805429746 |
| LTBP2 | 1.009782216 | ATL1 | 1.222044318 |
| LTBP4 | -1.555979655 | ATOH8 | 1.60127553 |
| LUM | 3.684015871 | ATP10A | -8.878890477 |
| LXN | 1.162495518 | ATP10D | 1.169114477 |
| LY6K | 1.395217491 | ATP13A5 | 4.725790052 |
| LYPD1 | -3.381466333 | ATP1A2 | -2.982004344 |
| LYPD6 | 2.230859049 | ATP1B2 | 2.575403932 |
| LYPD6B | 6.743777172 | ATP2A1-AS1 | -1.253382708 |
| LYPD8 | -2.711058112 | ATP2A2 | -1.242356193 |
| MAFB | 3.004340542 | ATP2B1 | -1.360393173 |
| MAGED1 | 1.352608137 | ATP2B1-AS1 | -1.063149591 |
| MAMDC2 | 5.801028805 | ATP2B2 | 6.406792447 |
| MAML2 | 1.750626779 | ATP2C2 | -2.508778505 |
| MAN1A1 | 5.260031331 | ATP2C2-AS1 | -2.789117488 |
| MANEA | 1.214899268 | ATP5MC3 | 1.360813591 |
| MAP2 | 2.45688982 | ATP5PF | 1.218994121 |
| MAP3K15 | -1.134766735 | ATP6V0A4 | 2.706435521 |
| MAP7D2 | -2.201455865 | ATP6V0E2-AS1 | -1.317650962 |
| MAPK15 | -1.092212099 | ATP6V1A | 1.080536186 |
| MAPK4 | 6.789109007 | ATP6V1G2 | 1.475364303 |
| MARCOL | 5.73925662 | ATP8A1 | 3.315310268 |
| MARK1 | 1.056643944 | ATP8A2 | 4.760538705 |
| MASP1 | 1.955979493 | ATP8B1 | -3.259313069 |
| MBL1P | 4.802743126 | ATP8B5P | -1.959099781 |
| MBNL3 | 5.75412753 | ATRAID | 1.407211269 |
| MCC | 3.384735044 | ATRNL1 | 3.011778757 |
| MCF2L | -2.001167152 | AURKA | 1.115539113 |
| MDGA1 | 2.208281075 | AURKC | -2.715322992 |
| ME3 | -1.452313166 | AXIN1 | -1.296368772 |
| MECOM | 3.798971963 | AZIN2 | 2.229734381 |
| MEDAG | 5.689616302 | B2M | 1.849223345 |
| MEF2C | 5.69475396 | B3GALT5 | -8.252735636 |
| MEG3 | -1.151075749 | B3GAT1 | 3.297168641 |
| MEG9 | -1.442226621 | B3GNT3 | -1.340787582 |
| MEGF11 | -1.525581974 | B4GALNT2 | -2.65544136 |
| MEIS3P2 | -1.673995912 | BAAT | -2.902476736 |
| MESP1 | -1.275567992 | BACE2 | 1.102398806 |
| METTL7A | 3.619223745 | BACH2 | 2.236983996 |
| MGAT2 | -1.592761238 | BAHCC1 | -1.583278024 |
| MGST3 | 1.145551048 | BAIAP2L1 | -1.057706188 |
| MICAL2 | -1.189578391 | BAIAP2L2 | -2.001430385 |
| MIF | -2.121856818 | BAIAP3 | -1.075672611 |
| MILR1 | 1.607610351 | BARX1 | -1.121229855 |
| MIR4697HG | -1.550230694 | BASP1-AS1 | 1.239218678 |
| MIR99AHG | 1.129866949 | BATF2 | -1.529135366 |
| MISP3 | -1.425022862 | BATF3 | -1.3509614 |
| MKX | 3.433243881 | BBC3 | -1.086275555 |
| MLXIPL | -2.213993706 | BBOX1 | 4.999199946 |
| MME | 9.489756579 | BCAM | 3.578763588 |
| MME-AS1 | 4.647441888 | BCAS4 | -2.346634888 |
| MMP15 | -1.063587837 | BCHE | 3.870095567 |
| MMP16 | 1.977857456 | BCL11A | 4.053292803 |
| MMP17 | -2.27457688 | BCL2 | 1.031394739 |
| MMP2 | 5.004666207 | BCL2A1 | -4.751626514 |
| MMP7 | -2.521826878 | BCL2L1 | -3.011052168 |
| MN1 | -1.246477324 | BCL2L10 | 1.615852493 |
| MNX1 | -1.285773627 | BCL2L11 | 1.898694201 |
| MNX1-AS1 | -1.237462061 | BCL3 | 1.63535199 |
| MORN3 | -1.834444402 | BCL6 | 2.009138364 |
| MOXD1 | 1.130376491 | BCL9 | 1.423181549 |
| MPP1 | 1.354732989 | BCO1 | -4.145750328 |
| MPP2 | -1.138100363 | BCO2 | 1.505873309 |
| MPP7 | 2.206430908 | BDH2 | 1.29517278 |
| MRPL12 | -1.072679198 | BDKRB1 | 1.170829146 |
| MSC-AS1 | 1.166375789 | BDKRB2 | 2.014949066 |
| MSX1 | 1.048297137 | BEGAIN | 1.617896546 |
| MTATP6P1 | 1.03765864 | BEST3 | -2.828508287 |
| MTCL1 | -1.07098455 | BEX2 | -2.154688601 |
| MTMR9LP | -1.360310476 | BEX4 | 1.300383293 |
| MT-ND5 | 1.101076521 | BHLHB9 | 1.112665509 |
| MTSS1 | -1.260969942 | BHLHE22 | 5.707030288 |
| MT-TF | -2.515335337 | BHLHE41 | 3.205699457 |
| MT-TT | 1.213082315 | BICC1 | -1.769948955 |
| MTUS2 | 2.940847837 | BICDL1 | -1.372915999 |
| MUC13 | 1.737636674 | BIRC2 | -1.857812392 |
| MUC19 | -5.017028962 | BIRC3 | -4.869454618 |
| MUC5AC | -1.158199698 | BIRC5 | 1.125796434 |
| MYCL | -1.372798166 | BIRC7 | -2.145567778 |
| MYEOV | -1.628221409 | BMF | 2.131097194 |
| MYOSLID | 3.541099349 | BMP2 | 2.553081496 |
| MYPN | 2.139785413 | BMP3 | 4.60375243 |
| N4BP2L1 | 1.428441952 | BMP5 | 3.009791234 |
| N4BP3 | -1.866793491 | BMP6 | -1.827576172 |
| NAALAD2 | 1.056248617 | BMP7 | 1.362305102 |
| NAALADL2 | 1.946154761 | BMPER | 1.527566217 |
| NALCN | -1.222699643 | BMS1P2 | -1.406440825 |
| NALT1 | -2.737605133 | BMS1P21 | 4.062020153 |
| NAMPT | 1.398156156 | BMS1P4 | 1.259015915 |
| NAMPTP1 | 1.49521394 | BNC1 | -2.440263416 |
| NAV1 | 1.521465394 | BOLA2P2 | 2.811755357 |
| NBPF4 | 2.239449987 | BRINP1 | 5.273292169 |
| NCAM1 | 3.703413888 | BRINP2 | 6.025289606 |
| NDNF | 5.143513672 | BRINP3 | 3.806347542 |
| NEB | 1.385877809 | BSPH1 | 4.876804818 |
| NEGR1 | 8.162000212 | BST1 | -1.443231942 |
| NELL2 | 1.507594358 | BST2 | 6.317789508 |
| NFKBIE | -1.052811601 | BTBD11 | -3.815585653 |
| NGEF | -1.499963339 | BTBD3 | 1.389801286 |
| NGF | -2.354535584 | BTN3A2 | 1.316684445 |
| NID2 | 2.835792102 | BTN3A3 | 2.13468415 |
| NIPAL4 | -1.748382622 | BUB1 | 1.130788204 |
| NKAIN1 | -1.586190301 | BUB1B | 1.015824015 |
| NKAIN4 | -1.768016364 | BX005266.2 | -2.22819754 |
| NKD1 | -2.27647807 | BX088651.4 | -2.136917227 |
| NKPD1 | -1.319891987 | BX284668.2 | -1.289540749 |
| NKX2-8 | -1.170138688 | BX322234.1 | -5.269042036 |
| NLGN1 | 3.156783193 | BX322234.2 | -5.515882464 |
| NLGN4Y | 2.433865203 | BX470102.2 | -2.245918073 |
| NME2 | -1.331250471 | BX537318.1 | -1.579995227 |
| NMNAT2 | 2.072893106 | BX842568.4 | 4.703209561 |
| NMRAL2P | 1.224955078 | C10orf90 | 1.601562983 |
| NOG | -1.86321925 | C11orf24 | -1.087176234 |
| NOS1 | -2.454436118 | C11orf44 | -4.791267144 |
| NOSTRIN | 1.050067476 | C11orf45 | -3.105734244 |
| NOTCH1 | -1.057946572 | C11orf68 | -1.29313256 |
| NOTCH3 | 2.812145093 | C11orf71 | 1.483854209 |
| NOV | 1.091410534 | C11orf86 | -1.168545188 |
| NOVA2 | -1.964179291 | C11orf87 | 3.700805313 |
| NOX5 | 2.167508793 | C11orf91 | -3.523741932 |
| NOXA1 | -1.885721818 | C11orf96 | 3.643455885 |
| NPAS1 | -1.248102923 | C12orf45 | -1.248565315 |
| NPAS3 | 3.692905092 | C12orf75 | -1.175604686 |
| NPBWR1 | 2.746364434 | C13orf46 | 2.293451836 |
| NPC2 | 1.209250877 | C14orf132 | 1.3932098 |
| NPL | 1.375687431 | C15orf48 | 3.642229534 |
| NPNT | 3.269136996 | C16orf45 | -2.364649644 |
| NPR2 | -1.027947938 | C16orf71 | -1.441694914 |
| NPTX1 | 3.972461544 | C16orf74 | -2.193889356 |
| NPY1R | 2.380078357 | C16orf89 | 2.413626845 |
| NPY4R | 2.401080379 | C17orf102 | -5.349529491 |
| NPY4R2 | 2.626953283 | C19orf33 | -3.420547112 |
| NQO1 | 1.018439783 | C19orf38 | -3.406566954 |
| NR2E3 | -2.094903886 | C1GALT1 | -1.294758116 |
| NR2F1 | -1.603220051 | C1GALT1C1 | 2.093504789 |
| NR2F1-AS1 | -1.435608465 | C1GALT1C1L | 1.412662546 |
| NR5A2 | -1.656960741 | C1orf109 | -1.035705902 |
| NRARP | -1.667241 | C1orf116 | -6.618192446 |
| NRG1 | -1.099880927 | C1orf162 | 1.659439511 |
| NRG4 | 1.563507861 | C1orf194 | -2.124840069 |
| NRTN | -1.896367884 | C1orf21 | 2.61627805 |
| NRXN2 | -2.014471859 | C1orf35 | -1.020610911 |
| NRXN3 | -2.013247855 | C1orf54 | 3.136311697 |
| NSG1 | -2.458276371 | C1orf61 | -1.814978784 |
| NT5E | 1.184127819 | C1orf74 | -1.294037862 |
| NTHL1 | -1.010789483 | C1QL4 | -2.625919508 |
| NTN1 | -2.892069296 | C1QTNF6 | 3.244401554 |
| NTN3 | -2.0639363 | C1R | 3.806569572 |
| NTRK2 | 3.487580322 | C1S | 3.804563902 |
| NTRK3 | -1.966140059 | C20orf141 | 4.815868079 |
| NTS | 3.64880049 | C20orf96 | 1.02265822 |
| NTSR1 | -2.542494835 | C21orf58 | 1.010796604 |
| NUDT16P1 | -5.798148946 | C21orf91 | 1.299188796 |
| NUP62CL | -1.409016815 | C22orf23 | -1.954563512 |
| NXNL2 | -1.845578614 | C2CD4D | 4.173500667 |
| OAS1 | 1.108858254 | C2orf27A | 1.366887107 |
| OASL | 1.908448796 | C2orf76 | 1.070620499 |
| OBSCN-AS1 | 1.121826534 | C3 | 2.073594525 |
| OCLN | -1.578421807 | C3AR1 | 3.904531481 |
| OLFML2A | 1.627938598 | C3orf20 | -2.12908817 |
| OPN1SW | 1.226166934 | C3orf52 | -1.465704473 |
| OSR1 | 1.14457335 | C3orf58 | 2.099125783 |
| OSTM1 | 1.047091751 | C3orf70 | 4.446014948 |
| OTULINL | -1.577505781 | C3orf80 | 1.575887231 |
| OVCH1 | 3.636827637 | C4B | 2.577671485 |
| OXCT1 | 1.849091576 | C4orf19 | 4.350833675 |
| OXTR | -1.199996348 | C4orf54 | 4.550960089 |
| P3H3 | -1.403672484 | C5 | -2.284170414 |
| P4HA3 | 1.939396092 | C5AR1 | 4.143255602 |
| PAG1 | 4.221900967 | C5AR2 | 5.067430786 |
| PAK3 | 2.443804073 | C6orf132 | -7.081453824 |
| PALD1 | -1.433621271 | C6orf163 | 1.505762815 |
| PALMD | 5.420726688 | C6orf48 | -1.927546886 |
| PAM | 1.477740673 | C6orf99 | -1.900235841 |
| PANX2 | 1.403544526 | C8orf31 | 4.355783403 |
| PAPPA2 | 1.721942295 | C8orf58 | -1.185364361 |
| PAPSS2 | 2.09413401 | C8orf88 | 1.616454507 |
| PARD6A | -1.654833431 | CA11 | 1.981321482 |
| PARM1 | -1.72766139 | CA13 | 1.024415868 |
| PAX8-AS1 | 1.234785298 | CA2 | 4.161229656 |
| PCBP3 | 2.627653545 | CA3-AS1 | 3.569621194 |
| PCDH1 | -1.412267381 | CA8 | 1.745931137 |
| PCDH10 | 3.003327771 | CA9 | 2.66658789 |
| PCDH15 | -2.756196075 | CABYR | 1.475544298 |
| PCDH18 | 6.660301275 | CACNA1B | -5.358588695 |
| PCDH7 | 4.066084924 | CACNA1C | 2.546612486 |
| PCDHA11 | -1.437548644 | CACNA1D | -1.208384051 |
| PCDHB8 | -1.906157636 | CACNA1H | -2.050425903 |
| PCDHGB4 | 1.217263188 | CACNA2D3 | 6.790800648 |
| PCSK1 | 1.8103628 | CACNB1 | -1.545256504 |
| PCSK5 | 1.333363263 | CACNB2 | 2.740439233 |
| PDCD1LG2 | 1.880148623 | CACNB3 | -1.628686312 |
| PDE10A | 1.262426687 | CACNG4 | -1.05568438 |
| PDE1A | 5.671082276 | CACNG6 | -7.899411113 |
| PDE3A | 1.117796677 | CACNG7 | -4.697544682 |
| PDE3B | 1.75549738 | CACNG8 | -4.884709045 |
| PDE9A | -1.192648303 | CADM2 | 2.212261446 |
| PDGFB | -3.065393922 | CADM3 | 6.373087854 |
| PDGFRB | 2.927882801 | CADM3-AS1 | 3.91625688 |
| PDGFRL | -1.521984689 | CADPS2 | 2.408831732 |
| PDLIM1 | -1.111785295 | CALB1 | 3.278531009 |
| PDZK1 | 1.043903901 | CALB2 | 1.460597063 |
| PELI2 | 1.23090302 | CALCB | 2.740964034 |
| PER1 | -1.202081704 | CALCR | 4.678506552 |
| PER2 | -1.492737034 | CALCRL | 1.274076575 |
| PET117 | -1.06152712 | CALHM5 | 4.004450881 |
| PEX11G | -1.065664896 | CALM3 | 1.046905815 |
| PGBD5 | 6.174526788 | CALR | 1.537334708 |
| PGM5P2 | -1.555576748 | CAMK1D | -1.542357494 |
| PGR | 2.383822327 | CAMK2B | -3.531837847 |
| PGRMC1 | 1.432480571 | CAMK2N1 | -1.106022418 |
| PHLDB3 | -1.164817791 | CAMK4 | 4.34696789 |
| PHOSPHO1 | -1.438297157 | CAMKV | -2.717115762 |
| PI15 | 1.649234555 | CAPN12 | -1.281692208 |
| PICALM | 1.1564751 | CAPN13 | -1.823294333 |
| PID1 | 1.793562577 | CAPN5 | -2.480614868 |
| PIEZO2 | 3.230377221 | CAPN6 | 8.227565368 |
| PIP5K1B | 6.207727958 | CARD10 | -2.408341803 |
| PITPNC1 | 2.505015993 | CARD16 | 5.541657395 |
| PKDCC | 1.96118716 | CARD6 | 1.148335546 |
| PKIA | 2.793390932 | CARD8 | 1.374827537 |
| PLA2G16 | -1.067972093 | CARD8-AS1 | 1.60994921 |
| PLA2R1 | 1.506921566 | CARD9 | -1.321415024 |
| PLAG1 | 2.937429954 | CARF | 1.221079953 |
| PLAU | 1.665373645 | CARMN | 3.470231323 |
| PLBD1 | 1.298148871 | CARNS1 | -1.996941805 |
| PLCB1 | 2.728466086 | CARS | -1.241935928 |
| PLCH1 | -1.387846672 | CASC10 | -1.239058243 |
| PLCH2 | -1.726806113 | CASC19 | -5.876738811 |
| PLCL1 | 2.543393218 | CASC8 | -6.773431874 |
| PLEKHA2 | 1.759712297 | CASP1 | 5.562801027 |
| PLEKHA7 | -1.26017697 | CASP10 | -2.1811783 |
| PLEKHG6 | 1.865143551 | CASP4 | 1.565760057 |
| PLEKHM3 | 1.110482564 | CASZ1 | -1.09843274 |
| PLGLB1 | 1.693989377 | CAT | 1.251954633 |
| PLS1 | -1.320759295 | CATSPER1 | -3.380593356 |
| PLSCR4 | 1.410546655 | CATSPERB | 4.089836194 |
| PLXDC2 | 7.326085664 | CATSPERG | -2.282749422 |
| PODNL1 | 1.640479408 | CAV1 | -2.450287169 |
| POPDC3 | 1.852163488 | CAV2 | -2.537056262 |
| POSTN | 9.533117332 | CAVIN1 | -1.535233288 |
| POU4F1 | -1.265231157 | CBL | -1.055046379 |
| PPARA | 1.1047578 | CBLN2 | 2.059420865 |
| PPFIBP2 | -1.02489336 | CBLN3 | 1.713368327 |
| PPL | -1.055969178 | CBR3 | 1.419906623 |
| PPP1R14A | -1.720758956 | CBS | -1.556841385 |
| PPP1R14C | -1.494911727 | CCBE1 | 1.510338247 |
| PPP1R16B | -2.009000876 | CCDC102B | 3.328577771 |
| PPP1R1C | 1.289951018 | CCDC110 | 6.186042155 |
| PPP1R3C | 1.079138948 | CCDC113 | -1.586630796 |
| PRAG1 | -1.491885756 | CCDC114 | -1.050803221 |
| PRDM1 | 1.108142801 | CCDC121 | 1.318984993 |
| PRICKLE1 | -1.878552485 | CCDC137 | -1.367783283 |
| PRKAG2-AS1 | -1.95516639 | CCDC14 | 1.081020505 |
| PRKCG | -1.894607175 | CCDC146 | -1.672372648 |
| PRKCZ | -1.046411419 | CCDC148 | -2.582477764 |
| PRKG1 | 2.414291019 | CCDC15 | -1.369389006 |
| PRKG1-AS1 | -1.304256429 | CCDC173 | -1.818931852 |
| PRODH2 | 2.887489135 | CCDC177 | 3.63692896 |
| PRR16 | 3.486902469 | CCDC178 | 2.762662408 |
| PRR26 | 2.214098453 | CCDC180 | -3.25453298 |
| PRR29 | -1.801111523 | CCDC183-AS1 | -1.027455238 |
| PRR36 | -1.918089568 | CCDC184 | -1.902834093 |
| PRR5L | 3.213202157 | CCDC189 | -2.780865615 |
| PRRT4 | -2.151380894 | CCDC191 | -1.26314004 |
| PRRX2 | 1.335625997 | CCDC198 | -6.936130348 |
| PRSS12 | 1.45013911 | CCDC27 | -3.528488803 |
| PRSS3 | -4.748701477 | CCDC33 | -1.922514974 |
| PRUNE2 | 4.521273268 | CCDC40 | -1.547397293 |
| PSD2 | 3.537353763 | CCDC54 | 3.224413329 |
| PTCHD4 | 2.693319335 | CCDC69 | 1.176854726 |
| PTGER4 | -1.151644073 | CCDC74B | -1.008434254 |
| PTGFR | 5.584823383 | CCDC80 | -1.368348541 |
| PTPDC1 | -1.13857315 | CCDC85B | -2.070913944 |
| PTPN6 | -1.28388496 | CCDC86 | -1.382650926 |
| PTPRD | -1.543664621 | CCDC88B | -1.274141335 |
| PTPRE | 1.298194777 | CCDC92B | -2.1573646 |
| PTPRN2 | -1.364570639 | CCDC96 | -1.56534194 |
| PTPRU | -1.025499247 | CCL20 | -2.476928928 |
| PWAR5 | -1.056161487 | CCL26 | -1.883941712 |
| PXDN | -5.29640856 | CCNA2 | 1.547947695 |
| PXK | 1.803046369 | CCNB1 | 1.679343579 |
| PYCARD | -5.738494008 | CCNB1IP1 | -1.021298601 |
| QPRT | 1.433597693 | CCNB2 | 2.068212025 |
| QRFPR | 2.182603971 | CCND1 | -1.620852162 |
| RAB11FIP4 | -1.319184814 | CCND2 | 7.13367194 |
| RAB20 | 1.980620745 | CCND2-AS1 | 2.119794918 |
| RAB24 | -1.073945898 | CCNF | 1.758105591 |
| RAB26 | -1.545569604 | CCNG1 | 1.001758696 |
| RAB27A | 1.441840475 | CCNG2 | 1.393098234 |
| RAB27B | 1.048805466 | CCNJL | -1.420307742 |
| RAB31 | 1.599069186 | CCNO | -1.606659357 |
| RAB3C | 5.251808681 | CCR1 | 6.500461228 |
| RAB7B | 2.054330394 | CCR10 | -2.078449297 |
| RADIL | -1.255914693 | CCR3 | 5.094874268 |
| RAET1G | 1.987714033 | CCSER1 | 2.727583458 |
| RAP2B | -1.006879886 | CD101 | 1.753354335 |
| RAPGEF5 | -1.538813931 | CD109 | -1.40913442 |
| RARB | 2.490888168 | CD14 | -2.087909762 |
| RASA3 | 1.331881261 | CD163L1 | -1.746659039 |
| RASEF | -1.353142944 | CD164 | 1.194242805 |
| RASSF10 | -1.139051652 | CD1D | 3.272591958 |
| RASSF2 | -1.521574018 | CD200 | 5.70893943 |
| RASSF6 | 1.633102262 | CD22 | -8.550886192 |
| RASSF7 | -1.022411871 | CD226 | -2.463091204 |
| RASSF8 | 2.245994023 | CD276 | 1.188275976 |
| RASSF8-AS1 | 1.562926451 | CD300C | 3.254127734 |
| RBFOX3 | -2.1206324 | CD302 | 1.955714389 |
| RBM11 | 5.253597027 | CD320 | 1.02212106 |
| RBMS3 | 1.08430698 | CD36 | 2.602633734 |
| RCOR2 | -2.766017162 | CD38 | -2.159239938 |
| REEP1 | 1.148278778 | CD44 | -1.769782339 |
| REEP2 | -1.916358201 | CD44-AS1 | -2.292484309 |
| RERG | 3.058667342 | CD55 | 2.25904121 |
| RETREG1 | 1.036741903 | CD70 | -1.635816917 |
| RFTN1 | -1.339939096 | CD74 | 6.995323795 |
| RFXAP | 1.033534261 | CD82 | 1.318167496 |
| RGL1 | 2.421746726 | CD86 | 4.626591719 |
| RGMA | -3.747342011 | CD8A | -3.22081636 |
| RGS4 | 3.984858274 | CD9 | -1.027949449 |
| RHCG | -1.937629486 | CD99L2 | 1.324635833 |
| RHOB | 1.874959008 | CDA | -3.205455409 |
| RHOD | -4.08445914 | CDC25B | 1.006602703 |
| RHOV | 1.810848249 | CDC25C | 1.484464238 |
| RHPN1 | -1.389187622 | CDC42BPG | -2.609546138 |
| RILP | -1.202831539 | CDC42EP1 | -1.586256323 |
| RINL | -1.957177445 | CDC42EP3 | -1.04094647 |
| RIPK4 | -1.48938694 | CDC42EP5 | 3.605963469 |
| RN7SKP239 | 3.413481555 | CDC42P3 | 3.502459878 |
| RN7SL2 | -3.098394013 | CDCA2 | 1.040753106 |
| RN7SL417P | 2.80667411 | CDCA3 | 1.798704061 |
| RNF128 | 4.609949649 | CDCP1 | -3.029916527 |
| RNF165 | -1.136622933 | CDH1 | -2.916184624 |
| RNF182 | 4.105680685 | CDH11 | 6.648114262 |
| RNF19A | 1.340111258 | CDH17 | -6.566799249 |
| RNF212 | -2.26233617 | CDH19 | 1.978158321 |
| RNF24 | 1.345362348 | CDH2 | 1.062876848 |
| ROBO3 | -1.12595475 | CDH6 | 3.60354415 |
| RORA | 4.137001756 | CDH7 | 5.636402121 |
| RORB | 7.084658159 | CDH8 | 5.497587652 |
| ROS1 | 1.251046022 | CDHR5 | -1.046312038 |
| RPH3AL | -1.213331064 | CDK1 | 1.253548974 |
| RPRM | 4.050487181 | CDK14 | 2.282063035 |
| RSPH1 | -1.679100439 | CDK18 | 2.485962851 |
| RSPO2 | 2.817937098 | CDK5R1 | -1.029543151 |
| RSPO3 | 1.170603515 | CDK5R2 | 1.799424633 |
| RTN1 | 7.718170295 | CDKL1 | -1.420747505 |
| RTN4RL1 | -2.179768823 | CDKL5 | -1.133773494 |
| RTN4RL2 | 1.006200806 | CDKN1C | 1.135789671 |
| RUNDC3A | -1.28758877 | CDKN2C | 2.218657091 |
| RXFP2 | 8.846978284 | CDKN3 | 2.415979367 |
| S100A9 | 1.490178867 | CDR2L | -2.296323384 |
| S1PR1 | 2.781050234 | CDS1 | 1.383828245 |
| SAGE1 | -6.853654246 | CDX2 | 2.310191439 |
| SAMD11 | 1.416646281 | CDYL2 | 3.903205128 |
| SAMD5 | 1.764679672 | CEACAM5 | 3.87330674 |
| SAMD9L | 2.324481289 | CEACAM6 | -3.859837339 |
| SATB1 | 3.392075771 | CEACAM7 | 7.127474917 |
| SBK1 | -1.563505496 | CEBPD | 1.483781587 |
| SBK2 | -1.894452539 | CEBPG | -1.033381877 |
| SC5D | 1.017411194 | CECR2 | 2.014285743 |
| SCD | 1.613728437 | CELF2 | 7.072444213 |
| SCG2 | 7.605396279 | CELF2-AS1 | 4.348796844 |
| SCN1B | -1.252832727 | CELF5 | -1.669652384 |
| SCN2A | 2.079856478 | CEMIP | 1.936593546 |
| SCN4A | -1.902641153 | CEMIP2 | 1.104073724 |
| SCN5A | -2.563084844 | CENPA | 1.7332352 |
| SDC2 | 1.822182443 | CENPE | 1.504408287 |
| SDK1 | 1.731301742 | CENPF | 2.012694493 |
| SDK2 | -1.723825965 | CENPV | -1.364908467 |
| SEC24D | 1.197534559 | CENPVL3 | 2.616728731 |
| SELL | 4.187408464 | CEP126 | -1.225153555 |
| SEMA3E | 2.207002801 | CEP164 | -1.096383357 |
| SEMA6D | 3.046336654 | CEP170 | -1.219483465 |
| SESN3 | 1.703183831 | CEP290 | -1.107290194 |
| SESTD1 | 1.019920988 | CEP70 | 1.130834432 |
| SEZ6 | -2.52829819 | CEP85 | 1.002268705 |
| SFRP1 | 1.539942156 | CERKL | 2.407312311 |
| SGK1 | 1.714066904 | CERS1 | -2.816173449 |
| SGSM3 | -1.434030136 | CES1 | 10.83259504 |
| SH2B3 | 3.005010059 | CES3 | 1.11181841 |
| SH2D5 | -1.272511941 | CFAP300 | -1.271248329 |
| SH3BGRL | 1.153542812 | CFAP53 | -1.574789546 |
| SH3BP5 | 1.425136747 | CFAP57 | -1.599637486 |
| SH3GL2 | 4.436237509 | CFAP58-DT | 4.787991303 |
| SH3RF3 | -1.21155281 | CFAP61 | -1.813428925 |
| SH3RF3-AS1 | -2.819775564 | CFAP65 | -2.529213994 |
| SH3TC1 | -1.33522198 | CFAP69 | -1.93022708 |
| SH3TC2 | 1.358759891 | CFC1 | 3.78555005 |
| SHC4 | 3.834522108 | CFD | 1.687887235 |
| SHISAL1 | 3.077058679 | CFH | 2.418775651 |
| SIGLEC10 | -1.291312655 | CFI | 6.074105342 |
| SIK2 | 1.507350848 | CFLAR | -1.10654693 |
| SKIDA1 | 1.177158037 | CGA | 12.23058584 |
| SLC12A8 | 1.997711794 | CGAS | -6.999751423 |
| SLC16A11 | -1.811744351 | CGB5 | -3.88563234 |
| SLC16A14 | -2.016140162 | CGB7 | -3.049699471 |
| SLC16A6 | 2.221854306 | CGB8 | -8.183227285 |
| SLC17A1 | 3.516067138 | CH25H | 6.71917033 |
| SLC17A3 | 3.485544735 | CHAC1 | -1.73037366 |
| SLC22A15 | 1.017646776 | CHCHD7 | 1.782986526 |
| SLC22A3 | -1.203079936 | CHD3 | 1.189480199 |
| SLC22A31 | -1.247819378 | CHD5 | -3.023390348 |
| SLC23A1 | 1.098074962 | CHEK1 | -1.098534686 |
| SLC24A4 | -2.854073221 | CHFR | -1.195726776 |
| SLC25A10 | -1.13119287 | CHGB | 2.059802408 |
| SLC26A2 | 1.010306089 | CHL1 | 9.887583647 |
| SLC27A2 | -1.231227553 | CHL1-AS1 | 7.487277631 |
| SLC29A2 | -1.017745745 | CHL1-AS2 | 5.995346614 |
| SLC29A4 | -1.261884123 | CHMP1B | 1.738405896 |
| SLC30A1 | 1.401765817 | CHN1 | -1.017061851 |
| SLC30A3 | -1.9401061 | CHRD | 3.218641345 |
| SLC35F3 | -1.446532363 | CHRDL1 | 8.716295617 |
| SLC35G2 | 1.125955766 | CHRDL2 | 5.379737823 |
| SLC38A6 | 1.076087014 | CHRFAM7A | 1.153227653 |
| SLC40A1 | 1.399871985 | CHRM2 | 6.333327702 |
| SLC43A2 | -1.363698311 | CHRM3 | 2.089468062 |
| SLC44A1 | 1.486095799 | CHRNA3 | -1.276292886 |
| SLC45A1 | -1.832891907 | CHRNB2 | -2.166820147 |
| SLC4A7 | -1.042840624 | CHST11 | -1.481264694 |
| SLC51B | 1.207277036 | CHST2 | 2.34287321 |
| SLC52A1 | -2.517163184 | CHST4 | 6.25375147 |
| SLC6A15 | 1.130540979 | CHST6 | 1.154479496 |
| SLC7A7 | 2.83550292 | CHST7 | -1.530108724 |
| SLC7A8 | 4.375014903 | CHSY3 | 3.427646446 |
| SLC8A1 | -2.269344797 | CIB4 | 5.680706484 |
| SLC8A2 | -1.378109775 | CICP22 | -3.2331707 |
| SLC9A3R2 | -2.674443353 | CIITA | 2.811251219 |
| SLC9A9 | 1.389935655 | CILP | 3.237550265 |
| SLCO2B1 | 2.465839899 | CILP2 | -1.770336724 |
| SLCO4A1 | -1.524999351 | CISD3 | -1.020501234 |
| SLCO5A1 | -2.839119663 | CISH | 1.150854101 |
| SLFN11 | -6.072714742 | CKAP2 | 1.281142222 |
| SLFN13 | -1.315961786 | CKAP2L | 1.809668033 |
| SLIT1 | -1.62511805 | CKAP4 | 1.451503027 |
| SLIT2 | 5.578122314 | CKMT1A | 1.943872595 |
| SLIT3 | 1.258273928 | CKS2 | 1.549866695 |
| SMAD9 | 2.669488469 | CLBA1 | -1.226294551 |
| SMKR1 | -1.034692502 | CLCA2 | 2.891041648 |
| SMPX | 2.16616646 | CLDN1 | -3.457457391 |
| SNAI1 | 4.104883768 | CLDN16 | -2.770994705 |
| SNAI2 | 2.496963325 | CLDN2 | -1.770631401 |
| SNAP25-AS1 | 1.922792755 | CLDN3 | -2.406210895 |
| SNAP91 | 2.549630541 | CLDN4 | -1.850540966 |
| SNCA | 5.079836798 | CLDN9 | -3.45252451 |
| SNED1 | 1.965184035 | CLDND1 | 1.275278035 |
| SNHG28 | 1.243881644 | CLEC11A | 1.865284516 |
| SNX18P3 | -1.307958691 | CLEC19A | 2.990052981 |
| SNX29 | 1.011953384 | CLEC2B | 2.753133478 |
| SORBS2 | 2.6907569 | CLEC2D | 1.001457906 |
| SORT1 | -2.132862821 | CLIC2 | 4.028854887 |
| SOX18 | 1.197542254 | CLIC3 | 2.240752418 |
| SOX2 | -3.206485829 | CLIP1 | -1.083783293 |
| SOX4 | -1.149474363 | CLIP1-AS1 | -4.700374632 |
| SOX8 | -2.409734723 | CLIP2 | -1.103396066 |
| SPARC | 4.016328914 | CLIP4 | -1.099424518 |
| SPATA3-AS1 | -1.644723155 | CLMN | -2.003011069 |
| SPATA6L | -1.200826277 | CLMP | 1.105535531 |
| SPEF1 | -1.25087556 | CLSTN2 | 4.196146724 |
| SPEG | 3.166647072 | CLU | 2.383717043 |
| SPINK13 | 7.184035161 | CLVS2 | 1.956414254 |
| SPINK6 | 11.59966158 | CLYBL | 1.164515569 |
| SPINT2 | -1.428469455 | CMKLR1 | 9.417794348 |
| SPOCD1 | 2.966475059 | CMPK2 | 3.623400961 |
| SPON2 | -6.219065368 | CMTM8 | 1.317435315 |
| SPRY2 | -1.000099275 | CNDP2 | -1.343014849 |
| SPRY3 | 2.537088796 | CNKSR2 | -3.297571147 |
| SPTA1 | 4.041233282 | CNNM2 | 1.10777147 |
| SPTBN2 | -1.330774225 | CNR1 | 3.0598373 |
| SPTBN5 | -1.113641772 | CNRIP1 | 2.984663723 |
| SPTLC3 | 4.500738298 | CNTD2 | -1.714265624 |
| SPTSSA | 1.487982846 | CNTN1 | -4.203951275 |
| SRCIN1 | -1.215013532 | CNTN4 | 4.208085783 |
| SRGN | 1.177935583 | CNTN5 | 7.273602948 |
| SRPX | 2.452004666 | CNTN6 | 4.571144685 |
| SRPX2 | 1.209171834 | CNTNAP2 | 3.764077104 |
| SRRM3 | -1.01850441 | CNTNAP3B | -1.528645216 |
| SSBP3 | 1.169614083 | CNTNAP3P2 | -1.110921848 |
| SSPN | 2.233446637 | CNTNAP4 | 2.948085915 |
| ST5 | 1.137207281 | COBLL1 | 1.891069897 |
| ST6GAL2 | 1.102661992 | COL12A1 | 2.846578365 |
| ST6GALNAC3 | 1.543193677 | COL13A1 | -1.734914358 |
| ST7 | 1.017374059 | COL14A1 | 10.79238323 |
| ST8SIA4 | -1.682491481 | COL15A1 | 8.695251042 |
| STAC2 | 2.018808133 | COL17A1 | -3.238369447 |
| STARD4 | 2.798277435 | COL19A1 | 2.658936441 |
| STARD4-AS1 | 1.473715107 | COL1A1 | 1.858739894 |
| STARD8 | 1.300472127 | COL20A1 | -1.998210634 |
| STC1 | 2.083322327 | COL21A1 | -2.670476534 |
| STK17A | 1.062645303 | COL24A1 | 3.12401182 |
| STK32A | 2.612726827 | COL25A1 | 3.71041058 |
| STRC | -1.934014066 | COL3A1 | 8.61263567 |
| STXBP5L | 6.256230961 | COL4A1 | 2.903746514 |
| STYK1 | 1.389584763 | COL4A2 | 1.783282346 |
| SULF2 | -1.014414941 | COL4A5 | 1.034023322 |
| SULT1A1 | 2.073376224 | COL5A2 | 1.065051104 |
| SULT1C2 | 2.522026638 | COL5A3 | 5.483462844 |
| SUSD5 | 1.480177118 | COL6A1 | 2.325359317 |
| SV2C | 7.139137168 | COL6A2 | 2.320220117 |
| SYCE1L | -1.470555921 | COL6A6 | 3.803206074 |
| SYNC | 1.073421357 | COL8A1 | 11.8961113 |
| SYNE3 | 1.540089612 | COL9A3 | 5.119364928 |
| SYNGR3 | -1.722235245 | COLEC10 | 6.165964587 |
| SYPL2 | 1.541518172 | COLEC12 | 6.234915731 |
| SYT1 | -1.272450783 | COLGALT2 | 2.889344093 |
| SYT12 | -1.019493766 | COPS4 | 1.026468425 |
| SYT13 | -2.437979261 | COQ10A | -1.607244151 |
| SYT14 | 4.051317552 | CORO1C | -1.636156587 |
| SYT16 | 1.081325289 | CORO2B | -2.236105113 |
| SYTL2 | 1.37712438 | COX16 | 1.072196483 |
| SYTL5 | 4.099559248 | COX19 | -1.737200898 |
| TACR2 | 1.949903643 | COX6B2 | 1.713823854 |
| TAF10 | -1.259385399 | COX7B | 1.35185437 |
| TBC1D3P1 | -2.401477507 | COX7C | 1.202581528 |
| TBC1D8B | 1.50846713 | CP | 2.89051438 |
| TBILA | -1.062619695 | CPA4 | -1.168304003 |
| TBL1X | 1.080079207 | CPE | 2.462574129 |
| TBX2 | 2.417068185 | CPEB2 | -1.124114512 |
| TBX22 | 5.716173928 | CPEB3 | 1.08597574 |
| TBXA2R | 3.241471462 | CPEB4 | -1.368551478 |
| TCAF1P1 | -1.0441764 | CPED1 | 7.351300859 |
| TCEAL9 | 1.154850993 | CPLANE2 | -1.688923631 |
| TCHH | -2.611287551 | CPLX1 | 1.438707536 |
| TCIM | -1.13997634 | CPLX2 | 2.201818279 |
| TDO2 | 1.146756665 | CPM | -1.735218611 |
| TDP2 | 1.355116572 | CPNE4 | -1.924181075 |
| TEDC1 | -1.294832352 | CPNE7 | -2.629123748 |
| TENM1 | 2.474178084 | CPPED1 | -1.318118123 |
| TENM2 | 5.076482239 | CPQ | 3.419048198 |
| TENM4 | -4.959030228 | CPSF1P1 | -1.142844684 |
| TENT5A | 1.603951082 | CPSF4L | -4.00148004 |
| TENT5C | 2.031804724 | CPT2 | 1.094689962 |
| TERT | -1.887440431 | CPVL | 3.991322214 |
| TFCP2L1 | -1.829172754 | CR2 | 2.675527432 |
| TFEC | 3.218986514 | CRB3 | -4.035696 |
| TFPI | 1.826445493 | CRCP | -1.11303777 |
| TFPI2 | 1.200144611 | CREB3L1 | -2.409896943 |
| TGFB2-AS1 | -1.253929409 | CREG1 | 1.922677388 |
| TGFBI | 2.137331386 | CRELD1 | 1.33154848 |
| TGM2 | 1.461365056 | CRELD2 | 1.926687804 |
| THCAT158 | 4.771519442 | CREM | 1.439452702 |
| THRB | 1.006943428 | CRH | -4.890699035 |
| TIAM1 | -1.483707146 | CRIP1 | -1.522786472 |
| TIMP1 | 1.231358484 | CRISP3 | 4.429765007 |
| TIMP2 | 2.027283544 | CRISPLD1 | 5.148449488 |
| TINAGL1 | -1.397167974 | CRISPLD2 | 2.838069619 |
| TLCD2 | -1.035164734 | CRLF1 | 1.932410858 |
| TLE1 | -1.196623686 | CRLF3 | -1.018949662 |
| TLE4 | -1.350167713 | CRTAC1 | 1.132896627 |
| TLL1 | 3.078311958 | CRTAP | 1.254870135 |
| TM4SF1 | 1.187978802 | CRTC3 | 1.625995186 |
| TM4SF19 | 1.701060644 | CRYBG2 | -4.395722538 |
| TM4SF20 | 2.314622984 | CRYGN | 4.278949284 |
| TM4SF4 | 3.182144038 | CRYGS | 1.871628657 |
| TM7SF2 | -1.597722036 | CRYM-AS1 | -1.705908287 |
| TMC4 | -1.006407829 | CRYZ | -2.405722206 |
| TMC5 | -1.960317505 | CSF1 | 1.329060662 |
| TMC8 | -2.315602131 | CSF2RA | -2.758263462 |
| TMCC3 | 1.12468122 | CSMD1 | 2.542716638 |
| TMEM105 | -2.072967771 | CSMD3 | 5.597104031 |
| TMEM132D-AS1 | -2.591823074 | CSPG4 | -1.42544907 |
| TMEM155 | 3.765816784 | CSRNP2 | -1.18628865 |
| TMEM156 | -1.214018297 | CSRNP3 | 3.260522579 |
| TMEM160 | -1.029440624 | CT45A1 | 2.458105469 |
| TMEM215 | -4.744630834 | CT62 | -3.837786224 |
| TMEM255B | -1.99415613 | CTB-178M22.2 | -2.299097576 |
| TMEM37 | 1.632237446 | CTH | 1.984264741 |
| TMEM45A | 1.653034264 | CTHRC1 | 2.503996307 |
| TMEM74B | -1.234664939 | CTNNB1 | 1.146767507 |
| TMEM92 | 1.076197324 | CTNND2 | 9.126420175 |
| TMEM92-AS1 | 1.524441233 | CTPS1 | -2.159678595 |
| TMOD1 | 2.90052405 | CTRB1 | -4.327109377 |
| TMTC1 | 7.10536809 | CTRB2 | -5.121575041 |
| TNC | 2.375770554 | CTSA | 1.771537853 |
| TNFAIP6 | 4.490538072 | CTSB | 1.096322514 |
| TNFRSF10D | 1.009912621 | CTSC | 1.399127703 |
| TNFRSF11B | 10.3539889 | CTSD | 1.473266274 |
| TNFRSF19 | 2.729258322 | CTSH | 1.143236675 |
| TNFSF13 | -1.073962013 | CTSO | 1.717398097 |
| TNIP3 | 1.919864514 | CTSV | 1.92903203 |
| TNK1 | -1.479731137 | CTSW | -3.06258727 |
| TNS1 | 3.960198804 | CTSZ | 1.288681142 |
| TNS4 | 1.339154457 | CTTNBP2 | 3.000728785 |
| TOR4A | -1.298101148 | CTU1 | -1.107705784 |
| TOX3 | -3.201651277 | CTXN1 | -1.243500482 |
| TPP1 | 1.159997169 | CTXND1 | 3.50923845 |
| TPPP | -1.160294359 | CU634019.1 | -1.483546815 |
| TPST1 | 1.386902746 | CUBN | 1.172382651 |
| TRABD2A | 2.178228583 | CUL4A | 1.51904298 |
| TRAF1 | -1.071585877 | CUL4B | 1.422108238 |
| TRAM1L1 | 1.362826031 | CUTC | -1.12677195 |
| TREM1 | 4.831845358 | CXCL1 | -1.116196298 |
| TRIM2 | 2.180842027 | CXCL12 | 8.402508851 |
| TRIM22 | 1.677657198 | CXCL14 | 5.57912226 |
| TRIM31 | 3.687635155 | CXCL2 | -2.098173356 |
| TRIM36 | -1.47260802 | CXCL3 | -1.034635771 |
| TRIM45 | 1.515504572 | CXCL5 | -2.622013135 |
| TRIM67 | -1.975614777 | CXCL8 | -5.051504555 |
| TRMT9B | 2.259360469 | CXCR4 | 2.041080087 |
| TRNP1 | -1.141201504 | CXorf57 | 2.696164852 |
| TRPA1 | 2.912260222 | CXXC4 | 2.875557546 |
| TRPC4 | 7.758773235 | CXXC5 | 3.241639537 |
| TRPM3 | 4.306862554 | CYB561 | -1.078406642 |
| TRPM6 | 1.016426671 | CYBRD1 | 1.454639662 |
| TRPS1 | 1.894185229 | CYCSP10 | 1.477646083 |
| TRPV3 | -1.482026924 | CYP17A1 | 4.756028129 |
| TSHZ3 | -2.128158623 | CYP1A1 | -3.158422868 |
| TSPAN10 | 1.065078565 | CYP24A1 | -2.750094323 |
| TSPAN15 | -1.380372844 | CYP26A1 | -3.414825521 |
| TSPAN2 | -1.628169924 | CYP26B1 | 1.461965099 |
| TSPAN33 | -1.18649412 | CYP2C9 | -4.41253579 |
| TSPAN5 | -1.72713088 | CYP2E1 | -2.195527906 |
| TSPAN7 | 2.140475894 | CYP2J2 | 2.657161174 |
| TTC39C | 1.026039884 | CYP2U1 | 1.340779854 |
| TTC9 | -1.198496629 | CYP2W1 | -2.248852205 |
| TUBA3FP | 1.13613828 | CYP39A1 | 1.383617508 |
| TUBB4A | -1.55634176 | CYP3A5 | -1.560819421 |
| TUSC1 | -1.519634369 | CYP46A1 | 2.071823884 |
| TXNIP | -2.413739633 | CYP4F11 | 1.623075365 |
| U2AF1L5 | -1.511546594 | CYP4F12 | 2.082583758 |
| U52111.1 | 2.109384328 | CYP4F29P | -4.988663808 |
| UBA7 | 1.663732278 | CYP4F35P | -8.491127521 |
| UBAC2-AS1 | -1.285432586 | CYP4X1 | 2.533878319 |
| UBASH3B | -1.079030584 | CYP51A1 | -1.377067396 |
| UBE2QL1 | -3.182576058 | CYP7B1 | 3.316815768 |
| UBE2SP1 | 1.167540586 | CYR61 | -1.838003481 |
| UFSP1 | -1.259644321 | CYSLTR2 | 8.952152884 |
| UGT1A1 | 2.054997915 | CYSRT1 | -1.355866163 |
| UGT1A7 | 2.171721311 | CYTH3 | -1.165230282 |
| UGT2B7 | 1.263627489 | CYTH4 | -2.189037894 |
| UGT8 | -2.074468186 | D2HGDH | 1.145223969 |
| UNC13A | -1.22783889 | DAAM2 | 4.817664112 |
| UNC5D | -2.923810675 | DAB2 | 3.59369378 |
| UNC80 | 2.015516749 | DACH1 | 9.946813133 |
| UPK1B | 1.873834571 | DACT1 | 3.078791225 |
| UPP1 | -1.012894635 | DACT3 | 2.297346115 |
| USH1C | 4.715884414 | DAGLB | -1.922131124 |
| USP18 | -1.075493866 | DAPK1 | -3.055172325 |
| USP2 | -3.152854858 | DBI | 1.457541921 |
| USP44 | -2.300511548 | DBP | 1.572674784 |
| USP51 | 1.258463599 | DCAF12L2 | 6.413596137 |
| USP53 | -1.252377142 | DCBLD1 | -1.878131117 |
| UST | 1.683427253 | DCBLD2 | -3.302810919 |
| VAC14-AS1 | 1.740468346 | DCDC2 | -4.092560907 |
| VASH2 | 1.487270569 | DCHS1 | 3.304400485 |
| VAV1 | -1.406714662 | DCLK1 | 1.326508627 |
| VAV3 | 1.559130329 | DCLK3 | 4.264327259 |
| VCAN | 2.58006834 | DCN | 4.994091773 |
| VDR | 1.083954826 | DCTPP1 | -1.155256653 |
| VEGFD | 1.634748007 | DCUN1D3 | -1.215851045 |
| VGF | -1.602627762 | DCUN1D5 | -1.290644939 |
| VGLL3 | 1.584576016 | DCX | 3.563001884 |
| VIPR1 | 1.018617997 | DDAH2 | -1.524092242 |
| VSTM1 | -4.540688524 | DDC | -3.750181902 |
| VSTM4 | -1.7336031 | DDIT3 | -2.641245228 |
| VWA1 | -1.011802384 | DDIT4L | 3.508765917 |
| VWA5A | 1.856157089 | DDR2 | 4.587654914 |
| VWA5B2 | -2.153155811 | DDX10 | -1.647334839 |
| VWDE | -1.419781988 | DDX11-AS1 | -1.306621607 |
| WASF3 | 4.69266677 | DDX51 | -1.078210479 |
| WFDC21P | 2.697619547 | DDX58 | -1.049879787 |
| WIPF1 | 1.050621172 | DDX60L | -1.22905618 |
| WIPI1 | 1.710584506 | DECR1 | 1.818853612 |
| WISP1 | 5.176144571 | DEFB131E | 7.416440288 |
| WISP2 | 1.772363118 | DEGS2 | -2.367287403 |
| WNK2 | -1.048686454 | DENND1C | -2.88384161 |
| WNT16 | 6.810069787 | DENND2A | -1.946596866 |
| WNT5A | 4.852812634 | DEPDC1 | 1.315959652 |
| WNT5A-AS1 | 3.272534597 | DEPDC1B | 1.547325897 |
| WNT7B | -1.779375728 | DEPTOR | 1.561167491 |
| WSCD1 | -1.189068679 | DES | 4.44973576 |
| WT1-AS | -1.947399429 | DEXI | 1.104981494 |
| WTIP | -1.357910529 | DGKH | -1.129595649 |
| XKR8 | 1.093913815 | DGKI | 3.352752224 |
| XKRX | 1.443052505 | DGLUCY | 1.031004108 |
| YDJC | -1.053298638 | DHCR24 | 1.085557256 |
| ZDHHC22 | -1.870535772 | DHFR | 1.018184189 |
| ZEB2 | 1.833537031 | DHFR2 | 1.370969679 |
| ZFHX2 | -1.124973058 | DHFRP1 | 1.516170661 |
| ZFP3 | -3.035307694 | DHRS2 | -2.422730577 |
| ZFP30 | -2.57168699 | DHRS3 | -2.388642725 |
| ZFPM2 | 1.120510822 | DHRS9 | -2.767857188 |
| ZGPAT | -1.022288046 | DHX37 | -1.132585546 |
| ZMIZ1 | 1.003858674 | DIO2 | 2.952022399 |
| ZMIZ1-AS1 | 2.608934462 | DIO3OS | -1.869396457 |
| ZNF136 | -2.190027755 | DIRAS1 | 1.078450143 |
| ZNF365 | -1.582724451 | DIRAS2 | 7.79311485 |
| ZNF385C | -1.099395674 | DISP1 | 2.468674464 |
| ZNF385D | 5.36104046 | DISP2 | -2.044833995 |
| ZNF418 | -3.086826768 | DKFZP434K028 | -5.875952629 |
| ZNF423 | 2.394357738 | DKK1 | -5.409450558 |
| ZNF468 | -2.275157154 | DKK3 | -1.227572354 |
| ZNF471 | -3.567352717 | DLG2 | 3.833096572 |
| ZNF503 | 1.049167284 | DLGAP1-AS5 | 4.707032733 |
| ZNF525 | -1.198338378 | DLGAP3 | 1.820650215 |
| ZNF542P | -1.751536903 | DLGAP5 | 1.725124521 |
| ZNF608 | -4.086026806 | DLL1 | 2.011877324 |
| ZNF704 | 2.31803275 | DLL3 | -1.395767534 |
| ZNF711 | -2.025594258 | DLX5 | 11.73280284 |
| ZNF738 | -4.648989856 | DLX6 | 8.408523174 |
| ZNF804A | 1.986336204 | DLX6-AS1 | 4.14591955 |
| ZNF813 | -1.645535393 | DMBT1 | 2.550314092 |
| ZNF823 | -2.068570276 | DMC1 | 1.399387312 |
| ZNF853 | -1.253960787 | DMD | 6.316665254 |
| ZNF860 | 1.198300412 | DMGDH | -1.851621655 |
| ZP1 | 5.356212076 | DMRT3 | 2.771835424 |
| ZSCAN18 | -2.735201706 | DMRTA1 | -1.02027645 |
|  |  | DMTN | -1.52315844 |
|  |  | DNAAF3 | -1.022111416 |
|  |  | DNAAF4 | -1.71525163 |
|  |  | DNAH17 | 1.44157872 |
|  |  | DNAH17-AS1 | 2.317500018 |
|  |  | DNAH5 | -4.101692439 |
|  |  | DNAH7 | -2.008290487 |
|  |  | DNAJB13 | -3.006613058 |
|  |  | DNAJB2 | -1.090295554 |
|  |  | DNAJB4 | 1.015049042 |
|  |  | DNAJC17 | 1.029018588 |
|  |  | DNAJC22 | -1.026509297 |
|  |  | DNAJC3-DT | 1.321431059 |
|  |  | DNAJC4 | 1.039193371 |
|  |  | DNAJC6 | -1.255618627 |
|  |  | DNER | 4.292587497 |
|  |  | DNM1 | -1.02976558 |
|  |  | DNM1P51 | 1.162368641 |
|  |  | DNMT3A | 1.281155695 |
|  |  | DNMT3B | 1.458230412 |
|  |  | DOC2A | 1.38851116 |
|  |  | DOCK2 | -2.172764362 |
|  |  | DOCK5 | -1.907137603 |
|  |  | DOCK6 | -1.06552198 |
|  |  | DOK4 | -2.189805065 |
|  |  | DOK5 | 3.988710453 |
|  |  | DOK6 | 2.555210602 |
|  |  | DOK7 | -3.034786674 |
|  |  | DOP1B | -1.273354825 |
|  |  | DPCD | -1.453534522 |
|  |  | DPEP1 | 3.820195308 |
|  |  | DPF3 | -1.62477868 |
|  |  | DPP4 | 2.139757979 |
|  |  | DPY19L2P3 | -1.063816719 |
|  |  | DPY19L2P4 | 4.832443256 |
|  |  | DPYSL3 | 3.075890681 |
|  |  | DPYSL5 | 1.185809775 |
|  |  | DRAP1 | -1.045853669 |
|  |  | DRC7 | -3.459457222 |
|  |  | DRD1 | -5.954048935 |
|  |  | DSC2 | 1.850867576 |
|  |  | DSCAML1 | -2.553620875 |
|  |  | DSE | 1.463980329 |
|  |  | DSEL | 5.444555619 |
|  |  | DTNA | 3.532295586 |
|  |  | DTX1 | 2.358836093 |
|  |  | DTX2 | -1.290365453 |
|  |  | DTX4 | 1.119319761 |
|  |  | DUBR | 1.414550206 |
|  |  | DUOX2 | -3.458899297 |
|  |  | DUOXA1 | -2.894740689 |
|  |  | DUOXA2 | -5.168816412 |
|  |  | DUS4L | -1.122745121 |
|  |  | DUSP18 | -1.087756446 |
|  |  | DUSP2 | 2.847210243 |
|  |  | DUSP6 | -2.445979425 |
|  |  | DUSP8 | -1.113961008 |
|  |  | DVL1 | -1.407104252 |
|  |  | DYNC1I1 | 7.340000651 |
|  |  | DYNC2H1 | -1.215449276 |
|  |  | DYSF | -1.565170432 |
|  |  | DZANK1 | -1.197761834 |
|  |  | E2F8 | 1.548394051 |
|  |  | EBF1 | 2.305309856 |
|  |  | EBF2 | 2.327869135 |
|  |  | EBF4 | 1.510132415 |
|  |  | ECE1 | 1.148776486 |
|  |  | ECEL1 | 5.938524801 |
|  |  | ECHDC3 | 1.063183331 |
|  |  | ECM1 | 2.576710777 |
|  |  | EDA | 1.553244134 |
|  |  | EDIL3 | 4.183142773 |
|  |  | EDN1 | -3.970798371 |
|  |  | EDN2 | 1.225313446 |
|  |  | EDNRA | 5.667194072 |
|  |  | EDNRB | 9.537274279 |
|  |  | EEF1A2 | -1.393998277 |
|  |  | EEFSEC | 1.178843018 |
|  |  | EEPD1 | -1.836834482 |
|  |  | EFCAB10 | -2.097108101 |
|  |  | EFCAB6 | -1.57989374 |
|  |  | EFEMP1 | 3.172788563 |
|  |  | EFNA1 | 2.1076323 |
|  |  | EFNA2 | 1.348988803 |
|  |  | EFNA4 | 1.168734418 |
|  |  | EFNB3 | 1.638212048 |
|  |  | EFR3B | -1.460225366 |
|  |  | EGF | 4.587180623 |
|  |  | EGFEM1P | 3.567739926 |
|  |  | EGFLAM | 4.895885804 |
|  |  | EGFR | -3.112801891 |
|  |  | EGFR-AS1 | -4.366490634 |
|  |  | EGLN2 | 1.126253989 |
|  |  | EGLN3 | 3.489879415 |
|  |  | EGR1 | -3.936010887 |
|  |  | EHBP1 | -1.972490129 |
|  |  | EHBP1L1 | -1.15869969 |
|  |  | EHD1 | -1.39452362 |
|  |  | EHD4 | -1.695283125 |
|  |  | EHF | -1.997171544 |
|  |  | EHHADH | 1.390230851 |
|  |  | EID1 | 1.135706468 |
|  |  | EID3 | -2.430915013 |
|  |  | EIF4E3 | 1.749392232 |
|  |  | EIF4EBP2 | 1.423390605 |
|  |  | ELANE | 4.580563226 |
|  |  | ELAVL2 | 3.939900732 |
|  |  | ELAVL4 | 2.471596156 |
|  |  | ELDR | -3.832670532 |
|  |  | ELF3 | -6.818055333 |
|  |  | ELF4 | -1.233526773 |
|  |  | ELFN1 | 3.738219724 |
|  |  | ELK3 | -1.51674865 |
|  |  | ELMO1 | -1.763438264 |
|  |  | ELMOD1 | -1.628381295 |
|  |  | ELN | -3.481301691 |
|  |  | ELOA-AS1 | -2.503815579 |
|  |  | ELOVL1 | -1.83087295 |
|  |  | ELOVL2 | 1.581983117 |
|  |  | ELOVL3 | 3.709139094 |
|  |  | ELOVL5 | 1.195562331 |
|  |  | ELSPBP1 | 4.930263309 |
|  |  | EMC10 | 1.083016045 |
|  |  | EMC3 | 1.157058663 |
|  |  | EMC6 | -1.112189597 |
|  |  | EMG1 | -1.238163684 |
|  |  | EMILIN1 | -1.565750532 |
|  |  | EML2-AS1 | -3.833205193 |
|  |  | EML4 | 1.088975909 |
|  |  | EMP2 | 1.170915211 |
|  |  | EMX1 | 2.565204163 |
|  |  | EMX2OS | 7.891702691 |
|  |  | EN1 | -2.170482873 |
|  |  | ENAH | -1.030043652 |
|  |  | ENC1 | -1.569567758 |
|  |  | ENDOV | -1.411464933 |
|  |  | ENOX1 | 1.792506321 |
|  |  | ENPP1 | 1.959566017 |
|  |  | ENPP2 | 11.22894844 |
|  |  | ENPP4 | 1.669153721 |
|  |  | ENPP5 | 4.997502245 |
|  |  | ENTPD1 | 1.040032698 |
|  |  | ENTPD2 | 1.882184521 |
|  |  | ENTPD8 | 1.823967123 |
|  |  | EPB41L3 | 12.82569377 |
|  |  | EPB41L4A-AS1 | -2.076089511 |
|  |  | EPB41L4B | -1.29300998 |
|  |  | EPGN | 2.756514634 |
|  |  | EPHA10 | -1.945649179 |
|  |  | EPHA2 | -3.575854224 |
|  |  | EPHA3 | 14.10720749 |
|  |  | EPHA5 | 3.097646388 |
|  |  | EPHA5-AS1 | 1.992999882 |
|  |  | EPHA6 | 2.877829214 |
|  |  | EPHA7 | 4.613753348 |
|  |  | EPHB2 | -1.882554287 |
|  |  | EPHB3 | 3.245472717 |
|  |  | EPHB6 | 2.61523976 |
|  |  | EPHX1 | 2.170344044 |
|  |  | EPHX2 | 1.503398854 |
|  |  | EPM2AIP1 | 1.34073875 |
|  |  | EPN3 | -1.782374711 |
|  |  | EPOP | -1.586106367 |
|  |  | EPPK1 | -2.880619295 |
|  |  | EPS8L1 | -1.673657923 |
|  |  | EPS8L2 | -3.923754948 |
|  |  | EPS8L3 | -4.407800481 |
|  |  | EPYC | 5.100770263 |
|  |  | ERBB3 | -1.294956192 |
|  |  | ERBB4 | 2.662603687 |
|  |  | EREG | -5.636987722 |
|  |  | ERFE | -1.033020397 |
|  |  | ERG | 4.359409793 |
|  |  | ERGIC1 | 1.867029009 |
|  |  | ERICH2 | 3.401550568 |
|  |  | ERICH6-AS1 | 1.421570122 |
|  |  | ERN1 | -2.627928316 |
|  |  | ERP44 | 1.126905657 |
|  |  | ERRFI1 | -3.499056778 |
|  |  | ESCO2 | 1.66984392 |
|  |  | ESM1 | 4.330226294 |
|  |  | ESRRG | 3.533988877 |
|  |  | ESX1 | 3.487627946 |
|  |  | ESYT1 | -1.120196467 |
|  |  | ETFB | 1.486946844 |
|  |  | ETFDH | 1.182382746 |
|  |  | ETNPPL | 5.183331868 |
|  |  | ETS1 | -3.508378239 |
|  |  | EVA1B | -2.182477719 |
|  |  | EVA1C | 1.133085875 |
|  |  | EVC2 | -1.668125039 |
|  |  | EVPL | -3.092559195 |
|  |  | EWSAT1 | 5.953670964 |
|  |  | EXOC3L2 | -1.678122323 |
|  |  | EXOC3L4 | -1.389938817 |
|  |  | EXPH5 | -1.903511266 |
|  |  | EXT1 | 1.044801958 |
|  |  | EYA1 | 7.00334574 |
|  |  | EYA2 | -3.312770596 |
|  |  | F11R | -1.521260299 |
|  |  | F12 | -1.635799944 |
|  |  | F2 | -4.7555366 |
|  |  | F2R | -1.177850854 |
|  |  | F2RL1 | -2.862769823 |
|  |  | F2RL2 | 1.890874902 |
|  |  | F5 | 3.12796184 |
|  |  | FA2H | -1.124395741 |
|  |  | FAAH2 | -1.967850695 |
|  |  | FAAP100 | -1.0638381 |
|  |  | FABP2 | 4.705132363 |
|  |  | FABP4 | 4.747206117 |
|  |  | FABP6 | 1.895886307 |
|  |  | FABP9 | 3.93906731 |
|  |  | FADD | -1.203806194 |
|  |  | FADS2 | 1.773589933 |
|  |  | FADS2P1 | -5.611979282 |
|  |  | FADS3 | -1.634290686 |
|  |  | FAH | 1.024735808 |
|  |  | FAHD2CP | -1.033264116 |
|  |  | FAM106A | 1.067721709 |
|  |  | FAM110B | 1.379698391 |
|  |  | FAM110D | 6.38693444 |
|  |  | FAM111B | 1.035468341 |
|  |  | FAM120B | 1.601683865 |
|  |  | FAM131A | -2.217196163 |
|  |  | FAM131C | -3.761279691 |
|  |  | FAM133A | 5.665368191 |
|  |  | FAM13A | 1.192578846 |
|  |  | FAM13A-AS1 | 1.856590929 |
|  |  | FAM13C | 6.826037365 |
|  |  | FAM149A | 1.031411255 |
|  |  | FAM155A | 6.21053644 |
|  |  | FAM155A-IT1 | 4.47472647 |
|  |  | FAM156B | 3.427198755 |
|  |  | FAM157B | 2.312015382 |
|  |  | FAM157C | 2.779765936 |
|  |  | FAM160A1 | 2.939175323 |
|  |  | FAM163A | 6.609156933 |
|  |  | FAM167B | -2.445990011 |
|  |  | FAM172A | 1.545816133 |
|  |  | FAM174B | -1.135552517 |
|  |  | FAM181B | 2.177915206 |
|  |  | FAM182B | -1.069279668 |
|  |  | FAM184A | 1.797262299 |
|  |  | FAM198B | 1.453219564 |
|  |  | FAM19A2 | 1.88315838 |
|  |  | FAM19A5 | -3.692422893 |
|  |  | FAM20A | 4.54686234 |
|  |  | FAM20B | 1.08524814 |
|  |  | FAM217B | 1.25512857 |
|  |  | FAM222A | -1.3840645 |
|  |  | FAM224B | -2.973127702 |
|  |  | FAM238C | 1.013777973 |
|  |  | FAM241B | -1.261045238 |
|  |  | FAM24B | -1.631383279 |
|  |  | FAM27C | -2.271785565 |
|  |  | FAM43A | 2.746067346 |
|  |  | FAM43B | 1.468272912 |
|  |  | FAM49A | 3.750398338 |
|  |  | FAM50A | -1.089653552 |
|  |  | FAM78A | 1.407064509 |
|  |  | FAM83A | 1.247273552 |
|  |  | FAM83D | 1.939135344 |
|  |  | FAM83G | -1.630854752 |
|  |  | FAM84A | 4.816016406 |
|  |  | FAM85B | -2.440308595 |
|  |  | FAM86B1 | -1.20322803 |
|  |  | FAM86B2 | -1.912773119 |
|  |  | FAM86B3P | -1.630722167 |
|  |  | FAM86FP | -1.513966869 |
|  |  | FAM86HP | -1.236517784 |
|  |  | FAM86JP | -1.264753532 |
|  |  | FAM89A | -1.851605392 |
|  |  | FAM90A1 | -4.429332099 |
|  |  | FAM90A2P | -4.709431315 |
|  |  | FAM98A | 1.117763015 |
|  |  | FAM98B | 1.228873601 |
|  |  | FANCE | -1.185783562 |
|  |  | FANK1 | -1.296911686 |
|  |  | FAP | 3.213201101 |
|  |  | FAR2 | 1.196300393 |
|  |  | FAR2P1 | -2.385497483 |
|  |  | FAR2P2 | 1.581763112 |
|  |  | FAT3 | -4.011897995 |
|  |  | FBLIM1 | -3.075384811 |
|  |  | FBLN1 | 1.548616967 |
|  |  | FBLN2 | 1.486058675 |
|  |  | FBLN5 | 5.055392664 |
|  |  | FBN1 | 4.256728367 |
|  |  | FBXL19-AS1 | -1.044244327 |
|  |  | FBXL21 | 1.733186617 |
|  |  | FBXL7 | 3.504517035 |
|  |  | FBXO17 | -1.234814005 |
|  |  | FBXO2 | -1.066153248 |
|  |  | FBXO27 | -1.836467239 |
|  |  | FBXO36 | -1.339974229 |
|  |  | FBXO42 | -1.215692896 |
|  |  | FBXO43 | 1.180802226 |
|  |  | FBXO48 | -1.162395552 |
|  |  | FBXO6 | -1.380314786 |
|  |  | FBXW10 | 1.294170729 |
|  |  | FCGBP | -3.82890771 |
|  |  | FCHO1 | -2.655440744 |
|  |  | FCMR | -1.573313186 |
|  |  | FDXR | -1.253485387 |
|  |  | FENDRR | 11.56263975 |
|  |  | FEZ1 | 1.340034521 |
|  |  | FEZ2 | -1.297148696 |
|  |  | FEZF1 | 6.161663053 |
|  |  | FEZF1-AS1 | 5.19152371 |
|  |  | FGA | -2.227576049 |
|  |  | FGD4 | -1.354796404 |
|  |  | FGD6 | -2.504286711 |
|  |  | FGF1 | -2.598819477 |
|  |  | FGF10 | 9.20111124 |
|  |  | FGF10-AS1 | 4.026747864 |
|  |  | FGF11 | -2.455232415 |
|  |  | FGF12 | 1.625102865 |
|  |  | FGF13 | 3.114905471 |
|  |  | FGF2 | -2.14867319 |
|  |  | FGF7 | 7.092484742 |
|  |  | FGF9 | 9.365426192 |
|  |  | FGFR1 | -1.682894371 |
|  |  | FGFR4 | -2.250436328 |
|  |  | FGFRL1 | 1.142622428 |
|  |  | FGL1 | -3.9127701 |
|  |  | FHDC1 | 4.544317901 |
|  |  | FHL1 | -1.015305616 |
|  |  | FIBIN | 2.035624629 |
|  |  | FICD | -1.260742315 |
|  |  | FILIP1 | 3.851130729 |
|  |  | FIRRE | 2.297397595 |
|  |  | FJX1 | -1.060607787 |
|  |  | FKBP10 | 1.510633776 |
|  |  | FKBP7 | 3.201898393 |
|  |  | FLII | -1.001579432 |
|  |  | FLJ20021 | -1.174545298 |
|  |  | FLJ22447 | 1.375018751 |
|  |  | FLJ31104 | -1.960686758 |
|  |  | FLJ45513 | -1.99200954 |
|  |  | FLNA | -1.084843832 |
|  |  | FLNB | -1.323155589 |
|  |  | FLNB-AS1 | -1.077749827 |
|  |  | FLNC | -3.591472402 |
|  |  | FLRT2 | 6.836259784 |
|  |  | FLRT3 | -4.348816991 |
|  |  | FLT1 | 3.090352496 |
|  |  | FMN2 | -1.801469336 |
|  |  | FMO3 | 5.093253078 |
|  |  | FMO5 | 1.543878853 |
|  |  | FN1 | 3.122913226 |
|  |  | FNDC1 | 8.614468732 |
|  |  | FNDC1-IT1 | 6.370915331 |
|  |  | FNDC3A | 1.735377901 |
|  |  | FNDC8 | -2.040961421 |
|  |  | FNIP2 | 1.093176504 |
|  |  | FOLH1 | 4.097967389 |
|  |  | FOS | 2.131151976 |
|  |  | FOSB | -1.802038455 |
|  |  | FOSL1 | -3.492448571 |
|  |  | FOSL2 | -1.129766846 |
|  |  | FOXA1 | -1.150963995 |
|  |  | FOXA2 | -3.78698934 |
|  |  | FOXA3 | -3.841709009 |
|  |  | FOXB1 | 6.346414151 |
|  |  | FOXC1 | 1.383402842 |
|  |  | FOXD4L1 | -1.355734923 |
|  |  | FOXD4L4 | -3.829879508 |
|  |  | FOXE1 | -1.274766581 |
|  |  | FOXF1 | 12.20340067 |
|  |  | FOXG1 | 4.586539422 |
|  |  | FOXI3 | 5.667945933 |
|  |  | FOXJ1 | -4.895261497 |
|  |  | FOXL1 | -2.153504216 |
|  |  | FOXL2 | -2.745403627 |
|  |  | FOXL2NB | -1.312065455 |
|  |  | FOXM1 | 1.098507131 |
|  |  | FOXN3 | 1.222793061 |
|  |  | FOXO1 | 1.344099765 |
|  |  | FOXO6 | 2.112172523 |
|  |  | FOXP2 | 5.637703638 |
|  |  | FOXS1 | 4.792573067 |
|  |  | FP236383.1 | 2.06175447 |
|  |  | FPR1 | 1.520751945 |
|  |  | FPR2 | 3.009402997 |
|  |  | FPR3 | 5.471284369 |
|  |  | FRAS1 | -1.095353214 |
|  |  | FRAT2 | 1.946848959 |
|  |  | FREM1 | 3.700925403 |
|  |  | FREM2 | 8.095050696 |
|  |  | FRMD3 | -3.794405928 |
|  |  | FRMD4B | 1.369685525 |
|  |  | FRMD5 | -3.066861981 |
|  |  | FRMD6 | -1.602421733 |
|  |  | FRMD8 | -1.054235655 |
|  |  | FRMPD3 | 7.144656967 |
|  |  | FRRS1 | 1.361599657 |
|  |  | FRRS1L | -4.522515474 |
|  |  | FRY | 4.791073128 |
|  |  | FSCN2 | -3.294714478 |
|  |  | FSD1 | -1.870172902 |
|  |  | FSIP1 | -1.528823999 |
|  |  | FST | 3.160390247 |
|  |  | FSTL1 | 4.218571803 |
|  |  | FSTL3 | -1.384926989 |
|  |  | FSTL4 | 3.19389747 |
|  |  | FSTL5 | 2.579966947 |
|  |  | FTCDNL1 | 1.28906051 |
|  |  | FTL | 1.735964051 |
|  |  | FTLP3 | 2.688174855 |
|  |  | FUCA1 | 1.041429619 |
|  |  | FUNDC2 | 1.094451811 |
|  |  | FURIN | 1.812624748 |
|  |  | FUT2 | 1.556520522 |
|  |  | FUT3 | -4.385654488 |
|  |  | FXYD2 | -5.656346771 |
|  |  | FXYD5 | -1.794338984 |
|  |  | FYN | 4.521299403 |
|  |  | FZD1 | 1.790818759 |
|  |  | FZD4 | 1.413865896 |
|  |  | G0S2 | -5.356979488 |
|  |  | GAA | 1.257947846 |
|  |  | GAB1 | 2.404426182 |
|  |  | GABBR1 | 1.300653475 |
|  |  | GABBR2 | 4.869360492 |
|  |  | GABRA1 | 6.767706469 |
|  |  | GABRA2 | 7.084136968 |
|  |  | GABRA3 | 1.393925067 |
|  |  | GABRA5 | 1.046052495 |
|  |  | GABRB2 | 8.463413604 |
|  |  | GABRD | 1.19614309 |
|  |  | GABRG1 | 5.162775035 |
|  |  | GABRG3 | 1.275551619 |
|  |  | GABRQ | 4.24498856 |
|  |  | GABRR2 | 2.7332327 |
|  |  | GACAT2 | -2.436526503 |
|  |  | GAD1 | 1.892930409 |
|  |  | GADD45A | -3.801650323 |
|  |  | GAL3ST1 | -4.615697446 |
|  |  | GAL3ST4 | -2.256596926 |
|  |  | GALC | 1.675335073 |
|  |  | GALE | -1.261603357 |
|  |  | GALM | -1.200716045 |
|  |  | GALNS | 1.093011195 |
|  |  | GALNT10 | -1.537220689 |
|  |  | GALNT15 | 6.500811626 |
|  |  | GALNT16 | 1.343689141 |
|  |  | GALNT17 | -5.394267481 |
|  |  | GALNT18 | -2.090573371 |
|  |  | GALNT2 | 2.69180511 |
|  |  | GALNT3 | 1.753403023 |
|  |  | GALNT6 | 2.612335709 |
|  |  | GALNT9 | -1.62106756 |
|  |  | GAP43 | 9.413075671 |
|  |  | GARS | -1.267023828 |
|  |  | GAS1 | 9.696288321 |
|  |  | GAS1RR | 4.529829394 |
|  |  | GAS2 | 2.51122491 |
|  |  | GAS2L2 | -5.033530224 |
|  |  | GAS2L3 | 1.060354809 |
|  |  | GAS5 | -1.167536126 |
|  |  | GAS5-AS1 | 1.798546054 |
|  |  | GAS6 | 1.179700897 |
|  |  | GAS6-AS1 | 1.631823604 |
|  |  | GAS6-DT | 2.280187792 |
|  |  | GAS7 | 3.253218251 |
|  |  | GASAL1 | 1.480075807 |
|  |  | GATA2 | 3.657945003 |
|  |  | GATA3 | -1.833551824 |
|  |  | GATA4 | 1.010136023 |
|  |  | GATA6 | -3.057486004 |
|  |  | GATA6-AS1 | -3.850868566 |
|  |  | GBE1 | 1.067081511 |
|  |  | GBP1 | 2.51014998 |
|  |  | GBP4 | 3.326435288 |
|  |  | GBX2 | -1.168676715 |
|  |  | GCC1 | -1.692925425 |
|  |  | GCC2-AS1 | -1.466866237 |
|  |  | GCNT3 | -2.711678512 |
|  |  | GCOM1 | -2.025520035 |
|  |  | GCSAM | -1.351314873 |
|  |  | GDA | 5.227210373 |
|  |  | GDF10 | 6.549727223 |
|  |  | GDF15 | -4.006680882 |
|  |  | GDF6 | 4.173491827 |
|  |  | GDF7 | 3.776780722 |
|  |  | GDPD1 | -1.544831236 |
|  |  | GDPD5 | -1.465848108 |
|  |  | GET4 | -1.298480113 |
|  |  | GFAP | -1.254804529 |
|  |  | GFOD1 | -1.924553537 |
|  |  | GFPT2 | -1.915662945 |
|  |  | GFRA2 | 5.663577342 |
|  |  | GFY | 4.722289131 |
|  |  | GGH | 1.678491601 |
|  |  | GGN | -1.38299362 |
|  |  | GGNBP2 | -1.100647298 |
|  |  | GGT1 | -1.58562518 |
|  |  | GGT4P | -8.449350833 |
|  |  | GGT5 | 5.849264764 |
|  |  | GGT8P | -4.173181036 |
|  |  | GGTLC4P | 2.451576206 |
|  |  | GHET1 | -2.664010462 |
|  |  | GHR | 5.916774709 |
|  |  | GIMAP2 | 4.161230019 |
|  |  | GIPC1 | -1.244435619 |
|  |  | GIPC3 | 2.192395879 |
|  |  | GJB2 | 2.526435525 |
|  |  | GJD3 | -2.773034719 |
|  |  | GLB1 | 1.244834449 |
|  |  | GLB1L3 | 2.934265174 |
|  |  | GLCE | 2.09386515 |
|  |  | GLDC | -2.556070498 |
|  |  | GLDN | 3.721336365 |
|  |  | GLI2 | -3.86467561 |
|  |  | GLI3 | 5.580927415 |
|  |  | GLIPR2 | 1.623415928 |
|  |  | GLIS1 | 2.971794061 |
|  |  | GLIS2 | -1.033927437 |
|  |  | GLP2R | 1.644380381 |
|  |  | GLRB | -1.18372536 |
|  |  | GLRX | 1.057437757 |
|  |  | GLS | -1.339512492 |
|  |  | GLT8D2 | 2.83711219 |
|  |  | GLTPD2 | -1.118162142 |
|  |  | GLUD2 | 1.607717801 |
|  |  | GLUL | 5.447533055 |
|  |  | GM2A | 1.715487175 |
|  |  | GMDS | 1.72772695 |
|  |  | GMIP | -1.041014373 |
|  |  | GMPR | 1.703056633 |
|  |  | GNAI1 | -1.776778061 |
|  |  | GNAO1 | 5.442214579 |
|  |  | GNAS | 1.236160006 |
|  |  | GNB1L | -1.570337355 |
|  |  | GNB4 | 1.701663645 |
|  |  | GNG13 | -1.076959898 |
|  |  | GNG2 | 2.343476248 |
|  |  | GNS | 1.216068906 |
|  |  | GOLGA6A | -2.995366145 |
|  |  | GOLGA8N | 1.704861512 |
|  |  | GOLGA8O | 1.373394959 |
|  |  | GOLGA8Q | 2.185800564 |
|  |  | GOLT1A | -4.435325247 |
|  |  | GP6 | -1.747531739 |
|  |  | GPC1 | 1.046416842 |
|  |  | GPC2 | -1.61894 |
|  |  | GPC3 | 8.709905808 |
|  |  | GPC4 | 6.161941465 |
|  |  | GPC5 | 10.31461811 |
|  |  | GPCPD1 | 1.046369662 |
|  |  | GPD1 | -1.140909768 |
|  |  | GPER1 | 4.489287587 |
|  |  | GPI | 1.316739228 |
|  |  | GPIHBP1 | 2.342282213 |
|  |  | GPNMB | 4.924616998 |
|  |  | GPR132 | -2.80639926 |
|  |  | GPR137C | 3.820514565 |
|  |  | GPR139 | 4.82118117 |
|  |  | GPR153 | -1.178690573 |
|  |  | GPR155 | 1.396460546 |
|  |  | GPR158 | 1.236453322 |
|  |  | GPR160 | -2.408533348 |
|  |  | GPR161 | 1.273010523 |
|  |  | GPR162 | 3.390722994 |
|  |  | GPR176 | 1.44105068 |
|  |  | GPR20 | 3.039761464 |
|  |  | GPR27 | 1.277032533 |
|  |  | GPR35 | -1.477019891 |
|  |  | GPR37 | 1.533960207 |
|  |  | GPR37L1 | -4.410399448 |
|  |  | GPR39 | -2.449166456 |
|  |  | GPR50 | 7.624657447 |
|  |  | GPR63 | 1.206920788 |
|  |  | GPR78 | -3.823970692 |
|  |  | GPR85 | 4.441227566 |
|  |  | GPRC5A | -2.009455483 |
|  |  | GPRC5B | -1.410132759 |
|  |  | GPRIN1 | -1.071477879 |
|  |  | GPRIN2 | -1.225678316 |
|  |  | GPS2 | -1.057196512 |
|  |  | GPSM1 | -1.0055044 |
|  |  | GPSM2 | 1.11691752 |
|  |  | GPT2 | -1.015929147 |
|  |  | GPX2 | -3.635787902 |
|  |  | GRAMD1A | -1.190858626 |
|  |  | GRAMD1B | 2.071067778 |
|  |  | GRAMD4P8 | 6.911252059 |
|  |  | GRB10 | -1.377016652 |
|  |  | GRB14 | -3.537257788 |
|  |  | GRB7 | -2.960263395 |
|  |  | GREB1 | 1.849126099 |
|  |  | GREM1 | -3.643800578 |
|  |  | GREM2 | 5.281140778 |
|  |  | GRIA4 | 2.836980801 |
|  |  | GRID2 | 4.046281321 |
|  |  | GRIK1 | 3.050411789 |
|  |  | GRIK2 | -5.135412508 |
|  |  | GRIN1 | -2.542951014 |
|  |  | GRIN2B | -4.399202197 |
|  |  | GRIN2C | -2.145438394 |
|  |  | GRIN2D | -1.461247597 |
|  |  | GRIP2 | -1.886799381 |
|  |  | GRK5 | 3.419204825 |
|  |  | GRM1 | 3.495953719 |
|  |  | GRM7 | 6.23375881 |
|  |  | GRM8 | 5.792988814 |
|  |  | GRTP1 | 1.000032708 |
|  |  | GSC | -5.035399225 |
|  |  | GSDMC | 5.463895158 |
|  |  | GSDME | -2.347491238 |
|  |  | GSEC | 1.107515549 |
|  |  | GSN | -1.111112764 |
|  |  | GSR | 1.050814964 |
|  |  | GSTA4 | 1.260413483 |
|  |  | GSTM2 | 2.280296219 |
|  |  | GSTM4 | 2.236021185 |
|  |  | GSTO2 | 1.533304514 |
|  |  | GTF2IP23 | -1.561470024 |
|  |  | GTF2IP7 | -2.517697656 |
|  |  | GTPBP2 | -1.346636505 |
|  |  | GTPBP4 | -1.06776405 |
|  |  | GTSE1 | 1.524347546 |
|  |  | GTSE1-DT | 2.370045999 |
|  |  | GUCY1A1 | 7.287057644 |
|  |  | GUCY1B1 | 1.446404194 |
|  |  | GXYLT2 | 1.714818295 |
|  |  | GZMM | -2.237990745 |
|  |  | H1FX-AS1 | 1.64766163 |
|  |  | H2AFZ | 1.170197363 |
|  |  | H3F3A | 1.153141653 |
|  |  | H3F3AP4 | 1.27883446 |
|  |  | H3F3AP6 | 1.119980828 |
|  |  | H6PD | 1.249219687 |
|  |  | HABP4 | -2.019799731 |
|  |  | HACD4 | 1.266173786 |
|  |  | HADH | 1.578307176 |
|  |  | HAGLR | 2.674257891 |
|  |  | HAP1 | 2.229673101 |
|  |  | HAPLN3 | -2.175147503 |
|  |  | HARBI1 | -1.002302517 |
|  |  | HAS1 | 2.78907072 |
|  |  | HAS2 | 1.366302429 |
|  |  | HAS3 | -3.448854739 |
|  |  | HAUS6 | -1.222789619 |
|  |  | HAVCR1 | -5.350136369 |
|  |  | HBEGF | -2.635515259 |
|  |  | HBQ1 | -1.299979795 |
|  |  | HCAR2 | -2.367951001 |
|  |  | HCN1 | 5.356519795 |
|  |  | HCN4 | -1.161040635 |
|  |  | HCP5 | 4.369234941 |
|  |  | HDAC4 | 2.606331554 |
|  |  | HDAC9 | 1.057584191 |
|  |  | HDX | -2.307415348 |
|  |  | HEATR6 | -1.193606079 |
|  |  | HEG1 | 1.124859128 |
|  |  | HEIH | -1.055926779 |
|  |  | HERC5 | 1.077607225 |
|  |  | HES1 | -1.221619029 |
|  |  | HES2 | -1.592316331 |
|  |  | HES6 | -1.855682688 |
|  |  | HES7 | -1.507762882 |
|  |  | HEXA | 1.030914427 |
|  |  | HEXB | 1.651088481 |
|  |  | HEY1 | 2.189366829 |
|  |  | HEY2 | 2.528912488 |
|  |  | HEYL | -1.058934132 |
|  |  | HFE | 1.558877387 |
|  |  | HGF | 3.016951105 |
|  |  | HIC1 | -1.794966481 |
|  |  | HID1 | -2.524640118 |
|  |  | HILPDA | -1.535607089 |
|  |  | HIP1 | -1.294215308 |
|  |  | HIP1R | -1.876420258 |
|  |  | HIST1H2AI | 1.437173976 |
|  |  | HIST1H2BK | 1.410905804 |
|  |  | HIST1H2BO | 2.422249399 |
|  |  | HIST1H3H | 2.064690932 |
|  |  | HIST1H4H | 1.304716054 |
|  |  | HIST1H4I | 1.542323604 |
|  |  | HK2 | 1.008230752 |
|  |  | HKDC1 | -5.468626409 |
|  |  | HLA-A | 1.41341881 |
|  |  | HLA-B | 3.774410802 |
|  |  | HLA-C | 1.475500346 |
|  |  | HLA-DMA | 4.074467891 |
|  |  | HLA-DMB | 3.819974924 |
|  |  | HLA-DOA | 9.112066972 |
|  |  | HLA-DPA1 | 9.781533665 |
|  |  | HLA-DPB1 | 8.080666902 |
|  |  | HLA-DQA1 | 4.612068426 |
|  |  | HLA-DQA2 | 5.18843191 |
|  |  | HLA-DQB1 | 5.578582999 |
|  |  | HLA-DQB2 | 3.786994961 |
|  |  | HLA-DRA | 9.416344817 |
|  |  | HLA-DRB1 | 8.304098484 |
|  |  | HLA-DRB5 | 6.949564432 |
|  |  | HLA-F | 2.011032468 |
|  |  | HLA-F-AS1 | 1.562935757 |
|  |  | HLA-W | 3.033591692 |
|  |  | HLX | -1.177778179 |
|  |  | HMCN1 | 4.485953867 |
|  |  | HMGA1 | -2.190678144 |
|  |  | HMGA1P3 | -4.514770969 |
|  |  | HMGA2 | -2.672440334 |
|  |  | HMGB1 | 1.397231319 |
|  |  | HMGB1P10 | 1.189231228 |
|  |  | HMGB1P5 | 1.361055152 |
|  |  | HMGB1P6 | 1.239351045 |
|  |  | HMGB2 | 1.604048299 |
|  |  | HMGB2P1 | -5.169875783 |
|  |  | HMGB3 | 1.379404786 |
|  |  | HMGB3P32 | -5.031097897 |
|  |  | HMGN2 | 1.074146809 |
|  |  | HMGN3 | 1.242805179 |
|  |  | HMGN5 | 3.054596232 |
|  |  | HMMR | 1.718030875 |
|  |  | HNF1A | -4.188754781 |
|  |  | HNF1A-AS1 | -6.018295082 |
|  |  | HNF1B | -3.624443632 |
|  |  | HNF4A | -6.242420383 |
|  |  | HNF4G | -1.328726955 |
|  |  | HNRNPA1P33 | -1.567087331 |
|  |  | HNRNPLP2 | 1.584145956 |
|  |  | HOMEZ | 1.081546406 |
|  |  | HOOK1 | 1.589396818 |
|  |  | HOXA13 | 1.284174775 |
|  |  | HOXA5 | 2.095689794 |
|  |  | HOXB13 | -1.327737696 |
|  |  | HOXB2 | -1.341626339 |
|  |  | HOXB-AS4 | -1.273050692 |
|  |  | HOXD11 | 3.338844793 |
|  |  | HOXD8 | 1.958000961 |
|  |  | HPD | 1.420260755 |
|  |  | HPGD | -6.188743847 |
|  |  | HPN | -4.718017369 |
|  |  | HPSE | 1.355423704 |
|  |  | HPSE2 | 5.942673039 |
|  |  | HPX | -1.408161514 |
|  |  | HR | 3.694091482 |
|  |  | HRASLS | 2.400655907 |
|  |  | HRH1 | -1.486788058 |
|  |  | HS3ST3B1 | 2.378622132 |
|  |  | HS3ST4 | 6.078078425 |
|  |  | HS3ST6 | -2.827100152 |
|  |  | HS6ST1 | 2.986641958 |
|  |  | HS6ST2 | 2.333349553 |
|  |  | HS6ST3 | 9.018970066 |
|  |  | HSD17B11 | 1.121459636 |
|  |  | HSD17B13 | 2.978325806 |
|  |  | HSD17B2 | 6.715313206 |
|  |  | HSD3B7 | -1.052123068 |
|  |  | HSD3BP5 | -1.863670391 |
|  |  | HSP90B1 | 1.378059765 |
|  |  | HSPA1A | 2.906030026 |
|  |  | HSPA1B | 3.071426887 |
|  |  | HSPA2 | 3.570879626 |
|  |  | HSPA8 | 1.711275864 |
|  |  | HSPB8 | 1.643675931 |
|  |  | HSPG2 | -1.418126638 |
|  |  | HTATIP2 | -2.053159679 |
|  |  | HTR1D | -1.3066233 |
|  |  | HTR2A | 4.33417986 |
|  |  | HTR7 | -1.611033822 |
|  |  | HTRA1 | 1.222644433 |
|  |  | HTRA4 | 3.01800704 |
|  |  | HYAL1 | 2.627581339 |
|  |  | ICAM5 | -1.360002883 |
|  |  | ID2-AS1 | -1.786173075 |
|  |  | ID4 | 2.118775157 |
|  |  | IDI1 | 1.108492387 |
|  |  | IDNK | -1.319075592 |
|  |  | IER2 | -1.142754178 |
|  |  | IER3 | -1.351118213 |
|  |  | IER5 | -1.156753776 |
|  |  | IER5L | 1.052170967 |
|  |  | IFFO1 | -1.986164927 |
|  |  | IFFO2 | -1.395101994 |
|  |  | IFI16 | 2.756492048 |
|  |  | IFI44 | 3.037473791 |
|  |  | IFI44L | 6.223054086 |
|  |  | IFI6 | 1.251890112 |
|  |  | IFIH1 | 1.173526333 |
|  |  | IFIT1 | 2.698023081 |
|  |  | IFITM1 | 6.817364281 |
|  |  | IFITM10 | -1.088610803 |
|  |  | IFITM2 | 3.764972832 |
|  |  | IFITM3 | 4.576990016 |
|  |  | IFNLR1 | -2.76864823 |
|  |  | IFNWP19 | -1.050086936 |
|  |  | IFRD1 | -1.067460597 |
|  |  | IFT22 | -1.029718961 |
|  |  | IFT81 | -1.325026665 |
|  |  | IGDCC3 | 2.520886809 |
|  |  | IGDCC4 | -1.632378966 |
|  |  | IGF2BP1 | -1.006359653 |
|  |  | IGF2BP2 | -1.049920451 |
|  |  | IGFBP1 | -5.423648022 |
|  |  | IGFBP5 | 8.573433697 |
|  |  | IGFBP6 | -2.712055148 |
|  |  | IGFBP7 | 6.0367239 |
|  |  | IGFBPL1 | -1.429421651 |
|  |  | IGFL1P1 | -3.082835875 |
|  |  | IGFL2-AS1 | -3.000177803 |
|  |  | IGFL4 | 1.329030224 |
|  |  | IGFN1 | -3.215453494 |
|  |  | IGIP | 1.112435924 |
|  |  | IGSF10 | 4.35794683 |
|  |  | IGSF11 | 2.567105553 |
|  |  | IGSF5 | -4.794995476 |
|  |  | IKZF1 | 4.427381755 |
|  |  | IL10RB | -1.89096514 |
|  |  | IL11RA | 1.446466932 |
|  |  | IL13RA2 | 4.740934061 |
|  |  | IL16 | 2.252633663 |
|  |  | IL17RC | 1.291184734 |
|  |  | IL17RE | 1.377254773 |
|  |  | IL18 | -2.719571392 |
|  |  | IL18BP | -1.342652568 |
|  |  | IL18R1 | 1.573389747 |
|  |  | IL1R1 | 2.05730234 |
|  |  | IL1RAPL1 | 6.676546084 |
|  |  | IL20RA | 4.624279402 |
|  |  | IL20RB | -1.010141889 |
|  |  | IL21R | 1.450201518 |
|  |  | IL32 | -5.913923784 |
|  |  | IL33 | 4.871797666 |
|  |  | IL6 | 1.954971899 |
|  |  | IL6R | 1.088418506 |
|  |  | IL6ST | 1.251983894 |
|  |  | IL7 | 2.053220626 |
|  |  | IL7R | 4.864739091 |
|  |  | IL9RP3 | -1.427527405 |
|  |  | ILDR2 | 3.720674839 |
|  |  | ILF3-DT | 1.254692598 |
|  |  | IMMP2L | -1.204796525 |
|  |  | IMPA2 | 2.345922429 |
|  |  | IMPDH1 | -1.454797728 |
|  |  | INA | -2.674996466 |
|  |  | INAVA | -3.755088661 |
|  |  | INHBA | 4.564047534 |
|  |  | INHBB | -3.675635218 |
|  |  | INHBE | -1.784735687 |
|  |  | INKA1 | -5.098346312 |
|  |  | INSIG1 | 1.558922564 |
|  |  | INSL4 | 2.507930194 |
|  |  | INSRR | 2.690698994 |
|  |  | INSYN1 | 1.830698613 |
|  |  | INSYN2B | -3.220007054 |
|  |  | INTS6-AS1 | 1.053214757 |
|  |  | INTS6L | 2.399386664 |
|  |  | IQANK1 | -1.108698674 |
|  |  | IQCD | -1.622293259 |
|  |  | IQCE | -1.092368014 |
|  |  | IQCN | -1.222271531 |
|  |  | IQGAP2 | 2.751310404 |
|  |  | IRAK2 | -1.06589545 |
|  |  | IREB2 | 1.007679528 |
|  |  | IRF5 | -2.065813075 |
|  |  | IRF7 | -2.130428896 |
|  |  | IRF9 | 1.932462315 |
|  |  | IRS1 | -1.199238277 |
|  |  | IRX3 | 2.069935681 |
|  |  | IRX6 | 3.263684798 |
|  |  | ISCA2 | 1.036817194 |
|  |  | ISL1 | 6.422433578 |
|  |  | ISL2 | -1.104912159 |
|  |  | ISLR | -2.695772954 |
|  |  | ISM1 | 3.201774685 |
|  |  | ISYNA1 | -2.715705114 |
|  |  | ITFG1 | 1.403826312 |
|  |  | ITGA1 | 3.592388347 |
|  |  | ITGA11 | 5.247777983 |
|  |  | ITGA2 | -1.026007823 |
|  |  | ITGA3 | -1.440951874 |
|  |  | ITGA4 | 3.728772932 |
|  |  | ITGA6 | -1.927056413 |
|  |  | ITGA8 | 5.479868362 |
|  |  | ITGA9 | 3.357145368 |
|  |  | ITGAM | -5.824159749 |
|  |  | ITGB1-DT | -3.711664812 |
|  |  | ITGB4 | -2.609367273 |
|  |  | ITGB6 | -2.240059804 |
|  |  | ITGB8 | 2.314761809 |
|  |  | ITIH2 | -1.569181968 |
|  |  | ITIH5 | 1.942561464 |
|  |  | ITK | 2.342277232 |
|  |  | ITM2A | 9.779944535 |
|  |  | ITM2B | 1.629700018 |
|  |  | ITPKB | 1.449450491 |
|  |  | ITPR1 | 4.275679637 |
|  |  | ITPR1-DT | 2.244811908 |
|  |  | ITPR2 | 1.079430336 |
|  |  | ITPR3 | -1.647504228 |
|  |  | ITPRIP | 1.432857363 |
|  |  | ITSN1 | 1.008241962 |
|  |  | JAG1 | -1.028761039 |
|  |  | JAG2 | -1.531094743 |
|  |  | JAK3 | -1.052876866 |
|  |  | JAKMIP2 | 7.675252826 |
|  |  | JAKMIP3 | 1.402762655 |
|  |  | JAM2 | 2.501438619 |
|  |  | JAZF1 | 2.124544412 |
|  |  | JMJD4 | -1.270313629 |
|  |  | JPH1 | 5.404163338 |
|  |  | JPH2 | -1.911559389 |
|  |  | JPH3 | 1.182684226 |
|  |  | JUN | -3.785138941 |
|  |  | JUNB | -1.475531846 |
|  |  | JUP | 2.704089061 |
|  |  | KAAG1 | -6.101710027 |
|  |  | KALRN | 2.906179089 |
|  |  | KANK1 | 1.684437495 |
|  |  | KANK2 | 1.691125092 |
|  |  | KANK3 | -1.766377915 |
|  |  | KANK4 | 5.853798054 |
|  |  | KAT6B | 1.199161694 |
|  |  | KATNAL2 | 1.030827252 |
|  |  | KATNB1 | -1.338832035 |
|  |  | KAZN | 3.530805791 |
|  |  | KBTBD7 | 1.726998739 |
|  |  | KC6 | 3.084522125 |
|  |  | KC877982.1 | 7.452417007 |
|  |  | KCNA2 | 5.659771829 |
|  |  | KCNA7 | -5.723123707 |
|  |  | KCNAB1 | 1.772623617 |
|  |  | KCNAB2 | -1.64505683 |
|  |  | KCNC1 | 1.737083209 |
|  |  | KCND2 | 8.870559093 |
|  |  | KCNE3 | 5.059296513 |
|  |  | KCNE4 | 2.759548987 |
|  |  | KCNE5 | 2.268099175 |
|  |  | KCNF1 | -1.509549523 |
|  |  | KCNG2 | 2.706045086 |
|  |  | KCNG3 | 1.119919268 |
|  |  | KCNH3 | -1.640501055 |
|  |  | KCNH4 | -2.327029123 |
|  |  | KCNH5 | 8.403554098 |
|  |  | KCNIP3 | 1.657690674 |
|  |  | KCNJ14 | -1.080391864 |
|  |  | KCNJ2 | 2.131142292 |
|  |  | KCNJ3 | 4.316950784 |
|  |  | KCNJ6 | 3.750770563 |
|  |  | KCNJ8 | 6.290822155 |
|  |  | KCNK15 | -4.996278898 |
|  |  | KCNK3 | 3.253692728 |
|  |  | KCNK5 | -1.30228521 |
|  |  | KCNK6 | -2.673817951 |
|  |  | KCNMA1 | 1.852270066 |
|  |  | KCNMB4 | 1.523087895 |
|  |  | KCNN2 | 1.870978687 |
|  |  | KCNN4 | -1.128331632 |
|  |  | KCNQ1 | -2.882759815 |
|  |  | KCNQ5 | 1.950581528 |
|  |  | KCNT1 | -4.030127579 |
|  |  | KCNT2 | 1.131274964 |
|  |  | KCNU1 | 7.896816434 |
|  |  | KCNV1 | 2.229333898 |
|  |  | KCP | -3.075758358 |
|  |  | KCTD1 | -1.276296863 |
|  |  | KCTD11 | -1.276138681 |
|  |  | KCTD12 | 1.222914548 |
|  |  | KCTD16 | 1.967920039 |
|  |  | KCTD20 | 1.439527577 |
|  |  | KCTD8 | 6.347557965 |
|  |  | KDELC1 | 1.636792758 |
|  |  | KDELC2 | 1.693174102 |
|  |  | KDM4A | -1.210152652 |
|  |  | KDR | 5.199121931 |
|  |  | KHDRBS3 | 3.921791963 |
|  |  | KIAA0040 | 4.14889923 |
|  |  | KIAA0825 | 1.484754687 |
|  |  | KIAA0895L | 1.207232181 |
|  |  | KIAA1217 | 1.723895846 |
|  |  | KIAA1324L | 4.37430604 |
|  |  | KIAA1549L | -3.124001347 |
|  |  | KIAA1755 | -5.209970114 |
|  |  | KIAA1841 | -1.054141865 |
|  |  | KIF11 | 1.014261948 |
|  |  | KIF12 | -1.909522466 |
|  |  | KIF15 | 1.497995928 |
|  |  | KIF17 | -3.102986753 |
|  |  | KIF20A | 2.897014664 |
|  |  | KIF21B | -1.395298431 |
|  |  | KIF22 | 1.045008616 |
|  |  | KIF26A | 3.078491659 |
|  |  | KIF26B | 4.894116424 |
|  |  | KIF3C | -2.720153525 |
|  |  | KIF5A | -1.652845813 |
|  |  | KIF5C | 1.368679046 |
|  |  | KIFAP3 | 1.108405648 |
|  |  | KIFC1 | 1.588730148 |
|  |  | KIT | 10.92804722 |
|  |  | KLC2 | -1.505371965 |
|  |  | KLF11 | -2.407807272 |
|  |  | KLF12 | 2.123652963 |
|  |  | KLF16 | -1.389784637 |
|  |  | KLF2 | -2.206049082 |
|  |  | KLF2P1 | -4.091623019 |
|  |  | KLF4 | -1.105540567 |
|  |  | KLF5 | -1.511983917 |
|  |  | KLF6 | -1.340385932 |
|  |  | KLF9 | 4.299935949 |
|  |  | KLHDC3 | 1.0351665 |
|  |  | KLHDC7A | -4.893667655 |
|  |  | KLHDC9 | -1.467614181 |
|  |  | KLHL13 | 3.034946606 |
|  |  | KLHL24 | 1.430821528 |
|  |  | KLHL29 | -1.137856719 |
|  |  | KLHL3 | 1.079223799 |
|  |  | KLHL30-AS1 | -2.358609116 |
|  |  | KLHL32 | 2.476686918 |
|  |  | KLHL35 | -3.76037516 |
|  |  | KLHL4 | -6.108277386 |
|  |  | KLHL5 | -1.588868648 |
|  |  | KLK10 | 3.614684414 |
|  |  | KLK14 | -3.74836125 |
|  |  | KLK2 | -5.603159031 |
|  |  | KLLN | -1.09055945 |
|  |  | KLRA1P | 1.593495678 |
|  |  | KLRC2 | -4.802651489 |
|  |  | KMO | 1.910509647 |
|  |  | KNDC1 | 1.143018015 |
|  |  | KNL1 | 1.21721863 |
|  |  | KPNA7 | -4.263738864 |
|  |  | KRBA2 | 1.150622059 |
|  |  | KREMEN1 | 1.106383526 |
|  |  | KRT15 | -7.829512462 |
|  |  | KRT16 | -5.208461619 |
|  |  | KRT18 | -1.402833245 |
|  |  | KRT19 | -6.374193531 |
|  |  | KRT34 | -5.956482163 |
|  |  | KRT6A | -6.75897283 |
|  |  | KRT7 | -1.898665019 |
|  |  | KRT7-AS | -1.378470159 |
|  |  | KRT8 | -1.490343154 |
|  |  | KRT81 | -1.373797078 |
|  |  | KRT84 | -4.254175938 |
|  |  | KRT86 | -1.270027698 |
|  |  | KRT87P | -1.269642125 |
|  |  | KRT8P30 | 3.08853155 |
|  |  | KRTAP5-AS1 | -3.331146499 |
|  |  | KSR1 | 2.87238049 |
|  |  | KSR2 | -3.017237025 |
|  |  | L1CAM | 3.744359265 |
|  |  | L3MBTL4 | 4.349726391 |
|  |  | LAG3 | 1.102600791 |
|  |  | LAIR1 | 7.412919672 |
|  |  | LAMA1 | 7.057264279 |
|  |  | LAMA4 | 5.30644409 |
|  |  | LAMA5-AS1 | -1.902939049 |
|  |  | LAMB2 | 1.817498227 |
|  |  | LAMC2 | -5.130025034 |
|  |  | LAMP1 | 1.503098448 |
|  |  | LAMP2 | 1.258685285 |
|  |  | LANCL1 | 1.46616747 |
|  |  | LANCL1-AS1 | 1.298173456 |
|  |  | LANCL3 | 5.693308784 |
|  |  | LAPTM4A | 1.249979031 |
|  |  | LARP6 | -1.509865619 |
|  |  | LAT2 | -2.35576128 |
|  |  | LBH | 1.065753187 |
|  |  | LBHD2 | -4.615990483 |
|  |  | LBR | 1.461253899 |
|  |  | LCA5L | -1.368428875 |
|  |  | LCN2 | -7.322933186 |
|  |  | LCORL | 1.264396206 |
|  |  | LCP1 | 4.677005893 |
|  |  | LDAH | 1.116456037 |
|  |  | LDB1 | 1.309963002 |
|  |  | LDB2 | 5.392063721 |
|  |  | LDHD | 1.422781871 |
|  |  | LDLR | 1.497771297 |
|  |  | LDLRAD4 | 2.568718141 |
|  |  | LEAP2 | 1.750262223 |
|  |  | LECT2 | 2.176392204 |
|  |  | LEF1 | 6.759142271 |
|  |  | LEF1-AS1 | 3.429692461 |
|  |  | LENG9 | -3.193977453 |
|  |  | LETM2 | -2.316135219 |
|  |  | LFNG | -2.962694915 |
|  |  | LGALS2 | -3.450514772 |
|  |  | LGALS3BP | 1.810731678 |
|  |  | LGALS9 | 4.109514929 |
|  |  | LGALS9B | 5.478049302 |
|  |  | LGALS9C | 2.83438168 |
|  |  | LGI2 | 1.970129541 |
|  |  | LGI4 | -4.997299489 |
|  |  | LGR5 | 3.726020131 |
|  |  | LGR6 | -3.635855916 |
|  |  | LHCGR | 6.046251977 |
|  |  | LHFPL4 | -2.756821787 |
|  |  | LHFPL5 | -4.800966348 |
|  |  | LHFPL6 | 1.938179373 |
|  |  | LHX1 | -2.104320185 |
|  |  | LHX1-DT | -2.911565195 |
|  |  | LHX8 | -2.303390943 |
|  |  | LHX9 | 1.383223886 |
|  |  | LIF | -1.238814346 |
|  |  | LIFR | 1.52737655 |
|  |  | LIMA1 | 2.716471946 |
|  |  | LIMCH1 | 1.132522666 |
|  |  | LIMD2 | -1.29214655 |
|  |  | LIMS2 | -1.659613528 |
|  |  | LIN7A | 2.312244739 |
|  |  | LINC00052 | 3.461466988 |
|  |  | LINC00294 | -1.308326206 |
|  |  | LINC00304 | -3.061469186 |
|  |  | LINC00319 | 2.04213397 |
|  |  | LINC00322 | 1.979006286 |
|  |  | LINC00355 | 5.101877764 |
|  |  | LINC00452 | 4.473878514 |
|  |  | LINC00471 | -2.132739047 |
|  |  | LINC00476 | 1.24416986 |
|  |  | LINC00482 | 1.08548572 |
|  |  | LINC00488 | 3.040953223 |
|  |  | LINC00511 | -2.249973753 |
|  |  | LINC00513 | -2.902445859 |
|  |  | LINC00519 | 6.568417687 |
|  |  | LINC00526 | 1.93624764 |
|  |  | LINC00540 | 1.685113748 |
|  |  | LINC00565 | 5.022116391 |
|  |  | LINC00570 | 3.441397669 |
|  |  | LINC00601 | 3.792355386 |
|  |  | LINC00604 | 4.70904039 |
|  |  | LINC00607 | 2.713340411 |
|  |  | LINC00623 | 1.344203396 |
|  |  | LINC00630 | -1.369209118 |
|  |  | LINC00632 | 1.831621119 |
|  |  | LINC00638 | -1.142289517 |
|  |  | LINC00648 | 4.108820866 |
|  |  | LINC00662 | -1.662676633 |
|  |  | LINC00665 | -3.152973275 |
|  |  | LINC00689 | 4.786685818 |
|  |  | LINC00842 | -1.814474357 |
|  |  | LINC00862 | -2.502718582 |
|  |  | LINC00869 | 1.637289411 |
|  |  | LINC00886 | -1.05653484 |
|  |  | LINC00887 | -1.638106301 |
|  |  | LINC00920 | 1.403773324 |
|  |  | LINC00941 | -2.779581917 |
|  |  | LINC00942 | 1.915959532 |
|  |  | LINC00968 | 4.718542156 |
|  |  | LINC00973 | -7.371510813 |
|  |  | LINC00993 | -5.314084294 |
|  |  | LINC01006 | -1.020695597 |
|  |  | LINC01012 | -1.33458506 |
|  |  | LINC01081 | 3.629024356 |
|  |  | LINC01087 | 5.461853144 |
|  |  | LINC01107 | -3.909898409 |
|  |  | LINC01116 | 2.632778589 |
|  |  | LINC01121 | 1.833474372 |
|  |  | LINC01127 | 4.884980732 |
|  |  | LINC01133 | -3.070565828 |
|  |  | LINC01139 | 1.73723307 |
|  |  | LINC01145 | 1.623860382 |
|  |  | LINC01186 | -1.768420431 |
|  |  | LINC01210 | 4.812289879 |
|  |  | LINC01249 | 6.010808485 |
|  |  | LINC01303 | 4.261959506 |
|  |  | LINC01352 | -2.604874161 |
|  |  | LINC01388 | 3.380911583 |
|  |  | LINC01433 | 2.034519793 |
|  |  | LINC01460 | -4.143231007 |
|  |  | LINC01480 | 2.846544008 |
|  |  | LINC01504 | -2.523575869 |
|  |  | LINC01529 | 1.82362727 |
|  |  | LINC01551 | 5.243257565 |
|  |  | LINC01559 | -4.626845314 |
|  |  | LINC01583 | -2.798133163 |
|  |  | LINC01588 | -1.305712455 |
|  |  | LINC01589 | -5.717733181 |
|  |  | LINC01632 | 5.177049912 |
|  |  | LINC01637 | -4.062676215 |
|  |  | LINC01669 | 2.560452985 |
|  |  | LINC01687 | 4.209621595 |
|  |  | LINC01693 | 3.283147833 |
|  |  | LINC01697 | 6.61134257 |
|  |  | LINC01778 | -2.632316483 |
|  |  | LINC01806 | -5.878762478 |
|  |  | LINC01842 | 2.218105464 |
|  |  | LINC01843 | -5.334277937 |
|  |  | LINC01909 | -2.302100673 |
|  |  | LINC01910 | -3.453932171 |
|  |  | LINC01914 | 2.994459584 |
|  |  | LINC01942 | -2.87809531 |
|  |  | LINC01952 | -5.353529804 |
|  |  | LINC01973 | 4.846942808 |
|  |  | LINC02057 | -1.646502817 |
|  |  | LINC02104 | 5.000739318 |
|  |  | LINC02138 | -4.34048626 |
|  |  | LINC02163 | 2.857490817 |
|  |  | LINC02212 | -1.960133918 |
|  |  | LINC02223 | 2.337275259 |
|  |  | LINC02249 | 4.232861157 |
|  |  | LINC02253 | 5.544866948 |
|  |  | LINC02254 | 4.49727347 |
|  |  | LINC02274 | 5.457354401 |
|  |  | LINC02289 | 3.416456854 |
|  |  | LINC02301 | 4.888143807 |
|  |  | LINC02328 | 2.747606054 |
|  |  | LINC02331 | -4.571495165 |
|  |  | LINC02395 | 5.090031074 |
|  |  | LINC02407 | -4.806335418 |
|  |  | LINC02454 | -3.842361819 |
|  |  | LINC02475 | 8.279169159 |
|  |  | LINC02532 | -2.82228324 |
|  |  | LINC02535 | -3.176398645 |
|  |  | LINC02551 | -2.282841616 |
|  |  | LINC02582 | -11.04283031 |
|  |  | LINC02593 | 1.195559177 |
|  |  | LINC02595 | -2.545528846 |
|  |  | LINC-PINT | -3.528118973 |
|  |  | LINGO2 | 1.804653501 |
|  |  | LINGO4 | 3.862632149 |
|  |  | LIPC | -5.038230152 |
|  |  | LIPE-AS1 | -2.091463298 |
|  |  | LIPH | -2.819260194 |
|  |  | LITAF | 1.32884481 |
|  |  | LLGL2 | -2.086525853 |
|  |  | LMAN2 | 1.058306781 |
|  |  | LMBRD1 | 1.680913097 |
|  |  | LMCD1 | -4.201409512 |
|  |  | LMCD1-AS1 | -3.230105647 |
|  |  | LMNB1 | 1.20632272 |
|  |  | LMNTD1 | 5.589234804 |
|  |  | LMNTD2 | -1.551305403 |
|  |  | LMO1 | 2.686435328 |
|  |  | LMO2 | 3.96219362 |
|  |  | LMO3 | 1.423640323 |
|  |  | LMO7 | -1.156268222 |
|  |  | LMOD1 | -1.370614683 |
|  |  | LNCAROD | -5.703892198 |
|  |  | LOXL1 | 2.019130132 |
|  |  | LOXL1-AS1 | -2.721030977 |
|  |  | LOXL3 | 1.544859762 |
|  |  | LPAL2 | -1.270084948 |
|  |  | LPAR4 | 4.461177635 |
|  |  | LPAR6 | 3.394412386 |
|  |  | LPCAT1 | 2.025798455 |
|  |  | LPCAT2 | 1.110162641 |
|  |  | LPIN1 | 1.365947381 |
|  |  | LPL | 8.091472288 |
|  |  | LPXN | -1.067114157 |
|  |  | LRCH1 | -1.069150757 |
|  |  | LRCH2 | 6.90532569 |
|  |  | LRFN5 | 4.403244097 |
|  |  | LRIG1 | 2.8974404 |
|  |  | LRP11 | 1.096922057 |
|  |  | LRP1B | 5.939613198 |
|  |  | LRP2 | 4.078700907 |
|  |  | LRPAP1 | 1.237652732 |
|  |  | LRRC10B | -3.831053999 |
|  |  | LRRC15 | 2.762229254 |
|  |  | LRRC17 | 3.644364193 |
|  |  | LRRC20 | -1.012760153 |
|  |  | LRRC31 | -5.795353608 |
|  |  | LRRC37A9P | 1.849202466 |
|  |  | LRRC4C | 4.571726193 |
|  |  | LRRC56 | -1.651309011 |
|  |  | LRRC63 | 3.221994156 |
|  |  | LRRC7 | 1.933307991 |
|  |  | LRRC75B | -1.152538406 |
|  |  | LRRC8C | -1.50428613 |
|  |  | LRRCC1 | 1.921674519 |
|  |  | LRRIQ1 | -2.478030757 |
|  |  | LRRK2 | -1.412090627 |
|  |  | LRRTM3 | 7.51371975 |
|  |  | LRRTM4 | 8.107024489 |
|  |  | LRSAM1 | -1.057526802 |
|  |  | LRWD1 | -1.148837158 |
|  |  | LSAMP | 6.701667544 |
|  |  | LTBP1 | 1.135763963 |
|  |  | LTO1 | -1.206503056 |
|  |  | LUM | 8.859742253 |
|  |  | LURAP1L | -1.852327529 |
|  |  | LUZP2 | 3.881459849 |
|  |  | LXN | 2.750294345 |
|  |  | LY6E | 1.332178403 |
|  |  | LY6H | 5.361296743 |
|  |  | LYPD3 | 6.962736242 |
|  |  | LYPD6 | 4.164591257 |
|  |  | LYPD6B | 10.15271994 |
|  |  | LYPD8 | 1.402469165 |
|  |  | LZTS1 | 5.301882262 |
|  |  | LZTS3 | -1.797313312 |
|  |  | MAATS1 | -5.407097455 |
|  |  | MACF1 | -1.595733924 |
|  |  | MACROD1 | -1.579191122 |
|  |  | MAD1L1 | -1.317871985 |
|  |  | MAF | 3.105164345 |
|  |  | MAFB | 6.389123662 |
|  |  | MAFF | -2.670893884 |
|  |  | MAFG-DT | -1.130895202 |
|  |  | MAFK | -1.241296765 |
|  |  | MAGEB6 | -3.425816747 |
|  |  | MAGED1 | 2.160728753 |
|  |  | MAGED2 | 1.262964436 |
|  |  | MAGED4 | 4.084763022 |
|  |  | MAGED4B | 2.168909392 |
|  |  | MAGEH1 | 2.405067036 |
|  |  | MAJIN | -4.066249992 |
|  |  | MAL2 | 6.487793696 |
|  |  | MALL | -1.78469905 |
|  |  | MALRD1 | 3.090148811 |
|  |  | MAMDC2 | 6.04373597 |
|  |  | MAML2 | 1.144177218 |
|  |  | MAN1A1 | 7.660452552 |
|  |  | MANBA | 1.3694074 |
|  |  | MANEA | 2.049033021 |
|  |  | MANF | 1.193034138 |
|  |  | MAOA | 1.510455719 |
|  |  | MAP1B | -1.616245111 |
|  |  | MAP1LC3A | -4.242233417 |
|  |  | MAP2 | 4.849152497 |
|  |  | MAP3K10 | -1.137153462 |
|  |  | MAP3K14 | -1.355903877 |
|  |  | MAP3K15 | -1.206746879 |
|  |  | MAP3K7CL | -1.360582837 |
|  |  | MAP3K9 | -1.224336942 |
|  |  | MAP6 | 1.356467254 |
|  |  | MAP7 | 1.031813038 |
|  |  | MAP7D2 | -2.561234871 |
|  |  | MAPK15 | -1.911939136 |
|  |  | MAPK4 | 7.186394819 |
|  |  | MAPK8IP1 | -1.510933891 |
|  |  | MAPRE2 | 1.282019938 |
|  |  | MAPT | 3.144542116 |
|  |  | MARCOL | 7.18947186 |
|  |  | MARK1 | 1.942907817 |
|  |  | MAST2 | -1.131394431 |
|  |  | MAST4 | 1.884174107 |
|  |  | MAT1A | 2.482076567 |
|  |  | MATN1-AS1 | -2.975654013 |
|  |  | MBL1P | 5.565053278 |
|  |  | MBLAC1 | -1.047446194 |
|  |  | MBNL3 | 7.755149434 |
|  |  | MBOAT2 | 1.366187259 |
|  |  | MBP | -1.285257894 |
|  |  | MCAM | 3.235583088 |
|  |  | MCC | 4.162111077 |
|  |  | MCF2 | 5.525174797 |
|  |  | MCF2L | 1.867757113 |
|  |  | MCIDAS | -1.051944239 |
|  |  | MCL1 | -1.263469766 |
|  |  | MCOLN3 | 1.805489874 |
|  |  | MCTP2 | 8.891741765 |
|  |  | MCUB | -1.174478109 |
|  |  | MDGA1 | 1.504531318 |
|  |  | MDGA2 | 4.92658673 |
|  |  | MECOM | 6.174544866 |
|  |  | MED10 | -1.539425442 |
|  |  | MED12 | 1.118479803 |
|  |  | MED12L | 1.186212502 |
|  |  | MED13L | -1.207955233 |
|  |  | MED15P6 | 4.123927293 |
|  |  | MED17 | -1.375105135 |
|  |  | MED28 | 1.006117695 |
|  |  | MEDAG | 6.890176961 |
|  |  | MEF2C | 7.981880937 |
|  |  | MEF2C-AS1 | 2.838406977 |
|  |  | MEG3 | 1.055133033 |
|  |  | MEG9 | 1.112680088 |
|  |  | MEGF11 | -2.362248156 |
|  |  | MEGF6 | -4.915832673 |
|  |  | MEGF8 | 1.402711901 |
|  |  | MEGF9 | 1.562669049 |
|  |  | MEIOB | -1.476520883 |
|  |  | MEIS1 | 2.918892315 |
|  |  | MELTF | -1.550858762 |
|  |  | MEMO1P1 | 2.287639102 |
|  |  | MEOX2 | 4.676751544 |
|  |  | MESP1 | -1.833436037 |
|  |  | MEST | 1.213573666 |
|  |  | MET | -1.700807144 |
|  |  | METRNL | 1.669156581 |
|  |  | METTL15P1 | -1.930389098 |
|  |  | METTL27 | -1.381321581 |
|  |  | METTL7A | 8.071980494 |
|  |  | MEX3B | 1.032643844 |
|  |  | MFGE8 | -1.061062269 |
|  |  | MFHAS1 | -1.723143698 |
|  |  | MFNG | -3.29608117 |
|  |  | MFSD12 | 1.015438535 |
|  |  | MFSD2A | 3.16769454 |
|  |  | MFSD2B | -1.692148976 |
|  |  | MFSD4A | 2.305623273 |
|  |  | MGAT4A | 1.790734863 |
|  |  | MGAT4C | 1.992743303 |
|  |  | MGAT5B | 2.088372352 |
|  |  | MGLL | -2.37151975 |
|  |  | MGP | 11.23588451 |
|  |  | MGST2 | 1.539701843 |
|  |  | MGST3 | 2.059204737 |
|  |  | MICAL2 | -4.045598623 |
|  |  | MICALL1 | -1.160950371 |
|  |  | MICD | 1.768969276 |
|  |  | MID1 | -2.460103143 |
|  |  | MID1IP1 | -1.019592552 |
|  |  | MIF4GD | -1.24483311 |
|  |  | MIIP | -1.412284688 |
|  |  | MILR1 | 1.350188417 |
|  |  | MIOS | -1.027330794 |
|  |  | MIR1244-2 | 1.697824288 |
|  |  | MIR137HG | -3.19994901 |
|  |  | MIR210HG | 2.442004983 |
|  |  | MIR221 | -5.843370246 |
|  |  | MIR222HG | -1.630211085 |
|  |  | MIR29B2CHG | 1.355303208 |
|  |  | MIR3189 | -3.002361733 |
|  |  | MIR3936HG | -1.28047009 |
|  |  | MIR4453HG | 1.223441126 |
|  |  | MIR4458HG | 1.068466564 |
|  |  | MIR4500HG | 1.672823963 |
|  |  | MIR503HG | -1.651477139 |
|  |  | MIR548XHG | 6.612409845 |
|  |  | MIR600HG | 1.608256012 |
|  |  | MIR616 | -2.253266997 |
|  |  | MIR6775 | -2.970899189 |
|  |  | MIR6784 | -3.013732709 |
|  |  | MIR99AHG | 2.021875247 |
|  |  | MIRLET7BHG | -1.098476174 |
|  |  | MISP | -1.979695479 |
|  |  | MKI67 | 1.772894597 |
|  |  | MKLN1-AS | -1.398512878 |
|  |  | MKX | 5.460353278 |
|  |  | MLPH | -3.036611303 |
|  |  | MLXIPL | -2.075899013 |
|  |  | MME | 9.723098264 |
|  |  | MME-AS1 | 5.413463728 |
|  |  | MMP1 | -3.989477963 |
|  |  | MMP16 | 2.677140373 |
|  |  | MMP19 | 1.501514458 |
|  |  | MMP2 | 5.298332504 |
|  |  | MMP24 | -3.591332196 |
|  |  | MMP24OS | -2.221003106 |
|  |  | MMP7 | -5.959808271 |
|  |  | MMP9 | 3.488291917 |
|  |  | MND1 | 1.155402156 |
|  |  | MNX1 | -2.183222606 |
|  |  | MNX1-AS1 | -1.517802189 |
|  |  | MOCOS | 1.257978546 |
|  |  | MOK | -2.372577763 |
|  |  | MORC4 | -1.111834976 |
|  |  | MORF4L2-AS1 | 1.482181691 |
|  |  | MORN4 | -1.804221923 |
|  |  | MOXD1 | 1.05334542 |
|  |  | MPC1 | 2.145116897 |
|  |  | MPP1 | 1.132308847 |
|  |  | MPP2 | -1.641526847 |
|  |  | MPP3 | -1.366492153 |
|  |  | MPP7 | 4.666745521 |
|  |  | MPPED2 | 7.716679879 |
|  |  | MPV17L | -1.327740789 |
|  |  | MPZL2 | 2.880074875 |
|  |  | MRC2 | 1.543879643 |
|  |  | MRO | 3.796409826 |
|  |  | MROH3P | -3.032732397 |
|  |  | MRPL12 | -1.271527867 |
|  |  | MRPL45P2 | -1.116850912 |
|  |  | MRPS30-DT | 2.497303979 |
|  |  | MRTFA | -1.862512835 |
|  |  | MRTFA-AS1 | -3.523589455 |
|  |  | MSI2 | 1.378568605 |
|  |  | MSRB2 | 1.163060087 |
|  |  | MST1 | 1.010250066 |
|  |  | MST1R | -4.451225504 |
|  |  | MSX1 | 2.043715535 |
|  |  | MT1F | -2.198725824 |
|  |  | MT1X | 1.417227295 |
|  |  | MT1XP1 | -5.645056744 |
|  |  | MT2P1 | -2.057634771 |
|  |  | MTATP6P1 | 1.800146769 |
|  |  | MTCL1 | -2.544449951 |
|  |  | MTHFD2 | -1.082698593 |
|  |  | MTND1P23 | 1.45941713 |
|  |  | MT-ND5 | 1.309619568 |
|  |  | MT-ND6 | 1.355983794 |
|  |  | MT-RNR1 | 1.801062696 |
|  |  | MTRNR2L4 | -3.271072196 |
|  |  | MT-TG | 1.636128235 |
|  |  | MT-TH | 1.24859421 |
|  |  | MT-TR | 1.021408105 |
|  |  | MT-TT | 1.634339732 |
|  |  | MTUS2 | 3.604028766 |
|  |  | MUC1 | 1.070150694 |
|  |  | MUC12 | -2.408624473 |
|  |  | MUC13 | -1.543933401 |
|  |  | MUC19 | -3.504035316 |
|  |  | MUC20-OT1 | 1.060257023 |
|  |  | MUC20P1 | 1.088201203 |
|  |  | MUC3A | -3.636738636 |
|  |  | MUC5AC | -8.063678556 |
|  |  | MUC5B | -7.950279258 |
|  |  | MUC6 | -1.51837883 |
|  |  | MUM1L1 | 7.82733606 |
|  |  | MUS81 | -1.234641956 |
|  |  | MXD1 | -1.647836427 |
|  |  | MXD3 | 1.503137461 |
|  |  | MYADML2 | -1.543332931 |
|  |  | MYBBP1A | -1.023528819 |
|  |  | MYBPC3 | -3.03223447 |
|  |  | MYC | -1.49703138 |
|  |  | MYCBPAP | -1.757477415 |
|  |  | MYCL | 1.466473421 |
|  |  | MYCN | 5.748770541 |
|  |  | MYEOV | -7.168429621 |
|  |  | MYH11 | -1.390760096 |
|  |  | MYH14 | -1.098506812 |
|  |  | MYH16 | -1.86703007 |
|  |  | MYH3 | -1.101985859 |
|  |  | MYH9 | -1.220113726 |
|  |  | MYL5 | -1.188186404 |
|  |  | MYLIP | 1.564329323 |
|  |  | MYLK | -1.412665771 |
|  |  | MYLK3 | 4.597965444 |
|  |  | MYO15B | -2.009552991 |
|  |  | MYO1C | -1.615081265 |
|  |  | MYO1D | 1.406480597 |
|  |  | MYO1E | -2.551800465 |
|  |  | MYO3A | 3.120272086 |
|  |  | MYO7A | -1.759578869 |
|  |  | MYOCD | -1.997182775 |
|  |  | MYOF | -1.459223251 |
|  |  | MYOM3 | -1.714721151 |
|  |  | MYRF | -3.179490347 |
|  |  | MYRIP | 1.694537155 |
|  |  | NAALAD2 | 3.705179591 |
|  |  | NAALADL2 | 2.728580296 |
|  |  | NALCN | -1.763154567 |
|  |  | NAMPT | 1.30979588 |
|  |  | NAMPTP1 | 1.105358105 |
|  |  | NANOS1 | 1.669150993 |
|  |  | NAP1L2 | 2.59021248 |
|  |  | NAP1L3 | 2.592247299 |
|  |  | NAP1L5 | 1.934996676 |
|  |  | NAT16 | 2.632133322 |
|  |  | NAT8L | -1.188670651 |
|  |  | NAT9 | -1.286181704 |
|  |  | NAV1 | 1.475803627 |
|  |  | NAV3 | -1.686344148 |
|  |  | NBL1 | -2.503032211 |
|  |  | NBPF1 | -1.044827774 |
|  |  | NBPF21P | -4.21199476 |
|  |  | NBPF4 | 2.069097034 |
|  |  | NBR2 | -1.156279195 |
|  |  | NCALD | 2.677050459 |
|  |  | NCAM1 | 6.909299643 |
|  |  | NCAM2 | 8.348130155 |
|  |  | NCAPD2 | 1.087107387 |
|  |  | NCAPG | 1.045466588 |
|  |  | NCAPH | 1.17023752 |
|  |  | NCEH1 | -1.209628068 |
|  |  | NCKAP5 | 1.356182658 |
|  |  | NCOA1 | 1.018588538 |
|  |  | NCS1 | -1.161211497 |
|  |  | NCSTN | 1.398118604 |
|  |  | NDNF | 7.677334354 |
|  |  | NDRG2 | 3.451220558 |
|  |  | NDRG4 | -1.67874644 |
|  |  | NDST3 | 5.665139684 |
|  |  | NDST4 | 5.632188977 |
|  |  | NDUFA4L2 | -1.921093963 |
|  |  | NDUFAF8 | -1.165392465 |
|  |  | NDUFB4 | 1.484880795 |
|  |  | NDUFB5 | 1.109362072 |
|  |  | NECTIN2 | 1.677585786 |
|  |  | NECTIN4 | -2.103064312 |
|  |  | NEDD4L | -1.910805767 |
|  |  | NEFL | 3.008214621 |
|  |  | NEFM | 1.986041018 |
|  |  | NEGR1 | 9.065558441 |
|  |  | NEK10 | -1.900835672 |
|  |  | NEK11 | -1.559425818 |
|  |  | NEK2 | 1.42478306 |
|  |  | NELL2 | 4.168014875 |
|  |  | NEMP2 | 1.438813447 |
|  |  | NES | 2.539270113 |
|  |  | NETO1 | 4.637862444 |
|  |  | NEU1 | 1.07333718 |
|  |  | NEU4 | -1.348986828 |
|  |  | NEURL1 | -2.53935466 |
|  |  | NEURL1B | 3.525722441 |
|  |  | NEURL3 | -6.104950694 |
|  |  | NEUROG2 | 4.399147359 |
|  |  | NEXN | -1.648312152 |
|  |  | NEXN-AS1 | -3.613849372 |
|  |  | NFASC | -1.097003491 |
|  |  | NFATC4 | 8.846424739 |
|  |  | NFE2 | 2.472788234 |
|  |  | NFKB2 | -2.38988759 |
|  |  | NFKBIB | -1.132329317 |
|  |  | NFKBIE | -1.415199421 |
|  |  | NGEF | -3.917596402 |
|  |  | NGF | -3.366212916 |
|  |  | NHSL2 | 1.792123251 |
|  |  | NID1 | 2.512182367 |
|  |  | NID2 | 2.86487091 |
|  |  | NINJ2 | 2.107179427 |
|  |  | NIPAL2 | 1.442553403 |
|  |  | NIPAL4 | -3.568934673 |
|  |  | NIPBL-DT | 1.662425352 |
|  |  | NKAIN2 | 2.455233363 |
|  |  | NKAIN3 | 1.409460268 |
|  |  | NKAIN3-IT1 | 2.96542938 |
|  |  | NKAIN4 | -1.77257257 |
|  |  | NKILA | -1.362093452 |
|  |  | NKPD1 | -1.898434832 |
|  |  | NKX2-1 | 4.952324883 |
|  |  | NKX2-2 | 3.287277634 |
|  |  | NKX2-4 | 4.927975192 |
|  |  | NKX2-5 | -1.137645907 |
|  |  | NKX2-8 | -1.733946578 |
|  |  | NKX3-2 | 4.90641242 |
|  |  | NKX6-1 | 2.708764659 |
|  |  | NLE1 | -1.338416149 |
|  |  | NLGN1 | 4.801084351 |
|  |  | NLGN3 | -1.210728782 |
|  |  | NLGN4X | 7.656184141 |
|  |  | NLGN4Y | -1.424056706 |
|  |  | NLRC3 | -1.007582013 |
|  |  | NLRC4 | -1.184031334 |
|  |  | NLRC5 | 1.033450689 |
|  |  | NLRP1 | 1.505464088 |
|  |  | NLRP12 | -4.623866959 |
|  |  | NLRP9 | -4.185835487 |
|  |  | NME2 | -1.338101525 |
|  |  | NME3 | -1.52357222 |
|  |  | NMRAL1 | -1.00352418 |
|  |  | NMRAL2P | 1.196633554 |
|  |  | NMRK1 | 1.250490304 |
|  |  | NMRK2 | -5.016119469 |
|  |  | NMU | 8.290802606 |
|  |  | NNAT | 1.305280758 |
|  |  | NNMT | 1.211261586 |
|  |  | NNT | 1.528353146 |
|  |  | NOCT | -2.022782915 |
|  |  | NOG | -2.073806259 |
|  |  | NOL4L | 1.124473907 |
|  |  | NOP14-AS1 | -1.204879417 |
|  |  | NOS1 | 5.45187315 |
|  |  | NOS2 | -4.275794718 |
|  |  | NOS2P3 | 2.05485794 |
|  |  | NOTCH3 | 5.346979368 |
|  |  | NOV | 4.251134962 |
|  |  | NOX5 | 2.53283441 |
|  |  | NPAS1 | -1.656700622 |
|  |  | NPAS2 | -1.297173006 |
|  |  | NPAS3 | 4.610196702 |
|  |  | NPBWR1 | 1.339136516 |
|  |  | NPC1 | -1.015016545 |
|  |  | NPC2 | 1.323693041 |
|  |  | NPHP1 | -1.000909309 |
|  |  | NPHS1 | -6.138049612 |
|  |  | NPL | 2.224667031 |
|  |  | NPNT | 6.404156619 |
|  |  | NPR1 | 2.9199164 |
|  |  | NPR3 | 1.318729972 |
|  |  | NPTN | 1.3735452 |
|  |  | NPTX1 | 4.681517845 |
|  |  | NPY | 4.271082968 |
|  |  | NPY1R | 5.157698134 |
|  |  | NPY2R | 4.229914404 |
|  |  | NPY5R | 3.546586991 |
|  |  | NPY6R | 3.387822651 |
|  |  | NQO1 | 1.672323296 |
|  |  | NR0B1 | 1.413788283 |
|  |  | NR2E3 | -1.961563322 |
|  |  | NR3C1 | -1.136910864 |
|  |  | NR3C2 | 2.793986916 |
|  |  | NR4A2 | 1.97453499 |
|  |  | NR4A3 | 1.971762908 |
|  |  | NR5A2 | -5.769865859 |
|  |  | NREP | 1.908467812 |
|  |  | NRG1 | -2.407868959 |
|  |  | NRG4 | 2.880458866 |
|  |  | NRGN | 1.116622749 |
|  |  | NRK | 3.637556044 |
|  |  | NRM | 1.050862614 |
|  |  | NRN1 | 2.449035541 |
|  |  | NRP1 | -1.11349716 |
|  |  | NRSN1 | -4.704207967 |
|  |  | NRXN3 | 1.535832166 |
|  |  | NSUN7 | -4.139156321 |
|  |  | NT5C2 | -1.068748918 |
|  |  | NT5DC2 | 1.574362199 |
|  |  | NT5DC3 | -1.240563662 |
|  |  | NTF4 | -2.240221833 |
|  |  | NTN4 | -2.945763622 |
|  |  | NTNG1 | 3.88149082 |
|  |  | NTNG2 | 2.055048252 |
|  |  | NTRK1 | 2.010783821 |
|  |  | NTRK2 | 8.86723078 |
|  |  | NTRK3 | -1.252767711 |
|  |  | NTS | -1.349033478 |
|  |  | NUAK2 | -1.460034354 |
|  |  | NUCB1 | 1.543839888 |
|  |  | NUCB2 | 1.322431161 |
|  |  | NUDC | -1.098845752 |
|  |  | NUDT10 | 1.320534003 |
|  |  | NUDT11 | 1.47049686 |
|  |  | NUDT16P1 | -4.792050318 |
|  |  | NUDT7 | 1.619418081 |
|  |  | NUF2 | 1.884339656 |
|  |  | NUGGC | 2.859451534 |
|  |  | NUP62CL | -1.469204614 |
|  |  | NUPR1 | 1.280223256 |
|  |  | NUSAP1 | 1.985874624 |
|  |  | NWD1 | 2.546433048 |
|  |  | NXF3 | 4.196161956 |
|  |  | NXPE3 | 1.09821709 |
|  |  | NXPH2 | 4.440097856 |
|  |  | NXT1 | -1.321724254 |
|  |  | NYAP2 | 3.601139281 |
|  |  | NYNRIN | 7.283831807 |
|  |  | OBSCN-AS1 | 1.961910073 |
|  |  | OBSL1 | 1.552486587 |
|  |  | OCIAD2 | -1.1337047 |
|  |  | OCLN | -3.447271299 |
|  |  | ODF3B | 1.019086655 |
|  |  | ODF3L1 | 3.242462699 |
|  |  | OFD1P2Y | -4.60529943 |
|  |  | OGDHL | 3.555605897 |
|  |  | OGFRP1 | -1.381563269 |
|  |  | OGN | 3.921079337 |
|  |  | OLFM1 | 7.914787518 |
|  |  | OLFM2 | 1.35879416 |
|  |  | OLFM3 | 6.393739082 |
|  |  | OLFML1 | 3.67122519 |
|  |  | OLFML2B | 1.915236048 |
|  |  | OLIG3 | 5.971502526 |
|  |  | ONECUT3 | 4.681027066 |
|  |  | OPRK1 | 4.232351329 |
|  |  | OPRL1 | -1.236218497 |
|  |  | OPTN | -1.417768516 |
|  |  | OR2B6 | 1.824098787 |
|  |  | OR2T4 | 5.965869778 |
|  |  | OR51E2 | 4.719220503 |
|  |  | OR7E14P | -3.223777518 |
|  |  | ORAI2 | -1.199823933 |
|  |  | OSBP2 | -1.243134888 |
|  |  | OSBPL10 | 1.196363553 |
|  |  | OSCAR | -1.144685177 |
|  |  | OSCP1 | -1.07199896 |
|  |  | OSR1 | 1.15937228 |
|  |  | OSR2 | 1.889126606 |
|  |  | OSTC | 1.27733018 |
|  |  | OSTM1 | 1.143697025 |
|  |  | OTOF | 2.118527798 |
|  |  | OTUD6B-AS1 | 1.030405776 |
|  |  | OTULINL | 1.256722603 |
|  |  | OVCH1 | 3.743401286 |
|  |  | OVOL2 | 3.323300104 |
|  |  | OXCT1 | 3.149793523 |
|  |  | OXER1 | 4.890913526 |
|  |  | OXTR | -3.224166429 |
|  |  | P2RX5-TAX1BP3 | 1.47274798 |
|  |  | P2RY1 | 1.469776665 |
|  |  | P2RY2 | -1.686824831 |
|  |  | P2RY6 | 3.094301119 |
|  |  | P3H1 | 1.072362031 |
|  |  | P3H2 | 2.117722845 |
|  |  | P3H3 | 1.033422251 |
|  |  | P4HA3 | 2.957763341 |
|  |  | PABPC1L | -1.353587638 |
|  |  | PABPC4L | 4.343894217 |
|  |  | PABPC5 | 6.547562508 |
|  |  | PADI1 | -2.032344316 |
|  |  | PADI4 | 4.325343509 |
|  |  | PAG1 | 6.292834419 |
|  |  | PAK3 | 5.550860563 |
|  |  | PAK5 | 4.632056169 |
|  |  | PALD1 | -1.873651294 |
|  |  | PALMD | 4.359677213 |
|  |  | PAM | 1.043673619 |
|  |  | PANO1 | -1.282229048 |
|  |  | PANX2 | 1.608362656 |
|  |  | PAPPA | -1.275294515 |
|  |  | PAPPA2 | 5.241392914 |
|  |  | PAPSS2 | 2.864962084 |
|  |  | PAQR9 | -1.515756174 |
|  |  | PAQR9-AS1 | -2.36289987 |
|  |  | PARD6A | -1.660443069 |
|  |  | PARD6B | -1.526867355 |
|  |  | PARP10 | 1.130210089 |
|  |  | PARP9 | 2.146313053 |
|  |  | PAWR | -1.664923589 |
|  |  | PAX1 | 4.621831175 |
|  |  | PAX6 | 1.677708168 |
|  |  | PAX8 | -1.264838856 |
|  |  | PAX8-AS1 | -2.434085988 |
|  |  | PBK | 2.426685277 |
|  |  | PBX4 | -1.729375376 |
|  |  | PC | 2.271387427 |
|  |  | PCAT19 | 3.266249895 |
|  |  | PCBP1 | 1.337629466 |
|  |  | PCBP3 | 3.3687596 |
|  |  | PCDH10 | 6.306911052 |
|  |  | PCDH11X | 6.261639282 |
|  |  | PCDH17 | 7.559783794 |
|  |  | PCDH18 | 10.38091744 |
|  |  | PCDH19 | 4.821768052 |
|  |  | PCDH7 | 5.014871981 |
|  |  | PCDHA11 | -1.589994177 |
|  |  | PCDHAC1 | 1.09315347 |
|  |  | PCDHB14 | 1.41826391 |
|  |  | PCDHB15 | 1.825452689 |
|  |  | PCDHB5 | 2.309397381 |
|  |  | PCDHB7 | 3.21330734 |
|  |  | PCDHGA6 | -1.350209148 |
|  |  | PCDHGB5 | -3.454645387 |
|  |  | PCGF6 | -1.205739396 |
|  |  | PCK1 | 6.750874855 |
|  |  | PCLAF | 1.228046908 |
|  |  | PCMTD1 | 1.064191561 |
|  |  | PCNX1 | 1.33083149 |
|  |  | PCOLCE | 2.322130509 |
|  |  | PCOLCE2 | 1.34917539 |
|  |  | PCSK1 | 1.376451642 |
|  |  | PCSK2 | 11.36712511 |
|  |  | PCSK5 | 4.083463905 |
|  |  | PCSK9 | -2.009713906 |
|  |  | PCYT1B | 3.554920343 |
|  |  | PDCD11 | -1.011910791 |
|  |  | PDCD6IPP2 | -3.076742659 |
|  |  | PDCL3P4 | 1.221914932 |
|  |  | PDE10A | 2.505060775 |
|  |  | PDE1A | 7.55527134 |
|  |  | PDE1C | 1.956448812 |
|  |  | PDE3A | 1.626230109 |
|  |  | PDE3B | 3.171492428 |
|  |  | PDE7B | 1.154547948 |
|  |  | PDE8B | 1.283545288 |
|  |  | PDGFB | -3.360025695 |
|  |  | PDGFRA | 5.689638887 |
|  |  | PDGFRB | 5.069619331 |
|  |  | PDGFRL | 1.775658612 |
|  |  | PDIA3 | 1.544856424 |
|  |  | PDIA4 | 1.210834074 |
|  |  | PDIA6 | 1.172004337 |
|  |  | PDK2 | 1.034400964 |
|  |  | PDK3 | 1.568513849 |
|  |  | PDK4 | 2.630467649 |
|  |  | PDLIM1 | -1.078823967 |
|  |  | PDLIM3 | 10.27545932 |
|  |  | PDP1 | -1.60642418 |
|  |  | PDX1 | 4.178867752 |
|  |  | PDXP | -1.599668667 |
|  |  | PDZK1IP1 | -4.50946711 |
|  |  | PDZRN3 | 5.652826493 |
|  |  | PEA15 | -1.042986441 |
|  |  | PEAR1 | -4.3991407 |
|  |  | PECAM1 | 2.363363176 |
|  |  | PEG10 | 1.143645871 |
|  |  | PELI2 | 3.486340299 |
|  |  | PELP1 | -1.174175508 |
|  |  | PER1 | -1.089523846 |
|  |  | PER2 | -1.616527282 |
|  |  | PERM1 | -1.543883894 |
|  |  | PERP | 1.031384224 |
|  |  | PET117 | -1.075554886 |
|  |  | PEX10 | -1.080734284 |
|  |  | PEX16 | -1.362973563 |
|  |  | PFAS | -1.02767131 |
|  |  | PFDN2 | -1.000502425 |
|  |  | PFKFB3 | -2.476114893 |
|  |  | PFKP | -1.766309259 |
|  |  | PGAM1P5 | -3.135248421 |
|  |  | PGAM5 | -1.034965399 |
|  |  | PGBD4 | 1.15129074 |
|  |  | PGBD5 | 5.078121492 |
|  |  | PGF | -1.674704295 |
|  |  | PGM2L1 | 1.344968243 |
|  |  | PGM5P2 | -1.829221571 |
|  |  | PGR | 5.134646972 |
|  |  | PHC2 | -1.018238963 |
|  |  | PHF24 | -5.77018995 |
|  |  | PHF7 | 1.366195996 |
|  |  | PHKA1P1 | -1.725695809 |
|  |  | PHLDA1 | -1.421088933 |
|  |  | PHLDA2 | -1.467015296 |
|  |  | PHLDA3 | -1.059080301 |
|  |  | PHLDB2 | -1.307823486 |
|  |  | PHOSPHO1 | -1.194434325 |
|  |  | PHYHIP | -1.598058918 |
|  |  | PHYHIPL | -3.860471748 |
|  |  | PI15 | 4.305538215 |
|  |  | PIANP | 1.534263043 |
|  |  | PICART1 | -1.998654538 |
|  |  | PID1 | 1.637779428 |
|  |  | PIEZO2 | 2.506161183 |
|  |  | PIF1 | 2.293789384 |
|  |  | PIGA | -1.224850914 |
|  |  | PIGCP1 | -1.311841697 |
|  |  | PIGT | 1.358313155 |
|  |  | PIGZ | 1.520994701 |
|  |  | PIK3AP1 | -1.784467624 |
|  |  | PIK3CD | -1.018081716 |
|  |  | PIK3R1 | 1.218336932 |
|  |  | PIK3R3 | 1.661301294 |
|  |  | PIP4K2C | -1.113223961 |
|  |  | PIP4P2 | 1.083595025 |
|  |  | PIP5K1B | 9.643919121 |
|  |  | PIR | 1.859237767 |
|  |  | PITPNC1 | 1.718392999 |
|  |  | PITPNM3 | -1.561588153 |
|  |  | PITX1 | 1.927576055 |
|  |  | PITX2 | 1.745975021 |
|  |  | PJA2 | 1.202739674 |
|  |  | PKDCC | 3.267664779 |
|  |  | PKIA | 5.159501611 |
|  |  | PKN2-AS1 | -2.39019101 |
|  |  | PKNOX1 | 1.563675015 |
|  |  | PKNOX2 | 4.10809342 |
|  |  | PKP2 | -1.942923556 |
|  |  | PKP3 | -1.666177627 |
|  |  | PLA2G15 | 1.143248124 |
|  |  | PLA2G16 | -2.283338942 |
|  |  | PLA2G4A | 1.75356983 |
|  |  | PLA2R1 | 1.754269357 |
|  |  | PLAC1 | 1.944473799 |
|  |  | PLAG1 | 3.897193938 |
|  |  | PLAT | 1.736066692 |
|  |  | PLAUR | 1.011711699 |
|  |  | PLBD1 | -1.798653876 |
|  |  | PLCB1 | 4.419253378 |
|  |  | PLCD3 | -2.090903499 |
|  |  | PLCE1 | -1.374833422 |
|  |  | PLCH1 | -2.524609041 |
|  |  | PLCH2 | -4.262815261 |
|  |  | PLCL1 | 4.493324661 |
|  |  | PLCXD2 | -2.113143689 |
|  |  | PLD1 | 1.106918917 |
|  |  | PLD3 | 1.030689791 |
|  |  | PLD5 | -3.229113087 |
|  |  | PLD6 | -1.332366189 |
|  |  | PLEC | -1.292251248 |
|  |  | PLEK2 | -4.359885585 |
|  |  | PLEKHA2 | 2.972746504 |
|  |  | PLEKHA4 | 3.382336198 |
|  |  | PLEKHA5 | 1.707388721 |
|  |  | PLEKHA7 | -3.183938799 |
|  |  | PLEKHG4B | 1.76898629 |
|  |  | PLEKHG5 | -1.443594622 |
|  |  | PLEKHN1 | -1.85565553 |
|  |  | PLEKHO1 | 1.659853939 |
|  |  | PLEKHO2 | 1.404654218 |
|  |  | PLGLB1 | 1.59366304 |
|  |  | PLIN1 | 2.265291165 |
|  |  | PLK1 | 2.042453681 |
|  |  | PLK2 | -1.159526021 |
|  |  | PLK3 | -1.239628862 |
|  |  | PLPP3 | -1.56191245 |
|  |  | PLPPR3 | 1.111172426 |
|  |  | PLPPR4 | 7.726600879 |
|  |  | PLS1 | -1.486770928 |
|  |  | PLSCR1 | 1.199046321 |
|  |  | PLSCR4 | 2.084695381 |
|  |  | PLTP | -2.824636418 |
|  |  | PLXDC2 | 7.120466552 |
|  |  | PLXNA2 | 1.547983978 |
|  |  | PLXNA4 | 3.979666721 |
|  |  | PLXND1 | 1.11433215 |
|  |  | PM20D1 | -5.188778798 |
|  |  | PMAIP1 | -2.202977284 |
|  |  | PMEPA1 | -1.268738913 |
|  |  | PMS2P4 | -1.650727314 |
|  |  | PNKD | 1.030220837 |
|  |  | PNP | -1.083333827 |
|  |  | PODNL1 | 4.313198358 |
|  |  | PODXL | -2.175457067 |
|  |  | POF1B | -7.905569171 |
|  |  | POLD4 | -1.376217229 |
|  |  | POLE2 | 1.22685642 |
|  |  | POM121L9P | 2.801900815 |
|  |  | POMGNT2 | 1.285288711 |
|  |  | PON3 | 1.010081677 |
|  |  | PORCN | -1.46675791 |
|  |  | POSTN | 7.697452662 |
|  |  | POT1-AS1 | 1.610891633 |
|  |  | POTEKP | 7.509788306 |
|  |  | POU3F1 | 6.588806807 |
|  |  | POU3F2 | 4.636492612 |
|  |  | POU3F3 | 2.673068026 |
|  |  | POU5F1P4 | -4.607108973 |
|  |  | PPARA | 1.529872715 |
|  |  | PPARGC1A | 4.257209096 |
|  |  | PPDPFL | 5.371008143 |
|  |  | PPEF1 | 1.433581215 |
|  |  | PPFIA4 | 2.059421688 |
|  |  | PPFIBP2 | -3.150759612 |
|  |  | PPIAP14 | 4.591400248 |
|  |  | PPIAP29 | -1.003474857 |
|  |  | PPIB | 1.043611907 |
|  |  | PPIC | 1.295858457 |
|  |  | PPIF | 1.206263158 |
|  |  | PPIL6 | -1.270440959 |
|  |  | PPM1AP1 | 2.680269311 |
|  |  | PPM1E | 1.280856183 |
|  |  | PPM1J | -2.519610203 |
|  |  | PPM1K | 1.088145786 |
|  |  | PPM1L | 1.751972042 |
|  |  | PPP1R13L | -1.12223489 |
|  |  | PPP1R14A | -5.08506633 |
|  |  | PPP1R15A | -3.554134633 |
|  |  | PPP1R16B | -2.920201542 |
|  |  | PPP1R3F | -1.11056358 |
|  |  | PPP1R9A | -1.266843187 |
|  |  | PPP2R1B | 2.166464328 |
|  |  | PPP2R2B | 4.830627966 |
|  |  | PPP2R3B | -1.199085137 |
|  |  | PPP2R5B | -1.53110439 |
|  |  | PPP4R4 | 3.007087297 |
|  |  | PPT1 | 1.219735877 |
|  |  | PRADC1 | 1.107861055 |
|  |  | PRAG1 | -3.393164901 |
|  |  | PRAME | 1.162421441 |
|  |  | PRCP | 1.267288192 |
|  |  | PRDM13 | 1.3233766 |
|  |  | PRDM6 | 2.112870345 |
|  |  | PRELID2 | -1.402798628 |
|  |  | PRELID3A | -2.273049667 |
|  |  | PREX2 | 2.852120762 |
|  |  | PRICKLE1 | -4.376430746 |
|  |  | PRIMA1 | 2.663499358 |
|  |  | PRKAR1A | 1.066708282 |
|  |  | PRKAR1B | -1.957842941 |
|  |  | PRKCB | 5.209636549 |
|  |  | PRKCD | -1.151003834 |
|  |  | PRKCG | -3.351778937 |
|  |  | PRKCH | -1.478648826 |
|  |  | PRKCZ | -1.440509204 |
|  |  | PRKD1 | 1.743759855 |
|  |  | PRKD3 | 1.545601973 |
|  |  | PRKG1 | 3.792850584 |
|  |  | PRKG1-AS1 | -2.997976568 |
|  |  | PRKRIP1 | -1.019222489 |
|  |  | PRLR | 5.764825551 |
|  |  | PRND | 5.575549461 |
|  |  | PROB1 | -1.774473084 |
|  |  | PROCR | -2.153507086 |
|  |  | PRODH2 | -4.724118348 |
|  |  | PROK1 | 5.005407238 |
|  |  | PROK2 | 5.003852374 |
|  |  | PRORSD1P | 1.027054668 |
|  |  | PROSER2 | -2.40357569 |
|  |  | PROSER2-AS1 | -1.807214866 |
|  |  | PROX1 | 2.65675192 |
|  |  | PROZ | 1.438565854 |
|  |  | PRPH | -2.296854674 |
|  |  | PRR11 | 1.35084919 |
|  |  | PRR15 | -2.63585363 |
|  |  | PRR16 | 1.637506825 |
|  |  | PRR29 | -4.635134553 |
|  |  | PRR5L | 3.624205737 |
|  |  | PRRG4 | 2.135800871 |
|  |  | PRRT1 | 1.469198757 |
|  |  | PRRX1 | 7.457016396 |
|  |  | PRSS12 | 2.34845395 |
|  |  | PRSS22 | -5.020661651 |
|  |  | PRSS3 | -6.188114524 |
|  |  | PRSS35 | 2.889993904 |
|  |  | PRSS56 | 5.971915593 |
|  |  | PRTN3 | 2.734118903 |
|  |  | PRUNE2 | 5.38936793 |
|  |  | PSD2 | 1.947751917 |
|  |  | PSG4 | -2.789326322 |
|  |  | PSMB8 | 1.716521656 |
|  |  | PSMB8-AS1 | 2.447442539 |
|  |  | PSMB9 | 1.146926521 |
|  |  | PSMD10P2 | -5.343461828 |
|  |  | PSPH | -1.124395801 |
|  |  | PSRC1 | 1.551048739 |
|  |  | PSTPIP2 | 2.468956989 |
|  |  | PTBP3 | -1.377387005 |
|  |  | PTCHD1 | 2.925348088 |
|  |  | PTCHD4 | 3.169083368 |
|  |  | PTGER2 | 2.106493185 |
|  |  | PTGER3 | 6.361204319 |
|  |  | PTGES3L | -2.17614555 |
|  |  | PTGFR | 5.09693282 |
|  |  | PTGFRN | 1.257558618 |
|  |  | PTHLH | -3.069889817 |
|  |  | PTK6 | -2.096841811 |
|  |  | PTK7 | 1.995461902 |
|  |  | PTN | 11.5812387 |
|  |  | PTOV1-AS1 | 1.270716852 |
|  |  | PTP4A1 | -1.750335206 |
|  |  | PTPDC1 | -1.27807124 |
|  |  | PTPN12 | -1.227626026 |
|  |  | PTPN22 | 4.338916571 |
|  |  | PTPN6 | -3.646640383 |
|  |  | PTPRD | 2.075974771 |
|  |  | PTPRF | -2.372632536 |
|  |  | PTPRG | 1.484703154 |
|  |  | PTPRN | 5.848300396 |
|  |  | PTPRN2 | -5.690454563 |
|  |  | PTPRO | 4.820061819 |
|  |  | PTPRZ1 | 4.22360617 |
|  |  | PTTG1 | 1.900510656 |
|  |  | PUM3 | -1.100471518 |
|  |  | PURG | 3.70504808 |
|  |  | PUS1 | -1.192512285 |
|  |  | PVALB | 5.02629133 |
|  |  | PVR | -1.022396918 |
|  |  | PVRIG | 1.649755968 |
|  |  | PXDN | 5.440833478 |
|  |  | PXDNL | 2.254387845 |
|  |  | PXN | -1.777421494 |
|  |  | PXYLP1 | 1.756501696 |
|  |  | PYCARD | -5.695195146 |
|  |  | PYCR1 | -1.315445698 |
|  |  | PYCR3 | -1.020587173 |
|  |  | PYGB | -1.886318112 |
|  |  | QPCT | 3.096909716 |
|  |  | QPRT | 3.967887555 |
|  |  | QRFPR | 3.917239906 |
|  |  | RAB11FIP4 | -2.203996559 |
|  |  | RAB20 | 5.234750847 |
|  |  | RAB24 | -1.090595005 |
|  |  | RAB27A | 1.289032199 |
|  |  | RAB27B | -1.35914981 |
|  |  | RAB31 | 1.054298464 |
|  |  | RAB36 | -1.269278425 |
|  |  | RAB37 | 1.676403652 |
|  |  | RAB39B | -1.923189878 |
|  |  | RAB3A | 3.526169304 |
|  |  | RAB3B | -4.266356048 |
|  |  | RAB3C | 9.151195703 |
|  |  | RAB3D | 1.381458199 |
|  |  | RAB3IL1 | -1.330254046 |
|  |  | RAB3IP | -1.257990628 |
|  |  | RAB6C | 4.5649376 |
|  |  | RAB6C-AS1 | 4.871408189 |
|  |  | RAB6D | 5.121976357 |
|  |  | RAB7B | 2.546331687 |
|  |  | RAB9B | 3.603954827 |
|  |  | RAC3 | -1.13814202 |
|  |  | RAD21 | 1.168211953 |
|  |  | RAD51B | -1.145691138 |
|  |  | RAET1G | 1.593110541 |
|  |  | RAG1 | 1.092525793 |
|  |  | RAI2 | 4.148571587 |
|  |  | RAP2B | -1.03050102 |
|  |  | RAPGEF3 | -1.851068145 |
|  |  | RARA-AS1 | -2.375979608 |
|  |  | RARB | 2.029661383 |
|  |  | RARRES1 | 3.799602302 |
|  |  | RASA3 | 2.025499 |
|  |  | RASAL2-AS1 | -1.782156572 |
|  |  | RASEF | -4.809779762 |
|  |  | RASGEF1A | -3.974852156 |
|  |  | RASGEF1B | 3.594881181 |
|  |  | RASGEF1C | -5.104183271 |
|  |  | RASGRP3 | -2.02236664 |
|  |  | RASSF2 | -2.110666335 |
|  |  | RASSF6 | 1.263186193 |
|  |  | RASSF7 | -2.304803144 |
|  |  | RASSF8 | 2.393999741 |
|  |  | RASSF8-AS1 | 1.882028939 |
|  |  | RASSF9 | -2.995678722 |
|  |  | RBCK1 | -1.060004771 |
|  |  | RBFA | -1.026864959 |
|  |  | RBFOX3 | -3.708547467 |
|  |  | RBKS | -1.160893743 |
|  |  | RBM11 | 6.167131007 |
|  |  | RBM24 | -3.650007535 |
|  |  | RBM47 | 1.042858524 |
|  |  | RBMS3 | 2.471665308 |
|  |  | RBMS3-AS3 | 2.203253005 |
|  |  | RBP4 | 1.721688695 |
|  |  | RBPMS | -1.038256586 |
|  |  | RBPMS-AS1 | -2.303163647 |
|  |  | RCAN2 | 3.643794438 |
|  |  | RCBTB2 | 1.776470432 |
|  |  | RCN1 | 1.575078251 |
|  |  | RCN1P2 | 1.79345511 |
|  |  | RCN3 | 1.85751292 |
|  |  | RCOR2 | -1.220398242 |
|  |  | RDH10 | 2.365010531 |
|  |  | RDH10-AS1 | 3.247648103 |
|  |  | RECK | 1.856803208 |
|  |  | REEP1 | 3.549051616 |
|  |  | REEP2 | -2.229933077 |
|  |  | RELB | -2.932559456 |
|  |  | RELL2 | -1.385976688 |
|  |  | RELN | 4.162202577 |
|  |  | RELT | -1.104035917 |
|  |  | RERG | 4.699857054 |
|  |  | RERG-IT1 | 4.847051984 |
|  |  | RET | -1.749250556 |
|  |  | RETREG1 | 1.153917496 |
|  |  | RF00019 | 4.201546282 |
|  |  | RFPL1S | 4.080014111 |
|  |  | RFPL2 | -1.195841607 |
|  |  | RFTN2 | 1.452522318 |
|  |  | RFX6 | 4.70904039 |
|  |  | RFXAP | 1.337110589 |
|  |  | RGCC | 6.002268262 |
|  |  | RGL1 | 3.371469413 |
|  |  | RGL3 | -4.482669747 |
|  |  | RGPD5 | 1.004662227 |
|  |  | RGS11 | -3.220473129 |
|  |  | RGS16 | 2.727949168 |
|  |  | RGS17 | -1.674650912 |
|  |  | RGS18 | 7.720395732 |
|  |  | RGS3 | 1.051592544 |
|  |  | RGS4 | 6.666495736 |
|  |  | RGS5 | 1.280666344 |
|  |  | RGS6 | 7.006292534 |
|  |  | RHBDF2 | -2.35241189 |
|  |  | RHCG | -4.2549932 |
|  |  | RHEBL1 | -1.279466998 |
|  |  | RHOB | 2.816277561 |
|  |  | RHOD | -3.837204793 |
|  |  | RHOF | -2.267928943 |
|  |  | RHOJ | 5.345425004 |
|  |  | RHOQP1 | -2.036009576 |
|  |  | RHOT1P1 | -3.483294753 |
|  |  | RHOU | 1.170059868 |
|  |  | RHOV | 4.384088579 |
|  |  | RHPN2 | -1.204481534 |
|  |  | RILP | -1.815483982 |
|  |  | RILPL1 | -1.315335073 |
|  |  | RIMKLA | 1.038403276 |
|  |  | RIMS1 | 4.344828869 |
|  |  | RIMS2 | 1.486152479 |
|  |  | RIN1 | -2.422238919 |
|  |  | RIN3 | -1.040079277 |
|  |  | RINL | -3.034896968 |
|  |  | RMDN2 | 1.408950351 |
|  |  | RMND5A | 1.313976198 |
|  |  | RMST | 5.969298209 |
|  |  | RN7SKP173 | -2.886926743 |
|  |  | RN7SL2 | -2.483151768 |
|  |  | RN7SL417P | 2.634953363 |
|  |  | RN7SL67P | 6.591405907 |
|  |  | RN7SL726P | 5.792148029 |
|  |  | RNA5SP118 | 4.045835485 |
|  |  | RNASEL | 2.080647495 |
|  |  | RND1 | -4.311869547 |
|  |  | RND3 | -1.393031243 |
|  |  | RNF112 | -2.197223122 |
|  |  | RNF121 | -1.073022605 |
|  |  | RNF128 | 4.072283022 |
|  |  | RNF139-AS1 | 1.020529032 |
|  |  | RNF144A | 1.868763588 |
|  |  | RNF144A-AS1 | 2.581545716 |
|  |  | RNF150 | 3.014400008 |
|  |  | RNF152 | 3.043760344 |
|  |  | RNF180 | 1.212402652 |
|  |  | RNF182 | 4.167052856 |
|  |  | RNF19B | -1.82791821 |
|  |  | RNF215 | -1.222008632 |
|  |  | RNF216P1 | -1.106398635 |
|  |  | RNF217-AS1 | 1.489812275 |
|  |  | RNF227 | -1.366871644 |
|  |  | RNF24 | 2.310892496 |
|  |  | RNF32 | -1.067038884 |
|  |  | RNF41 | -1.054836776 |
|  |  | RNU4ATAC18P | 4.386794554 |
|  |  | RNU6-1263P | -4.772434053 |
|  |  | RNU6-26P | -2.532528053 |
|  |  | RNU6-882P | 4.277849206 |
|  |  | ROBO1 | 1.353007459 |
|  |  | ROBO2 | 6.918042791 |
|  |  | ROBO3 | -1.33463045 |
|  |  | ROBO4 | -6.436917136 |
|  |  | ROCK1P1 | -3.671388602 |
|  |  | ROR1 | -2.249887378 |
|  |  | ROR2 | 7.029756634 |
|  |  | RORA | 4.081646402 |
|  |  | RORB | 8.302268565 |
|  |  | RORB-AS1 | 5.179928206 |
|  |  | RORC | 6.054116696 |
|  |  | ROS1 | -2.255568464 |
|  |  | RP9 | -1.663945794 |
|  |  | RP9P | -2.040342435 |
|  |  | RPGR | -1.045409372 |
|  |  | RPL12P14 | -3.066798712 |
|  |  | RPL13AP20 | -2.796626516 |
|  |  | RPL22L1 | 1.012898811 |
|  |  | RPL22P1 | -1.095066581 |
|  |  | RPL22P2 | -2.152150844 |
|  |  | RPL26P30 | 2.026536282 |
|  |  | RPL30P7 | -2.170399806 |
|  |  | RPLP0P2 | -3.840117807 |
|  |  | RPN2 | 1.367337877 |
|  |  | RPRM | 9.105303051 |
|  |  | RPS10P5 | 4.344698754 |
|  |  | RPS10P7 | -2.246934017 |
|  |  | RPS20P22 | -2.085094565 |
|  |  | RPS6KA2 | 3.472662009 |
|  |  | RPS6KA4 | -1.197727242 |
|  |  | RPS6P25 | -3.994851213 |
|  |  | RPSAP52 | -5.105454989 |
|  |  | RPSAP72 | 4.675117878 |
|  |  | RRAGD | 1.380327799 |
|  |  | RREB1 | 1.071462901 |
|  |  | RRM2 | 1.172802116 |
|  |  | RRP12 | -1.321433855 |
|  |  | RSAD1 | -1.137259757 |
|  |  | RSAD2 | 5.900349305 |
|  |  | RSPH1 | -2.839338544 |
|  |  | RSPH14 | -2.614549381 |
|  |  | RSPH9 | -2.053012502 |
|  |  | RSPO2 | 7.913749468 |
|  |  | RSRC1 | 1.080871121 |
|  |  | RTKN2 | 1.39630719 |
|  |  | RTL1 | 2.148316776 |
|  |  | RTL10 | 1.187096127 |
|  |  | RTL9 | 4.856577985 |
|  |  | RTN1 | 9.625324173 |
|  |  | RTTN | -1.82724382 |
|  |  | RUNDC1 | -1.106983814 |
|  |  | RUNDC3A-AS1 | -1.691203161 |
|  |  | RUNX1T1 | 4.914833503 |
|  |  | RUSC2 | -2.560317734 |
|  |  | RXFP2 | 6.331683181 |
|  |  | RYR2 | 2.639720628 |
|  |  | S100A16 | -2.153061655 |
|  |  | S100A4 | 1.361278789 |
|  |  | S100A8 | 5.090334407 |
|  |  | S100A9 | 4.498299636 |
|  |  | S100P | -2.873220559 |
|  |  | S1PR1 | 4.762540806 |
|  |  | S1PR3 | 1.634938143 |
|  |  | S1PR5 | 1.822205545 |
|  |  | SAC3D1 | -1.047375354 |
|  |  | SACM1L | 1.163483023 |
|  |  | SACS | -1.35290286 |
|  |  | SAGE1 | -6.810431552 |
|  |  | SALL1 | 2.334329111 |
|  |  | SALL2 | 3.114277929 |
|  |  | SALL4 | -1.011040511 |
|  |  | SAMD11 | 1.534975638 |
|  |  | SAMD12 | 1.411875239 |
|  |  | SAMD14 | -1.054402344 |
|  |  | SAMD4A | -2.664369101 |
|  |  | SAMD5 | 2.516497866 |
|  |  | SAMD9L | 1.92470164 |
|  |  | SAP30L-AS1 | -2.635479092 |
|  |  | SAPCD2 | 1.348800203 |
|  |  | SAR1A | 1.111053821 |
|  |  | SARDH | 2.237730124 |
|  |  | SARM1 | -2.937883706 |
|  |  | SATB1 | 6.010684124 |
|  |  | SATB1-AS1 | 4.775335081 |
|  |  | SBDSP1 | -1.211069509 |
|  |  | SBF2-AS1 | -1.149519124 |
|  |  | SBK2 | -5.555247532 |
|  |  | SBSN | -2.689028848 |
|  |  | SBSPON | 2.413202246 |
|  |  | SCARA5 | 5.204941475 |
|  |  | SCART1 | -1.434844144 |
|  |  | SCD | 3.082504927 |
|  |  | SCEL | -4.451750864 |
|  |  | SCG2 | 8.849628826 |
|  |  | SCML1 | 1.078773566 |
|  |  | SCN2A | 2.644477782 |
|  |  | SCN3A | 2.972714148 |
|  |  | SCN4A | -4.194536668 |
|  |  | SCN5A | 1.222733415 |
|  |  | SCNN1B | 5.064235896 |
|  |  | SCNN1G | 6.465119239 |
|  |  | SCPEP1 | 1.103886672 |
|  |  | SCRN1 | -1.269692281 |
|  |  | SCRN3 | 1.015476092 |
|  |  | SCT | -2.867867446 |
|  |  | SCUBE1 | 2.687851908 |
|  |  | SDC1 | -1.28977997 |
|  |  | SDC2 | 6.767468889 |
|  |  | SDCBP2 | 2.086178258 |
|  |  | SDHAF4 | 1.149477502 |
|  |  | SDK1 | 3.889169306 |
|  |  | SDK2 | -5.249863421 |
|  |  | SDR16C5 | 3.423482592 |
|  |  | SDR42E1 | -1.930880634 |
|  |  | SDSL | -1.421398606 |
|  |  | SEH1L | -1.038042905 |
|  |  | SEL1L3 | 1.233262316 |
|  |  | SELENBP1 | 3.197885027 |
|  |  | SELENOP | 6.898880618 |
|  |  | SELL | 6.610149523 |
|  |  | SELPLG | -2.023437421 |
|  |  | SEMA3C | -1.514116608 |
|  |  | SEMA3D | 4.184234046 |
|  |  | SEMA3E | 2.354772549 |
|  |  | SEMA4A | -1.783699891 |
|  |  | SEMA4B | 1.20267333 |
|  |  | SEMA5B | 5.379737618 |
|  |  | SEMA6A | 2.236207434 |
|  |  | SEMA6B | 2.215871336 |
|  |  | SEMA6D | 10.5552438 |
|  |  | SEMA7A | -1.644027016 |
|  |  | SENP3 | -1.03458517 |
|  |  | SENP7 | 1.081360679 |
|  |  | SENP8 | 1.593407499 |
|  |  | SERF2 | 1.286753862 |
|  |  | SERINC2 | -1.013177565 |
|  |  | SERPINA1 | 3.698708679 |
|  |  | SERPINB7 | -7.103211745 |
|  |  | SERPINB8 | -1.20953563 |
|  |  | SERPINB9 | 1.633731962 |
|  |  | SERPINE1 | -3.645143684 |
|  |  | SERPING1 | 4.04513255 |
|  |  | SERPINH1 | 1.033763978 |
|  |  | SERPINI1 | 2.079349952 |
|  |  | SERTAD4 | 2.126747518 |
|  |  | SERTM1 | 8.621247972 |
|  |  | SERTM2 | 3.465484904 |
|  |  | SESN1 | 1.001220678 |
|  |  | SESN2 | -2.583674427 |
|  |  | SESN3 | 4.057483728 |
|  |  | SESTD1 | 1.585182788 |
|  |  | SETBP1 | 2.022289783 |
|  |  | SETMAR | 1.41424068 |
|  |  | SEZ6 | -5.664941519 |
|  |  | SFMBT2 | -1.891275885 |
|  |  | SFN | -1.560979349 |
|  |  | SFR1 | -1.311727184 |
|  |  | SFRP1 | 4.139805826 |
|  |  | SFRP2 | 9.423757475 |
|  |  | SFTA1P | 6.145550363 |
|  |  | SFTPC | 2.420162234 |
|  |  | SFTPD-AS1 | 4.928679037 |
|  |  | SGCB | 1.049814138 |
|  |  | SGCD | -6.813835237 |
|  |  | SGCZ | 7.813409698 |
|  |  | SGK1 | 2.496461588 |
|  |  | SGK2 | -3.03260496 |
|  |  | SGK3 | 1.781647596 |
|  |  | SGMS1 | 1.412371874 |
|  |  | SGMS1-AS1 | 1.088577964 |
|  |  | SGO2 | 1.237973285 |
|  |  | SGPL1 | -1.117282481 |
|  |  | SGPP2 | -1.679653982 |
|  |  | SGSM3 | -1.141119091 |
|  |  | SH2D3A | -2.335471938 |
|  |  | SH2D4B | -3.044751262 |
|  |  | SH2D5 | -4.589641556 |
|  |  | SH3BGRL | 1.650383452 |
|  |  | SH3BGRL2 | 2.162534408 |
|  |  | SH3BGRL3 | -2.195847416 |
|  |  | SH3BP2 | -1.548500065 |
|  |  | SH3BP5 | 4.012590455 |
|  |  | SH3BP5-AS1 | 1.708865144 |
|  |  | SH3GL2 | 7.534014084 |
|  |  | SH3GL3 | 2.649646988 |
|  |  | SH3KBP1 | -1.723860645 |
|  |  | SH3PXD2B | 3.027791074 |
|  |  | SH3RF1 | -2.194596554 |
|  |  | SH3TC1 | 1.802740727 |
|  |  | SH3TC2 | 1.773411062 |
|  |  | SHANK1 | 4.525321762 |
|  |  | SHANK2 | -1.907984913 |
|  |  | SHB | -2.337168336 |
|  |  | SHC2 | 3.196494673 |
|  |  | SHC4 | 3.472021983 |
|  |  | SHCBP1 | 1.021806988 |
|  |  | SHH | -1.350235493 |
|  |  | SHISA2 | 3.621729561 |
|  |  | SHISA3 | 6.229095821 |
|  |  | SHISA4 | -1.018439969 |
|  |  | SHISAL1 | 3.346731048 |
|  |  | SHISAL2B | 5.982371 |
|  |  | SHOX2 | 2.292816773 |
|  |  | SHROOM1 | 1.032504962 |
|  |  | SIAE | 1.626168765 |
|  |  | SIDT1 | 1.592129004 |
|  |  | SIGLEC16 | -1.785330921 |
|  |  | SIK2 | 2.320085547 |
|  |  | SIM2 | -1.633975716 |
|  |  | SINHCAF | -1.214168569 |
|  |  | SIPA1 | -1.462799014 |
|  |  | SIPA1L3 | -1.042885796 |
|  |  | SIRLNT | 6.33251706 |
|  |  | SIRPA | 1.34349345 |
|  |  | SIX4 | 1.428673857 |
|  |  | SKP2 | 2.505069009 |
|  |  | SLA | 2.448763177 |
|  |  | SLAIN1 | 1.648559212 |
|  |  | SLAMF7 | -8.422615042 |
|  |  | SLC10A7 | 1.05364456 |
|  |  | SLC12A8 | 2.280998288 |
|  |  | SLC13A5 | 2.695471823 |
|  |  | SLC15A2 | 1.677425048 |
|  |  | SLC16A10 | 2.018861512 |
|  |  | SLC16A11 | -2.504413102 |
|  |  | SLC16A13 | -1.50415891 |
|  |  | SLC16A14 | 4.542905258 |
|  |  | SLC16A1-AS1 | -1.92822711 |
|  |  | SLC16A4 | 1.669550233 |
|  |  | SLC16A6 | 1.77267737 |
|  |  | SLC17A7 | 1.628715996 |
|  |  | SLC18B1 | 1.430791398 |
|  |  | SLC1A3 | 6.805867865 |
|  |  | SLC22A11 | -5.413571999 |
|  |  | SLC22A17 | 2.236271183 |
|  |  | SLC22A18AS | -2.239090119 |
|  |  | SLC22A20P | -2.135317248 |
|  |  | SLC22A23 | 1.017459386 |
|  |  | SLC22A3 | -3.952356134 |
|  |  | SLC24A4 | -4.388546795 |
|  |  | SLC25A12 | 1.016487636 |
|  |  | SLC25A13 | 1.203614129 |
|  |  | SLC25A19 | -1.265798623 |
|  |  | SLC25A23 | 1.178147631 |
|  |  | SLC25A25 | -1.143716213 |
|  |  | SLC25A33 | -1.58194985 |
|  |  | SLC26A4-AS1 | -1.75088485 |
|  |  | SLC27A2 | -1.281055593 |
|  |  | SLC27A6 | 3.87819803 |
|  |  | SLC2A12 | 3.551898933 |
|  |  | SLC2A13 | 2.244679484 |
|  |  | SLC2A14 | 2.459617563 |
|  |  | SLC2A3 | 1.100082853 |
|  |  | SLC2A6 | -1.734620205 |
|  |  | SLC2A8 | -1.036922024 |
|  |  | SLC2A9 | -5.654499794 |
|  |  | SLC30A1 | 1.035952204 |
|  |  | SLC30A3 | 1.554650097 |
|  |  | SLC34A3 | -1.956896091 |
|  |  | SLC35D2 | -1.16491303 |
|  |  | SLC35F1 | 3.526428113 |
|  |  | SLC35F2 | -1.101970131 |
|  |  | SLC35F3 | -2.235280056 |
|  |  | SLC36A1 | 1.003060478 |
|  |  | SLC36A4 | -1.011326026 |
|  |  | SLC38A2 | -1.376049325 |
|  |  | SLC38A6 | 1.0751302 |
|  |  | SLC38A9 | -1.00860692 |
|  |  | SLC39A14 | -1.224082149 |
|  |  | SLC39A6 | 1.214638157 |
|  |  | SLC39A8 | 2.754995344 |
|  |  | SLC3A2 | -1.173894042 |
|  |  | SLC40A1 | 1.338356719 |
|  |  | SLC43A2 | -1.063229095 |
|  |  | SLC44A1 | 2.35549726 |
|  |  | SLC45A1 | -4.720809228 |
|  |  | SLC45A3 | -2.123197739 |
|  |  | SLC45A4 | 1.296779259 |
|  |  | SLC46A3 | 2.141905805 |
|  |  | SLC47A1P1 | -2.153094238 |
|  |  | SLC4A2 | -1.234371164 |
|  |  | SLC4A7 | -2.103900149 |
|  |  | SLC51B | -4.488511474 |
|  |  | SLC52A1 | -1.870361764 |
|  |  | SLC5A1 | -4.896399285 |
|  |  | SLC6A11 | 3.479891842 |
|  |  | SLC6A12 | -1.534172014 |
|  |  | SLC6A3 | 4.095357481 |
|  |  | SLC6A6 | 1.919748446 |
|  |  | SLC7A10 | 4.150560086 |
|  |  | SLC7A2 | 2.224041333 |
|  |  | SLC7A5 | -1.196485922 |
|  |  | SLC7A7 | -1.318687398 |
|  |  | SLC7A8 | 7.906963145 |
|  |  | SLC8A3 | 2.223345999 |
|  |  | SLC9A1 | -1.407332182 |
|  |  | SLC9A3R1 | 1.346025643 |
|  |  | SLC9A3R2 | -2.297218931 |
|  |  | SLC9A7 | -1.025074735 |
|  |  | SLC9A9 | 2.276606552 |
|  |  | SLC9C1 | -1.998257374 |
|  |  | SLCO1A2 | -1.572584235 |
|  |  | SLCO1B1 | -2.394770408 |
|  |  | SLCO1B3 | -2.758977299 |
|  |  | SLCO1B7 | -3.123379724 |
|  |  | SLCO1C1 | 2.217938163 |
|  |  | SLCO4C1 | 1.822141679 |
|  |  | SLFN11 | 2.233995056 |
|  |  | SLFN12L | 4.89871771 |
|  |  | SLFN5 | -1.923537033 |
|  |  | SLFNL1 | -2.62506673 |
|  |  | SLFNL1-AS1 | -2.71305006 |
|  |  | SLIT2 | 5.574796252 |
|  |  | SLIT3 | 1.275528769 |
|  |  | SLITRK2 | 6.375480036 |
|  |  | SLITRK3 | 3.33321887 |
|  |  | SLITRK6 | 5.578195529 |
|  |  | SMAD7 | -1.937295246 |
|  |  | SMAD9 | 3.263988692 |
|  |  | SMAGP | -2.515110024 |
|  |  | SMARCD3 | 1.37279302 |
|  |  | SMC2-AS1 | 2.304050397 |
|  |  | SMC4 | 1.267490085 |
|  |  | SMG1P2 | -1.024288137 |
|  |  | SMG7-AS1 | -1.204371774 |
|  |  | SMIM10L2B | 2.579912043 |
|  |  | SMIM25 | 5.699346887 |
|  |  | SMIM3 | 1.521297865 |
|  |  | SMIM6 | -4.387152662 |
|  |  | SMKR1 | -2.153200576 |
|  |  | SMOC1 | 2.583377698 |
|  |  | SMOC2 | 3.43022869 |
|  |  | SMPD1 | 1.293573158 |
|  |  | SMPDL3A | 2.095240388 |
|  |  | SMPX | 3.315777702 |
|  |  | SMURF2 | -1.771505671 |
|  |  | SMURF2P1 | -1.112751135 |
|  |  | SMYD4 | -1.072964265 |
|  |  | SNAI1 | 3.207938341 |
|  |  | SNAP25 | 1.766822781 |
|  |  | SNAP25-AS1 | 2.056314423 |
|  |  | SNAP91 | 2.232435086 |
|  |  | SNAPC1 | -1.470651005 |
|  |  | SNCA | 4.416129923 |
|  |  | SNCG | -2.372926604 |
|  |  | SNED1 | 5.03734226 |
|  |  | SNHG1 | -1.405011815 |
|  |  | SNHG12 | -1.158662679 |
|  |  | SNHG15 | -1.688308803 |
|  |  | SNHG17 | -1.083664568 |
|  |  | SNHG19 | 1.511415268 |
|  |  | SNHG8 | -1.331637597 |
|  |  | SNN | 2.822392644 |
|  |  | SNORC | -2.201908692 |
|  |  | SNORD17 | -1.293782932 |
|  |  | SNTB1 | 1.595352556 |
|  |  | SNTG1 | 4.956486806 |
|  |  | SNX11 | -1.214730467 |
|  |  | SNX18P12 | 2.192347223 |
|  |  | SNX21 | 1.095990088 |
|  |  | SNX29 | 1.092621077 |
|  |  | SOCS1 | 1.361693949 |
|  |  | SOCS2 | -1.92875567 |
|  |  | SOCS2-AS1 | -1.575920693 |
|  |  | SOCS6 | -2.191264424 |
|  |  | SOHLH2 | 4.798415886 |
|  |  | SORBS2 | 6.773134833 |
|  |  | SORCS2 | 1.728171818 |
|  |  | SORCS3 | 3.355220456 |
|  |  | SORL1 | 1.716549454 |
|  |  | SORT1 | -1.604046688 |
|  |  | SOWAHB | 2.118048768 |
|  |  | SOWAHC | -1.358020047 |
|  |  | SOX14 | 3.949942574 |
|  |  | SOX18 | 3.729025508 |
|  |  | SOX2 | 3.438254652 |
|  |  | SOX21-AS1 | 2.204819082 |
|  |  | SOX4 | -1.072864511 |
|  |  | SOX5 | 8.668056908 |
|  |  | SOX6 | 2.771230534 |
|  |  | SP140 | 2.363362319 |
|  |  | SP2-AS1 | -1.113669584 |
|  |  | SP5 | 2.808506813 |
|  |  | SP6 | -1.871396811 |
|  |  | SP8 | 3.299014064 |
|  |  | SPACA6 | 1.933479641 |
|  |  | SPACA6P-AS | 2.018195268 |
|  |  | SPAG17 | 2.263762765 |
|  |  | SPAG5 | 1.459978376 |
|  |  | SPANXB1 | -5.40532217 |
|  |  | SPARC | 9.30397088 |
|  |  | SPARCL1 | 4.217504361 |
|  |  | SPART | 1.529295287 |
|  |  | SPATA2L | -1.075076608 |
|  |  | SPATA3-AS1 | -2.550258993 |
|  |  | SPATA4 | -3.708392365 |
|  |  | SPATA6L | -2.351501357 |
|  |  | SPATS2 | -1.02782497 |
|  |  | SPC24 | 1.621894415 |
|  |  | SPC25 | 1.188188596 |
|  |  | SPCS2P4 | -1.173389898 |
|  |  | SPDEF | -4.231459005 |
|  |  | SPDL1 | -1.133013882 |
|  |  | SPDYE18 | -1.51108701 |
|  |  | SPEF1 | -2.770076322 |
|  |  | SPEG | 2.006341637 |
|  |  | SPHK1 | -1.235314609 |
|  |  | SPI1 | 3.111560889 |
|  |  | SPIB | -2.697715617 |
|  |  | SPINK13 | 7.188002597 |
|  |  | SPINK4 | 3.028332295 |
|  |  | SPINK5 | 2.79924931 |
|  |  | SPINK6 | 8.414024275 |
|  |  | SPINT2 | -6.033007636 |
|  |  | SPIRE1 | -1.521866542 |
|  |  | SPNS2 | -1.844216126 |
|  |  | SPOCK1 | 1.460312819 |
|  |  | SPOCK3 | 1.918220637 |
|  |  | SPP1 | 2.239407644 |
|  |  | SPR | -1.020221598 |
|  |  | SPRY2 | -1.364049972 |
|  |  | SPRY3 | 2.946622852 |
|  |  | SPRYD3 | -1.450177502 |
|  |  | SPSB2 | -1.133118247 |
|  |  | SPSB4 | 2.107697756 |
|  |  | SPTA1 | 5.85584537 |
|  |  | SPTBN1 | -1.057380283 |
|  |  | SPTBN5 | -1.842118225 |
|  |  | SPTLC3 | 4.970977371 |
|  |  | SPTSSA | 3.528361656 |
|  |  | SPX | -1.303332533 |
|  |  | SRC | -1.736501625 |
|  |  | SRD5A3 | 1.099640835 |
|  |  | SREBF1 | 1.347697466 |
|  |  | SREBF2 | 1.006168123 |
|  |  | SRGAP1 | -2.582576933 |
|  |  | SRGN | 1.761238758 |
|  |  | SRPX | 1.832622042 |
|  |  | SRPX2 | 2.982789501 |
|  |  | SRRM2-AS1 | -1.617579725 |
|  |  | SSBP2 | 1.646075852 |
|  |  | SSBP3 | 1.827822491 |
|  |  | SSC5D | 3.825621692 |
|  |  | SSH1 | -1.415774253 |
|  |  | SSH3 | -1.005118693 |
|  |  | SSPN | 3.257699184 |
|  |  | SSR1 | 1.110188551 |
|  |  | SSR2 | 1.066619279 |
|  |  | SST | 6.394749306 |
|  |  | SSTR1 | 3.190797629 |
|  |  | SSTR2 | 4.514113501 |
|  |  | SSTR5 | -4.388810236 |
|  |  | ST14 | -2.334955228 |
|  |  | ST18 | 1.899903415 |
|  |  | ST5 | 1.151064423 |
|  |  | ST6GAL1 | -1.539081795 |
|  |  | ST6GAL2 | -9.286495083 |
|  |  | ST6GALNAC5 | 3.563595075 |
|  |  | ST8SIA1 | 2.48700172 |
|  |  | ST8SIA2 | 3.362386164 |
|  |  | ST8SIA4 | 3.389888119 |
|  |  | ST8SIA5 | 1.708892894 |
|  |  | STAC2 | 5.859372438 |
|  |  | STAM-AS1 | 1.504580762 |
|  |  | STAMBPL1 | -1.37515613 |
|  |  | STARD13 | -1.423111553 |
|  |  | STARD4 | 3.454793588 |
|  |  | STARD4-AS1 | 2.679531137 |
|  |  | STARD5 | 1.363471484 |
|  |  | STARD8 | 2.406788912 |
|  |  | STC1 | 3.076422992 |
|  |  | STC2 | -2.157794528 |
|  |  | STEAP1 | -2.324540997 |
|  |  | STEAP1B | 2.523321536 |
|  |  | STEAP2 | -2.291860973 |
|  |  | STIM1 | -1.096607896 |
|  |  | STIM2 | 1.03460168 |
|  |  | STK10 | -1.360019645 |
|  |  | STK26 | 1.248040981 |
|  |  | STK31 | -1.444737228 |
|  |  | STK32A | 5.008289869 |
|  |  | STK32A-AS1 | 5.471284369 |
|  |  | STK32C | -1.609386693 |
|  |  | STMN1 | 1.162893152 |
|  |  | STMN3 | 1.484686964 |
|  |  | STON1 | 1.996765178 |
|  |  | STON2 | 1.074909743 |
|  |  | STOX2 | 2.841470845 |
|  |  | STPG1 | -1.639956695 |
|  |  | STPG2-AS1 | 5.651361177 |
|  |  | STRA6 | -1.270850588 |
|  |  | STS | 1.701885296 |
|  |  | STUM | 4.67731544 |
|  |  | STX1A | -1.611026602 |
|  |  | STX1B | -1.446268393 |
|  |  | STXBP5L | 7.622397839 |
|  |  | STYK1 | 1.551361107 |
|  |  | SUCLG1 | 1.533973675 |
|  |  | SUFU | 1.212123659 |
|  |  | SUGP2 | 1.093333768 |
|  |  | SULF1 | 5.099882547 |
|  |  | SULT1A1 | 4.659919917 |
|  |  | SULT1A2 | 3.127647807 |
|  |  | SULT1C2 | 2.306351169 |
|  |  | SULT2B1 | -2.548493037 |
|  |  | SUMF1 | 1.5296256 |
|  |  | SUN2 | 1.061520497 |
|  |  | SUSD2 | -1.037624858 |
|  |  | SUSD5 | 3.825758203 |
|  |  | SUZ12 | -10.86886577 |
|  |  | SUZ12P1 | -1.18383896 |
|  |  | SV2B | 2.67654487 |
|  |  | SV2C | 9.35355274 |
|  |  | SVOPL | 5.306617449 |
|  |  | SYCE1L | -1.326493484 |
|  |  | SYDE2 | -1.130076698 |
|  |  | SYK | 2.609012226 |
|  |  | SYN2 | 1.056693983 |
|  |  | SYNDIG1 | 1.116096438 |
|  |  | SYNE1 | -1.657036881 |
|  |  | SYNE2 | 1.067811456 |
|  |  | SYNE3 | 1.374096376 |
|  |  | SYNGR2 | -1.43464427 |
|  |  | SYNGR3 | -1.957123556 |
|  |  | SYNJ2BP | 1.011582898 |
|  |  | SYPL2 | 4.307028298 |
|  |  | SYT1 | -3.405728364 |
|  |  | SYT11 | 1.13735122 |
|  |  | SYT13 | -1.944960978 |
|  |  | SYT14 | 3.978414611 |
|  |  | SYT4 | 7.659453459 |
|  |  | SYT5 | -1.099089263 |
|  |  | SYT6 | -5.027456668 |
|  |  | SYTL1 | 1.591728453 |
|  |  | SYTL3 | 2.457602853 |
|  |  | SYTL4 | 1.103337744 |
|  |  | SYTL5 | 2.281339948 |
|  |  | TAC1 | 6.550274531 |
|  |  | TAC4 | -4.880610692 |
|  |  | TACR1 | 4.352595886 |
|  |  | TAF10 | -1.761539482 |
|  |  | TAF1A | -1.747840987 |
|  |  | TANC2 | -1.237340107 |
|  |  | TAOK3 | 1.527400257 |
|  |  | TAPBPL | 1.461541914 |
|  |  | TAS2R14 | -1.257578561 |
|  |  | TBC1D10C | -1.466372586 |
|  |  | TBC1D19 | 1.329389093 |
|  |  | TBC1D22A | -1.019061975 |
|  |  | TBC1D2B | 1.374861171 |
|  |  | TBC1D3P1 | -3.942010003 |
|  |  | TBC1D4 | 1.050227132 |
|  |  | TBC1D8-AS1 | 1.694401588 |
|  |  | TBC1D8B | 2.477539591 |
|  |  | TBILA | -1.079608557 |
|  |  | TBX1 | 3.56448207 |
|  |  | TBX20 | -1.185442449 |
|  |  | TBX22 | 9.06059904 |
|  |  | TBX5 | 4.769522268 |
|  |  | TBXAS1 | -1.17844373 |
|  |  | TBXT | 4.652134137 |
|  |  | TC2N | 3.16582113 |
|  |  | TCAF1P1 | -1.280726982 |
|  |  | TCAF2 | 1.395065251 |
|  |  | TCEAL2 | 4.35511925 |
|  |  | TCEAL3 | -1.368491157 |
|  |  | TCEAL8 | 1.622083732 |
|  |  | TCF24 | 1.339905906 |
|  |  | TCF4 | 1.635557554 |
|  |  | TCF7L1 | 1.402861614 |
|  |  | TCHH | 1.649828032 |
|  |  | TCIM | -2.408437988 |
|  |  | TCP11L1 | -1.681137874 |
|  |  | TCTEX1D4 | -1.393373624 |
|  |  | TDO2 | 2.004271412 |
|  |  | TDRD5 | 3.090173087 |
|  |  | TDRD6 | 1.674806005 |
|  |  | TEAD2 | 1.157537433 |
|  |  | TEAD4 | -1.301435531 |
|  |  | TECR | 1.169762073 |
|  |  | TENM1 | 7.366080078 |
|  |  | TENM2 | 3.791294533 |
|  |  | TENM3 | 1.412662087 |
|  |  | TENM4 | 2.17730212 |
|  |  | TENT5A | 4.24400513 |
|  |  | TENT5C | 5.774411108 |
|  |  | TERT | -1.668289318 |
|  |  | TESC | 1.691978323 |
|  |  | TESK2 | 2.099465691 |
|  |  | TET1 | 2.434985088 |
|  |  | TET2 | 1.004856802 |
|  |  | TEX15 | 2.026476708 |
|  |  | TEX19 | 2.275839534 |
|  |  | TEX41 | -3.513038413 |
|  |  | TFCP2L1 | -3.209006088 |
|  |  | TFEC | 2.949292762 |
|  |  | TFF1 | -4.06849864 |
|  |  | TFPI | 1.60408321 |
|  |  | TFR2 | -1.036879702 |
|  |  | TFRC | 1.32845112 |
|  |  | TG | 1.960239087 |
|  |  | TGFA | 1.093549352 |
|  |  | TGFB1 | 1.40802719 |
|  |  | TGFB1I1 | -1.532394036 |
|  |  | TGFB3 | 2.934597991 |
|  |  | TGFBI | 1.941539773 |
|  |  | TGFBR1 | 1.385035176 |
|  |  | TGM2 | -2.310399237 |
|  |  | THBD | -2.215956742 |
|  |  | THBS2 | -3.141830723 |
|  |  | THEM5 | 7.89691171 |
|  |  | THORLNC | -3.329466267 |
|  |  | THPO | 4.219303701 |
|  |  | THRA | 1.212114387 |
|  |  | THSD1 | -2.488043503 |
|  |  | THSD4 | -1.495174389 |
|  |  | THSD7A | -1.349429128 |
|  |  | TIA1 | 1.041815882 |
|  |  | TIGD7 | -1.666124244 |
|  |  | TIMD4 | -6.906025113 |
|  |  | TIMP1 | 2.09080822 |
|  |  | TIMP2 | 1.584890317 |
|  |  | TIMP3 | 1.326510004 |
|  |  | TINAGL1 | -5.315807435 |
|  |  | TIPARP | -2.688428604 |
|  |  | TKT | 1.300563688 |
|  |  | TLCD1 | -1.534361435 |
|  |  | TLCD2 | -1.671545235 |
|  |  | TLDC1 | -2.134510982 |
|  |  | TLE1 | -2.787492058 |
|  |  | TLE2 | 1.276132856 |
|  |  | TLE3 | 1.571594894 |
|  |  | TLE4 | -2.639615011 |
|  |  | TLE6 | 2.210451194 |
|  |  | TLK2P1 | -1.519256694 |
|  |  | TLL1 | 5.53327722 |
|  |  | TLL2 | 1.374788597 |
|  |  | TLR3 | 1.46926278 |
|  |  | TLR4 | 1.292927276 |
|  |  | TLR6 | -1.222908357 |
|  |  | TM4SF18 | -3.776313305 |
|  |  | TM4SF19 | -2.354670564 |
|  |  | TM4SF19-AS1 | -1.211216021 |
|  |  | TM4SF20 | -3.503394585 |
|  |  | TM4SF4 | -8.519846599 |
|  |  | TM7SF2 | -1.624612002 |
|  |  | TMC3-AS1 | 2.398312327 |
|  |  | TMC5 | -3.682255766 |
|  |  | TMC7 | -1.185778857 |
|  |  | TMC8 | -1.566632554 |
|  |  | TMED10 | 1.089666705 |
|  |  | TMED3 | 1.216173391 |
|  |  | TMEM100 | 9.469027085 |
|  |  | TMEM105 | -4.350822126 |
|  |  | TMEM108 | 4.020850216 |
|  |  | TMEM116 | 1.112555173 |
|  |  | TMEM121B | 1.701637983 |
|  |  | TMEM131L | 2.081405205 |
|  |  | TMEM132B | 2.860159277 |
|  |  | TMEM132D-AS1 | -3.175971002 |
|  |  | TMEM145 | 1.118697115 |
|  |  | TMEM155 | 4.877554569 |
|  |  | TMEM156 | -6.430261928 |
|  |  | TMEM159 | -1.04500441 |
|  |  | TMEM160 | -1.046192801 |
|  |  | TMEM161B-AS1 | 1.298192192 |
|  |  | TMEM163 | 5.617726184 |
|  |  | TMEM17 | -1.106513371 |
|  |  | TMEM173 | 1.688163963 |
|  |  | TMEM176A | 3.942378539 |
|  |  | TMEM176B | 3.341658828 |
|  |  | TMEM184A | -1.803311172 |
|  |  | TMEM190 | 1.211214201 |
|  |  | TMEM191A | -3.043932037 |
|  |  | TMEM200C | 6.891719744 |
|  |  | TMEM213 | -4.725364818 |
|  |  | TMEM214 | 1.437949849 |
|  |  | TMEM215 | 3.856806443 |
|  |  | TMEM229A | 5.218921078 |
|  |  | TMEM233 | 5.452054212 |
|  |  | TMEM234 | -1.199806573 |
|  |  | TMEM241 | -1.181099001 |
|  |  | TMEM255A | 6.075419076 |
|  |  | TMEM268 | -1.287880832 |
|  |  | TMEM269 | -1.43891233 |
|  |  | TMEM30A | 1.452745099 |
|  |  | TMEM37 | 3.001116198 |
|  |  | TMEM45A | 2.580959119 |
|  |  | TMEM51-AS1 | -1.699873942 |
|  |  | TMEM53 | -1.014548278 |
|  |  | TMEM54 | -1.434989643 |
|  |  | TMEM59L | -1.121987754 |
|  |  | TMEM63C | 1.569274841 |
|  |  | TMEM64 | 1.086565258 |
|  |  | TMEM71 | 2.178588362 |
|  |  | TMEM72 | 5.33332294 |
|  |  | TMEM74 | 7.159631098 |
|  |  | TMEM79 | -1.268569473 |
|  |  | TMEM87B | -1.547842037 |
|  |  | TMEM98 | 1.912191351 |
|  |  | TMIE | -3.024530832 |
|  |  | TMOD1 | 3.474045248 |
|  |  | TMPO-AS1 | 1.160271026 |
|  |  | TMPRSS15 | 6.855805008 |
|  |  | TMPRSS3 | 5.033482649 |
|  |  | TMPRSS9 | -1.292531849 |
|  |  | TMSB10 | 1.007397408 |
|  |  | TMSB15A | 8.545109829 |
|  |  | TMTC1 | 6.947345816 |
|  |  | TMTC2 | 1.285186361 |
|  |  | TMX4 | 2.196989347 |
|  |  | TNC | 4.13750668 |
|  |  | TNFAIP3 | 1.334053727 |
|  |  | TNFAIP8L1 | 1.973856282 |
|  |  | TNFAIP8L3 | 1.444508014 |
|  |  | TNFRSF10A | -1.966950841 |
|  |  | TNFRSF10A-AS1 | -1.785991335 |
|  |  | TNFRSF10B | -1.253736766 |
|  |  | TNFRSF10D | 2.191530163 |
|  |  | TNFRSF11B | 12.12581151 |
|  |  | TNFRSF12A | -2.615717411 |
|  |  | TNFRSF19 | 5.157109748 |
|  |  | TNFRSF9 | -1.105281024 |
|  |  | TNFSF10 | 4.005072678 |
|  |  | TNFSF13 | -1.59877563 |
|  |  | TNIP2 | -1.087458855 |
|  |  | TNIP3 | 1.989617135 |
|  |  | TNK1 | -1.361191296 |
|  |  | TNPO1P3 | -2.848430682 |
|  |  | TNRC18 | -1.160997546 |
|  |  | TNRC6C | 1.566923435 |
|  |  | TNS1 | 5.498412151 |
|  |  | TNS2 | 1.380909327 |
|  |  | TNS3 | -1.810727064 |
|  |  | TNXB | -1.167632198 |
|  |  | TOB1 | 1.427366924 |
|  |  | TOP2A | 1.350176155 |
|  |  | TOR4A | -2.40616721 |
|  |  | TOX | 2.315086061 |
|  |  | TP53I11 | -1.694442113 |
|  |  | TP53INP2 | 2.38589894 |
|  |  | TP63 | 1.292336172 |
|  |  | TPBG | -1.35279544 |
|  |  | TPBGL | 1.839053717 |
|  |  | TPD52L1 | 1.078842813 |
|  |  | TPP1 | 1.062972333 |
|  |  | TPRA1 | -1.486130867 |
|  |  | TPRG1 | -1.72379413 |
|  |  | TPT1P8 | 5.46567001 |
|  |  | TPTE2P1 | 1.174517529 |
|  |  | TPTEP1 | 5.769506719 |
|  |  | TPTEP2 | 1.060633596 |
|  |  | TRABD2A | 5.386704726 |
|  |  | TRABD2B | 5.040502834 |
|  |  | TRAF1 | -2.081853768 |
|  |  | TRAF4 | -1.048166664 |
|  |  | TRAM1L1 | 1.994867797 |
|  |  | TRAPPC3L | 4.753824628 |
|  |  | TREH | -4.616807361 |
|  |  | TREM1 | 5.905725009 |
|  |  | TREML3P | 3.698313474 |
|  |  | TRG-AS1 | 2.349528831 |
|  |  | TRIB1 | -1.249369242 |
|  |  | TRIB2 | 4.555670141 |
|  |  | TRIB3 | -1.717756058 |
|  |  | TRIL | 5.558185003 |
|  |  | TRIM2 | 2.547294076 |
|  |  | TRIM22 | 3.117424344 |
|  |  | TRIM24 | 1.260096038 |
|  |  | TRIM25 | -1.396732524 |
|  |  | TRIM29 | -1.821397773 |
|  |  | TRIM36 | -1.083754472 |
|  |  | TRIM45 | 1.777807167 |
|  |  | TRIM47 | -1.300918741 |
|  |  | TRIM67 | -2.640066546 |
|  |  | TRIM69 | 1.121652525 |
|  |  | TRIML2 | -1.618122534 |
|  |  | TRIO | -1.819933387 |
|  |  | TRMT9B | 4.080499526 |
|  |  | TRNP1 | -5.005982887 |
|  |  | TRO | 1.837913641 |
|  |  | TROAP | 1.448194745 |
|  |  | TRPA1 | 4.623614267 |
|  |  | TRPC3 | 4.582660569 |
|  |  | TRPC4 | 10.77871914 |
|  |  | TRPC6 | 1.413929663 |
|  |  | TRPM2 | -2.565151152 |
|  |  | TRPM3 | 8.573648962 |
|  |  | TRPM6 | 1.483672573 |
|  |  | TRPS1 | 2.379363485 |
|  |  | TSHZ2 | 7.680008459 |
|  |  | TSKU | 1.80155483 |
|  |  | TSPAN11 | -2.026027041 |
|  |  | TSPAN12 | 2.000588353 |
|  |  | TSPAN14 | -1.676026283 |
|  |  | TSPAN15 | -2.534098642 |
|  |  | TSPAN18 | 3.345183795 |
|  |  | TSPAN33 | -2.354884479 |
|  |  | TSPAN5 | -1.354421645 |
|  |  | TSPAN7 | 3.037959146 |
|  |  | TSPAN8 | 9.024099481 |
|  |  | TSPYL4 | 1.651107613 |
|  |  | TSSK5P | -1.490256865 |
|  |  | TSSK6 | -1.282319387 |
|  |  | TTC16 | -4.951138914 |
|  |  | TTC19 | -1.081746244 |
|  |  | TTC25 | -1.288537626 |
|  |  | TTC39C | 1.581518162 |
|  |  | TTC39C-AS1 | 3.395806942 |
|  |  | TTC3P1 | -1.148881975 |
|  |  | TTC4 | -1.470596838 |
|  |  | TTC6 | -2.554908459 |
|  |  | TTC7A | -1.00013951 |
|  |  | TTC9 | -1.369502146 |
|  |  | TTK | 1.158331536 |
|  |  | TTLL7 | 1.540662635 |
|  |  | TTTY14 | -1.726945222 |
|  |  | TTTY15 | -1.137694969 |
|  |  | TUB | 1.006211961 |
|  |  | TUBA4A | -1.732632021 |
|  |  | TUBAL3 | -2.805611438 |
|  |  | TUBB2BP1 | -4.485180047 |
|  |  | TUBB4B | 1.083527113 |
|  |  | TUFT1 | -2.425283266 |
|  |  | TUSC3 | 1.049466798 |
|  |  | TVP23C | -1.175605339 |
|  |  | TXNDC16 | 2.151960383 |
|  |  | TXNIP | -2.975578487 |
|  |  | TXNL4B | -1.067373598 |
|  |  | TYMP | 1.298402865 |
|  |  | TYRP1 | 5.493121367 |
|  |  | U91328.1 | 1.461337426 |
|  |  | UAP1L1 | 1.876692973 |
|  |  | UBA7 | 3.049921927 |
|  |  | UBASH3B | -4.561085734 |
|  |  | UBBP4 | 3.873771553 |
|  |  | UBE2C | 1.837519609 |
|  |  | UBE2E3 | 1.083634995 |
|  |  | UBE2QL1 | -3.315435269 |
|  |  | UBE2SP1 | 1.453437107 |
|  |  | UBXN11 | -1.051112653 |
|  |  | UCN2 | -1.596627386 |
|  |  | UCP2 | -1.473403705 |
|  |  | UCP3 | 1.158306823 |
|  |  | UFSP1 | -1.24496553 |
|  |  | UGCG | -1.767564943 |
|  |  | UGGT2 | 1.081803083 |
|  |  | UGT1A1 | -4.716992417 |
|  |  | UGT1A7 | -5.397131573 |
|  |  | UGT2B11 | -4.610182921 |
|  |  | UGT2B7 | -5.676662791 |
|  |  | UGT8 | 2.541018033 |
|  |  | UHRF1 | -1.240557987 |
|  |  | ULBP1 | -1.365640125 |
|  |  | ULK2 | -1.309880787 |
|  |  | ULK4 | -1.089226174 |
|  |  | UNC13A | -1.318943014 |
|  |  | UNC13D | -5.231901919 |
|  |  | UNC5B | 1.280509083 |
|  |  | UNC5C | 2.829735027 |
|  |  | UNC79 | 1.704416673 |
|  |  | UNC80 | 1.585429886 |
|  |  | UNCX | 5.537438787 |
|  |  | UPF3B | -1.376998995 |
|  |  | UPK2 | -2.013634849 |
|  |  | UPP1 | -1.737913168 |
|  |  | UQCC3 | -1.211338548 |
|  |  | UQCR10 | 1.3378841 |
|  |  | UQCRB | 1.12463952 |
|  |  | USH1C | 4.279867901 |
|  |  | USP2 | -3.325956425 |
|  |  | USP2-AS1 | -2.243848029 |
|  |  | USP44 | -3.33656216 |
|  |  | USP51 | 3.128945416 |
|  |  | USP53 | -2.496217314 |
|  |  | UST | 3.037605033 |
|  |  | UTP25 | -1.20057657 |
|  |  | VAMP1 | 1.102807243 |
|  |  | VAMP8 | -2.069500258 |
|  |  | VANGL2 | 2.652729274 |
|  |  | VASH2 | 1.166118615 |
|  |  | VAT1 | -1.244608611 |
|  |  | VAT1L | 4.686418436 |
|  |  | VAV1 | -5.247883108 |
|  |  | VAV3 | 3.066401172 |
|  |  | VCAM1 | 5.828908105 |
|  |  | VCAN | 2.362903334 |
|  |  | VEGFC | -1.371545005 |
|  |  | VEGFD | 2.839266277 |
|  |  | VEPH1 | 2.916840132 |
|  |  | VEZT | -1.505232335 |
|  |  | VGF | 1.723966316 |
|  |  | VGLL3 | 1.232086733 |
|  |  | VIPR2 | 3.555102698 |
|  |  | VIT | 4.858831179 |
|  |  | VLDLR-AS1 | -2.340187315 |
|  |  | VMP1 | -1.10360512 |
|  |  | VN1R53P | -4.79287699 |
|  |  | VNN3 | 3.817056879 |
|  |  | VPS37C | -1.339489428 |
|  |  | VPS37D | -1.397755067 |
|  |  | VPS9D1-AS1 | -1.106227993 |
|  |  | VSIR | -3.9911978 |
|  |  | VSNL1 | 4.388878963 |
|  |  | VSTM1 | -5.459639354 |
|  |  | VSTM2L | -4.325489461 |
|  |  | VTN | -1.664024683 |
|  |  | VWA5A | 4.701506812 |
|  |  | VWA5B2 | -1.750153625 |
|  |  | VWA7 | -1.154457814 |
|  |  | VWCE | -1.624324528 |
|  |  | VWDE | -1.139587747 |
|  |  | WASF3 | 5.154258368 |
|  |  | WDFY3-AS2 | 1.327827602 |
|  |  | WDR35 | -1.070986022 |
|  |  | WDR86 | 2.768063937 |
|  |  | WDR86-AS1 | 4.481408602 |
|  |  | WDR88 | 1.184730249 |
|  |  | WFDC1 | 4.923478015 |
|  |  | WFDC21P | 1.178880443 |
|  |  | WIF1 | 12.35697971 |
|  |  | WIPF3 | -1.707522087 |
|  |  | WIPI1 | 1.800246481 |
|  |  | WISP1 | 9.907897156 |
|  |  | WISP2 | 1.413392658 |
|  |  | WNK3 | 1.609343719 |
|  |  | WNT10B | -2.63675806 |
|  |  | WNT16 | 6.198632556 |
|  |  | WNT4 | 6.839214922 |
|  |  | WNT5A | 4.114608967 |
|  |  | WNT5A-AS1 | 3.524512019 |
|  |  | WNT5B | 2.881894756 |
|  |  | WNT6 | 7.639051392 |
|  |  | WNT7B | -8.211597996 |
|  |  | WNT9A | -2.007819759 |
|  |  | WSCD1 | 1.506409011 |
|  |  | WSCD2 | 4.557975543 |
|  |  | WT1-AS | -1.523742045 |
|  |  | WTIP | -2.447554373 |
|  |  | WWC1 | -2.366378433 |
|  |  | WWC3 | -1.627675209 |
|  |  | XBP1 | -1.124750598 |
|  |  | XDH | -3.487525062 |
|  |  | XKR4 | 2.629902308 |
|  |  | XKR6 | 2.303963308 |
|  |  | XKR8 | -5.34671726 |
|  |  | XKRX | -2.095889209 |
|  |  | XPNPEP3 | 1.0538434 |
|  |  | XXYLT1 | 1.302429397 |
|  |  | XYLT1 | 4.460548493 |
|  |  | YAP1 | -1.211306896 |
|  |  | YDJC | -1.935220484 |
|  |  | YEATS4 | -1.330392135 |
|  |  | YIPF3 | 1.167004034 |
|  |  | YKT6 | -1.014967244 |
|  |  | YRDC | -1.002362869 |
|  |  | YY2 | -1.390703413 |
|  |  | Z82186.1 | -5.225095802 |
|  |  | Z82188.2 | 4.492842248 |
|  |  | Z95331.1 | -1.105556973 |
|  |  | Z97180.1 | -1.148056325 |
|  |  | Z97200.1 | 4.689581768 |
|  |  | Z97985.1 | -3.704291269 |
|  |  | ZBED2 | -3.310127362 |
|  |  | ZBTB21 | -1.025998178 |
|  |  | ZBTB32 | -2.228012558 |
|  |  | ZBTB42 | 1.143004809 |
|  |  | ZBTB7C | 3.249842398 |
|  |  | ZC3H12C | -1.489510473 |
|  |  | ZC3HAV1 | 1.507138113 |
|  |  | ZCCHC12 | 2.83929087 |
|  |  | ZCCHC24 | 2.361328578 |
|  |  | ZCCHC8 | -1.118398248 |
|  |  | ZDHHC11B | 1.624304051 |
|  |  | ZDHHC23 | 1.072716951 |
|  |  | ZDHHC9 | -1.120225491 |
|  |  | ZEB2 | 1.127822034 |
|  |  | ZFAND2A | -1.579305162 |
|  |  | ZFAS1 | -1.555347386 |
|  |  | ZFHX4 | 1.49147129 |
|  |  | ZFP28 | -1.333869547 |
|  |  | ZFP3 | -1.996290691 |
|  |  | ZFP30 | -4.361978788 |
|  |  | ZFP69B | -1.242845441 |
|  |  | ZFP82 | -2.838195058 |
|  |  | ZFPM2 | 2.418852853 |
|  |  | ZFYVE19 | 1.542231104 |
|  |  | ZG16B | -5.987792395 |
|  |  | ZMAT3 | 1.589962947 |
|  |  | ZMAT4 | 2.189634487 |
|  |  | ZMIZ1-AS1 | 1.864882666 |
|  |  | ZNF140 | -1.204529839 |
|  |  | ZNF141 | -2.976143791 |
|  |  | ZNF185 | -2.25248449 |
|  |  | ZNF204P | 6.587276508 |
|  |  | ZNF217 | -1.275539659 |
|  |  | ZNF229 | -2.663224646 |
|  |  | ZNF239 | -1.405099458 |
|  |  | ZNF254 | -1.029787324 |
|  |  | ZNF256 | -7.11855506 |
|  |  | ZNF26 | -1.151299235 |
|  |  | ZNF264 | -1.770566543 |
|  |  | ZNF267 | -1.006566957 |
|  |  | ZNF274 | -2.088374629 |
|  |  | ZNF28 | -4.351083584 |
|  |  | ZNF285 | -1.954705816 |
|  |  | ZNF296 | -1.60999408 |
|  |  | ZNF30 | -1.366602209 |
|  |  | ZNF304 | -1.070998913 |
|  |  | ZNF318 | 1.022277703 |
|  |  | ZNF329 | -1.68524072 |
|  |  | ZNF341-AS1 | -4.515641483 |
|  |  | ZNF345 | -4.603530097 |
|  |  | ZNF385A | 1.264823655 |
|  |  | ZNF385C | -1.799781421 |
|  |  | ZNF385D | 8.210953338 |
|  |  | ZNF408 | -1.208109657 |
|  |  | ZNF418 | -2.725170662 |
|  |  | ZNF420 | -1.077369101 |
|  |  | ZNF423 | 1.706350207 |
|  |  | ZNF425 | -1.35687911 |
|  |  | ZNF426 | -1.723144712 |
|  |  | ZNF43 | -1.245365253 |
|  |  | ZNF460 | -1.188020588 |
|  |  | ZNF467 | 3.121789399 |
|  |  | ZNF469 | 1.283416995 |
|  |  | ZNF470 | -1.444867461 |
|  |  | ZNF471 | -3.928745299 |
|  |  | ZNF48 | -1.051614382 |
|  |  | ZNF485 | -1.106493249 |
|  |  | ZNF488 | -1.040520814 |
|  |  | ZNF503 | 2.080220133 |
|  |  | ZNF513 | -1.027181058 |
|  |  | ZNF521 | 7.229413252 |
|  |  | ZNF529 | -1.389845922 |
|  |  | ZNF529-AS1 | -1.348184779 |
|  |  | ZNF536 | 6.63457521 |
|  |  | ZNF542P | -3.109728913 |
|  |  | ZNF543 | -1.504771757 |
|  |  | ZNF544 | -1.957253635 |
|  |  | ZNF547 | -1.453470188 |
|  |  | ZNF568 | -3.375496199 |
|  |  | ZNF569 | -1.431630434 |
|  |  | ZNF571 | -3.682174449 |
|  |  | ZNF573 | -1.728225551 |
|  |  | ZNF582 | -1.362642447 |
|  |  | ZNF582-AS1 | -2.19230154 |
|  |  | ZNF583 | -1.195387187 |
|  |  | ZNF605 | -1.047301729 |
|  |  | ZNF607 | -4.37104459 |
|  |  | ZNF611 | -3.040808485 |
|  |  | ZNF622 | -1.177387977 |
|  |  | ZNF630 | -1.538726305 |
|  |  | ZNF671 | -1.415327273 |
|  |  | ZNF674 | -1.482648419 |
|  |  | ZNF675 | -2.700472735 |
|  |  | ZNF699 | -1.632102421 |
|  |  | ZNF701 | -3.056891649 |
|  |  | ZNF704 | 2.910452579 |
|  |  | ZNF708 | -5.720812116 |
|  |  | ZNF718 | -4.682078985 |
|  |  | ZNF726 | -5.850827263 |
|  |  | ZNF736 | -5.101543022 |
|  |  | ZNF738 | -5.567214286 |
|  |  | ZNF75A | -1.00730295 |
|  |  | ZNF773 | -1.005266071 |
|  |  | ZNF790-AS1 | -5.603503918 |
|  |  | ZNF792 | 1.136482135 |
|  |  | ZNF799 | -1.619224619 |
|  |  | ZNF804A | 1.851064236 |
|  |  | ZNF829 | -2.786707498 |
|  |  | ZNF83 | -5.292902504 |
|  |  | ZNF860 | 1.565078657 |
|  |  | ZNF879 | -1.226412224 |
|  |  | ZNF880 | -1.767166877 |
|  |  | ZNF891 | -1.048925652 |
|  |  | ZNF91 | -3.814016691 |
|  |  | ZP1 | 2.947329023 |
|  |  | ZP3 | -1.171701863 |
|  |  | ZPR1 | -1.373158849 |
|  |  | ZSCAN16 | -1.210498243 |
|  |  | ZSCAN16-AS1 | 1.789510546 |
|  |  | ZSCAN18 | -5.691971747 |
|  |  | ZSCAN21 | -1.528022281 |
|  |  | ZSCAN31 | 3.179565731 |
|  |  | ZSWIM1 | 1.161192085 |
|  |  | ZSWIM3 | 1.427278025 |
|  |  | ZSWIM4 | -1.799090853 |
|  |  | ZSWIM6 | 1.470464743 |
|  |  | ZXDB | -1.204244035 |

Table S2. Gene expression log2FoldChange for EZH2ko vs TFK1-WT and SUZ12ko vs TFK1-WT collectively.

| gene_name | EZH2ko vs TFK1-WT log2FoldChange | SUZ12ko- vs TFK1-WT log2FoldChange |
| --- | --- | --- |
| A2M-AS1 | 1.077503505 | 2.0194 |
| A4GALT | 1.728532599 | 3.303421 |
| AARD | 4.976965206 | 4.662494 |
| ABCA1 | 1.688454787 | 1.893773 |
| ABCA12 | -1.283464233 | -3.17144 |
| ABCA5 | 1.029574076 | 1.299115 |
| ABCA8 | 3.774690164 | 5.793923 |
| ABCA9 | 2.068967249 | 2.559296 |
| ABCC9 | 2.455063199 | 2.773393 |
| ABCG1 | -1.272092539 | 1.024576 |
| ABI3BP | 4.779732062 | 6.086043 |
| ABLIM3 | 2.074239511 | 2.435093 |
| AC002401.4 | 1.001507639 | -2.7299 |
| AC004585.1 | -1.105715929 | -1.21133 |
| AC004656.1 | 1.295794023 | 1.830124 |
| AC004704.1 | 7.963497379 | 7.990559 |
| AC004706.3 | -1.742920313 | -1.95637 |
| AC004816.1 | -1.021161286 | -1.17321 |
| AC005336.1 | 1.634784135 | 1.562238 |
| AC006262.1 | -2.670889254 | -1.73632 |
| AC007066.2 | 1.08972097 | 1.303863 |
| AC007405.3 | 1.568192533 | 1.849362 |
| AC007686.3 | 1.217665338 | 1.780919 |
| AC007743.1 | -1.808405103 | -1.31496 |
| AC008440.3 | -1.150588237 | -4.67861 |
| AC010343.3 | 1.632502223 | -4.74468 |
| AC010754.1 | 3.299936898 | 4.003082 |
| AC010890.1 | 1.871833468 | 1.519081 |
| AC012313.8 | -1.031441229 | -1.40357 |
| AC017048.3 | 3.370008255 | 3.718809 |
| AC022239.1 | 3.82360154 | 1.665033 |
| AC022613.1 | -1.083731041 | -1.32764 |
| AC023043.3 | 3.234367212 | 3.370765 |
| AC025884.2 | -1.289951569 | -1.21257 |
| AC026368.1 | 1.570346501 | 2.163617 |
| AC034213.1 | -1.253939297 | -4.50669 |
| AC040970.1 | -1.015062529 | -1.24151 |
| AC062029.1 | 1.237536194 | 1.692837 |
| AC079949.1 | -1.287094504 | -2.72054 |
| AC080038.1 | 1.754567765 | 2.118649 |
| AC087482.1 | -1.362992515 | -2.25106 |
| AC087623.1 | -1.141845927 | -2.10311 |
| AC090001.1 | -2.443137441 | -3.06161 |
| AC090409.1 | -2.895238189 | -2.21014 |
| AC090502.1 | -1.58727643 | -1.76067 |
| AC091133.2 | -1.130748673 | -1.39084 |
| AC091212.1 | -1.291557501 | -4.52892 |
| AC091948.1 | 8.000708119 | 9.358698 |
| AC096537.1 | -2.483639385 | -3.65272 |
| AC098617.1 | -1.94642614 | -2.54116 |
| AC099850.3 | 1.012022608 | 1.532887 |
| AC103702.2 | -1.230576971 | -1.22969 |
| AC104072.1 | 5.606197256 | 4.716555 |
| AC104137.1 | 1.887900928 | 2.97176 |
| AC106869.1 | -2.082084698 | -1.9495 |
| AC112236.2 | -1.995598806 | -2.10576 |
| AC125232.2 | -1.574233726 | -1.82657 |
| AC127070.1 | -1.896463516 | -3.25809 |
| AC127502.2 | 1.108566081 | 1.489321 |
| AC129492.1 | -1.224271615 | -1.53439 |
| AC137936.2 | 1.002637942 | -2.08025 |
| AC139718.1 | 5.963691533 | 5.647284 |
| AC211433.1 | 2.756337495 | 4.289763 |
| AC239859.6 | 1.948433632 | 1.824838 |
| AC243562.2 | 1.83564315 | 1.613263 |
| AC244197.2 | -2.612753711 | -2.90973 |
| ACAP1 | -1.310466429 | -1.34694 |
| ACBD4 | -1.06083481 | -1.3016 |
| ACHE | -2.22263214 | -1.0952 |
| ACKR3 | 4.796999012 | 5.166765 |
| ACOX2 | 1.504238992 | 2.293774 |
| ACSL4 | 1.927912736 | 2.002559 |
| ACSS3 | -3.561752973 | 3.740413 |
| ACVR1C | 2.954742215 | 2.678844 |
| ADAM11 | -1.241646655 | -1.78137 |
| ADAM12 | 2.053318237 | 1.839134 |
| ADAM23 | 1.626064219 | 2.561601 |
| ADAMTS1 | 2.733149102 | 5.271247 |
| ADAMTS12 | 2.499423875 | 2.002934 |
| ADAMTS16 | -1.384376873 | -1.39918 |
| ADAMTS2 | -2.480136123 | -3.81463 |
| ADAMTS6 | 4.999069755 | 3.867089 |
| ADAMTS8 | -2.243790377 | 1.282077 |
| ADAMTSL1 | 4.441161482 | 8.870152 |
| ADAMTSL4-AS1 | 1.058632333 | -2.39536 |
| ADAP1 | -1.170628216 | -1.3687 |
| ADARB1 | 2.216932174 | 2.794716 |
| ADD3 | 2.044525169 | 4.005383 |
| ADGRB1 | -1.177570922 | 1.551094 |
| ADGRE2 | -2.204046781 | -1.9369 |
| ADGRL3 | 1.401693878 | 3.086923 |
| ADORA2B | -1.042989212 | -2.16924 |
| ADRA1B | -1.509993999 | -1.51775 |
| ADRA2A | 5.033384859 | 4.680727 |
| ADRB2 | 1.543187423 | 2.97504 |
| AF121898.1 | 4.219623134 | 7.389578 |
| AFAP1L2 | -1.443048394 | -3.22834 |
| AFF3 | 4.020658445 | 3.894051 |
| AFP | 2.690519004 | 3.792906 |
| AGPAT4 | 1.612144786 | 1.453304 |
| AGTR1 | 3.051633854 | 3.078531 |
| AK5 | 2.193327129 | -1.06133 |
| AKAP12 | -1.027469559 | -3.52495 |
| AKNAD1 | -2.451313724 | -3.96937 |
| AKR1C1 | 2.27562055 | 1.764238 |
| AL021392.1 | -1.988861596 | -1.84115 |
| AL023284.4 | 1.838322129 | 1.831318 |
| AL137003.2 | 1.227227933 | 1.004954 |
| AL139393.2 | 2.19224048 | 1.795677 |
| AL353147.1 | 6.453323492 | 5.713691 |
| AL355032.1 | -1.214409245 | -1.44339 |
| AL356274.2 | 3.295295214 | 3.292513 |
| AL356414.1 | 3.956481564 | 3.147743 |
| AL358334.2 | 1.324485515 | 3.945945 |
| AL390726.6 | -2.971939268 | -4.8259 |
| AL391056.1 | 2.213097237 | 4.005358 |
| AL391427.1 | 1.714881016 | 1.81219 |
| AL445309.1 | 1.599005797 | 1.754586 |
| AL513318.2 | 1.113409174 | 2.38169 |
| AL845552.2 | 3.380182941 | 3.550248 |
| ALG1L | -1.448646168 | -1.62825 |
| ALG1L7P | -1.439339953 | -2.78601 |
| ALOXE3 | -1.279118991 | -5.892 |
| ALPK2 | 1.000955431 | -1.32357 |
| ALX4 | 1.199605769 | 2.733353 |
| AMOT | 2.554156501 | 6.729922 |
| AMPH | 2.0430607 | 4.564279 |
| ANGPT1 | 2.76472747 | 4.638211 |
| ANK1 | -2.654192564 | -2.99898 |
| ANKRD1 | -1.200406921 | -6.90153 |
| ANKRD18A | -1.262811469 | -1.1074 |
| ANKRD2 | -1.103951105 | -2.44597 |
| ANKRD20A5P | -1.082747405 | -3.99094 |
| ANKRD44 | 1.20403045 | 2.040385 |
| ANKS1B | 2.263940739 | 2.399715 |
| ANPEP | 3.710381348 | -2.55647 |
| ANTXR1 | 1.547327024 | 2.426158 |
| ANXA10 | 2.814210057 | 1.042226 |
| ANXA13 | 1.692139437 | -7.88656 |
| ANXA6 | 1.104031618 | 1.532624 |
| AP000662.1 | -1.673963762 | -2.36547 |
| AP001542.3 | 2.961209729 | 3.013102 |
| AP001972.5 | 1.306972579 | 1.256041 |
| AP002852.1 | 2.724184394 | 4.789758 |
| AP003119.3 | 1.826414767 | 1.781829 |
| AP005233.2 | -1.418673857 | -7.97136 |
| APBB1IP | 1.773759202 | -2.04719 |
| APOE | 1.273016783 | 3.514655 |
| AREG | -1.57312837 | -6.60905 |
| ARHGAP28 | 1.13042985 | 2.022769 |
| ARHGAP30 | -1.041346873 | -2.03822 |
| ARHGDIG | -2.64715708 | -1.36283 |
| ARHGEF6 | 4.118265585 | 4.248357 |
| ARL4C | -1.505769349 | -1.60925 |
| ARLNC1 | 2.687635718 | 2.739432 |
| ARMC12 | -1.460412299 | -1.72836 |
| ARMC3 | -2.234019436 | -1.33274 |
| ARMC4 | 3.169473626 | 3.214197 |
| ARMCX4 | 2.293785584 | 3.119768 |
| ARRDC4 | -1.396417284 | -2.46992 |
| ARSE | -1.16895602 | -2.13242 |
| ARVCF | -2.363614618 | -2.15968 |
| ARX | -1.286748753 | -1.11415 |
| ASS1 | 1.769981526 | 1.889179 |
| ATL1 | 1.138782169 | 1.222044 |
| ATP8B1 | -1.222462232 | -3.25931 |
| B3GNT3 | 1.098962373 | -1.34079 |
| B4GALNT2 | -5.025253582 | -2.65544 |
| BAHCC1 | -1.634661145 | -1.58328 |
| BAIAP3 | -1.695061026 | -1.07567 |
| BARX1 | -3.719230329 | -1.12123 |
| BCHE | 1.887424239 | 3.870096 |
| BCL2L10 | 1.438443045 | 1.615852 |
| BCL2L11 | 1.490882508 | 1.898694 |
| BCO1 | -1.410504042 | -4.14575 |
| BDKRB1 | 2.174760529 | 1.170829 |
| BDKRB2 | 1.646883784 | 2.014949 |
| BEGAIN | 1.452553597 | 1.617897 |
| BIRC7 | -1.075673831 | -2.14557 |
| BMP6 | -1.396221592 | -1.82758 |
| BMP7 | -3.583924966 | 1.362305 |
| BMPER | -1.606026235 | 1.527566 |
| BNC1 | -3.062996083 | -2.44026 |
| BX005266.2 | -1.325146575 | -2.2282 |
| BX088651.4 | -1.378377448 | -2.13692 |
| BX322234.2 | -3.117852056 | -5.51588 |
| C10orf90 | 3.065496291 | 1.601563 |
| C11orf45 | -1.090195038 | -3.10573 |
| C13orf46 | 1.600695529 | 2.293452 |
| C15orf48 | 1.653382497 | 3.64223 |
| C17orf102 | -4.432716752 | -5.34953 |
| C19orf33 | -1.466113921 | -3.42055 |
| C1GALT1C1 | 1.46154871 | 2.093505 |
| C1orf116 | 1.26258562 | -6.61819 |
| C1orf21 | 3.556140197 | 2.616278 |
| C1QL4 | -2.036937555 | -2.62592 |
| C1R | 1.504231963 | 3.80657 |
| C1S | 1.594492834 | 3.804564 |
| C3orf52 | -1.076832316 | -1.4657 |
| C5AR1 | 1.087962066 | 4.143256 |
| CA2 | 1.25976581 | 4.16123 |
| CA8 | -3.341935688 | 1.745931 |
| CACNA1B | -4.442209814 | -5.35859 |
| CACNA1H | -1.290680845 | -2.05043 |
| CACNG4 | -1.252318784 | -1.05568 |
| CACNG6 | -2.523720669 | -7.89941 |
| CACNG7 | -2.74835743 | -4.69754 |
| CACNG8 | -1.809983968 | -4.88471 |
| CADM2 | -4.604720081 | 2.212261 |
| CALCB | 1.614737852 | 2.740964 |
| CALHM5 | 1.569619277 | 4.004451 |
| CAMK4 | 5.139973325 | 4.346968 |
| CAMKV | -2.211993728 | -2.71712 |
| CARMN | 7.087222093 | 3.470231 |
| CASC19 | 1.482253375 | -5.87674 |
| CASP1 | 3.425428232 | 5.562801 |
| CASP4 | 1.154554955 | 1.56576 |
| CAT | 1.095573223 | 1.251955 |
| CATSPERG | -1.292590801 | -2.28275 |
| CCDC102B | 1.990316905 | 3.328578 |
| CCDC114 | -1.986118829 | -1.0508 |
| CCDC184 | -1.044740582 | -1.90283 |
| CCDC189 | -1.720685345 | -2.78087 |
| CCND2 | -3.897550862 | 7.133672 |
| CCNJL | -1.754063949 | -1.42031 |
| CCR1 | 5.645289 | 6.500461 |
| CD1D | 1.349937545 | 3.272592 |
| CD302 | 1.384718852 | 1.955714 |
| CD36 | 2.247787653 | 2.602634 |
| CD38 | -2.376854216 | -2.15924 |
| CD55 | 1.421129226 | 2.259041 |
| CD70 | -2.298948012 | -1.63582 |
| CD74 | 1.629087955 | 6.995324 |
| CDC42BPG | -1.381910631 | -2.60955 |
| CDCP1 | -1.684605429 | -3.02992 |
| CDH17 | 1.841004363 | -6.5668 |
| CDH19 | -3.218210795 | 1.978158 |
| CDK14 | 2.254347349 | 2.282063 |
| CDYL2 | 3.188670645 | 3.903205 |
| CEACAM6 | 1.535019614 | -3.85984 |
| CELF5 | -1.176650779 | -1.66965 |
| CENPVL3 | -2.866841731 | 2.616729 |
| CERKL | 2.968990528 | 2.407312 |
| CERS1 | -1.956580674 | -2.81617 |
| CFAP57 | -2.911736181 | -1.59964 |
| CFAP58-DT | 3.374796561 | 4.787991 |
| CFAP65 | -1.4114124 | -2.52921 |
| CFI | 3.549198226 | 6.074105 |
| CGAS | -7.942084388 | -6.99975 |
| CGB8 | -1.347299687 | -8.18323 |
| CHD5 | -1.855823711 | -3.02339 |
| CHL1 | 7.90454287 | 9.887584 |
| CHMP1B | 1.13709058 | 1.738406 |
| CHRDL1 | 5.406778796 | 8.716296 |
| CHRDL2 | 2.856636209 | 5.379738 |
| CHRM3 | -4.480269797 | 2.089468 |
| CHRNB2 | -1.385081274 | -2.16682 |
| CHST4 | 2.726423855 | 6.253751 |
| CHSY3 | 1.57046724 | 3.427646 |
| CLDN1 | -1.221419393 | -3.45746 |
| CLDN2 | 1.50338448 | -1.77063 |
| CLDN3 | -2.450166951 | -2.40621 |
| CLDND1 | 1.019468547 | 1.275278 |
| CLEC2B | 1.984448599 | 2.753133 |
| CLIC2 | 2.439255416 | 4.028855 |
| CLMP | 3.629899354 | 1.105536 |
| CLVS2 | 1.963429041 | 1.956414 |
| CNKSR2 | -2.241762148 | -3.29757 |
| CNR1 | 2.733276846 | 3.059837 |
| CNRIP1 | 1.8096461 | 2.984664 |
| CNTNAP2 | 4.665000097 | 3.764077 |
| CNTNAP3B | -1.786041089 | -1.52865 |
| CNTNAP3P2 | -1.665731085 | -1.11092 |
| COL12A1 | 1.363027288 | 2.846578 |
| COL13A1 | -2.632146881 | -1.73491 |
| COL14A1 | 4.45621095 | 10.79238 |
| COL15A1 | 3.735859378 | 8.695251 |
| COL21A1 | -2.002403587 | -2.67048 |
| COL3A1 | 3.985769675 | 8.612636 |
| COL5A3 | 4.680835025 | 5.483463 |
| COL6A1 | 2.217864548 | 2.325359 |
| COL6A2 | 2.980590182 | 2.32022 |
| COL8A1 | 8.774685249 | 11.89611 |
| CPED1 | 5.497300334 | 7.351301 |
| CPQ | 2.052794495 | 3.419048 |
| CPVL | 2.405009358 | 3.991322 |
| CREB3L1 | 2.89102919 | -2.4099 |
| CREG1 | 1.276771947 | 1.922677 |
| CRISPLD1 | 3.792392079 | 5.148449 |
| CRISPLD2 | 4.003282882 | 2.83807 |
| CRTAC1 | -2.562135393 | 1.132897 |
| CRYBG2 | 2.984328324 | -4.39572 |
| CSF2RA | -2.303837819 | -2.75826 |
| CSRNP3 | 2.178557419 | 3.260523 |
| CT62 | -2.449601954 | -3.83779 |
| CTH | 1.758355623 | 1.984265 |
| CTSA | 1.144791725 | 1.771538 |
| CTSB | 1.1452772 | 1.096323 |
| CTSD | 1.067747066 | 1.473266 |
| CTSO | 1.222218797 | 1.717398 |
| CTXN1 | -2.262052427 | -1.2435 |
| CUL4B | 1.813008098 | 1.422108 |
| CXCL5 | -1.030637481 | -2.62201 |
| CXorf57 | 1.739939073 | 2.696165 |
| CXXC5 | 2.426079372 | 3.24164 |
| CYBRD1 | 1.445078253 | 1.45464 |
| CYP2J2 | 1.71252519 | 2.657161 |
| CYP2U1 | 1.024000922 | 1.34078 |
| CYP2W1 | -1.017626677 | -2.24885 |
| CYP4F11 | 1.619155043 | 1.623075 |
| CYP7B1 | 1.592616093 | 3.316816 |
| DAB2 | 1.815791726 | 3.593694 |
| DDR2 | 5.620420301 | 4.587655 |
| DENND2A | -1.971687968 | -1.9466 |
| DGKI | 4.58856728 | 3.352752 |
| DHCR24 | 1.213617163 | 1.085557 |
| DHFRP1 | 1.047030568 | 1.516171 |
| DHRS2 | -2.724701797 | -2.42273 |
| DIO2 | 1.068084893 | 2.952022 |
| DIO3OS | -1.696423552 | -1.8694 |
| DIRAS2 | 6.231050254 | 7.793115 |
| DKK1 | -1.606848197 | -5.40945 |
| DLG2 | 1.973114724 | 3.833097 |
| DMD | 3.523065457 | 6.316665 |
| DNAAF3 | -1.480681114 | -1.02211 |
| DNAH17-AS1 | 1.984964753 | 2.3175 |
| DNAH5 | -1.299441424 | -4.10169 |
| DNAH7 | -1.514812151 | -2.00829 |
| DNER | 2.728882911 | 4.292587 |
| DNM1P51 | 1.430708623 | 1.162369 |
| DPYSL5 | -1.598340146 | 1.18581 |
| DRC7 | -3.24135301 | -3.45946 |
| DRD1 | -2.638941908 | -5.95405 |
| DSE | 1.51018227 | 1.46398 |
| DSEL | 2.320449753 | 5.444556 |
| DUSP8 | -1.167556108 | -1.11396 |
| DYNC1I1 | 3.658023806 | 7.340001 |
| DYSF | -1.758927732 | -1.56517 |
| EBF1 | 2.115810132 | 2.30531 |
| ECM1 | 1.346328114 | 2.576711 |
| EDIL3 | 3.282103327 | 4.183143 |
| EDN1 | -1.413856905 | -3.9708 |
| EDNRA | 1.780390613 | 5.667194 |
| EDNRB | 5.055825338 | 9.537274 |
| EEF1A2 | -1.561582243 | -1.394 |
| EFEMP1 | 2.808373561 | 3.172789 |
| EFNA1 | 1.467023363 | 2.107632 |
| EFNB3 | -1.150897763 | 1.638212 |
| EGF | 2.192022252 | 4.587181 |
| EGFR-AS1 | -2.072573858 | -4.36649 |
| ELF3 | -1.969927453 | -6.81806 |
| EMC6 | -1.145996456 | -1.11219 |
| EN1 | -2.264417877 | -2.17048 |
| ENOX1 | 1.641425797 | 1.792506 |
| ENPP4 | 1.012815098 | 1.669154 |
| EPHA10 | -1.326096476 | -1.94565 |
| EPHA2 | -1.175047733 | -3.57585 |
| EPHA5 | 2.098629776 | 3.097646 |
| EPOP | -1.257926108 | -1.58611 |
| EPS8L3 | 1.612582696 | -4.4078 |
| ERBB3 | 1.51543586 | -1.29496 |
| EREG | -1.709424297 | -5.63699 |
| ERGIC1 | 1.467117082 | 1.867029 |
| ERRFI1 | -1.385277934 | -3.49906 |
| ETS1 | -1.221869418 | -3.50838 |
| EVA1C | 1.093975456 | 1.133086 |
| EXOC3L2 | -1.647622083 | -1.67812 |
| EXOC3L4 | -1.084411782 | -1.38994 |
| EXPH5 | -1.918158069 | -1.90351 |
| EYA2 | -1.995526198 | -3.31277 |
| F2RL1 | -1.333232987 | -2.86277 |
| F2RL2 | 1.427824323 | 1.890875 |
| F5 | 6.411322507 | 3.127962 |
| FAM13C | 3.386888359 | 6.826037 |
| FAM155A | -2.669894557 | 6.210536 |
| FAM160A1 | 1.385277682 | 2.939175 |
| FAM174B | -1.463554629 | -1.13555 |
| FAM198B | 1.848357113 | 1.45322 |
| FAM19A5 | -1.516643684 | -3.69242 |
| FAM224B | -4.025131862 | -2.97313 |
| FAM43A | 1.919992748 | 2.746067 |
| FAM83A | 2.242546123 | 1.247274 |
| FAP | 6.208474175 | 3.213201 |
| FBLN1 | 1.27139073 | 1.548617 |
| FBLN2 | 3.65716569 | 1.486059 |
| FBN1 | 1.737956346 | 4.256728 |
| FBXL7 | 2.139842867 | 3.504517 |
| FDXR | -1.327472112 | -1.25349 |
| FENDRR | 8.214521705 | 11.56264 |
| FGA | -1.346239947 | -2.22758 |
| FGF12 | 1.186549609 | 1.625103 |
| FGF9 | 5.045603298 | 9.365426 |
| FHDC1 | 3.451675572 | 4.544318 |
| FIBIN | 2.188917151 | 2.035625 |
| FILIP1 | 1.70436933 | 3.851131 |
| FJX1 | -1.614980208 | -1.06061 |
| FKBP7 | 1.394621308 | 3.201898 |
| FLNC | 3.854300261 | -3.59147 |
| FLRT2 | -3.699834809 | 6.83626 |
| FLRT3 | -1.041379845 | -4.34882 |
| FN1 | 3.035564657 | 3.122913 |
| FNDC1 | 1.659359769 | 8.614469 |
| FNDC3A | 1.036093425 | 1.735378 |
| FOXA2 | -1.788181607 | -3.78699 |
| FOXA3 | -1.929318236 | -3.84171 |
| FOXF1 | 9.12184453 | 12.2034 |
| FOXL2 | -3.42875069 | -2.7454 |
| FOXL2NB | -2.326609346 | -1.31207 |
| FOXO1 | 1.253913639 | 1.3441 |
| FOXP2 | 2.267529523 | 5.637704 |
| FPR1 | 1.013243696 | 1.520752 |
| FRAS1 | -1.42587382 | -1.09535 |
| FRMD5 | -1.216433776 | -3.06686 |
| FSIP1 | -1.065932137 | -1.52882 |
| FSTL1 | 2.596321881 | 4.218572 |
| FSTL4 | -1.334594751 | 3.193897 |
| FYN | 3.090696962 | 4.521299 |
| GAB1 | 1.174947611 | 2.404426 |
| GABRA2 | 6.896155429 | 7.084137 |
| GABRA5 | 1.372009656 | 1.046052 |
| GABRD | -4.084154122 | 1.196143 |
| GABRQ | 2.222325854 | 4.244989 |
| GADD45A | -1.036093429 | -3.80165 |
| GAL3ST1 | 1.036163393 | -4.6157 |
| GALNT15 | 4.591436828 | 6.500812 |
| GALNT16 | -1.600613009 | 1.343689 |
| GALNT17 | 1.27834029 | -5.39427 |
| GALNT18 | -1.100492201 | -2.09057 |
| GALNT2 | 1.21146137 | 2.691805 |
| GAP43 | 5.101095873 | 9.413076 |
| GAS1 | 8.282138139 | 9.696288 |
| GATA2 | 1.314171411 | 3.657945 |
| GATA3 | -1.132952918 | -1.83355 |
| GATA6 | -1.22273761 | -3.05749 |
| GATA6-AS1 | -1.895853773 | -3.85087 |
| GBP1 | 2.596269361 | 2.51015 |
| GCOM1 | 1.019592638 | -2.02552 |
| GDA | 2.313719618 | 5.22721 |
| GDF15 | -1.352831142 | -4.00668 |
| GGT4P | 1.001859282 | -8.44935 |
| GGT5 | 2.466682324 | 5.849265 |
| GHR | 2.945670285 | 5.916775 |
| GIPC3 | 2.771519869 | 2.192396 |
| GJD3 | -1.977223239 | -2.77303 |
| GLB1L3 | -3.258422719 | 2.934265 |
| GLDN | 3.870912543 | 3.721336 |
| GLI2 | -1.463378215 | -3.86468 |
| GLI3 | 4.526108752 | 5.580927 |
| GLIPR2 | 1.432054999 | 1.623416 |
| GLIS1 | 6.387940307 | 2.971794 |
| GLRB | -1.267732247 | -1.18373 |
| GLUL | -4.254439049 | 5.447533 |
| GM2A | 1.034285683 | 1.715487 |
| GNAO1 | 2.487875753 | 5.442215 |
| GOLT1A | -1.358944683 | -4.43533 |
| GPC2 | -1.490225601 | -1.61894 |
| GPC3 | 7.853216353 | 8.709906 |
| GPC4 | 2.459961055 | 6.161941 |
| GPC5 | 9.123097486 | 10.31462 |
| GPER1 | 2.335864903 | 4.489288 |
| GPNMB | 3.973153544 | 4.924617 |
| GPR137C | 2.130907608 | 3.820515 |
| GPR162 | -4.311602648 | 3.390723 |
| GPR176 | 1.473803522 | 1.441051 |
| GPR37 | 1.476397243 | 1.53396 |
| GPRC5B | -1.239115948 | -1.41013 |
| GPS2 | -1.148772248 | -1.0572 |
| GPX2 | 1.854549318 | -3.63579 |
| GRAMD1B | 1.211831978 | 2.071068 |
| GREM1 | -7.859398602 | -3.6438 |
| GREM2 | 1.038700127 | 5.281141 |
| GRIK2 | 1.367340639 | -5.13541 |
| GRIP2 | -1.442957867 | -1.8868 |
| GSEC | 1.479680743 | 1.107516 |
| HAGLR | 2.661577733 | 2.674258 |
| HAS3 | -1.35569819 | -3.44885 |
| HBQ1 | -1.045625706 | -1.29998 |
| HDAC4 | 1.298782233 | 2.606332 |
| HES2 | -1.615734027 | -1.59232 |
| HES6 | -1.24751822 | -1.85568 |
| HES7 | -2.068691284 | -1.50776 |
| HEY2 | 1.262650942 | 2.528912 |
| HIC1 | -1.383420175 | -1.79497 |
| HID1 | -1.471868406 | -2.52464 |
| HIST1H2BK | 1.003527933 | 1.410906 |
| HIST1H4I | 1.216596348 | 1.542324 |
| HKDC1 | -1.038361183 | -5.46863 |
| HLA-DMA | 1.321934066 | 4.074468 |
| HLA-DPA1 | 5.526130164 | 9.781534 |
| HLA-DRA | 3.64231713 | 9.416345 |
| HLA-F | 1.222424861 | 2.011032 |
| HMGA2 | -1.476009222 | -2.67244 |
| HOXA5 | 1.273440074 | 2.09569 |
| HOXB2 | -2.6224679 | -1.34163 |
| HOXD8 | 1.307608773 | 1.958001 |
| HPD | 3.691039848 | 1.420261 |
| HPGD | -1.303679717 | -6.18874 |
| HS3ST6 | -3.025433483 | -2.8271 |
| HSPA2 | 1.186274438 | 3.57088 |
| HSPA8 | 1.126009499 | 1.711276 |
| HSPB8 | 1.142334508 | 1.643676 |
| HTRA1 | 1.874596268 | 1.222644 |
| HYAL1 | 1.749694962 | 2.627581 |
| ICAM5 | -1.273326455 | -1.36 |
| ID4 | 1.616711977 | 2.118775 |
| IDI1 | 1.148947993 | 1.108492 |
| IER5 | -1.239772195 | -1.15675 |
| IFFO1 | 1.21468775 | -1.98616 |
| IFFO2 | -1.020360539 | -1.3951 |
| IFI16 | 2.631175959 | 2.756492 |
| IFI44 | 2.093420968 | 3.037474 |
| IFI44L | 2.779376581 | 6.223054 |
| IFITM10 | 1.254774219 | -1.08861 |
| IGDCC4 | -1.746112581 | -1.63238 |
| IGFBP5 | 2.740991786 | 8.573434 |
| IGFBP7 | 3.44085716 | 6.036724 |
| IGFBPL1 | -3.135973743 | -1.42942 |
| IGFL2-AS1 | -3.969677492 | -3.00018 |
| IGFN1 | 3.34280123 | -3.21545 |
| IGSF10 | 3.293021801 | 4.357947 |
| IL10RB | -1.708628226 | -1.89097 |
| IL13RA2 | 4.559370829 | 4.740934 |
| IL1R1 | 1.167182966 | 2.057302 |
| IL33 | 6.653266229 | 4.871798 |
| ILDR2 | -2.216313352 | 3.720675 |
| IMPA2 | 1.914016843 | 2.345922 |
| INA | -1.277837211 | -2.675 |
| INHBA | 6.808767951 | 4.564048 |
| INSIG1 | 1.655649925 | 1.558923 |
| INSL4 | -2.351958071 | 2.50793 |
| INSYN1 | 1.142118496 | 1.830699 |
| IQGAP2 | 1.481682935 | 2.75131 |
| ISYNA1 | -1.844729755 | -2.71571 |
| ITGA1 | 2.35304863 | 3.592388 |
| ITGA11 | 2.81798549 | 5.247778 |
| ITGA4 | 4.029776887 | 3.728773 |
| ITIH2 | 1.220392575 | -1.56918 |
| ITPKB | -2.603367464 | 1.44945 |
| ITPR1 | 2.506476551 | 4.27568 |
| ITPRIP | 1.823331062 | 1.432857 |
| JAG2 | -1.23662602 | -1.53109 |
| JAKMIP2 | 6.371256879 | 7.675253 |
| JAZF1 | 2.327026804 | 2.124544 |
| JPH1 | 3.03409496 | 5.404163 |
| KALRN | 2.675770986 | 2.906179 |
| KANK1 | 1.074536181 | 1.684437 |
| KANK3 | -1.664316621 | -1.76638 |
| KANK4 | 4.855329951 | 5.853798 |
| KAZN | 1.760719868 | 3.530806 |
| KCND2 | 4.830447711 | 8.870559 |
| KCNE4 | 6.333348954 | 2.759549 |
| KCNF1 | -1.259469692 | -1.50955 |
| KCNH3 | -2.41722517 | -1.6405 |
| KCNH4 | -1.241673235 | -2.32703 |
| KCNJ6 | 1.96377785 | 3.750771 |
| KCNJ8 | 2.376304295 | 6.290822 |
| KCNK5 | 1.266359901 | -1.30229 |
| KCNK6 | -1.306180753 | -2.67382 |
| KCNMA1 | 1.577524332 | 1.85227 |
| KCNQ1 | -1.668797961 | -2.88276 |
| KDELC2 | 1.635771081 | 1.693174 |
| KIF17 | -1.407575911 | -3.10299 |
| KIF21B | 1.517223979 | -1.3953 |
| KIT | 6.276042693 | 10.92805 |
| KLF12 | 1.95917353 | 2.123653 |
| KLF9 | 2.055646918 | 4.299936 |
| KLHDC7A | -1.317421318 | -4.89367 |
| KLHDC9 | -1.404710513 | -1.46761 |
| KLHL13 | 4.014026293 | 3.034947 |
| KLK2 | -2.748246161 | -5.60316 |
| KLRA1P | 1.286663489 | 1.593496 |
| KRT16 | -4.358316743 | -5.20846 |
| KRT19 | -9.272515389 | -6.37419 |
| KRT6A | -1.234355361 | -6.75897 |
| KRT84 | -6.741205612 | -4.25418 |
| KRTAP5-AS1 | -2.724651617 | -3.33115 |
| KSR2 | -1.449114701 | -3.01724 |
| L1CAM | 1.29532717 | 3.744359 |
| LAMA1 | 5.905721454 | 7.057264 |
| LAMA4 | 6.179455355 | 5.306444 |
| LAMA5-AS1 | -1.496858259 | -1.90294 |
| LAMP2 | 1.245449208 | 1.258685 |
| LAPTM4A | 1.099533996 | 1.249979 |
| LAT2 | -1.246048654 | -2.35576 |
| LCP1 | 2.964440303 | 4.677006 |
| LDLR | 1.321328559 | 1.497771 |
| LENG9 | -1.277148153 | -3.19398 |
| LGALS9 | 1.782379912 | 4.109515 |
| LGI4 | -2.821995137 | -4.9973 |
| LGR6 | -3.686493552 | -3.63586 |
| LHFPL6 | 1.616030323 | 1.938179 |
| LIFR | 1.841448574 | 1.527377 |
| LIMA1 | 2.856637057 | 2.716472 |
| LIMD2 | -1.391322181 | -1.29215 |
| LINC00304 | -1.410214565 | -3.06147 |
| LINC00452 | 3.182794119 | 4.473879 |
| LINC00488 | 2.002310202 | 3.040953 |
| LINC00511 | -1.353844073 | -2.24997 |
| LINC00565 | 3.543264111 | 5.022116 |
| LINC00601 | 3.226578493 | 3.792355 |
| LINC00607 | 2.903706924 | 2.71334 |
| LINC00632 | -1.822293518 | 1.831621 |
| LINC01116 | 2.080954142 | 2.632779 |
| LINC01145 | 1.129660955 | 1.62386 |
| LINC01433 | 1.284396683 | 2.03452 |
| LINC01583 | -1.25833615 | -2.79813 |
| LINC01693 | 3.618826127 | 3.283148 |
| LINC02274 | 4.448333386 | 5.457354 |
| LINC02532 | 1.572695742 | -2.82228 |
| LINC02593 | 1.071088382 | 1.195559 |
| LINC-PINT | 1.104708652 | -3.52812 |
| LITAF | 1.054632347 | 1.328845 |
| LMCD1 | -2.461704143 | -4.20141 |
| LMCD1-AS1 | -2.045353099 | -3.23011 |
| LMNTD1 | 4.812777457 | 5.589235 |
| LOXL1 | 2.251495273 | 2.01913 |
| LOXL3 | 1.017835915 | 1.54486 |
| LPAR6 | 1.180436182 | 3.394412 |
| LPCAT2 | 1.963957098 | 1.110163 |
| LRCH2 | 5.556376434 | 6.905326 |
| LRP1B | 5.27915514 | 5.939613 |
| LRRC10B | -4.794564534 | -3.83105 |
| LUM | 3.684015871 | 8.859742 |
| LXN | 1.162495518 | 2.750294 |
| LYPD6 | 2.230859049 | 4.164591 |
| LYPD6B | 6.743777172 | 10.15272 |
| LYPD8 | -2.711058112 | 1.402469 |
| MAFB | 3.004340542 | 6.389124 |
| MAGED1 | 1.352608137 | 2.160729 |
| MAMDC2 | 5.801028805 | 6.043736 |
| MAML2 | 1.750626779 | 1.144177 |
| MAN1A1 | 5.260031331 | 7.660453 |
| MANEA | 1.214899268 | 2.049033 |
| MAP2 | 2.45688982 | 4.849152 |
| MAP3K15 | -1.134766735 | -1.20675 |
| MAP7D2 | -2.201455865 | -2.56123 |
| MAPK15 | -1.092212099 | -1.91194 |
| MAPK4 | 6.789109007 | 7.186395 |
| MARCOL | 5.73925662 | 7.189472 |
| MARK1 | 1.056643944 | 1.942908 |
| MBL1P | 4.802743126 | 5.565053 |
| MBNL3 | 5.75412753 | 7.755149 |
| MCC | 3.384735044 | 4.162111 |
| MCF2L | -2.001167152 | 1.867757 |
| MDGA1 | 2.208281075 | 1.504531 |
| MECOM | 3.798971963 | 6.174545 |
| MEDAG | 5.689616302 | 6.890177 |
| MEF2C | 5.69475396 | 7.981881 |
| MEG3 | -1.151075749 | 1.055133 |
| MEG9 | -1.442226621 | 1.11268 |
| MEGF11 | -1.525581974 | -2.36225 |
| MESP1 | -1.275567992 | -1.83344 |
| METTL7A | 3.619223745 | 8.07198 |
| MGST3 | 1.145551048 | 2.059205 |
| MICAL2 | -1.189578391 | -4.0456 |
| MILR1 | 1.607610351 | 1.350188 |
| MIR99AHG | 1.129866949 | 2.021875 |
| MKX | 3.433243881 | 5.460353 |
| MLXIPL | -2.213993706 | -2.0759 |
| MME | 9.489756579 | 9.723098 |
| MME-AS1 | 4.647441888 | 5.413464 |
| MMP16 | 1.977857456 | 2.67714 |
| MMP2 | 5.004666207 | 5.298333 |
| MMP7 | -2.521826878 | -5.95981 |
| MNX1 | -1.285773627 | -2.18322 |
| MNX1-AS1 | -1.237462061 | -1.5178 |
| MOXD1 | 1.130376491 | 1.053345 |
| MPP1 | 1.354732989 | 1.132309 |
| MPP2 | -1.138100363 | -1.64153 |
| MPP7 | 2.206430908 | 4.666746 |
| MRPL12 | -1.072679198 | -1.27153 |
| MSX1 | 1.048297137 | 2.043716 |
| MTATP6P1 | 1.03765864 | 1.800147 |
| MTCL1 | -1.07098455 | -2.54445 |
| MT-ND5 | 1.101076521 | 1.30962 |
| MT-TT | 1.213082315 | 1.63434 |
| MTUS2 | 2.940847837 | 3.604029 |
| MUC13 | 1.737636674 | -1.54393 |
| MUC19 | -5.017028962 | -3.50404 |
| MUC5AC | -1.158199698 | -8.06368 |
| MYCL | -1.372798166 | 1.466473 |
| MYEOV | -1.628221409 | -7.16843 |
| NAALAD2 | 1.056248617 | 3.70518 |
| NAALADL2 | 1.946154761 | 2.72858 |
| NALCN | -1.222699643 | -1.76315 |
| NAMPT | 1.398156156 | 1.309796 |
| NAMPTP1 | 1.49521394 | 1.105358 |
| NAV1 | 1.521465394 | 1.475804 |
| NBPF4 | 2.239449987 | 2.069097 |
| NCAM1 | 3.703413888 | 6.9093 |
| NDNF | 5.143513672 | 7.677334 |
| NEGR1 | 8.162000212 | 9.065558 |
| NELL2 | 1.507594358 | 4.168015 |
| NFKBIE | -1.052811601 | -1.4152 |
| NGEF | -1.499963339 | -3.9176 |
| NGF | -2.354535584 | -3.36621 |
| NID2 | 2.835792102 | 2.864871 |
| NIPAL4 | -1.748382622 | -3.56893 |
| NKAIN4 | -1.768016364 | -1.77257 |
| NKPD1 | -1.319891987 | -1.89843 |
| NKX2-8 | -1.170138688 | -1.73395 |
| NLGN1 | 3.156783193 | 4.801084 |
| NLGN4Y | 2.433865203 | -1.42406 |
| NME2 | -1.331250471 | -1.3381 |
| NMRAL2P | 1.224955078 | 1.196634 |
| NOG | -1.86321925 | -2.07381 |
| NOS1 | -2.454436118 | 5.451873 |
| NOTCH3 | 2.812145093 | 5.346979 |
| NOV | 1.091410534 | 4.251135 |
| NOX5 | 2.167508793 | 2.532834 |
| NPAS1 | -1.248102923 | -1.6567 |
| NPAS3 | 3.692905092 | 4.610197 |
| NPBWR1 | 2.746364434 | 1.339137 |
| NPC2 | 1.209250877 | 1.323693 |
| NPL | 1.375687431 | 2.224667 |
| NPNT | 3.269136996 | 6.404157 |
| NPTX1 | 3.972461544 | 4.681518 |
| NPY1R | 2.380078357 | 5.157698 |
| NQO1 | 1.018439783 | 1.672323 |
| NR2E3 | -2.094903886 | -1.96156 |
| NR5A2 | -1.656960741 | -5.76987 |
| NRG1 | -1.099880927 | -2.40787 |
| NRG4 | 1.563507861 | 2.880459 |
| NRXN3 | -2.013247855 | 1.535832 |
| NTRK2 | 3.487580322 | 8.867231 |
| NTRK3 | -1.966140059 | -1.25277 |
| NTS | 3.64880049 | -1.34903 |
| NUDT16P1 | -5.798148946 | -4.79205 |
| NUP62CL | -1.409016815 | -1.4692 |
| OBSCN-AS1 | 1.121826534 | 1.96191 |
| OCLN | -1.578421807 | -3.44727 |
| OSR1 | 1.14457335 | 1.159372 |
| OSTM1 | 1.047091751 | 1.143697 |
| OTULINL | -1.577505781 | 1.256723 |
| OVCH1 | 3.636827637 | 3.743401 |
| OXCT1 | 1.849091576 | 3.149794 |
| OXTR | -1.199996348 | -3.22417 |
| P3H3 | -1.403672484 | 1.033422 |
| P4HA3 | 1.939396092 | 2.957763 |
| PAG1 | 4.221900967 | 6.292834 |
| PAK3 | 2.443804073 | 5.550861 |
| PALD1 | -1.433621271 | -1.87365 |
| PALMD | 5.420726688 | 4.359677 |
| PAM | 1.477740673 | 1.043674 |
| PANX2 | 1.403544526 | 1.608363 |
| PAPPA2 | 1.721942295 | 5.241393 |
| PAPSS2 | 2.09413401 | 2.864962 |
| PARD6A | -1.654833431 | -1.66044 |
| PAX8-AS1 | 1.234785298 | -2.43409 |
| PCBP3 | 2.627653545 | 3.36876 |
| PCDH10 | 3.003327771 | 6.306911 |
| PCDH18 | 6.660301275 | 10.38092 |
| PCDH7 | 4.066084924 | 5.014872 |
| PCDHA11 | -1.437548644 | -1.58999 |
| PCSK1 | 1.8103628 | 1.376452 |
| PCSK5 | 1.333363263 | 4.083464 |
| PDE10A | 1.262426687 | 2.505061 |
| PDE1A | 5.671082276 | 7.555271 |
| PDE3A | 1.117796677 | 1.62623 |
| PDE3B | 1.75549738 | 3.171492 |
| PDGFB | -3.065393922 | -3.36003 |
| PDGFRB | 2.927882801 | 5.069619 |
| PDGFRL | -1.521984689 | 1.775659 |
| PDLIM1 | -1.111785295 | -1.07882 |
| PELI2 | 1.23090302 | 3.48634 |
| PER1 | -1.202081704 | -1.08952 |
| PER2 | -1.492737034 | -1.61653 |
| PET117 | -1.06152712 | -1.07555 |
| PGBD5 | 6.174526788 | 5.078121 |
| PGM5P2 | -1.555576748 | -1.82922 |
| PGR | 2.383822327 | 5.134647 |
| PHOSPHO1 | -1.438297157 | -1.19443 |
| PI15 | 1.649234555 | 4.305538 |
| PID1 | 1.793562577 | 1.637779 |
| PIEZO2 | 3.230377221 | 2.506161 |
| PIP5K1B | 6.207727958 | 9.643919 |
| PITPNC1 | 2.505015993 | 1.718393 |
| PKDCC | 1.96118716 | 3.267665 |
| PKIA | 2.793390932 | 5.159502 |
| PLA2G16 | -1.067972093 | -2.28334 |
| PLA2R1 | 1.506921566 | 1.754269 |
| PLAG1 | 2.937429954 | 3.897194 |
| PLBD1 | 1.298148871 | -1.79865 |
| PLCB1 | 2.728466086 | 4.419253 |
| PLCH1 | -1.387846672 | -2.52461 |
| PLCH2 | -1.726806113 | -4.26282 |
| PLCL1 | 2.543393218 | 4.493325 |
| PLEKHA2 | 1.759712297 | 2.972747 |
| PLEKHA7 | -1.26017697 | -3.18394 |
| PLGLB1 | 1.693989377 | 1.593663 |
| PLS1 | -1.320759295 | -1.48677 |
| PLSCR4 | 1.410546655 | 2.084695 |
| PLXDC2 | 7.326085664 | 7.120467 |
| PODNL1 | 1.640479408 | 4.313198 |
| POSTN | 9.533117332 | 7.697453 |
| PPARA | 1.1047578 | 1.529873 |
| PPFIBP2 | -1.02489336 | -3.15076 |
| PPP1R14A | -1.720758956 | -5.08507 |
| PPP1R16B | -2.009000876 | -2.9202 |
| PRAG1 | -1.491885756 | -3.39316 |
| PRICKLE1 | -1.878552485 | -4.37643 |
| PRKCG | -1.894607175 | -3.35178 |
| PRKCZ | -1.046411419 | -1.44051 |
| PRKG1 | 2.414291019 | 3.792851 |
| PRKG1-AS1 | -1.304256429 | -2.99798 |
| PRODH2 | 2.887489135 | -4.72412 |
| PRR16 | 3.486902469 | 1.637507 |
| PRR29 | -1.801111523 | -4.63513 |
| PRR5L | 3.213202157 | 3.624206 |
| PRSS12 | 1.45013911 | 2.348454 |
| PRSS3 | -4.748701477 | -6.18811 |
| PRUNE2 | 4.521273268 | 5.389368 |
| PSD2 | 3.537353763 | 1.947752 |
| PTCHD4 | 2.693319335 | 3.169083 |
| PTGFR | 5.584823383 | 5.096933 |
| PTPDC1 | -1.13857315 | -1.27807 |
| PTPN6 | -1.28388496 | -3.64664 |
| PTPRD | -1.543664621 | 2.075975 |
| PTPRN2 | -1.364570639 | -5.69045 |
| PXDN | -5.29640856 | 5.440833 |
| PYCARD | -5.738494008 | -5.6952 |
| QPRT | 1.433597693 | 3.967888 |
| QRFPR | 2.182603971 | 3.91724 |
| RAB11FIP4 | -1.319184814 | -2.204 |
| RAB20 | 1.980620745 | 5.234751 |
| RAB24 | -1.073945898 | -1.0906 |
| RAB27A | 1.441840475 | 1.289032 |
| RAB27B | 1.048805466 | -1.35915 |
| RAB31 | 1.599069186 | 1.054298 |
| RAB3C | 5.251808681 | 9.151196 |
| RAB7B | 2.054330394 | 2.546332 |
| RAET1G | 1.987714033 | 1.593111 |
| RAP2B | -1.006879886 | -1.0305 |
| RARB | 2.490888168 | 2.029661 |
| RASA3 | 1.331881261 | 2.025499 |
| RASEF | -1.353142944 | -4.80978 |
| RASSF2 | -1.521574018 | -2.11067 |
| RASSF6 | 1.633102262 | 1.263186 |
| RASSF7 | -1.022411871 | -2.3048 |
| RASSF8 | 2.245994023 | 2.394 |
| RASSF8-AS1 | 1.562926451 | 1.882029 |
| RBFOX3 | -2.1206324 | -3.70855 |
| RBM11 | 5.253597027 | 6.167131 |
| RBMS3 | 1.08430698 | 2.471665 |
| RCOR2 | -2.766017162 | -1.2204 |
| REEP1 | 1.148278778 | 3.549052 |
| REEP2 | -1.916358201 | -2.22993 |
| RERG | 3.058667342 | 4.699857 |
| RETREG1 | 1.036741903 | 1.153917 |
| RFXAP | 1.033534261 | 1.337111 |
| RGL1 | 2.421746726 | 3.371469 |
| RGS4 | 3.984858274 | 6.666496 |
| RHCG | -1.937629486 | -4.25499 |
| RHOB | 1.874959008 | 2.816278 |
| RHOD | -4.08445914 | -3.8372 |
| RHOV | 1.810848249 | 4.384089 |
| RILP | -1.202831539 | -1.81548 |
| RINL | -1.957177445 | -3.0349 |
| RN7SL2 | -3.098394013 | -2.48315 |
| RN7SL417P | 2.80667411 | 2.634953 |
| RNF128 | 4.609949649 | 4.072283 |
| RNF182 | 4.105680685 | 4.167053 |
| RNF24 | 1.345362348 | 2.310892 |
| ROBO3 | -1.12595475 | -1.33463 |
| RORA | 4.137001756 | 4.081646 |
| RORB | 7.084658159 | 8.302269 |
| ROS1 | 1.251046022 | -2.25557 |
| RPRM | 4.050487181 | 9.105303 |
| RSPH1 | -1.679100439 | -2.83934 |
| RSPO2 | 2.817937098 | 7.913749 |
| RTN1 | 7.718170295 | 9.625324 |
| RXFP2 | 8.846978284 | 6.331683 |
| S100A9 | 1.490178867 | 4.4983 |
| S1PR1 | 2.781050234 | 4.762541 |
| SAGE1 | -6.853654246 | -6.81043 |
| SAMD11 | 1.416646281 | 1.534976 |
| SAMD5 | 1.764679672 | 2.516498 |
| SAMD9L | 2.324481289 | 1.924702 |
| SATB1 | 3.392075771 | 6.010684 |
| SBK2 | -1.894452539 | -5.55525 |
| SCD | 1.613728437 | 3.082505 |
| SCG2 | 7.605396279 | 8.849629 |
| SCN2A | 2.079856478 | 2.644478 |
| SCN4A | -1.902641153 | -4.19454 |
| SCN5A | -2.563084844 | 1.222733 |
| SDC2 | 1.822182443 | 6.767469 |
| SDK1 | 1.731301742 | 3.889169 |
| SDK2 | -1.723825965 | -5.24986 |
| SELL | 4.187408464 | 6.61015 |
| SEMA3E | 2.207002801 | 2.354773 |
| SEMA6D | 3.046336654 | 10.55524 |
| SESN3 | 1.703183831 | 4.057484 |
| SESTD1 | 1.019920988 | 1.585183 |
| SEZ6 | -2.52829819 | -5.66494 |
| SFRP1 | 1.539942156 | 4.139806 |
| SGK1 | 1.714066904 | 2.496462 |
| SGSM3 | -1.434030136 | -1.14112 |
| SH2D5 | -1.272511941 | -4.58964 |
| SH3BGRL | 1.153542812 | 1.650383 |
| SH3BP5 | 1.425136747 | 4.01259 |
| SH3GL2 | 4.436237509 | 7.534014 |
| SH3TC1 | -1.33522198 | 1.802741 |
| SH3TC2 | 1.358759891 | 1.773411 |
| SHC4 | 3.834522108 | 3.472022 |
| SHISAL1 | 3.077058679 | 3.346731 |
| SIK2 | 1.507350848 | 2.320086 |
| SLC12A8 | 1.997711794 | 2.280998 |
| SLC16A11 | -1.811744351 | -2.50441 |
| SLC16A14 | -2.016140162 | 4.542905 |
| SLC16A6 | 2.221854306 | 1.772677 |
| SLC22A3 | -1.203079936 | -3.95236 |
| SLC24A4 | -2.854073221 | -4.38855 |
| SLC27A2 | -1.231227553 | -1.28106 |
| SLC30A1 | 1.401765817 | 1.035952 |
| SLC30A3 | -1.9401061 | 1.55465 |
| SLC35F3 | -1.446532363 | -2.23528 |
| SLC38A6 | 1.076087014 | 1.07513 |
| SLC40A1 | 1.399871985 | 1.338357 |
| SLC43A2 | -1.363698311 | -1.06323 |
| SLC44A1 | 1.486095799 | 2.355497 |
| SLC45A1 | -1.832891907 | -4.72081 |
| SLC4A7 | -1.042840624 | -2.1039 |
| SLC51B | 1.207277036 | -4.48851 |
| SLC52A1 | -2.517163184 | -1.87036 |
| SLC7A7 | 2.83550292 | -1.31869 |
| SLC7A8 | 4.375014903 | 7.906963 |
| SLC9A3R2 | -2.674443353 | -2.29722 |
| SLC9A9 | 1.389935655 | 2.276607 |
| SLFN11 | -6.072714742 | 2.233995 |
| SLIT2 | 5.578122314 | 5.574796 |
| SLIT3 | 1.258273928 | 1.275529 |
| SMAD9 | 2.669488469 | 3.263989 |
| SMKR1 | -1.034692502 | -2.1532 |
| SMPX | 2.16616646 | 3.315778 |
| SNAI1 | 4.104883768 | 3.207938 |
| SNAP25-AS1 | 1.922792755 | 2.056314 |
| SNAP91 | 2.549630541 | 2.232435 |
| SNCA | 5.079836798 | 4.41613 |
| SNED1 | 1.965184035 | 5.037342 |
| SNX29 | 1.011953384 | 1.092621 |
| SORBS2 | 2.6907569 | 6.773135 |
| SORT1 | -2.132862821 | -1.60405 |
| SOX18 | 1.197542254 | 3.729026 |
| SOX2 | -3.206485829 | 3.438255 |
| SOX4 | -1.149474363 | -1.07286 |
| SPARC | 4.016328914 | 9.303971 |
| SPATA3-AS1 | -1.644723155 | -2.55026 |
| SPATA6L | -1.200826277 | -2.3515 |
| SPEF1 | -1.25087556 | -2.77008 |
| SPEG | 3.166647072 | 2.006342 |
| SPINK13 | 7.184035161 | 7.188003 |
| SPINK6 | 11.59966158 | 8.414024 |
| SPINT2 | -1.428469455 | -6.03301 |
| SPRY2 | -1.000099275 | -1.36405 |
| SPRY3 | 2.537088796 | 2.946623 |
| SPTA1 | 4.041233282 | 5.855845 |
| SPTBN5 | -1.113641772 | -1.84212 |
| SPTLC3 | 4.500738298 | 4.970977 |
| SPTSSA | 1.487982846 | 3.528362 |
| SRGN | 1.177935583 | 1.761239 |
| SRPX | 2.452004666 | 1.832622 |
| SRPX2 | 1.209171834 | 2.98279 |
| SSBP3 | 1.169614083 | 1.827822 |
| SSPN | 2.233446637 | 3.257699 |
| ST5 | 1.137207281 | 1.151064 |
| ST6GAL2 | 1.102661992 | -9.2865 |
| ST8SIA4 | -1.682491481 | 3.389888 |
| STAC2 | 2.018808133 | 5.859372 |
| STARD4 | 2.798277435 | 3.454794 |
| STARD4-AS1 | 1.473715107 | 2.679531 |
| STARD8 | 1.300472127 | 2.406789 |
| STC1 | 2.083322327 | 3.076423 |
| STK32A | 2.612726827 | 5.00829 |
| STXBP5L | 6.256230961 | 7.622398 |
| STYK1 | 1.389584763 | 1.551361 |
| SULT1A1 | 2.073376224 | 4.65992 |
| SULT1C2 | 2.522026638 | 2.306351 |
| SUSD5 | 1.480177118 | 3.825758 |
| SV2C | 7.139137168 | 9.353553 |
| SYCE1L | -1.470555921 | -1.32649 |
| SYNE3 | 1.540089612 | 1.374096 |
| SYNGR3 | -1.722235245 | -1.95712 |
| SYPL2 | 1.541518172 | 4.307028 |
| SYT1 | -1.272450783 | -3.40573 |
| SYT13 | -2.437979261 | -1.94496 |
| SYT14 | 4.051317552 | 3.978415 |
| SYTL5 | 4.099559248 | 2.28134 |
| TAF10 | -1.259385399 | -1.76154 |
| TBC1D3P1 | -2.401477507 | -3.94201 |
| TBC1D8B | 1.50846713 | 2.47754 |
| TBILA | -1.062619695 | -1.07961 |
| TBX22 | 5.716173928 | 9.060599 |
| TCAF1P1 | -1.0441764 | -1.28073 |
| TCHH | -2.611287551 | 1.649828 |
| TCIM | -1.13997634 | -2.40844 |
| TDO2 | 1.146756665 | 2.004271 |
| TENM1 | 2.474178084 | 7.36608 |
| TENM2 | 5.076482239 | 3.791295 |
| TENM4 | -4.959030228 | 2.177302 |
| TENT5A | 1.603951082 | 4.244005 |
| TENT5C | 2.031804724 | 5.774411 |
| TERT | -1.887440431 | -1.66829 |
| TFCP2L1 | -1.829172754 | -3.20901 |
| TFEC | 3.218986514 | 2.949293 |
| TFPI | 1.826445493 | 1.604083 |
| TGFBI | 2.137331386 | 1.94154 |
| TGM2 | 1.461365056 | -2.3104 |
| TIMP1 | 1.231358484 | 2.090808 |
| TIMP2 | 2.027283544 | 1.58489 |
| TINAGL1 | -1.397167974 | -5.31581 |
| TLCD2 | -1.035164734 | -1.67155 |
| TLE1 | -1.196623686 | -2.78749 |
| TLE4 | -1.350167713 | -2.63962 |
| TLL1 | 3.078311958 | 5.533277 |
| TM4SF19 | 1.701060644 | -2.35467 |
| TM4SF20 | 2.314622984 | -3.50339 |
| TM4SF4 | 3.182144038 | -8.51985 |
| TM7SF2 | -1.597722036 | -1.62461 |
| TMC5 | -1.960317505 | -3.68226 |
| TMC8 | -2.315602131 | -1.56663 |
| TMEM105 | -2.072967771 | -4.35082 |
| TMEM132D-AS1 | -2.591823074 | -3.17597 |
| TMEM155 | 3.765816784 | 4.877555 |
| TMEM156 | -1.214018297 | -6.43026 |
| TMEM160 | -1.029440624 | -1.04619 |
| TMEM215 | -4.744630834 | 3.856806 |
| TMEM37 | 1.632237446 | 3.001116 |
| TMEM45A | 1.653034264 | 2.580959 |
| TMOD1 | 2.90052405 | 3.474045 |
| TMTC1 | 7.10536809 | 6.947346 |
| TNC | 2.375770554 | 4.137507 |
| TNFRSF10D | 1.009912621 | 2.19153 |
| TNFRSF11B | 10.3539889 | 12.12581 |
| TNFRSF19 | 2.729258322 | 5.15711 |
| TNFSF13 | -1.073962013 | -1.59878 |
| TNIP3 | 1.919864514 | 1.989617 |
| TNK1 | -1.479731137 | -1.36119 |
| TNS1 | 3.960198804 | 5.498412 |
| TOR4A | -1.298101148 | -2.40617 |
| TPP1 | 1.159997169 | 1.062972 |
| TRABD2A | 2.178228583 | 5.386705 |
| TRAF1 | -1.071585877 | -2.08185 |
| TRAM1L1 | 1.362826031 | 1.994868 |
| TREM1 | 4.831845358 | 5.905725 |
| TRIM2 | 2.180842027 | 2.547294 |
| TRIM22 | 1.677657198 | 3.117424 |
| TRIM36 | -1.47260802 | -1.08375 |
| TRIM45 | 1.515504572 | 1.777807 |
| TRIM67 | -1.975614777 | -2.64007 |
| TRMT9B | 2.259360469 | 4.0805 |
| TRNP1 | -1.141201504 | -5.00598 |
| TRPA1 | 2.912260222 | 4.623614 |
| TRPC4 | 7.758773235 | 10.77872 |
| TRPM3 | 4.306862554 | 8.573649 |
| TRPM6 | 1.016426671 | 1.483673 |
| TRPS1 | 1.894185229 | 2.379363 |
| TSPAN15 | -1.380372844 | -2.5341 |
| TSPAN33 | -1.18649412 | -2.35488 |
| TSPAN5 | -1.72713088 | -1.35442 |
| TSPAN7 | 2.140475894 | 3.037959 |
| TTC39C | 1.026039884 | 1.581518 |
| TTC9 | -1.198496629 | -1.3695 |
| TXNIP | -2.413739633 | -2.97558 |
| UBA7 | 1.663732278 | 3.049922 |
| UBASH3B | -1.079030584 | -4.56109 |
| UBE2QL1 | -3.182576058 | -3.31544 |
| UBE2SP1 | 1.167540586 | 1.453437 |
| UFSP1 | -1.259644321 | -1.24497 |
| UGT1A1 | 2.054997915 | -4.71699 |
| UGT1A7 | 2.171721311 | -5.39713 |
| UGT2B7 | 1.263627489 | -5.67666 |
| UGT8 | -2.074468186 | 2.541018 |
| UNC13A | -1.22783889 | -1.31894 |
| UNC80 | 2.015516749 | 1.58543 |
| UPP1 | -1.012894635 | -1.73791 |
| USH1C | 4.715884414 | 4.279868 |
| USP2 | -3.152854858 | -3.32596 |
| USP44 | -2.300511548 | -3.33656 |
| USP51 | 1.258463599 | 3.128945 |
| USP53 | -1.252377142 | -2.49622 |
| UST | 1.683427253 | 3.037605 |
| VASH2 | 1.487270569 | 1.166119 |
| VAV1 | -1.406714662 | -5.24788 |
| VAV3 | 1.559130329 | 3.066401 |
| VCAN | 2.58006834 | 2.362903 |
| VEGFD | 1.634748007 | 2.839266 |
| VGF | -1.602627762 | 1.723966 |
| VGLL3 | 1.584576016 | 1.232087 |
| VSTM1 | -4.540688524 | -5.45964 |
| VWA5A | 1.856157089 | 4.701507 |
| VWA5B2 | -2.153155811 | -1.75015 |
| VWDE | -1.419781988 | -1.13959 |
| WASF3 | 4.69266677 | 5.154258 |
| WFDC21P | 2.697619547 | 1.17888 |
| WIPI1 | 1.710584506 | 1.800246 |
| WISP1 | 5.176144571 | 9.907897 |
| WISP2 | 1.772363118 | 1.413393 |
| WNT16 | 6.810069787 | 6.198633 |
| WNT5A | 4.852812634 | 4.114609 |
| WNT5A-AS1 | 3.272534597 | 3.524512 |
| WNT7B | -1.779375728 | -8.2116 |
| WSCD1 | -1.189068679 | 1.506409 |
| WT1-AS | -1.947399429 | -1.52374 |
| WTIP | -1.357910529 | -2.44755 |
| XKR8 | 1.093913815 | -5.34672 |
| XKRX | 1.443052505 | -2.09589 |
| YDJC | -1.053298638 | -1.93522 |
| ZEB2 | 1.833537031 | 1.127822 |
| ZFP3 | -3.035307694 | -1.99629 |
| ZFP30 | -2.57168699 | -4.36198 |
| ZFPM2 | 1.120510822 | 2.418853 |
| ZMIZ1-AS1 | 2.608934462 | 1.864883 |
| ZNF385C | -1.099395674 | -1.79978 |
| ZNF385D | 5.36104046 | 8.210953 |
| ZNF418 | -3.086826768 | -2.72517 |
| ZNF423 | 2.394357738 | 1.70635 |
| ZNF471 | -3.567352717 | -3.92875 |
| ZNF503 | 1.049167284 | 2.08022 |
| ZNF542P | -1.751536903 | -3.10973 |
| ZNF704 | 2.31803275 | 2.910453 |
| ZNF738 | -4.648989856 | -5.56721 |
| ZNF804A | 1.986336204 | 1.851064 |
| ZNF860 | 1.198300412 | 1.565079 |
| ZP1 | 5.356212076 | 2.947329 |
| ZSCAN18 | -2.735201706 | -5.69197 |

Table S3. The primary antibodies used in this study.

| Target | Source | ID | Supplier | Application |
| --- | --- | --- | --- | --- |
| Ezh2 | Rabbit | 5246S | Cell Signaling Technology | WB, IHC, IF |
| SUZ12 | Rabbit | 3737S | Cell Signaling Technology | WB |
| EED | Rabbit | 85322S | Cell Signaling Technology | WB |
| SFRP1 | Rabbit | 3534S | Cell Signaling Technology | WB |
| SFRP1 | Rabbit | 26460-1-AP | Proteintech | IHC |
| Flag | Rabbit | 14793S | Cell Signaling Technology | ChIP |
| PCNA | Mouse | ab29 | Abcam | WB |
| Beta Actin | Mouse | 66009-1-Ig | Proteintech | WB |
| H3K27me3 | Rabbit | 9733S | Cell Signaling Technology | WB, ChIP |
| β-catenin | Rabbit | D10A8 | Cell Signaling Technology | WB |
| Histone H3 | Rabbit | ab176842 | Abcam | WB |
| Histone H3 | Rabbit | 4620S | Cell Signaling Technology | ChIP |
| rabbit IgG | Rabbit | A7016 | Beyotime | ChIP |
| rabbit IgG | Rabbit | 30000-0-AP | Proteintech | IP |
| Cytokeratin 19 | Rabbit | ab52625 | Abcam | IHC |

Table S4. Primers for plasmid construction.

| Gene | Sequence |
| --- | --- |
| ShEZH2#1F | CCGGGAAACAGCTGCCTTAGCTTCACTCGAGTGAAGCTAAGGCAGCTGTTTCTTTTTG |
| ShEZH2#1R | AATTCAAAAAGAAACAGCTGCCTTAGCTTCACTCGAGTGAAGCTAAGGCAGCTGTTTC |
| ShEZH2#2F | CCGGCCAACACAAGTCATCCCATTACTCGAGTAATGGGATGACTTGTGTTGGTTTTTG |
| ShEZH2#2R | AATTCAAAAACCAACACAAGTCATCCCATTACTCGAGTAATGGGATGACTTGTGTTGG |
| ShEzh2#1F | CCGGGCACAAGTCATCCCGTTAAAGCTCGAGCTTTAACGGGATGACTTGTGCTTTTTG |
| ShEzh2#1R | CATGCAAAAAGCACAAGTCATCCCGTTAAAGCTCGAGCTTTAACGGGATGACTTGTGC |
| ShEzh2#2F | CCGGGCGTATAAAGACACCACCTAACTCGAGTTAGGTGGTGTCTTTATACGCTTTTTG |
| ShEzh2#2R | CATGCAAAAAGCGTATAAAGACACCACCTAACTCGAGTTAGGTGGTGTCTTTATACGC |
| oeEZH2-F | CGGATCGGGTTTAAACGGATCCGCCACCATGGGCCAGACTGGGAAGAAAT |
| oeEZH2-R | GCCCTCTAGACTCGAGCGGCCGAGGGATTTCCATTTCTCTTTCGATG |
| oeEzh2-F | GTGTGGTGGAATTCTGCAGATAGCCACCATGGGCCAGACTGGGAAGAAATC |
| oeEzh2-R | GATCAGCGGGTTTAAACCATGGAGGGATTTCCATTTCTCGTTCG |
| oeSFRP1-F | CGGATCGGGTTTAAACGGATCCGCCACCATGGGCATCGGGCGCAG |
| oeSFRP1-R | GCCCTCTAGACTCGAGCGGCCGCTTAAACACGGACTGAAAGGTGGGG |
| oeSfrp1-F | GTGTGGTGGAATTCTGCAGATAGCCACCATGGGCGTCGGGCGCAGCGCG |
| oeSfrp1-R | GATCAGCGGGTTTAAACCATGGCTTAAAAACAGACTGGAAGGTG |
| oeDNMT3a-F | CGGATCGGGTTTAAACGGATCCGCCACCAATGCCCGCCATGCCCTCC |
| oeDNMT3a-R | GCCCTCTAGACTCGAGCGGCCGCACACACGCAAAATACTCCTTCAG |
| oeDNMT3b-F | CGGATCGGGTTTAAACGGATCCGCCACCATGAAGGGAGACACCAGGCATC |
| oeDNMT3b-R | GCCCTCTAGACTCGAGCGGCCGTTCACATGCAAAGTAGTCCTTCAG |
| oeDNMT1-F | CGGATCGGGTTTAAACGGATCCGCCACCATGCCGGCGCGTACCGCCCC |
| oeDNMT1-R | GCCCTCTAGACTCGAGCGGCCGGTCCTTAGCAGCTTCCTCCTCC |
| EZH2-sg-F | CACCGAGAAGGGACCAGTTTGTTGG |
| EZH2-sg-R | AAACCCAACAAACTGGTCCCTTCTC |
| SUZ12-sg-F | CACCGGCTTCGGGCGGCAAATCCGG |
| SUZ12-sg-R | AAACCCGGATTTGCCGCCCGAAGCC |
| EED-sg-F | CACCGGAGGGAAGTGTCGACTGCGC |
| EED-sg-R | AAACGCGCAGTCGACACTTCCCTCC |
| P1-sg1-F | CACCGCTCTTGGCGTCTGCCCTAGT |
| P1-sg1-R | AAACACTAGGGCAGACGCCAAGAGC |
| P1-sg2-F | TCTCAGGAATTCGAGGTAGA |
| P1-sg2-R | AAACTCTACCTCGAATTCCTGAGAC |
| P2-sg1-F | CACCGTTCCTGAACCCCTGTTAGTT |
| P2-sg1-R | AAACAACTAACAGGGGTTCAGGAAC |
| P2-sg2-F | CACCGGTTCAGGAAGGCTGAAGCGA |
| P2-sg2-R | AAACTCGCTTCAGCCTTCCTGAACC |
| P3-sg1-F | CACCGTTCGCTCTTGTTGCCTAAGC |
| P3-sg1-R | AAACGCTTAGGCAACAAGAGCGAAC |
| P3-sg2-F | CACCGGGTCTCGAACTCCTGACCTC |
| P3-sg2-R | AAACGAGGTCAGGAGTTCGAGACCC |
| P6-sg1-F | CACCGGGCCCATCTACCCGTGTCGC |
| P6-sg1-R | AAACGCGACACGGGTAGATGGGCCC |
| P6-sg2-F | CACCGAAGTGTGACAAGTTCCCCGA |
| P6-sg2-R | AAACTCGGGGAACTTGTCACACTTC |
| P7-sg1-F | CACCGGCCGCTGGGTCCCGGTGCTG |
| P7-sg1-R | AAACCAGCACCGGGACCCAGCGGCC |
| P7-sg2-F | CACCGACGTGATTCCTAAGCGCGTC |
| P7-sg2-R | AAACGACGCGCTTAGGAATCACGTC |

Table S5. Primers for RT-qPCR.

| Gene | Sequence |
| --- | --- |
| Beta Actin-F | CATGTACGTTGCTATCCAGGC |
| Beta Actin-R | CTCCTTAATGTCACGCACGAT |
| Ezh2-F | TGCCTCCTGAATGTACTCCAA |
| Ezh2-R | AGGGATGTAGGAAGCAGTCATAC |
| EZH2-F | AATCAGAGTACATGCGACTGAGA |
| EZH2-R | GCTGTATCCTTCGCTGTTTCC |
| SUZ12-F | TTGCAGCTTACGTTTACTGGTT |
| SUZ12-R | GGAACTTGCCTTATTGGACAACT |
| EED-F | GTGACGAGAACAGCAATCCAG |
| EED-R | TATCAGGGCGTTCAGTGTTTG |
| SFRP1-F | GCTCAACAAGAACTGCCAC |
| SFRP1-R | CTTGTCACACTTAAGCATCTCG |
| DNMT3a-F | GTCATGTGGTTCGGAGACGG |
| DNMT3a-R | AGTGTCACTCTCATCGCTGTC |
| DNMT3b-F | AGGGAAGACTCGATCCTCGTC |
| DNMT3b-R | GTGTGTAGCTTAGCAGACTGG |
| DNMT1-F | AGAACGGTGCTCATGCTTACA |
| DNMT1-R | CTCTACGGGCTTCACTTCTTG |

Table S6. Primers for RT-qPCR used to validate the possible off-target genes for target methylation.

| Gene | Sequence |
| --- | --- |
| ABHD14B -F | TCACTGAGTGGCATGTACTCC |
| ABHD14B -R | GTTGGCAGCATTGATTTTGTCA |
| ARHGEF7-F | TGCTTTCAACGTACCTACGGC |
| ARHGEF7-R | GGCAACTTGGTGCATTCTTCTAA |
| PDGFB-F | CTCGATCCGCTCCTTTGATGA |
| PDGFB-R | CGTTGGTGCGGTCTATGAG |
| FERMT1-F | GCGTTGACCATCCCAATGAAG |
| FERMT1-R | ACCAAAGAGCAAAGTCTGACC |
| CELSR3-F | GGTCAGGGAGCCTATCTTCGT |
| CELSR3-R | CTGGACGCCGTGTTCAATC |
| SDK1-F | GAGTGGCAGACCTACTCCTC |
| SDK1-R | CTGTCCCCAATGTCGTTGGT |
| OR8G5-F | ACTTGCAGCTCCCACATCTC |
| OR8G5-R | AGGCTGTAGATCAGGGGGTT |
| ENAH-F | TCTATCACCATACAGGCAACAAC |
| ENAH-R | GCACAGTTTATCACGACCTGA |
| SGOL2-F | TAAAGCACAACAACAGGGCAT |
| SGOL2-R | AGGCGAAGAAATGTGTTCTCAAA |
| STARD3NL-F | TTGGTGGGCAATAGCGTTGA |
| STARD3NL-R | GCAGCACATAGCCAAAAGCC |
| ARSJ-F | TGGAGAACTACTATGTCCAGCC |
| ARSJ-R | TGATAGAATGTTGAAGTCCGGTG |
| KATNAL1-F | CAACAGGTTCGGCAGGAATTA |
| KATNAL1-R | GATTGGGACGCCTGATCTGAG |
| KIAA0319L-F | GAGGTAGTCCCAGTGACGTAG |
| KIAA0319L-R | CTGGAAATTGTAATCGCCTTGTG |
| TCIRG1-F | CATGGTCCTTGCGGAGAACC |
| TCIRG1-R | GCCGGTGTAGATGGAGAACAG |
| WDR6-F | AGTTCAGCTCCCTTCTTAGCC |
| WDR6-R | GCGGCCAAACGTAGTCCTC |
| PTPN3-F | TTGAGCACCATACGTTCTTTCAG |
| PTPN3-R | GTGTTCCGAGAGCCCATAGT |
| NELL2-F | GAGCTGAACAGCGAATGAATAGA |
| NELL2-R | AATTCTCGGTAGGTGGTTCCC |
| HIVEP3-F | AACTGGTTGACCCCATGAGAC |
| HIVEP3-R | GGACGAGGCACGAAGACTTT |
| LSS-F | GCACTGGACGGGTGATTATGG |
| LSS-R | TCTCTTCTCTGTATCCGGCTG |
| BARX2-F | TGAAGCACAGGAACCGAAAG |
| BARX2-R | CCGAAGAGGGTATTGGCAACT |
| MKNK2-F | AGTACGCCGTCAAGATCATTG |
| MKNK2-R | GGCACTGGTACAGCATCTCC |
| KIAA2012-F | CAAGACCCAGAGCCAAGGAG |
| KIAA2012-R | ACACAGCACTCAACCTCTCG |
| KLHL25-F | AGCCGCTATTTTGAGGCCAT |
| KLHL25-R | GCGATGCGTGAGGAGTAGG |
| OSM-F | CACAGACTGGCCGACTTAGAG |
| OSM-R | AGTCCTCGATGTTCAGCCCA |
| TAX1BP3-F | GCCGTGGTGCAAAGAGTTG |
| TAX1BP3-R | CCCTTGTCCGTCTTGTCTTCA |
| PRPF4-F | GTTGCTCCGGTCGTGAAGAAA |
| PRPF4-R | CCCTGCTTTAAGTCCGTCTTT |
| ODF2-F | GCGAGGAATGAAAGGGGACA |
| ODF2-R | CCTTCATGCGCTCGATGTTG |
| IGSF3-F | TTTGCGGTGCTCTGGTATGT |
| IGSF3-R | ATGCCTCTCAAACTGGAGCC |
| TRIM71-F | GTGACGGCGAGGCATAAGAAA |
| TRIM71-R | GGGCCAGTAGGATGTCTAGC |
| MRC2-F | GGAGCAGGAGCCTTTGATGT |
| MRC2-R | CACAGCTGGTCGGTTTGTTG |
| KLHL36-F | CTGTCCTTTACGCCCGACTT |
| KLHL36-R | TGAGCGGGAAGTGGATGTTC |
| SOST-F | ACACAGCCTTCCGTGTAGTG |
| SOST-R | CGGACACGTCTTTGGTCTCA |
| HECA-F | GAGAAGGACGACTACCAGAAGG |
| HECA-R | CTCTCCCACTCGTAGAAGCA |
| CCDC148-F | GAGCAAGAGCTATCAGAACAACA |
| CCDC148-R | GCAAAGTGTGATGCTGTCTGTA |

Table S7. Primers for Bisulfite sequencing PCR (BSP).

| Gene | Sequence |
| --- | --- |
| P1-F | TTTTTAGGGGAATGTTTGAAATTTA |
| P1-R | AAATCCATCACTCAAACACACATAC |
| P2-F | GTTTTAGTGTTGAGGGTTATTATGG |
| P2-R | ATTTCCCAACTAACAAAAATTCAAA |
| P3-F | TTGAATTTTTGTTAGTTGGGAA |
| P3-R | AACCTAAATCATACTTACAAACCCA |
| P4-F | TGGTTTTGTTTTTTAAGGGGTGTTG |
| P4-R | CAAACTTCCAAAAACCTCCRAAAAC |
| P5-F | GTTTTYGGAGGTTTTTGGAAGTTTG |
| P5-R | CAACTACTAACCTACTACTTCACCTC |
| P6-F | TTGGGTGTTTTTGTTTAATAAGAATT |
| P6-R | CCTTACCTTAAAACTTAAAAACTTC |
| P7-F | GAAGTTTTTAAGTTTTAAGGTAAGG |
| P7-R | TATTTAAAACRCAAAACTTTATTTC |

Table S8. Primers for ChIP-qPCR and Reverse-ChIP.

| Gene | Sequence |
| --- | --- |
| S1-F | TTCGAGGTAGAAGGTGGCA |
| S1-R | TCCATCACTCAGGCACACAT |
| S2-F | TGCAAGTATGATCCAGGCTC |
| S2-R | TTACTAGAACCAGACGCGGC |
| S3-F | GGGCTACAAGAAGATGGTGC |
| S3-R | AAGCCGAAGAACTGCATGAC |
| S4-F | TGTGCACGTGATTCCTAAGC |
| S4-R | TCAGGTATGGGAAAGGCGAA |

Table S9. sequences of the probes for Reverse-ChIP.

| Gene | Sequence |
| --- | --- |
| NC-Reverse-ChIP | TTGTGACCAGCGAATACCTGTT |
| SFRP1-Reverse-ChIP-1 | TTCTCAGGAATTCGAGGTAGAA |
| SFRP1-Reverse-ChIP-2 | CTTCCTGAACCCCTGTTAGTTG |
| SFRP1-Reverse-ChIP-3 | TTTCGCTCTTGTTGCCTAAGCT |
| SFRP1-Reverse-ChIP-4 | TTAAGTGTGACAAGTTCCCCGA |
| SFRP1-Reverse-ChIP-5 | CACGTGATTCCTAAGCGCGTCT |
| SFRP1-Reverse-ChIP-6 | TAAAGCGCAAGGCTTTGTTTCA |
