# Supplementary figures and images for: Targeting polycomb repressor complex 2‐mediated bivalent promoter epigenetic silencing of secreted frizzled‐related protein 1 inhibits cholangiocarcinoma progression
Source: Clin Transl Med. 2023 Dec 4;13(12):e1502. doi: 10.1002/ctm2.1502 (PMC10696163; doi:10.1002/ctm2.1502)

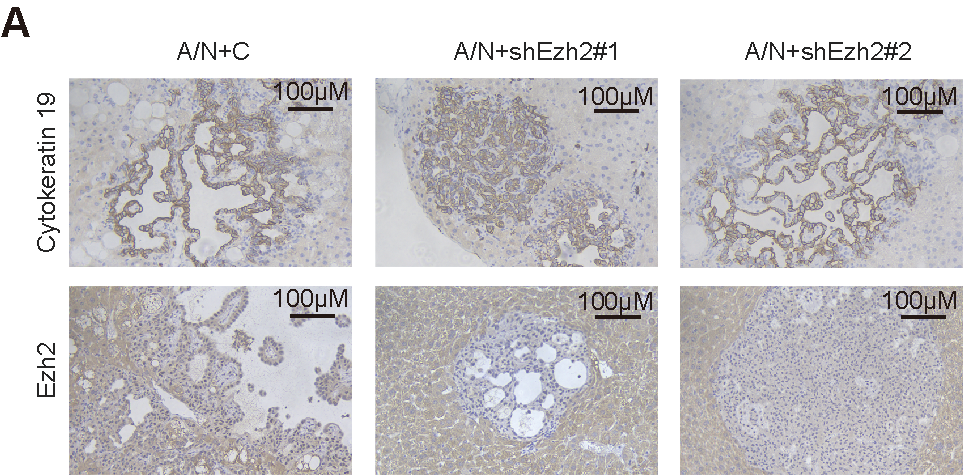

Supplement: Supplementary file 2 — FIGURE S1 (A) Immunohistochemical staining assay detected cytokeratin 19 and Ezh2 in the tumours. [file CTM2-13-e1502-s003.tif]

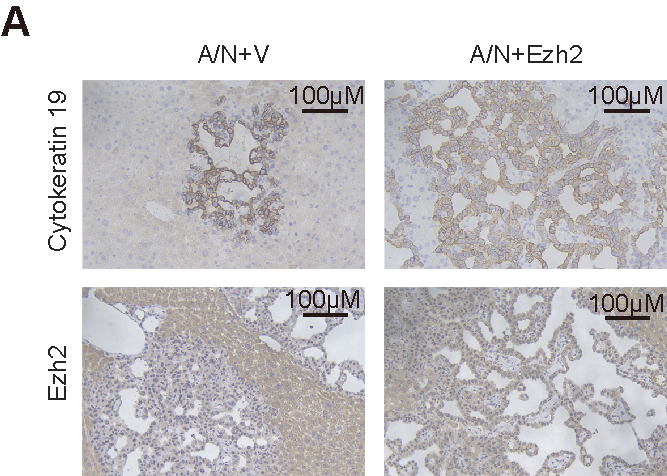

Supplement: Supplementary file 3 — FIGURE S2 (A) Immunohistochemical staining assay detected cytokeratin 19 and Ezh2 in the tumours. [file CTM2-13-e1502-s005.tif]

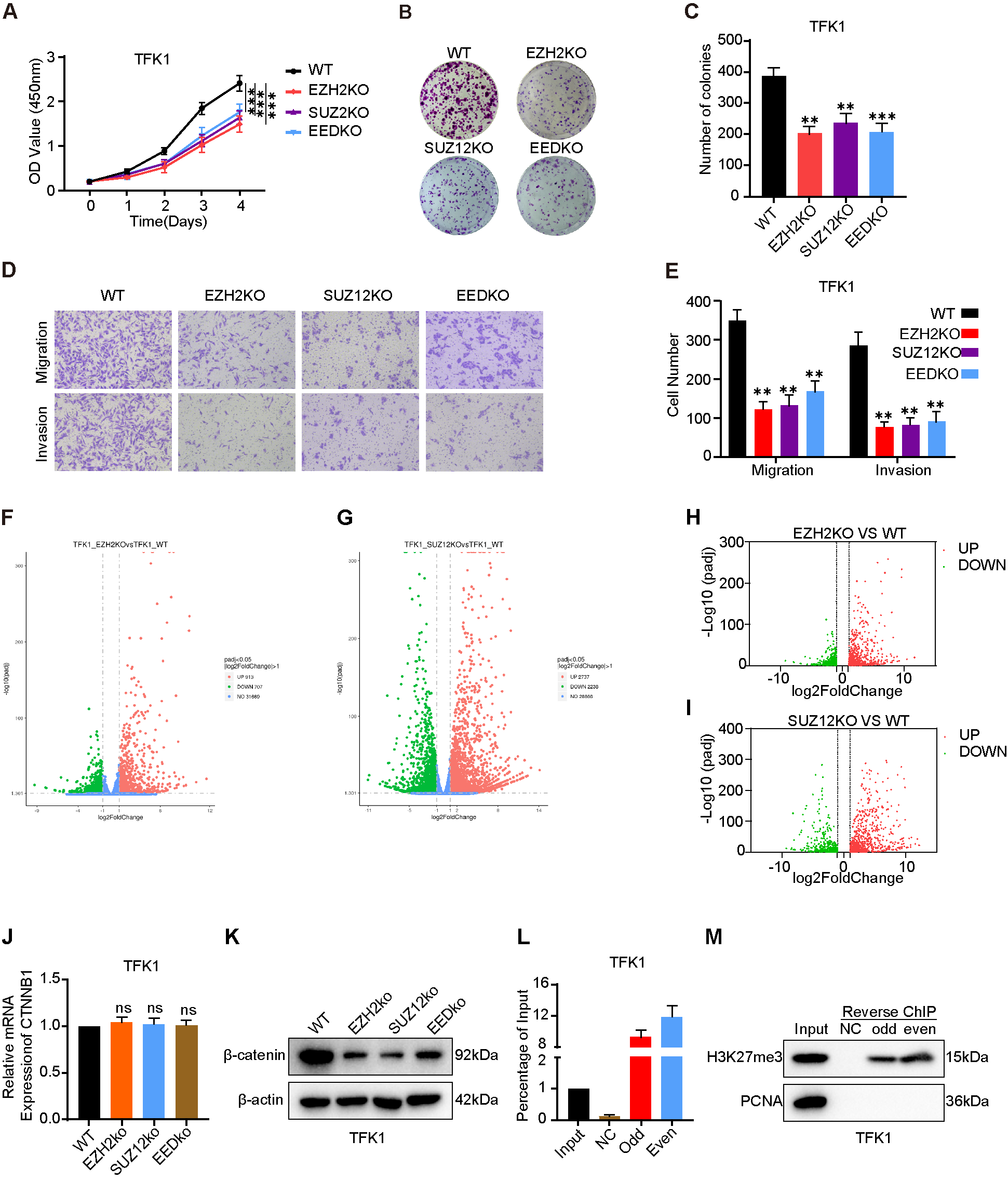

Supplement: Supplementary file 4 — FIGURE S3 (A) The proliferative ability of EZH2KO, SUZ12KO and EEDKO cell lines and WT cells was examined using the CCK‐8 assay. (B, C) The colony‐formation assay was performed on EZH2KO, SUZ12KO, EEDKO cell lines and WT cells (B: representative images. C: quantification data). (D, E) Cell migration assay and Matrigel invasion assay were conducted on EZH2KO, SUZ12KO and EEDKO cell lines and WT cells (D: representative images. E: quantification data). (F, G) Volcano plot showing the transcriptome analysis performed on TFK1 cells. F for EZH2KO and WT, G for SUZ12KO and WT. (H, I) Volcano plot showing the 1125 genes following EZH2KO and SUZ12KO. H for EZH2KO and WT, I for SUZ12KO and WT. (J, K) The relative mRNA expression (J) and protein level (K) of β‐catenin were measured using RT‐qPCR and Western blot assays after the knockout of EZH2, SUZ12 and EED in TFK1 cells. L‐M. To detect the distribution of H3K27me3 on the SFRP1 promoter, a Reverse‐ChIP assay was performed in TFK1 cells. The success of the Reverse‐ChIP assay in pulling down the SFRP1 promoter was verified through qPCR experiments (L), while the detection of proteins binding to the SFRP1 promoter was done using Western blot (M). [file CTM2-13-e1502-s007.tif]

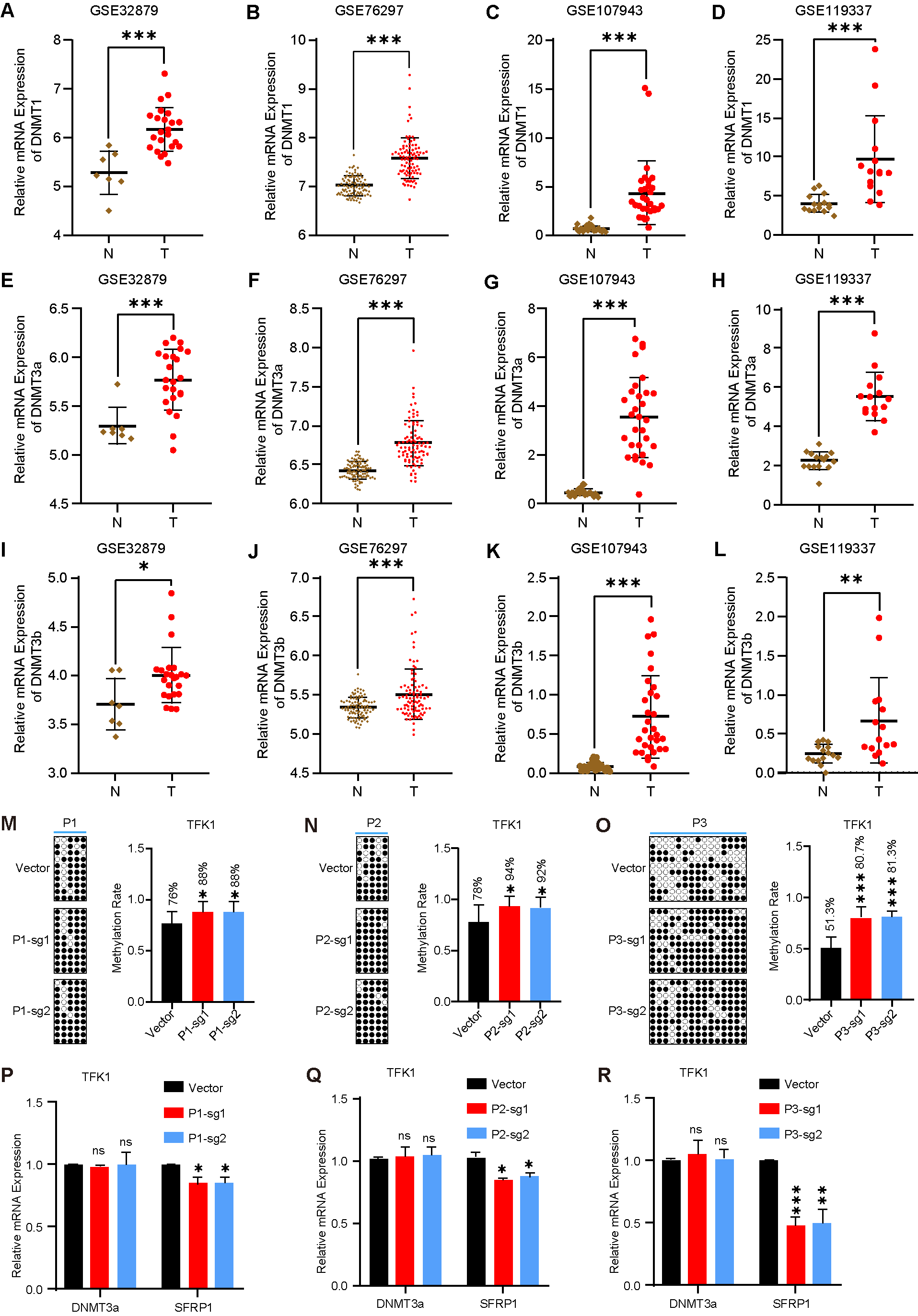

Supplement: Supplementary file 5 — FIGURE S4 (A–D) The expression level of DNMT1 in CCA and adjacent tissue from GSE32879, GSE76297, GSE107943 and GSE119337. (E–H) The expression level of DNMT3a in CCA and adjacent tissue from GSE32879, GSE76297, GSE107943 and GSE119337. (I–L) The expression level of DNMT3b in CCA and adjacent tissue from GSE32879, GSE76297, GSE107943 and GSE119337. (M–O) The BSP assay was performed on TFK1 cells transfected with vector and dCas9‐DNMT3a‐gRNA1(sg1), dCas9‐DNMT3a‐gRNA2(sg2) for 48 h. M represents P1‐sg1 and P1‐sg2, N represents P2‐sg1 and P2‐sg2 and O represents P3‐sg1 and P3‐sg2. (P–R) The expression levels of SFRP1 mRNA in TFK1 cells were examined using RT‐qPCR in the Vector and dCas9‐DNMT3a‐gRNA1, dCas9‐DNMT3a‐gRNA2 groups. P represents P1‐sg1 and P1‐sg2, Q represents P2‐sg1 and P2‐sg2 and R represents P3‐sg1 and P3‐sg2. [file CTM2-13-e1502-s008.tif]

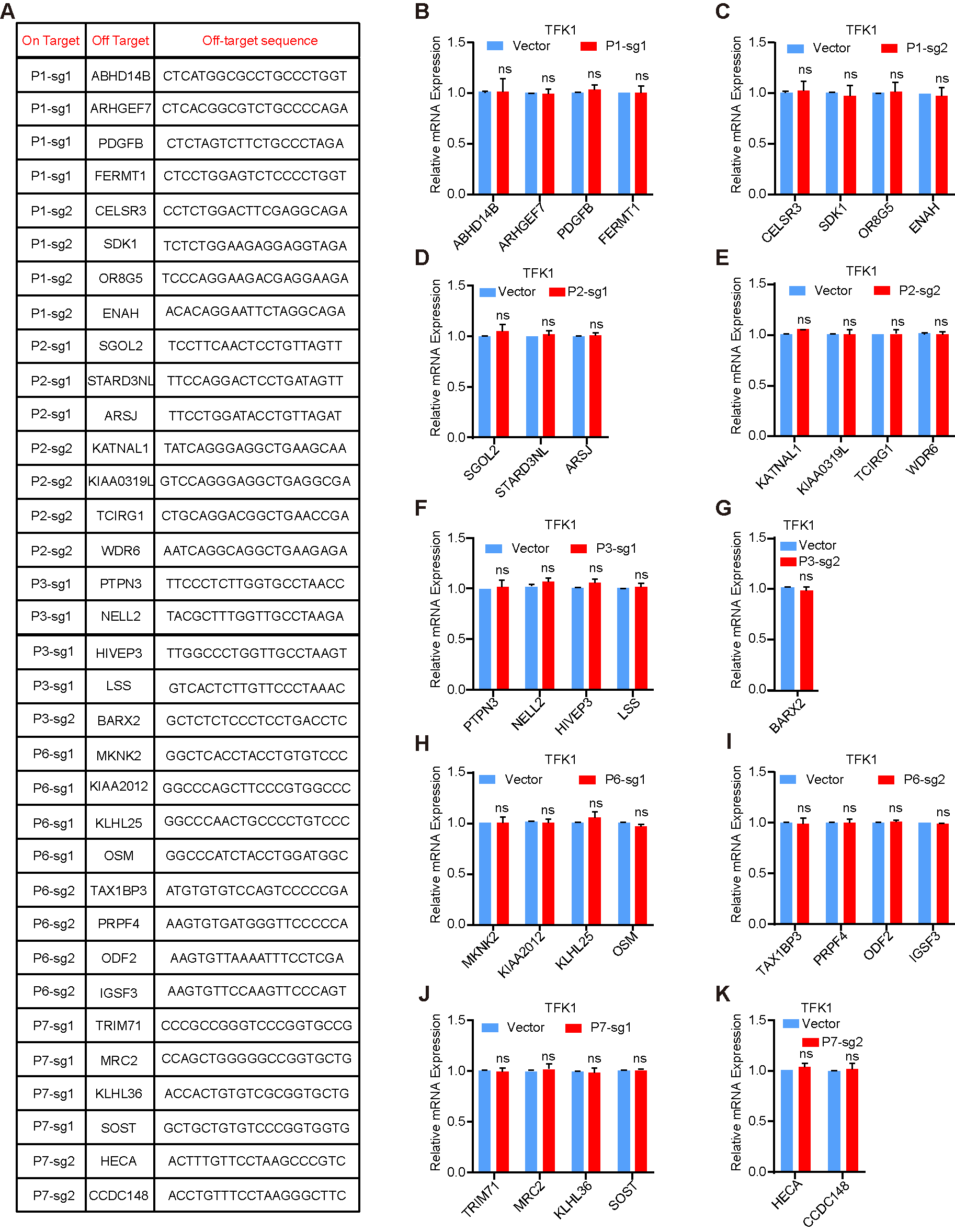

Supplement: Supplementary file 6 — FIGURE S5 (A) P1, P2, P3, P6 and P7 targeted methylated gRNA1 (sg1) and gRNA2 (sg2), selecting the four highest scoring off‐target gene sequences for each gRNA (individual off‐target sequences are less than four). (B–K) RT‐qPCR examination of the expression of off‐target genes targeted by methylated gRNA. B represents P1‐sg1, C represents P1‐sg2, D represents P2‐sg1, E represents P2‐sg2, F represents P3‐sg1, G represents P3‐sg2, H represents P6‐sg1, I represents P6‐sg2 and J represents P7‐sg1 and K represents P7‐sg2. [file CTM2-13-e1502-s006.tif]

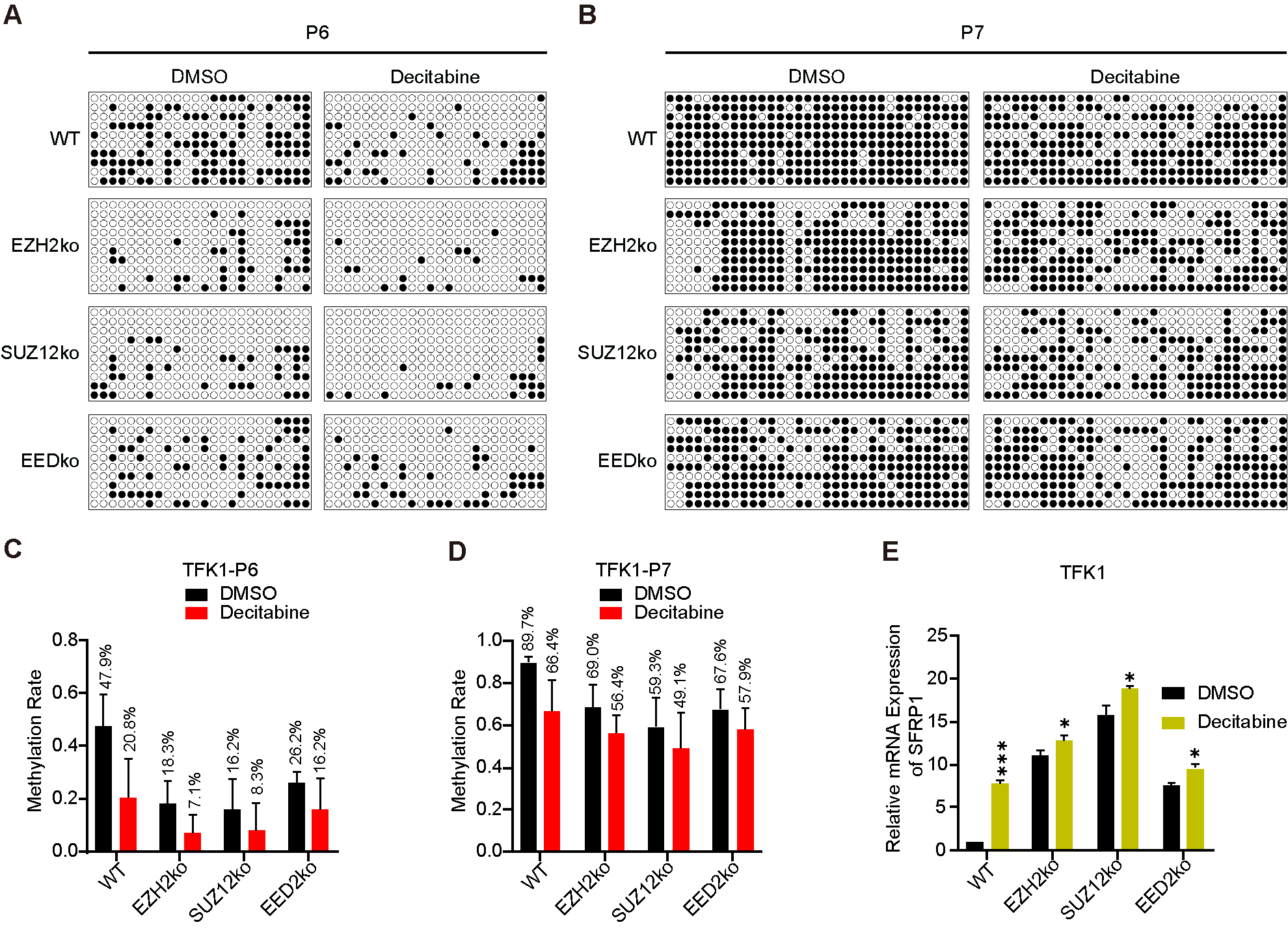

Supplement: Supplementary file 7 — FIGURE S6 (A–D) The SFRP1 promoter P6 and P7 BSP assay was performed on wild‐type, EZH2ko, SUZ12ko and EEDko TFK1 cells treated with 10 μM Decitabine for 72 h. (E) The expression of SFRP1 was tested by RT‐qPCR in wild‐type, EZH2ko, SUZ12ko and EEDko TFK1 cells treated with 10 μM Decitabine for 72 h. [file CTM2-13-e1502-s004.tif]

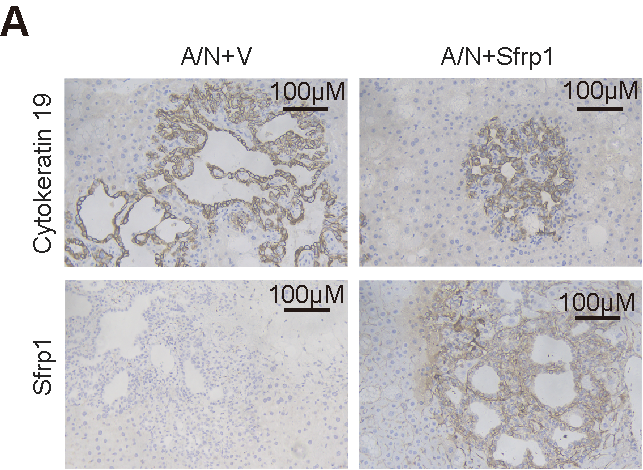

Supplement: Supplementary file 8 — FIGURE S7 Immunohistochemical staining assay detected cytokeratin 19 and Sfrp1 in the tumours. [file CTM2-13-e1502-s001.tif]

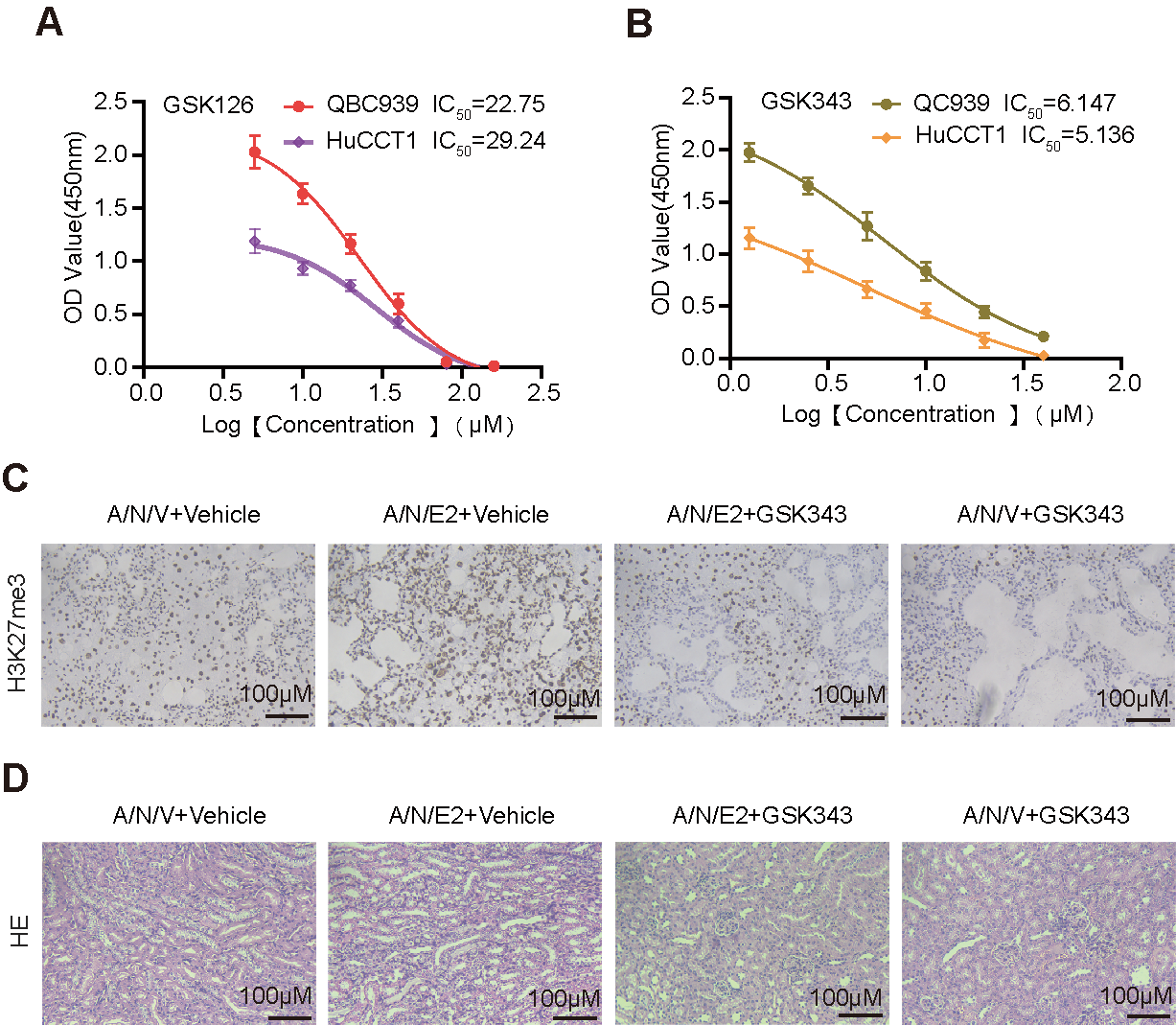

Supplement: Supplementary file 9 — FIGURE S8 (A, B) The IC50 values of GSK126 and GSK343 in QBC939/HuCCT1 cells. (C) Immunohistochemical staining assay detected H3K27me3 in the tumours. (D) The H&E‐staining of mouse kidney. [file CTM2-13-e1502-s002.tif]
